# 22nd Brazilian Diabetes Society Congress

**DOI:** 10.1186/s13098-019-0473-3

**Published:** 2019-10-16

## Oral presentations

### O1 A randomized controlled trial to compare the glycemic effects of dapagliflozin, a sodium glucose cotransporter 2 inhibitor, and gliclazide modified release, a sulphonylurea, assessed by CGMS

#### André Gustavo Daher Vianna, Claudio Silva de Lacerda, Luciana Muniz Pechmann, Michelle Garcia Polesel, Kleber Ramos Marques, Emerson Cestari Marino, Josiane Melchioretto Detsch, Claudia Pinheiro Sanches

##### Centro de Diabetes Curitiba, Curitiba, Brazil

*Diabetology & Metabolic Syndrome* 2019, **11(Suppl 1):**O1

**Introduction:** A reduced number of trials evaluated the effects of SGLT2 inhibitors on glucose pattern by CGM, but neither compared these effects with other class of antidiabetics. This is the first prospective and randomized study to compare the effects of dapagliflozin and an active comparator (gliclazide MR) on GV and glycemic control through the use of CGM parameters in patients with type 2 diabetes mellitus treatment naïve or not controlled on a stable dose of metformin.

Objective: This study aims to evaluate whether there is a difference between the effects of dapagliflozin and gliclazide MR (modified release) on glycemic variability (GV) and control, as assessed by continuous glucose monitoring (CGM), in individuals with uncontrolled type 2 diabetes (T2DM).

**Methods:** An open-label, randomized study was conducted in uncontrolled T2DM individuals drug naïve or on steady-dose metformin monotherapy which were treated with 10 mg dapagliflozin once daily or 60 to 120 mg of gliclazide MR once daily. CGM and GV indices calculation were performed at baseline and after 12 weeks.

**Results:** In total, 97 patients (age: 57.9 ± 8.7 years, male sex: 50.5%, baseline glycated hemoglobin (HbA1c): 7.9 ± 0.9) were randomized and 94 completed the 12-week protocol. Per protocol analysis demonstrated that the reduction of GV, as measured by the mean amplitude of glycemic excursions (MAGE), was superior in the dapagliflozin versus gliclazide MR group (− 17.8 ± 33.3 vs. − 3.3 ± 42.9 mg/dL, mean ± SD, p = 0.037). The improvement of GV, as measured by the coefficient of variation (CV) and the standard deviation (SD) was also superior in the dapagliflozin group (p = 0.021 and 0.024, respectively). Moreover, dapagliflozin increased the time in range (TIR, between 70 and 180 mg/dL) by 28.6 ± 24.4% vs. 19.8 ± 33.1% (p = 0.041). The intention-to-treat (ITT) analysis was also performed and did not alter the significance of the results.

**Conclusions:** Dapagliflozin reduced glycemic variability and increased the TIR more efficiently than gliclazide MR in individuals with T2DM after 12 weeks of treatment as demonstrated by continuous glucose monitoring evaluation.

**Financial support:** AstraZeneca do Brasil.

### O2 Association between pre-gestational BMI and adverse obstetric outcomes

#### Filipe Dias de Souza, Patricia Medici Dualib, Rosiane Mattar, Sergio Atala Dib, Bianca de Almeida Pititto

##### Universidade Federal de São Paulo, São Paulo, Brazil

*Diabetology & Metabolic Syndrome* 2019, **11(Suppl 1):**O2

**Introduction:** Previous studies have shown a higher incidence of obstetric complications in patients with increased body mass index (BMI). However, few studies provide details about the relation between obstetric complications and obesity degree.

**Objectives:** To compare occurrence of adverse obstetric outcomes in pregnant women with gestational diabetes mellitus (GDM) according to pre-gestational BMI.

**Methods:** 788 patients with GDM (IADPSG criteria) from a high-risk prenatal service between 2007 and 2019, were divided into five groups according to BMI: normal weight (18.5 to 24.5 kg/m^2^), overweight (25.0 to 29.9 kg/m^2^), grade I obesity (30.0 to 34.9 kg/m^2^), grade II obesity (35.0 to 39.9 kg/m^2^) and grade III obesity (≥ 40.0 kg/m^2^). Metabolic profile and occurrence of obstetric outcomes were compared between BMI groups using ANOVA or Chi square test. Obstetric outcomes included gestational hypertension (GHP), preeclampsia, c-section and others (hypothyroidism, pseudotumor cerebri, psychiatric disorders, thromboembolic events, HIV infection, dyslipidemia). Logistic regression was performed—preeclampsia and GHP as dependent variables.

**Results:** Mean age of the sample was 33.4 years and did not differ between BMI groups (p = 0.257). HbA1c levels increased (p = 0.006), while weight gain during pregnancy decreased (p < 0.001) progressively across the BMI degrees. Frequencies of previous GDM/macrosomia or preeclampsia were not different between groups (p = 0.51), but higher frequencies of prior hypertension were observed according to the increase in BMI (p < 0.001). Increase in frequencies of GHP (0.6 vs. 2.1 vs. 3.4 vs. 0 vs. 9.8%, p = 0.004) and preeclampsia (0.6 vs. 1.7 vs. 6.3 vs. 1.2 vs. 12.2, p < 0.001) were observed according to the progression of BMI degrees, respectively normal, overweight, GI, GII and GIII, while no difference was seen in occurrence of hypothyroidism, cesarean delivery or other maternal complication between groups. In logistic regression, BMI—used as independent variable as a continuous variable—was associated with preeclampsia (OR 1.12, 95% CI 1.04–1.20), adjusted for prior hypertension and weight gain during pregnancy, and GHP (OR 1.08, 95% CI 1.002–1.16) adjusted for weight gain during pregnancy.

**Conclusions:** Gestational hypertension and preeclampsia is positively associated with pre-gestational BMI in women with GDM independent of weight gain and metabolic profile.

**Financial support:** CAPES.

### O3 Bean consumption improves biochemical parameters and antioxidant capacity in female rats

#### Josilene Lopes de Oliveira^1^, Luiz Antônio Alves de Menezes Júnior^2^, Jonathas Assis de Oliveira^2^, Alice Helena de Souza Paulino^2^, Mayara Medeiros de Freitas Carvalho^2^, Ana Maria Fernandes Viana^2^, Joyce Ferreira da Costa^2^, Maria Lucia Pedrosa^2^

##### ^1^Universidade Estadual de Campinas, Campinas, Brazil; ^2^Universidade Federal de Ouro Preto, Ouro Preto, Brazil

*Diabetology & Metabolic Syndrome* 2019, **11(Suppl 1):**O3

**Introduction:** Oxidative damage caused by overproduction of reactive species is related to the pathogenesis of several diseases, such as diabetes, cardiovascular disease. The beans have shown numerous benefits beyond the supply of macro and micronutrients, highlighting the antioxidant activity.

**Objective:** To determine the in vitro antioxidant capacity of the three most consumed beans in Brazil and to verify if this effect is confirmed in vivo.

**Methodology:** The antioxidant capacity of carioca (C), black (B) and red (R) beans was determined by the DPPH method, the polyphenols by the Folin-Ciocalteu method. In the in vivo analysis, 32 wistar rats were used, divided into 4 groups: C (control), CB (C + 10% bean), H (hyperlipidic) and HB (H + 10% bean), they consumed the diets for 6 weeks. The blood and liver were used for dosing.

**Results:** Regarding antioxidant capacity, “C” (51%) presented lower value, when compared to “R” (81%) and “B” (87%), in the polyphenol quantification there was a direct correlation with antioxidant capacity. We attribute this result to the fact that “R” and “B” beans have a colored peel, the phenolic compounds, important exogenous antioxidants, are responsible for this coloration. Knowing the important antioxidant capacity of beans, we evaluated whether this profile results in antioxidant effect in vivo, since the in vitro study does not take into account the bioavailability of these compounds, their metabolism, tissue retention and cellular activity. In vivo, superoxide dismutase and catalase had a reduction in “HB” (3.8/138.5) when compared to “H” (5.4/195.6), suggesting that these enzymes were less likely to have had bean components contributing to antioxidant defenses, it is known that the activity of these enzymes is increased under oxidative stress. The bean also had a hypocholesterolemic effect, the “HB” group had a 40% decrease in serum cholesterol when compared to “H” among the possible mechanisms, we may mention the presence of soluble fibers, tannins, proteins and saponins. This legume also has a hypoglycemic effect, the “HB” (6.9) group presented lower glycemia in “H” (10.6) and may be associated with the presence of saponins and phytates. Or the presence of phaseolamin, which inhibits alpha-amylase, a starch glucose converting enzyme.

**Conclusion:** In addition to providing various nutrients, beans also have exogenous antioxidant function and can reduce cholesterol and blood glucose.

**Financial support:** CAPES.

### O4 Beneficial effect of using carbohydrate counting in type 1 diabetic patients stature: data from the largest Brazilian multicenter study of type 1 diabetes mellitus

#### Luana Matos de Souza^1^, Natasha Vasconcelos Albuquerque^1^, Milena Silva Sousa^1^, Ana Paula Dias Rangel Montenegro^2^, Virgínia Oliveira Fernandes^2^, Carlos Antônio Negrato^3^, Marília de Brito Gomes^4^, Renan Magalhães Montenegro Junior^2^

##### ^1^Programa de Pós Graduação em Saúde Pública-PPGSP/UFC, Fortaleza, Brazil; ^2^Universidade Federal do Ceará-UFC, Fortaleza, Brazil; ^3^Sociedade Brasileira de Diabetes-SBD, São Paulo, Brazil ^4^Universidade Estadual do Rio de Janeiro-UERJ, Rio de Janeiro, Brazil

*Diabetology & Metabolic Syndrome* 2019, **11(Suppl 1):**O4

**Introduction:** Inadequate glycemic control in children and adolescents with type 1 diabetes mellitus (T1DM) may interfere with growth. Thus, nutritional strategies that contribute to good control, such as carbohydrate counting, may be associated with adequate growth in this population.

**Objective:** To verify the impact of carbohydrate counting on the height of patients with T1DM.

**Methods:** This is a cross-sectional study, approved by the Ethics Committee (CPEA: 2.521.547), with participants from the Brazilian Type 1 Diabetes Study Group (BrazDiab1), conducted from 2008 to 2010. Participants aged < 19 years and with at least 12 months of follow-up in specialized services, were selected. Patients were categorized into two groups according to the use or no use of carbohydrate counting. For height analysis, the data were transformed into height-for-age Z score (Z H/A). Individuals with Z H/A < 0 were considered below average in relation to the general population. The glycated hemoglobin (HbA1c) collection method was defined according to the American Diabetes Association (2015) recommendations and considered as adequate glycemic control with HbA1c < 7.5%. Multiple logistic regression was performed to estimate odds ratios (OR) and 95% confidence interval (95% CI), with the Z H/A < 0 as the dependent variable, while the independent variable of interest was type of diet (use or no use of carbohydrate counting).

**Results:** A total of 1,441 participants were selected, from which 56% (807) were female, mean age was 12.1 ± 4.0 years and disease duration 5.0 ± 3.7 years. Only 34.6% (499) of the participants performed the carbohydrate counting technique. Regarding height, the mean Z H/A was 0.15 ± 1.21 in those who did carbohydrate counting and 0.02 ± 1.29 in those who did not (p = 0.39). Although both groups presented HbA1c values above the cutoff point, those who executed the technique, compared to those who did not, had significantly lower HbA1c values (9.1 ± 2.3 vs 9.5 ± 2.6, p = 0.00). In addition, there was a beneficial association between carbohydrate counting and Z H/A. Once, when the dependent variable was Z H/A < 0, it was found that individuals who performed the technique had a protection factor of 0.77 (95% CI: 0.60–0.97) in relation to the other participants.

**Conclusions:** Patients who performed carbohydrate counting had better glycemic control and lower chances of Z H/A < 0, suggesting a possible beneficial association between the technique and height in this population.

### O5 Chemotherapy treatment increases insulin resistance in breast cancer women

#### Jordana Carolina Marques Godinho Mota^1^, João Felipe Mota^2^, Raquel Machado Schincaglia^2^, Leonardo Ribeiro Soares^1^, Larissa Vaz Gonçalves^1^, Karine Anusca Martins^1^, Ruffo Freitas-Júnior^1^

##### ^1^Centro Avançado de Diagnóstico da Mama, Hospital das Clínicas, Federal University of Goiás, Goiânia, Brazil; ^2^Clinical and Sports Nutrition Research Laboratory (Labince), Federal University of Goiás, Goiânia, Brazil

*Diabetology & Metabolic Syndrome* 2019, **11(Suppl 1):**O5

**Introduction:** Chemotherapy is a type of systemic treatment commonly used in breast cancer with different adverse effects related to nutritional status. However, the side effects of chemotherapy on glycemic profile, according to the menopausal status and type of chemotherapy treatment still need to be better investigated.

**Objective:** To evaluate the impact of chemotherapy on glycemic profile of women with breast cancer according to the menopausal stage and type of chemotherapy.

**Methods:** This is a longitudinal study that included 99 newly diagnosed women in stage I to III of breast cancer during adjuvant or neoadjuvant chemotherapy. Glycemic profile was assessed in the first month and after the end of the last chemotherapy session by fasting glucose and insulin, homeostasis model assessment (HOMA) indexes, and quantitative insulin sensitivity check index (QUICKI).

**Results:** After chemotherapy, fasting insulin (9.62 ± 4.06 vs 13.37 ± 6.92 μU/mL, p < 0.001), HOMA-IR (2.37 ± 1.20 vs 2.83 ± 1.36, p = 0.01), and HOMA-B (136.05 ± 70.56 vs 162.34 ± 77.76, p = 0.01) were higher compared to the baseline. The quicki index decreased after the treatment (0.34 ± 0.03 vs 0.32 ± 0.02, p < 0.001). The adjuvant chemotherapeutic treatment was associated with greater increases in glycemia (9.82 95% CI: 3.27–16.37; p < 0.01). No differences were observed between women in pre and post-menopausal stage.

**Conclusions:** The chemotherapy treatment increased insulin resistance in women with breast cancer. The adjuvant chemotherapy has higher positive association with glycemia.

### O6 Comparison between the 2010 and 2018 EWGOP sarcopenia criteria in elderly with type 2 diabetes

#### Paula de Campos Calassara, Ana Luiza Nicoletti Dias, Paulo Sergio Chagas Gomes, Luciane Pires da Costa, Jefersson Teixeira, Roselee Pozzan, Lucianne Righetti Monteiro Tannus, Roberta Arnoldi Cobas

##### Universidade do Estado do Rio de Janeiro, Rio de Janeiro, Brazil

*Diabetology & Metabolic Syndrome* 2019, **11(Suppl 1):**O6

**Introduction:** The loss of muscle mass and strength/function related to aging, sarcopenia, represents a diagnostic challenge.

**Objectives:** the first purpose of the study was to compare sarcopenia prevalence in a sample of elderly subjects with type 2 diabetes, based on two different criteria of the European Working Group in Older People: EWGSOP—2010 and EWGSOP2—2018. The second purpose was to describe the clinical differences of patients classified according the each criterion.

**Methods**: a cross-sectional sample including elderly subjects with T2DM were submitted to the following testing protocol: muscle strength/function (sit-to-stand chair, get up and go, gait speed, handgrip strength, “Short Physical Performance Battery”), body composition (bioimpedance), clinical features, cognition and depression. The EWGSOP 2010 and 2018 (severe sarcopenia) criteria were applied and compared.

**Results:** A total of 77 patients were included, 46 (63%) women, age 73.8 ± 6.8 years, duration of diabetes 19.5 [10–25] years, glycohemoglobin (%) 7.9 ± 1.3%, BMI (kg/m^2^) 27.8 ± 4.6. Of the total, 37% did not meet any criteria for sarcopenia (subgroup 1), 38.4% only 2010 (subgroup 2), 9.6% only 2018 (subgroup 3) and 15.2% both criteria (subgroup 4). The 2018 criteria did not diagnose sarcopenia in men. Differences were observed when subgroups 1, 2, 3 and 4 were compared: Body mass index (kg/m^2^) = 29.6 ± 5.1 vs 27.4 ± 4.2 vs 29.5 ± 3.7 vs. 23.9 ± 2.8, p = 0.004); abdominal circumference (cm) = 100.9 ± 11.6 vs 101.4 ± 12 vs 103 ± 7.92 vs 89.95 ± 8.94, p = 0.023; glomerular filtration rate (CKD-EPI ml min^−1^ 1.73 m^−2^) 60.5 ± 16.6 vs 50.4 ± 18.7 vs 70.9 ± 16.8 vs. 59.4 ± 14.2, p = 0.013); presence of peripheral neuropathy (%) = 29.2 vs 73.1 vs 42.9 vs 20, p = 0.004; history of bone fracture (%) = 4 vs 14.8 vs 28.9 vs 45.5, p = 0.019; history of cardiovascular event (%) = 7.4 vs 10.7 vs 0 vs 45.5, p = 0.009; possible dementia (%) = 65.4 vs 63 vs 57.1 vs. 18.2, p = 0.049). There was no difference regarding age, duration of diabetes, A1c, blood pressure, retinopathy, history of fall and depression.

**Conclusion:** The EWGSOP 2018 criterion diagnosed sarcopenia in fewer patients compared to the 2010 version. The criteria compared were able to detect sarcopenia in patients, but with somewhat different clinical characteristics.

### O7 Comparison of glycated hemoglobin levels in type 1 diabetic patients with diabulimia

#### Luiza Costa Monteiro Hadler^1^, Raquel Oliveira Gomes^1^, Andreia Ribeiro de Carvalho Cavalcante^1^, Maria Claret Costa Monteiro Hadler^2^, André Neves Mascarenhas^3^, Isabela Fernandes Araujo^3^, Iago Barbosa Pinto Rodrigues^3^, Amanda Cristine Amador de Moura^3^

##### ^1^Unidade de Endocrinologia e Diabetes do Hospital Regional de Taguatinga (HRT), SES/DF, Brasília, Brazil; ^2^Universidade Federal de Goiás (UFG), Goiânia, Brazil; ^3^Escola Superior de Ciências da Saúde (ESCS), SES/DF, Brasília, Brazil

*Diabetology & Metabolic Syndrome* 2019, **11(Suppl 1):**O7

**Introduction:** Diabulimia is a specific diabetes eating disorder (ED) whose main feature is limiting and/or skipping insulin dosing and it is more likely to be presented among women with type 1 diabetes (T1DM). Worse metabolic control requiring hospitalization and higher risk to develop microvascular complications are frequent clinical issues related to those with ED compare to those without it. Moreover, the hypothesis that T1DM patients with diabulimia have divergent HbA1c levels compared to patients without ED has not yet been tested.

**Objective:** To compare the glycated hemoglobin (HbA1C) levels between T1DM patients who omitted insulin and those who did not.

**Methods:** A cross-sectional, observational and analytical study involving all patients diagnosed with T1DM1 who have been followed up at an Endocrinology and Diabetes United (UENDO) at a secondary level hospital, during 2 years (2016 to 2017).

A questionnaire was developed in order to evaluate socio-epidemiological information. Questionnaire included a question about omitted insulin doses focusing weight loss. The Mann–Whitney test was applied to compare medians among HbA1C levels between those who omitted insulin and those who did not. All data were analyzed with the SPSS—Statistical Package for Social Science version 18.0. Protocol was approved by local Ethics Committee.

**Results:** Original sample involved 138 eligible individuals, 26 refused to participate. Thus, final sample was 112 subjects aged 16 to 54 years (29.94 ± 9.30 years), there were 51 males, mean age 31.45 ± 10.05 years and 61 females (28.67 ± 8.5 years). Among those who answered the question about insulin omission (n = 110), 9.1% of patients reported omitting insulin doses for the purpose of weight loss, mainly were female (90%). Concerning median HbA1c, no difference between groups who omitted insulin and those who did not was found (p = 0.757).

**Conclusions:** The prevalence of diabulimia reported in the study was high, especially among female patients, a common previous finding. However, HbA1c levels were not higher in patients with diabulimia, despite ED patients have worse metabolic control. Other features need to be evaluated to clarify the present finding.

### O8 Diabetes and transplantation: what is the relationship to the gravity of peripheral neuropathy?

#### Geyse Gomes de Oliveira^1^, Emanuel Davi Simões dos Santos^2^, Edson Bruno Vidal de Sousa^2^, Camylla Bandeira Miranda^2^, Cristiany Azevedo Martins^1^, Hortência Diniz Teixeira^2^, Taciana Benevides Rocha^1^, Daniela Gardano Bucharles Mont’Alverne^2^

##### ^1^Hospital Universitário Walter Cantídio | Universidade Federal do Ceará, Fortaleza, Brazil; ^2^Universidade Federal do Ceará, Fortaleza, Brazil

*Diabetology & Metabolic Syndrome* 2019, **11(Suppl 1):**O8

**Introduction:** Posttransplant diabetes mellitus (DM) has been attributed to corticosteroid therapy. However, studies do not talk about the behavior of patients who were already diabetic before transplantation (TX). Does this population, when undergoing therapy after TX, worsen their condition, when compared with those who develop DM only after TX?

**Objective:** To compare, in patients with previous diabetes and in diabetic patients after transplantation, the incidence and severity of peripheral neuropathy.

**Methods:** A cross-sectional quantitative approach study was conducted in people with kidney or liver TX, regardless of sex and over 18 years old. All participants were evaluated for age, gender, time of TX, time since diagnosis of DM, last HbA1c value. The presence of diabetic peripheral neuropathy (DPN) was assessed by the Neuropathy Symptom Score (NSS) and Neuropathy Disability Score (NDS) in the lower limbs and the prayer sign test in the upper limbs. Results were expressed as mean ± standard deviation and/or percentage. For the comparisons, the t-test was used, being considered as statistically significant when p less than or equal to 0.05.

**Results:** One hundred and fifteen individuals who underwent liver or renal TX were evaluated, 53 of whom had DM before TX and 62 developed DM after TX. There was no statistical difference regarding gender (p = 0.325) and age (p = 0.120), and of the total, most were men (n = 64–55.7%) with a mean age of 57.8 ± 10.4 years old. When comparing the time since diagnosis of DM, a statistically significant difference was observed between the groups (p = 0.0001; 16.1 ± 7.8 years and 5.7 ± 3.6 years) and time since diagnosis of TX, being the group that developed DM after TX with the longest years of surgery (p = 0.001; 4.6 ± 4.4 years and 9.3 ± 5.9 years). There was also a difference regarding the NDS, and the group with previous DM had higher mean scores (p = 0.0001). When assessing the severity of DPN, it was found that patients who already had DM before TX had higher severity of DPN (p = 0.043), and 25% of them had a risk of ulceration while no patient in the DM group after TX had this risk. As for the sign of prayer, there was no difference between the two groups (p = 0.418).

**Conclusions:** A high incidence of DPN was observed in all participants, however people who had DM before TX had higher neuropathy disability score. This impairment was greater in the lower limbs than in the upper limbs.

### O9 Effects of aerobic, strength and combined training on expression of diabetic mice heart contractible proteins

#### Jonathan Nícolas dos Santos Ribeiro, Patricia Luana Barbosa da Silva Ribeiro, Bruno de Melo Carvalho, Stéfani Mendes da Silva, Fátima Lúcia Rodrigues Guimarães, Ruy Bandeira de Vasconcelos Júnior, Amanda Mota Vieira, Denise Maria Martins Vancea

##### Universidade de Pernambuco, Recife, Brazil

*Diabetology & Metabolic Syndrome* 2019, **11(Suppl 1):**O9

**Introduction:** Diabetic cardiomyopathy is characterized by ventricular diastolic/systolic dysfunction resulting from a malfunction of the proteins responsible for maintaining calcium (SERCA2a, PLP, FKBP 12.6) thus reducing the contractile performance of the heart, and physical exercise is one of the methods to reduce the progress of cardiomyopathy in diabetes.

**Objective:** To analyze the effects of aerobic, strength and combined training on the expression of contractile proteins in the heart of diabetic mice.

**Methods:** The study was approved by the Animal Use and Ethics Committee (No. 002/2018) and conducted with 22 male Swiss mice randomly randomized into five groups: Sedentary Normoglycemic Group (SNG, n = 5), Sedentary Diabetic Group (SDG, n = 5), Diabetic Aerobic Group (DAG, n = 4), Diabetic Strength Group (DSG, n = 4) and the Diabetic Combined Group (DCG, n = 4). Induction of type 1 diabetes mellitus in the SDG, DAG, DSG and DCG groups was performed by intraperitoneal administration of *streptozotocin* (40 mg/kg). Exercise protocols were performed three times a week for 8 weeks. The Aerobic Training (AT) was performed in mouse specific pools, with weight overload of 1% to 3% of the animals body weight. The Strength Training (ST) was performed in a rodent climbing apparatus, with weights attached in the syrup, with progressive overload from 50% to 100% of the maximum climbing. The combined training used the TA system plus the characteristics of the TF. Cardiac proteins: FKBP 12.6, SERCA2a, PLB; were obtained by extraction of the heart still in contraction and later analyzed by western blotting. Statistical analysis was performed using the one way ANOVA test together with their respective Bonferroni post hoc test and the paired student t test, always adopting the significance level of p ≤ 0.05.

**Results:** The analysis of protein expression of FKBP 12.6 (DGA = 9.5%/DSG = 32% and DCG = 33%) and PLB (DGA = 21.9%/DSG = 38.3% and DCG = 77.0%) showed significant increases (p = 0.00) for the use of combined training. The total concentration of SERCA2a showed a significant increase (p = 0.00) in DGA (40.714 ± 1206) when compared to DSG (14.746 ± 4158) and DCG (22506 ± 1542).

**Conclusions:** Aerobic, strength and combined training were effective in the modulation of cardiac proteins in diabetic mice, with PLB and FKBP 12.6 proteins being more expressed in combined training and SERCA2a presenting a greater expression in aerobic training.

### O10 Epigenetic regulation by micrornas in pathophysiology of diabetic cardiomyopathy

#### Mariana Borges Lopes^1^, Renata Caroline Costa de Freitas^2^, Flávio Santos Silva^3^, Bento José Abreu^4^, Adriana Augusto de Rezende^1^, Gabriela Placoná Diniz^5^, Raul Hernandes Bortolin^2^, André Ducati Luchessi^1^

##### ^1^Department of Clinical and Toxicological Analyzes, UFRN, Natal, Brazil; ^2^Department of Clinical and Toxicological Analyzes, USP, São Paulo, Brazil; ^3^Department of Health Sciences, UFERSA, Mossoró, Brazil; ^4^Life Science Center, Morphology department, UFRN, Natal, Brazil; ^5^Institute of Biomedical Sciences, Anatomy department, São Paulo, Brazil

*Diabetology & Metabolic Syndrome* 2019, **11(Suppl 1):**O10

**Introduction:** Diabetic cardiomyopathy (DCM), a recognized disease that affects cardiomyocytes, is a complication caused by chronic hyperglycemia and has high rates of mortality and morbidity in diabetic patients. However, despite medical advances in DCM, the molecular basis of this complication is not fully elucidated. In this context, the present study intends to validate the prediction regarding messenger RNAs (mRNAs) and their possible regulatory miRNAs with the molecular mechanisms involved in development of DCM through in vitro and in vivo approaches.

**Methods:** Myoblast lineage was cultivated under normoglycemic (NG, 5.5 mmol/L of glucose) and hyperglycemic (HG, 25 mmol/L of glucose) conditions for 24 h, after was performed total RNA extraction by Trizol reagent and gene expression analyzes using real time PCR (RTqPCR). Wistar rats used in in vivo model had diabetes pharmacologically induced by streptozotocin (40 mg/kg, i.v.; n = 7) than was compared with control group (citrate buffer, n = 7), after 30 days from induction the animals was euthanized, the DCM was characterized and their left ventricles was used for total RNA extraction by Trizol Reagent, mRNA and miRNA expression was analyzed by RTqPCR. The protocols used in this study were approved by the ethics committee at the Federal University of Rio Grande do Norte (017/2009).

**Results:** *Pla2g2a* expression was upregulated on the online datasets evaluated in our previous study using in silico strategies to predict gene expression in DCM, it result was also observed in the H9c2 cell culture under hyperglycemic conditions (3-fold increased, p-value = 0.043) and in left ventricles of diabetic rats from the in vivo study (3-fold increased, p-value = 0.004). *miR*-*214* was reduced in the databases analysis and also in the in vitro (p-value = 0.043) and in vivo (p = 0.025) studies. Furthermore, there was a tendency for decreased expression of *Hk2* in H9c2 cell culture under hyperglycemic conditions, and significant decrease in diabetic rats compared to their respective controls (p-value = 0.010). *miR*-*187* increased 3-fold in diabetic rats as well as in the H9c2 culture (p-value = 0.028 and p-value = 0.019, respectively) and *miR*-*17* was decreased (p-value = 0.015).

**Conclusions**: The experimental data performed in this study, validate the result of our *in silico* study previously published, as well as ratify prediction of these genes to participate in the pathophysiology of DCM.

**Financial support:** CAPES, CNPQ, FAPESP.

### O11 Evaluation of insulin application by diabetic patients

#### Marielle De Araújo Melo, Dandara Sampaio Leão De Carvalho, Isabella Carvalho Oliveira, Raíssa Carneiro Rezende, Guilherme Borges De Andrade, Daniela Espindola Antunes, Estela Muszkat Jatene, Daniela Pultrini Pereira De Oliveira Viggiano

##### Universidade Federal de Goiás – UFG, Goiânia, Brazil

*Diabetology & Metabolic Syndrome* 2019, **11(Suppl 1):**O11

**Introduction:** Insulin application is the therapy used in patients with Type 1 Mellitus Diabetes and a lot of attention is needed when it is used. The inadequate use and insecurities in the insulin auto-application can interfere in an adequate disease control.

**Objective**: To evaluate insulin application by patients and the connection with glycemic control.

**Methodology:** A cross-sectional study with 95 individuals with T1MD accompanied by the Endocrinology department in a university hospital. The data was collected between April and September in 2018 with the application of a standard questionnaire according to the Brazilian Diabetes Society published in 2017. The variables used were age, gender, schooling, the application object, knowledge on how to apply it, storage, expiration date, complications when applying it and glycated hemoglobin.

**Results:** From the 95 patients, 62.1% were women, 36.8% were in high school with 28.4% aged between 21 and 30 years old. From the sample, 67.4% were using the insulin pen. In this group, 40.6% showed number of application errors bigger then 7, while the percentage was higher in the disposable syringe (74.2%; p = 0.008). Most of the patients using the pen (89.6%) and syringe (93.5%) made the application in different parts of the body, properly. When it comes to sanitation, the patients that were using the pen showed the worst habit: 57.8% wash their hands, 25% clean the insulin bottles and 29.7% clean the skin versus 93.5%, 51.6% and 61.3% from those who used the syringe, respectively. About the storage of the insulin and the patients who were using the pen: 79.7% knew the ideal temperature for the insulin bottle, 73.4% knew the right place to store it but only 54.7% knew the expiration date when the insulin bottle is opened versus 90.3%; 80.6% and 51.6% from those who were using the syringe, respectively. In relation with complications like lipodystrophy, hypoglycemia and hyperglycemia, the proportion for the use of the pens was 57.8%, 56.4% and 42.2% versus 48.4%; 71% and 41.9% in syringes, respectively. The needles reutilization for more than 5 times was verified in 32.8% of the patients who used the pen and 31% with the syringe, with a bigger risk association of lipodystrophy (p = 0.014). The results didn’t show a statistical significance with glycated hemoglobin.

**Conclusion:** The results showed the importance of an intensive and continued education in diabetes for the conduction of a safe treatment and complications reductions.

### O12 HLA in patients with type 1 diabetes from an admixed population: a nationwide study in Brazil

#### Deborah Conte Santos^1^, Marcela Haas Pizarro^1^, Bianca Senger Vasconcelos Barros^1^, Laura Gomes Nunes de Melo^1^, Luiza Harcar Muniz^1^, LuisCristovão Porto^2^, Dayse Aparecida Silva^3^, Marilia Brito Gomes^1^

##### ^1^Universidade do Estado do Rio de Janeiro, Departamento de Medicina Interna, Diabetes, Rio de Janeiro, Brazil; ^2^Universidade do Estado do Rio de Janeiro, Laboratorio de Histocompatibilidade e Criopreservação (HLA), Rio de Janeiro, Brazil; ^3^Universidade do Estado do Rio de Janeiro, Laboratorio de Diagnostico DNA (LDD), Rio de Janeiro, Brazil

*Diabetology & Metabolic Syndrome* 2019, **11(Suppl 1):**O12

**Introduction:** HLA region on chromosome 6p21 is known to be responsible for almost 50% of the genetic risk and it has been studied for decades. The highest prevalence of T1D is observed on Caucasian population and most of the studies are concentrated on populations with small degrees of miscegenation. However, previous data show that the frequency of HLA haplotypes, and their effects on T1D risk or protection varies among populations. There is scarcity of data on the genetics of T1D population in Brazil.

**Objective:** In this study, we aimed to evaluate the HLA class II genetic profile of T1D patients from all the five regions of the country.

**Methods:** This was a nationwide multicenter cross-sectional study conducted between August 2011 and August 2014 in 14 public clinics located in 10 Brazilian cities in five geographical regions (North, Northeast, Midwest, Southeast and South). For the present study, we included 1019 type 1 diabetes patients and 5116 controls pared for region of birth and self-reported color/race. Control individuals belonged to the bone marrow transplant donor’s bank of Brazil (REDOME), which is the largest HLA data repository in the country. The study was approved by each center’s local ethics committee. HLA-class II alleles (DRB1, DQA1 and DQB1) were genotyped using the SSO and NGS method.

**Results:** The most frequently risk haplotype found in our population were DRB1*03:01 ~ DQA1*05:01 g ~ DQB1*02:01 (OR 5.8, p < 0.00001), DRB1*04:05 ~ DQA1*03:01 g ~ DQB1*03:02 (OR 5.34, p < 0.00001), DRB1*04:02 ~ DQA1*03:01 g ~ DQB1*03:02 (OR 3.43, p < 0.00001). Most prevalent protection haplotypes were DRB1*07:01 ~ DQA1*02:01 ~ DQB1*02:02 (OR 0.54, p < 0.0001), DRB1*13:01 ~ DQA1*01:03 ~ DQB1*06:03 (OR 0.30, p < 0.00001) and DRB1*01:02 ~ DQA1*01:01 g ~ DQB1*05:01(OR 0.45, p < 0.00001). The HLA-DR3/DR4 genotype presented the greatest risk (OR 12.1, p < 0.0001) in 23.6% of the patients, followed by –DR3/DR3 (OR 10.6, p < 0.0001) in 9.8% and –DR3/DR9 (9.01, p < 0.0001) in 2.7%.

**Conclusion:** Regarding the most prevalent risk alleles, such as DR3, DR4, our findings are in accordance with previous studies both in European and in admixed population. It is important to notice that DR7 allele which is usually protective only in European population showed to be protective also in our population. This fact could be explained by the higher proportions of European ancestry, compared to African ancestry proportions in type 1 diabetes Brazilian patients demonstrated in previous studies from our group.

### O13 Impact of the use of the flash glucose monitoring system as diagnostic tool for patients with type 1 diabetes mellitus

#### Leticia Bernardes Cunha, Luiza Macedo Travalloni, Julia Bruno Vieira, Pedro Felisberto dos Santos Neto, Isabella Sued Leão, Joana Rodrigues Dantas, Lenita Zajdenverg, Melanie Rodacki

##### Departamento de Clínica Médica-Serviço de Nutrologia-Hospital Universitário Clementino Fraga Filho, Rio de Janeiro, Brazil

*Diabetology & Metabolic Syndrome* 2019, **11(Suppl 1):**O13

**Introduction:** Glycemic control reduces the risk of micro and macrovascular complications in patients with type 1 diabetes (T1D), but it is still a big challenge. Flash glucose monitoring system for intermittent continuous glucose monitoring (iCGM) may detect hyperglycemic and hypoglycemic episodes not perceived by capillary blood glucose monitoring, which could be helpful for these patients, especially those with labile glucose control.

**Objectives:** (1) To identify, through iCGM, hyperglycemic peaks and hypoglycemic episodes not identified by capillary blood glucose monitoring in patients with T1D, labile glucose control but adherent to treatment; (2) To identify if there is improvement in 14 days in time in glucose range (between 70 and 180 mg/dL; TIR), time in hypoglycemia and hyperglycemia, and estimated glycated hemoglobin after intervention in insulin therapy based on data obtained from iCGM.

**Methods:** In this prospective study, adults with T1D used Flash monitoring system for 14 days as a diagnostic tool. After this period, insulin was titrated and each patient underwent more 14 days of iCGM. TIR, time in hyperglycemia and hypoglycemia and estimated HbA1c were recorded before and after the alteration.

**Results:** The study included 40 individuals (19 men and 21 women), with a mean age of 25.6 ± 7.2 years old and a mean duration of diabetes of 15.3 ± 7.8 years. Their mean HbA1c was 7.8 ± 1.3. All patients were on basal-bolus insulin therapy (9 used NPH and 31 used long-acting analogs; 6 used Regular insulin and 34 used ultra-rapid insulin analogs). 9 had HbA1c < 7%. In the first 14-days iCGM evaluation, only 3 patients had TIR > 70% and 50% subjects had TIR < 50%. Patients with HbA1c < 7% did not have superior TIR than others (p = 0.058). There were no differences in TIR (52% vs 53%; p 0.027), percentage of hypoglycemia (22% vs 21%; p 0.018) and of hyperglycemia (25% vs 25%; p 0.33) between the first and second evaluation.

**Conclusions:** The use of iCGM for 14 days as a diagnostic tool detected significant hyper and hypoglycemic episodes in individuals with T1D and labile glycemic control despite good adhesion to treatment. Only one medical intervention after 14-days iCGM was not sufficient to improve patients’ glycemic control over the next 14 days in these individuals. It is possible that a longer follow-up and further titration would enable significant changes.

### O14 Posttransplant diabetes mellitus impact on kidney transplantation related outcomes

#### Luisa Penso Farenza^1^, Thizá Massaia Londero Gai^2^, Roberto Ceratti Manfro^1^, Cristiane Bauermann Leitão^1^, Andrea Carla Bauer^1^

##### ^1^Universidade Federal do Rio Grande do Sul, Porto Alegre, Brazil; ^2^Universidade Federal de Santa Maria, Santa Maria, Brazil

*Diabetology & Metabolic Syndrome* 2019, **11(Suppl 1):**O14

**Introduction and objective:** Posttransplant diabetes mellitus (PTDM) is a frequent complication after solid organs transplantation, occurring in 10–50% of kidney transplanted patients. This study’s objective is to determine the impact of different glycemic status at transplantation on death and graft loss in kidney recipients.

**Methods:** 1167 patients received a kidney graft between January 2000 and December 2013 at a southern Brazilian reference center and were included in this cohort study. Recipients were classified in three categories regarding glycemic status: PTDM, non-PTDM and pre-transplantation DM. PTDM diagnosis was adjudicated according to the 2014 International Consensus. Evaluated variables included sex, ethnicity, type of donor and recipients’ age at transplantation. Effect of these variables on death after transplantation and kidney graft loss was assessed by regression analysis. Survival analysis was also performed. Data were collected by electronic medical record review and the study was approved by the hospital’s ethical committee.

**Results:** From 1167 patients, 160 (15.3%) developed PTDM and 142 (13.6%) were diagnosed with pre-transplantation DM. On multivariate regression analysis, death incidence was higher in patients with PTDM (OR 3.23, CI 1.35–7.61, p = 0.008) and in patients with pre-transplantation DM (OR 4.27, CI 1.73–10.48, p = 0.001), compared to NPTDM. Death risk was also higher in older patients and those who received graft from deceased donor. Only pre-transplantation DM was associated to higher incidence of kidney graft loss (OR 3.48, CI 1.36–9.04, p = 0.009). Patients with PTDM did not present higher risk of kidney graft loss (OR 1.47, CI 0.69–2.89, p = 0.28). On Kaplan–Meier analysis, pre-transplantation DM recipients died earlier (991 (357–1626) days) than PTDM (p = 0.006) and non-PTDM (p = 0.02) patients. Time until graft loss also was earlier only in pre-transplantation DM group (p = 0.04).

**Conclusions:** After 167 months of follow-up (mean time since transplantation, 123 ± 47 months) in this kidney recipients’ cohort, PTDM does not impact in precocious mortality nor in early graft loss. PTDM seems to be a death risk factor, but it seems to be not due to kidney graft loss or events immediately related to the transplantation, as occurs in pre-transplantation DM group. PTDM could be associated to later death after transplantation related to chronic-metabolic and cardiovascular disease.

### O15 Preliminary performance of a new patch biosensor (LIVSEN^®^) for glucose monitoring

#### Monike Lourenço Dias Rodrigues, João Otávio Sedovski Garcia, David Evangelista da Silveira Junior, Aécio Marques Teixeira, CleytonLouredo Xavier, Raul Hilário Amaral, Larisse Silva Dalla Libera, Nelson Rassi

##### Hauttis Health Technology, Goiânia, Brazil

*Diabetology & Metabolic Syndrome* 2019, **11(Suppl 1):**O15

**Introduction:** A new patch sensor for sweat glucose detection was recently developed (Livsen^®^). After glucose-oxidase enzymatic reaction, continuous ruptures in a specific metal substrate are produced, and amperometric interference in radio signals by timely ruptures in the metal substrate are reflected by sensor´s built-in antenna to cell phone. Each glucose reading is based on radio signal interpretation by Near-Field-communication (NFC) technology and the corresponding app for iPhone.

**Objective:** The present study aimed to investigate the clinical performance of this sensor.

**Methods:** Nineteen patients (11 women) with diabetes (13 Type 1 DM, 6 Type 2 DM), aged 19–71 years (mean 42 years), were tested. Patients had the sensor attached to left forearm, during a 4-h test, during three consecutive days. Each patient returned to the test room in the same period (morning or afternoon). Sensor warm—up was 60 min, after an initial calibration with capillary blood glucose. After sensor warm-up in the first day, patients were guided to a room with stable temperature (23^o^ C), and stood seated during most part of the test. Two controlled meals were allowed during the 4-h test, and blood glucose calibration was not again performed. Fasting measurements, than postprandial measurements were obtained in 15 min intervals during 120 min, than hourly (180 and 240 min). Each measurement was a simultaneous assessment of capillary blood glucose in 2 glucometers (Accu-Chek Guide^®^, Roche and Freestyle Lite ^®^, Abbott) and a sensor reading by Livsen App for Iphone with NFC technology.

**Results:** The mean value of glucometers readings was compared to Livsen readings; 600 valid pairs of measurements were obtained (183, 210 and 207 in first (D1), second (D2) and third days (D3), respectively). The mean average relative difference (MARD) between mean glucometer values and Livsen readings was 11.1%. Mard of D1 was 8.7%, D2 was 11.86% and D3 was 12.36%, with ANOVA testing showing statistical difference between D1 MARD and D3 MARD. (Tukey multiple comparisons, *p* = 0.00078, IC = 95%). Clarke error grid analysis showed 98.6% of measurements in Clarke Zones A and B in D1, 98.8% in D2 and 98.2% in D3.

**Conclusion:** The new patch Livsen sensor showed adequate MARD and Clarke error grid Zone performance overall. The study will be extended to a greater sample, and the MARD difference among days 1 to 3 can be minimized with repeated calibrations.

### O16 Suboptimal glycaemic control in adults with type 1 diabetes in Latin America: characteristics, glycaemic control, hypoglycaemia and disease management. Results from sage analyses

#### Andre Gustavo Daher Vianna^1^, Balduíno Tschiedel^2^, Jose Fernando Botero^3^, Maurício Aguiar de Paula^4^, Juan Jose Gagliardino^5^

##### ^1^Centro de Diabetes de Curitiba, Curitiba, Brazil; ^2^Instituto da Criança com Diabetes - Grupo Hospitalar Conceição, Porto Alegre, Brazil; ^3^Universidad Pontificia Bolivariana, Medellín, Colombia; ^4^Sanofi, São Paulo, Brazil, ^5^CENEXA. Centro de Endocrinología Experimental y Aplicada, La Plata, Argentina

*Diabetology & Metabolic Syndrome* 2019, **11(Suppl 1):**O15

**Introduction:** Although achievement of glycaemic target effectively prevents the development/progression of diabetes complications, many people with type 1 diabetes (T1D) do not achieve their goal. SAGE study evaluated the glycaemic control in adults with T1D in LatAmcountries.

**Methods:** SAGE was a multinational, observational, cross-sectional study of patients aged ≥ 26 years with T1D for ≥ 1 year. Primary endpoint: percentage of participants in predefined age groups (26–44; 45–64; ≥ 65 years old) achieving HbA1c < 7%. Secondary endpoints: other glycaemic outcomes, hypoglycaemia, therapeutic management and diabetes technology use.

**Results:** SAGE included 488 eligible patients from Argentina, Brazil, Chile and Colombia; 61.5% female, mean (SD) age 45.6 (13.8) years, BMI 25.5 (4.2) kg/m^2^ and ≥ 10 years T1D duration was found in 83%. Hypertension or dyslipidaemia were present in 24.1% and hypothyroidism in 15.1%. Mean (SD) HbA1c was 8.15 (1.64)  %. Only 23.8% achieved HbA1c < 7.0%; this proportion was higher (27.9%) in the older subgroup. Mean (SD) FPG and PPG was 152.0 (77.59) and 168.3 (70.27) mg/dL, respectively. Mean (SD) total insulin dose was 49.5 (25.5) U/day (0.71 (0.34) U/kg/day) and 68.9% were on a basal-bolus regimen. Only 34.9% titrated basal insulin at least once a week. Insulin devices used were injections/pens (84.6%) or insulin pump (15.2%). 14.0% had at least one severe hypoglycemia within 6 months and 82.8% had one symptomatic hypoglycemia within the last 3 months. Incidence of diabetic ketoacidosis was 4.5% mainly related to infection (27.3%) and missing insulin dose (22.7%). 96.9% of patients were in use of finger-stick blood glucose meter and 77.6% used it at least once daily in the last 7 days; 21.1% were using continuous glucose meter and only 4.1% blood ketone meter.

**Conclusion:** Despite guidelines recommendations, glycaemic control is suboptimal in adults with T1D in LatAm, irrespective of age groups. The low and infrequent insulin titration may partly explain the lack of glycaemic control. Further action is required globally including training of physicians and patients for appropriate insulin titration and carbohydrate counting. Use of new technologies and advanced insulin formulations may benefit HbA1c target achievement in this population.

Study sponsored by Sanofi.

### O17 Teleophthalmology screening for diabetic retinopathy in São Paulo metropolitan area, Brazil: economic assessment

#### Fernando Korn Malerbi^1^, Rubens Belfort Junior^1^, Olimpio J. Nogueira V. Bittar^2^, Paulo Henrique Morales^1^

##### ^1^Department of Ophthalmology, Federal University of São Paulo, São Paulo, Brazil; ^2^Health State Secretary São Paulo, São Paulo, Brazil

*Diabetology & Metabolic Syndrome* 2019, **11(Suppl 1):**O17

**Introduction and objectives:** Diagnosis and timely treatment of diabetic retinopathy are the mainstay for diabetic blindness prevention; however, proper screening is limited by several barriers. Ampliation of access to retina examination is a global challenge. Brazil has the fourth largest diabetic population in the world, with increasing demand for diabetic retinopathy screening. The objectives of this study are: (1) to evaluate the rate of detected cases in a telemedicine program for diabetic retinopathy screening, and (2) to compare costs for such screening with those of a conventional program.

**Methods:** Retrospective study which assessed the outcomes of a diabetic retinopathy screening program undertaken at peripheral areas of Sao Paulo city and its Metropolitan Area, with a protocol that combined fundus photographs and telemedicine. Fixed costs for telemedicine screening were calculated, as well as the costs for conventional screening. Direct and indirect costs were taken into account; the former comprised diagnostic expenditures and the latter considered travel costs and lost working days. Different scenarios, depending on the resolutivity and the presence of a local ophthalmologist, were evaluated.

**Results:** A total of 28,842 patients were evaluated; treatment was indicated in 18.99%: cataract surgery (10.84%) or diabetic retinopathy treatment—laser photocoagulation (6.93%) or vitrectomy (1.21%). The remaining 81.01% were non-referrable patients who could be followed at the primary care setting; such protocol prevented unnecessary travel and consultation expenses, as well as less lost working days. The cost per case detected would be 2.65 to 5.35 higher if conventional screening protocols were used, the variables being the presence of a local ophthalmologist and the resolutivity of the service.

**Conclusion:** The present data indicate that diabetic retinopathy screening with telemedicine is feasible in Brazil, and such strategy addresses important barriers such as cost and availability of the specialist. Telemedicine screening has a good cost-effectivity profile for the public health system as it delivers a lower cost per case detected, mainly in areas lacking specialists. The present results encourage the implementation of such protocols in underserved areas, provided that treatment can be offered to the detected cases.

### O18 The effect of transcranial direct current stimulation associated with hypocaloric diet on food intake and weight loss in overweight or obesity: a double-blind, randomized clinical trial

#### Carina de Araujo^1^, Raquel Crespo Fitz^1^, Daniela Albugeri Nogara^1^, Amanda Farias Osório^3^, Gabriella Richter da Natividade^1^, Paula Nunes Merello^3^, Pedro Schestatsky^2^, Fernando Gerchman^1^

##### ^1^Endocrine and Metabolic Unit, Hospital de Clínicas de Porto Alegre, Porto Alegre, Brazil; ^2^Neurology Service, Hospital de Clínicas de Porto Alegre, Porto Alegre, Brazil; ^3^Universidade Federal do Rio Grande do Sul, Porto Alegre, Brazil

*Diabetology & Metabolic Syndrome* 2019, **11(Suppl 1):**O18

**Introduction:** The dorsolateral prefrontal cortex (DLPFC) plays an important role in appetite and food intake regulation and may be a target for transcranial direct current stimulation (tDCS), a treatment modality that has shown to reduce food craving and calorie intake. Thus, we tested the effect of active tDCS associated with a hypocaloric diet (HD) on weight loss and food consumption in overweight or obese adults.

**Methods:** In this randomized, placebo-controlled, double-blind study, overweight or obese adults were selected to completed a 4-wk (20 sessions; 5 weekdays) of fixed-dose tDCS (2 mA, 20 min) delivered over the right DLPFC associated whit a HD. Subjects were randomly assigned (1:1) and stratified by sex to active tDCS + HD (AG), or sham tDCS + HD (SG). The primary outcomes were weight loss and the secondary outcome were changes in food intake and in desire to eat. Body weight was assessed weekly at baseline (t_0_), and at days 5 (t_5_), 10 (t_10_), 15 (t_15_), 20 (t_20_), and at the end of the study (t_F_). Habitual food intake was assessed at t_0_, t_10_ and t_20_ using a 3-day weighed dietary records. A 100-mm visual analog scale was used to assess desire to eat something sweet, salty, savory, or fatty at t_0_ and t_F_. All analyses were performed in intention-to-treat (ITT) using generalized estimating equations. All participants provided written informed consent. ClinicalTrials.gov (NCT02683902).

**Results:** 28 individuals were randomized and included in the ITT (mean age, 37.6 ± 5.8 years; BMI, 31.5 ± 2.4 kg/m^2^); 23 received all 20 planned sessions. Although there was a greater weight loss in the AG than in the SG at t_F_ (− 4.5 kg [95% CI: − 9.4, 0.5] vs. − 2.3 kg [95% CI: − 5.0, 0.3]), this difference was not statistically significant. The energy intake was not statistically different between groups at t_0_, t_10_ and t_20_. There was a significant interaction in the desire to eat sweet foods (p = 0.005). The AG showed a significant 23.7 percentage points reduction (95% CI: − 40.2, − 7.1) in the desire for sweets over the study, whereas the SG had a non-significant 1.0 percentage point increased (95% CI: − 13.3, 15.2).

**Conclusions:** Although we might not show that repetitive active tDCS is able to optimize weight loss and decrease calorie intake, it was able to reduce the desire to eat sweet foods. These findings open a new perspective for this therapeutic modality as a potential strategy for the treatment of food craving and metabolic disorders related to carbohydrates consumption. FIPE 15-0119.

### O19 Translation, cultural adaptation and validation of IDSRQ to Brazilian Portuguese

#### Raquel Cristina Lopes Assis Coelho^1^, Adriana Silvino Pagano^2^, Aleida Nazareth Soares^1^, Janice Sepulveda Reis^1^

##### ^1^Instituto de Ensino e Pesquisa da Santa Casa de Belo Horizonte, Belo Horizonte, Brazil; ^2^faculdade de Letras, Universidade Federal de Minas Gerais, Belo Horizonte, Brazil

*Diabetology & Metabolic Syndrome* 2019, **11(Suppl 1):**O19

**Introduction:** The Insulin Delivery System Rating Questionnaire (IDSRQ) is a measure of health-related quality of life (HRQOL) and treatment preference for insulin delivery systems in persons with type 1 (T1DM) and type 2 diabetes mellitus (T2DM). The aim of the study was to translate and cross-culturally adapt the IDSRQ for Brazilian users as well as evaluate the validation of selected psychometric aspects.

**Materials and methods:** methodological study carried out in the following stages: forward translation, synthesis, back-translation, assessment by Judge Committee, pre-test and validation. International guidelines for translation and cross-cultural adaptation of measurement tools were followed. The validation provided information about the reliability (internal consistency, test–retest) and the construct validity of the studied tool.

**Results:** Regarding content validation, the instrument performed well in the Judges’ assessment with a mean Content Validity Index of 0.87 (± 0.2). Pre-test step involved 30 T1DM in face to face discussions. The IDSRQ validation study involved 113 T1DM patients, 46% male, mean age 32.61 (± 12.59) years and mean age at diagnosis of diabetes of 17.51 (± 12.41). 27.4% were using vial and syringe; 61.1% using pen and 11.5% using insulin pump. 76.5% of the patients administer insulin > 5 times daily. NPH was the most used basal insulin (39.8%), followed by glargine U100 (34.5%). Lispro was the most used fast insulin, by 60.2% of the patients. The scale presented acceptable internal consistency (Cronbach’s alpha = 0.785).

**Conclusions:** The translated and cross-culturally adapted Brazilian Portuguese version may be used to assess HRQoL and treatment preferences for insulin delivery systems in T1DM Brazilian patients.

**Keywords:** Diabetes mellitus; Type 1; Quality of life; Insulin

### O20 Triglycerides levels could be a marker of large for gestational age in gestational diabetes?

#### Micaela Frasson Montero, Filipe Dias de Souza, Rosiane Mattar, Sergio Abbud Dib, Patrícia Medici Dualib, Bianca de Almeida Pititto

##### Escola Paulista de Medicina - Universidade Federal de São Paulo, São Paulo, Brazil

*Diabetology & Metabolic Syndrome* 2019, **11(Suppl 1):**O20

**Introduction:** One of the complications of gestational diabetes (GDM) is the higher frequencies of elevated birth weight, which has also been associated with mother’s previous body mass index and weight gain during pregnancy. Recent studies have shown a positive association between triglyceride (TG) levels at late pregnancy and birth weight of newborns in pregnant women without gestational diabetes (GDM), but there is lack of knowledge regarding the relation of TG and birth weight in GDM.

**Objective:** The aim of the study was to evaluate the association of birth weight with levels of triglycerides (TG) in the third trimester of gestational diabetes (GDM).

**Methods:** The study included 645 pregnant women with GDM in follow-up at DM and Gestation service, from 2008 to April/2019 and data were collected from the participants’ medical records. Participants were stratified by median of TG (median = 173.0 mg/dL). Metabolic characteristics and birth weight were presented as mean (standard deviation) and compared by Student’s t-test; and the frequency of large for gestational age (LGA, weight > 90th percentile at birth) was presented as percentage(n) and compared using Chi Square test, p < 0.05.

**Results:** The sample had mean age of 34 (5) years, body mass index of 29.9 (9.5) kg/m^2^, HbA1c of 5.6 (0.6)% and weight gain during pregnancy of 9.5 (5.9) kg, that did not differ between groups. Both groups had also similar frequency in using insulin during pregnancy, 38% in the group above and 41% in the group below the median. Women with TG above de median had greater levels of total cholesterol [233 (45) vs. 202 (39) mg/dL, p < 0.001], LDL-cholesterol [126 (42) vs. 109 (34) mg/dL, p < 0.001] and lower levels of HDL-cholesterol [61 (14) vs. 67 (15) mg/dL, p < 0.001] comparing to the group with TG below the median; while birth weight of the newborn did not differ [3.2 (0.5) vs. 3.1 (0.5) kg, p = 0.29] between groups. Pearson correlation was also not significant between birth weight and TG. There was a borderline significance for the comparison of frequency of LGA [10% (32) vs. 6% (19), p = 0.056] considering the groups above and below the median of TG respectively.

**Conclusions:** In this homogeneous group of GDM women regarding age, BMI, weight gain and use of insulin during pregnancy, higher TG was associated with greater frequency of LGA. We hypothesize that TG might be representing a higher insulin resistance and could be a biomarker to consider preventive strategies against LGA in GDM.

## Poster presentations

### P1 10 years follow up of first degree relatives of type 1 diabetes patients - presence of autoimmune biomarkers and the progression to diabetes

#### Isabella Sued Leão^1^, Débora Batista Araujo^2^, Bianca Barone^3^, Matheus dos Santos Mantuano^2^, Joana Rodrigues Dantas Vezzani^1^, Lenita Zajdenverg^2^, Melanie Rodacki^2^

##### ^1^Hospital Universitário Clementino Fraga Filho - HUCFF, Rio de Janeiro, Brazil; ^2^Faculdade de Medicina da Universidade Federal do Rio de Janeiro - UFRJ, Rio de Janeiro, Brazil; ^3^Instituto Estadual de Diabetes e Endocrinologia Luiz Capriglione – IEDE, Rio de Janeiro, Brazil

*Diabetology & Metabolic Syndrome* 2019, **11(Suppl 1):**P1

**Introduction:** In caucasian first degree-relatives (FDR) of patients with type 1 diabetes (T1D), the presence of diabetes-related multiple autoantibodies is strongly associated with progression to the disease, but most individuals with only one antibody do not develop T1D. However, a long-term prospective evaluation of these individuals in the Brazilian population is still lacking.

**Objective:** To assess the autoimmunity in FDR of patients with T1D and the progression to T1D after 10 years of follow-up.

**Methods:** In this prospective study, non-diabetic FDR of T1D patients were interviewed and blood was drawn for autoantibodies measurement (GADA, IA-2A, IAA, ZnT8A). Serum samples were analyzed by standard radioligand binding assays (GADA, IAA, IA2A and ZnT8A). The FDR were interviewed by phone after 10 years to determine if they had developed T1D. The study was approved by the institutional Ethical Committee. Mann–Whitney U test and Chi square were used for comparing groups. Spearman coefficient and Fisher’s exact t-test were used to test correlation between variables.

**Results:** 81 individuals were analyzed (50 siblings and 31 offspring), with a median age of 20 years old. 16 subjects lost follow up.13 subjects presented positivity for autoantibodies associated with T1D.10 were positive for 1 autoantibody and 3 subjects were positive for multiple autoantibodies (1 of them showed positivity for 2 autoantibodies—GADA and ZnT8A—and the other two were positive for 3 autoantibodies—GADA, IA2A, ZnT8A). The 3 subjects with multiple positive autoantibodies developed T1DM within 10 years. None of the other individuals developed the disease.

**Conclusions:** After a 10-year follow-up, in Brazilian FDR of T1D patients, the positivity for two or more autoantibodies indicated a greater chance of progression to T1D, similar to that observed in Caucasians. ZnT8A enhanced T1D development prediction when combined with other autoantibodies, since it detected multiple antibody-positivity in one individual that later develop T1D. This suggests that ZnT8A might be useful for autoimmunity in FDR of patients with T1D in our population, but larger studies are still required to address this question.

### P2 A classification for non classified types of diabetes in a tertiary diabetes center

#### Fernanda Izabel Heckert, Alessandra Saldanha Matheus Fernandes Da Costa, Lucianne Righeti Monteiro Tannus, Livia Furtado Leba, Pâmela Suelen De Moraes, Ronaldo Gama Pena, Roberta Arnoldi Cobas, Marília Brito Gomes

##### Universidade do Estado do Rio de Janeiro, Rio de Janeiro, Brazil

*Diabetology & Metabolic Syndrome* 2019, **11(Suppl 1):**P2

**Introduction:** Classifying diabetes (DM) type is a challenge for a subset of patients especially if it is based on clinical data alone. This uncertainty can lead to difficulties in guiding the best therapy for the patient.

**Aims:** To apply pancreatic autoimmunity and beta cell function assessment for previously doubtful DM classification.

**Methods:** We retrospectively evaluated pancreatic autoimmunity (antiGAD, anti-insulin [IAA], anti-islet, antiIA2, antiZnT8) and fasting C-peptide levels of patients with unclassified Diabetes from 2016 to June/2019. Patients were then classified according to presence (A+) or absence (A−) of pancreatic autoantibodies and for preserved (β+) and non-preserved (β−) β cell function defined as fasting C-peptide level above or below 0.6 ng/mL. Type 1 DM was defined when classical acute Diabetes symptoms or Diabetic Ketoacidosis (DKA) were present at onset, associated with the continuous need of insulin since the diagnosis. LADA was defined as latent symptoms associated with no insulin use at the onset of the diagnosis ot Type 1DM in adults. Type 2 DM was considered when mild symptoms and a family history of Diabetes were present. MODY was considered when a relevant Diabetes family history was present associated with mild clinical symptoms. Results are presented as mean ± SD, median [interquartile range] or n(%). Statistical analysis was performed using SPSS-IBM 22.0 package.

**Results:** We included 47 patients, aged 27.4 ± 15.1 years (2–66) with age at DM diagnosis of 20.7 [11–31] years, duration of DM of 49 [12–84] months. Of the 26 patients classified as A−β+, 11 (42.3%) had Type 2 DM, 2 (7.7%) had recent onset Type 1 DM, 1 (3.8%) ketosis-prone DM, 2 (7.7%) “probably” MODY, 1 (3.8%) corticosteroids-induced DM and 9 (34.7%) persisted clinically non classified. Of the four A−β− patients, 2 (50%) had type 1B DM, 1 (25%) fulminant type 1 DM and 1 (25%) type 2 DM. Of the 5 A+β+ patients, 3 (60%) had type 1 DM, 1 (20%) had type 2 DM and 1 (20%) persisted non-classified. Of the 12 A+β− patients, 9 (75%) had type 1 DM and 3 (25%) LADA.

**Conclusion:** In addition to clinical features, evaluation of beta cell autoimmunity and function helped to classify the type of diabetes in the vast majority of our previously unclassified patients allowing us to withdraw insulin therapy, safely, in some cases when not necessary anymore. However, a small part of them may need longer clinical follow-up for their proper classification.

### P3 A screening of type 1 diabetes mellitus (T1DM)’ complications in a private clinic and a public outpatient clinic in Joinville - SC

#### Rejane Baggenstoss, Lucas Irineu Medeiros de Oliveira, Eloise Mariani Salamaia, Guilherme Thomaz, Goretti Silveira Rodrigues, Suely Keiko Kohara

##### UNIVILLE - Universidade da Região de Joinville, Joinville, Brazil

*Diabetology & Metabolic Syndrome* 2019, **11(Suppl 1):**P3

**Introduction:** T1DM is linked to micro and macrovascular long-term complications that result in a lower quality of life and life expectancy. The absence of data on prevalence of complications in T1DM patients in our city led us to conduct this study.

**Objective:** To determine the clinic and metabolic control and prevalence of complications in T1DM patients from a private clinic and a public outpatient clinic.

**Methods:** This was a retrospective, observational study with patients from a private (PV) and a public (PB) outpatient clinic. The inclusion criteria were: patients with T1DM for more than 10 years and above 18 years of age. The following variables were obtained from medical records: age, gender, height, weight, duration of diabetes, blood pressure, comorbidities, smoking status, type of insulin therapy, levels of HbA1c, total cholesterol, HDL-c, LDL-c, triglycerides, presence of microvascular complications.

**Results:** We evaluated 64 T1DM patients: 25 from PB and 39 from PV clinic. Patients from PB had mean age of 34.0 years (± 14.0), 7 (28%) had hypertension (sBP ≥ 140 mmHg and/or dBP ≥ 90 mmHg), 3 from 23 patients (12.5%) referred smoking. Patients from PV had mean age of 33.0 years (± 11.0), 7 (18%) had hypertension, 34 were non-smoking. Mean number of clinical visits in the last year was 1.92 (± 1.22) in PB and 3 (± 1.32) in PV *(p *= *0.0017)*. Thirteen patients from PB (52%) (10F/3M) and 14 (35.9%) from PV, had BMI > 25 kg/m^2^ (7F/7 M).

Mean HbA1c levels in PB group was 9.11% (± 2.03) and 8.45% (± 1.78) in PV group *(p *= *0.17* *ns)*. Mean triglycerides was 118 mg/dL (± 89) in PB group and 77.2 mg/dL (± 38.99) in PV group *(p *= *0.014).* There was no statistical difference in total cholesterol and HDL-c between the groups. Only 2 patients (8%) from PB and 10 (25%) from PV had HbA1c < 7.0%.

In the PB group, we found information about retinopathy in 4 from 23 patients (17.4%), about nephropathy in 5 from 24 patients (20.8%) and neuropathy in 2 from 20 patients (10%). In the PV group, we found information about retinopathy in 4 from 25 patients (16%), about nephropathy in 7 from 30 patients (23.3%) and neuropathy in 6 from 30 pacientes (20%).

**Conclusions:** Although the limited number of patients, our results are similar to previous national studies and emphasize the difficulties associated with the treatment of T1DM patients in routine clinical care and the need for improved treatment quality.

### P4 AAV9-mediated enhanced fatty-acid oxidation reverts obesity-induced hepatic steatosis

#### Mineia Weber Blattes^1^, Paula Mera^2^, Fina Casas^3^, David Sebastián^4^, Raquel Fucho^5^, Vicenta Llorente^6^, Laura Herrero^2^, Dolors Serra^2^

##### ^1^Universidade Franciscana, UFN, Santa Maria, Brazil; ^2^Department of Biochemistry and Physiology, Universitat de Barcelona, UB, Barcelona, Spain; ^3^Centro de Investigación Biomédica enRed de Enfermedades Hepáticas y Digestivas (CIBEREHD), Barcelona, Spain; ^4^Institute for Research in Biomedicine (IRB Barcelona), Universitat de Barcelona, UB, Barcelona, Spain; ^5^Centro de Investigación Biomédica enRed de Enfermedades Hepáticas y Digestivas, Barcelona, Spain; ^6^Institute of Biomedical Research of Barcelona (IIBB), Barcelona, Spain

*Diabetology & Metabolic Syndrome* 2019, **11(Suppl 1):**P4

**Introduction**: Despite the enormous efforts of health care providers and the research community, rates of obesity continue to rise. Unfortunately, safe and efficient treatments are currently unavailable. Of great concern is the parallel increase in the prevalence of obesity-associated pathological conditions such as insulin resistance, type 2 diabetes, nonalcoholic fatty liver disease (NAFLD), cardiovascular disease, and cancer. Hence it is vital to develop novel therapeutic strategies for combating this epidemic.

**Objective:** In this study, we evaluated the therapeutic potential of safe nonimmunoreactive adeno-associated virus (AAV) to mediate the expression of a permanently active mutant form of the human carnitine palmitoyltransferase 1A (hCPT1AM), the key enzyme in fatty acid β-oxidation (FAO), in the liver of a mouse model of high-fat diet-induced obesity and NAFLD. Moreover, we searched for new potential lipid biomarkers for monitoring liver steatosis in humans.

**Methods:** We used two viruses to express in mouse liver a human mutated isoform of CPT1A(hCPT1AM) or green fluorescent protein (GFP), which was used as a control (AAV9-hCPT1AM and AAV9-GFP, respectively).

**Results:** Our results showed that obese mice expressing hCPT1AM enhanced hepatic FAO which resulted in increased production of CO2, ATP, ketone bodies and heat. The increase in FAO activated autophagy, lipolysis and cholesterol mobilization in liver. As a result, the blood glucose levels and liver steatosis was reduced in hCPT1AM-expressing mice. In addition, the increase in hepatic FAO altered the mice liver and serum lipidomic profile, which drew attention to the specific C20:0 ceramide C38:5 diacylglyceride and C50:1 triacylglyceride (TAG) species which could act as potential biomarkers for NAFLD reversion.

**Conclusions:** In summary, AAV-mediated expression of hCPT1AM in the liver of obese mice is sufficient to improve NAFLD. Moreover, our study revealed a potential circulating biomarker that might be useful to monitor hepatic steatosis and NAFLD improvement.

### P5 Action of antioxidant therapy on oxidative stress and systemic inflammation of diabetic rats

#### Hilary Barros de Carvalho, Natan Reyges Castro da Purificação, Melyna Soares de Souto, Mário Fernando da Costa Pinto Neto, Mariana Braga Sampaio, Luan Carvalho de Assunção Rocha, João Vitor Ferreira Assunção de Araújo, Naianne Kelly Clebis

##### Universidade Federal do Rio Grande do Norte - Laboratório de Microscopia Celular e Tecidual/DMOR, Natal, Brazil

*Diabetology & Metabolic Syndrome* 2019, **11(Suppl 1):**P5

**Introduction:** Diabetes Mellitus (DM) is a disease that leads to hyperglycemia and increased oxidative stress and systemic inflammation, which can cause morphofunctional changes in different organs, with severe damage to the body.

Objective: This study aimed to analyze the effects of antioxidant treatment on the oxidative stress and systemic inflammation indicators in rats with diabetes mellitus.

**Methodology:** Twenty male rats were divided into four groups (n = 5): N (normoglycemic); D (diabetic); NT (normoglycemic treated with antioxidants) and; DT (diabetic treated with antioxidants). DM was induced by streptozotocin (35 mg/kg body weight). Antioxidant treatment was performed with: quercetin (100 mg/kg body weight), l-glutamine 1% and α-tocopherol 1% for 60 days. Blood glucose, MDA, GSH, IL-1β and IL-10 levels were analyzed. Fisher’s test was used (p < 0.05).

**Results:** Hyperglycemia was observed in D and DT, being higher in DT than in D. MDA and GSH levels were similar in D and N, and higher in DT than NT. There was no difference in IL-1β levels between D and N, and between DT and D. However, the concentration of 1L-10 was lower in D than in N and higher than DT and D. In NT there was a reduction of 1L. -10 compared to N.

**Conclusions:** The findings indicate that the combined treatment with antioxidants did not change the plasmatic glucose levels in DT. In addition, the experimental model used in this research did not generate oxidative stress in the untreated groups, while in the treated groups there was an increase in MDA (pro-oxidant) and GSH (antioxidant) levels suggesting a tentative of the organism in balancing the oxidative-antioxidant system. No induction of systemic inflammation (IL-1β levels) was identified, but antioxidant treatment promoted to increased activity of the anti-inflammatory pathway (IL-10 levels) in DT animals, indicating that antioxidants may be adjuvant in diabetes mellitus treatment, but further studies are needed to better elucidate these findings.

**Keywords:** Hyperglycemia; Oxidative stress; Inflammation.

### P6 Acute effect of isometric handgrip exercise on the glycemic response of physically active type 2 diabetics: randomized clinical trial

#### Theo Victor Alves Soares do Rêgo, Pedro Cauê Nunes De Oliveira e Silva, Gabriel Lima De Souza, Ricardo Azevedo Cavalcanti Aragão, Rodrigo dos Santos Rodrigues Alves, Jonathan Nícolas Dos Santos Ribeiro, Cláudio Barnabé Dos Santos Cavalcanti, Denise Maria Martins Vancea

##### Universidade de Pernambuco, Recife, Brazil

*Diabetology & Metabolic Syndrome* 2019, **11(Suppl 1):**P6

**Introduction:** The possibility of obtaining specific benefits with exercises for small muscle groups, such as those of handgrip, is already known in the current literature, however, the relationship of this type of exercise on the glycemic response of type 2 diabetics is not clear.

**Objective:** To evaluate the effect of a single session of an isometric handgrip protocol on the glycemic response of physically active type 2 diabetics.

**Methods:** It was a randomized controlled clinical trial (Ethics Commitment: 36498714.7.0000.5207). The research was conducted in the morning, in a laboratory of a Northeastern Public University. The sample was non-probabilistic, consisting of 16 physically active type 2 diabetics aged 68 ± 12 years. Diabetic patients were randomly assigned to two groups, the control group (CG—n = 8) and the intervention group (GI—n = 8). With a dynamometer, the maximum voluntary contraction force (MVCF) was measured in both hands. For the session, 50% of the load found in the MVCF test was used, applying the unilateral protocol of four sets of five repetitions, kept 10” in isometric contraction and 20” in recovery, with 60” rest between series and alternation of the hands. Capillary blood glucose was collected pre-intervention, immediately after, 30 and 60 min after the intervention with isometric handgrip protocol in both groups. The ANOVA parametrictest for repeated measures was performed. The program used was SPSS 20.0. A significance level of p ≤ 0.05 was considered.

**Results:** According to the data analysis, GI capillary blood glucose showed a significant decrease between pre-intervention and immediately after (pre 164.1 ± 36.7, post 137 ± 24.9, p = 0.01) and also between the moments immediately after and 60 min after the intervention (pre 137 ± 24.9, post 118.3 ± 20, p = 0.04).

**Conclusion:** The isometric handgrip protocol was able to promote a decrease in capillary blood glucose in physically active type 2 diabetics.

### P7 Acute effects of a moderate intensity exercise session on cognitive function and serum concentration of neurotrophic factors in type 1 diabetics

#### Nascimento MCC, Prado LS

##### Universidade Federal de Minas Gerais, Belo Horizonte, Brazil

*Diabetology & Metabolic Syndrome* 2019, **11(Suppl 1):**P7

**Introduction:** Previous studies indicate that the cognitive function (executive function) of people with type 1 diabetes can be affected by the sudden glycemic variation in the first years of life. However, studies also show improvement of cognitive function with regular exercise, regardless of modality. The explanation for this may be associated with the production of some neurotrophic factors, such as brain derived neurotrophic factor (BDNF) and insulin-like growth factor (IGF-1).

**Objective:** To compare cognitive performance responses and serum concentrations of neurotrophic factors in patients with type I diabetes after a single session of coordinative and aerobic exercise.

**Methods:** After approval by the Research Ethics Committee, the sample was selected by convenience and consisted of 23 individuals aged 8 to 18 years, diagnosed with Type 1 Diabetes and at least 3 years of disease duration. Two groups were defined: (I) Aerobic Exercise and (II) Coordinating Exercise, according to the activity they would perform in the first experimental session. The stages were performed in a weekend colony for type 1 diabetic patients for 2 days and consisted of blood collection, application of cognitive tests and exercise session previously elaborated. Blood collection and cognitive tests were applied before and immediately after each exercise session.

**Results:** It was observed that the coordinative exercise caused greater positive changes (reduction of time and number of errors, and increase of correct answers) in 6 variables of the applied psychological tests, while the aerobic exercise caused changes in only 2 of these variables. However, when adjusted by the baseline, this difference becomes significant for only two variables. Regarding blood dosages, before and after the coordinating exercise session, BDNF values (17.56 ± 3.40 and 17.73 ± 2.50, p = 0.959) and IGF-1 (1.32 ± 0.95 and 1.59 ± 1.17, p = 0.384), respectively. Before and after the aerobic exercise session, the results were BDNF (17.56 ± 1.79 and 17.89 ± 3.31, p = 0.959) and IGF-1 (1.58 ± 1, 17 and 1.92 ± 1.43, p = 0.384), respectively.

**Conclusions:** The results obtained suggest that improvements in executive function may be greater after exercises with more pronounced demands for motor coordination and sensory integration (coordinative exercise). Plasma concentrations of BDNF and IGF-1 did not change as a result of the physical exercises studied.

**Keywords:** Diabetes; Exercise; Cognition

### P8 Acute physical exercise promotes peripheral analgesia by activation of the opioidergic system in mice with diabetic peripheral neuropathy (DPN)

#### William Valadares Campos Pereira, Douglas Lamounier de Almeida, Igor Dimitri Gama Duarte, Sérgio Henrique Sousa Santos, Thiago Roberto Lima Romero

##### Departamento de Farmacologia, Instituto de ciências Biológicas, ICB-UFMG, Belo Horizonte, Brazil

*Diabetology & Metabolic Syndrome* 2019, **11(Suppl 1):**P8

**Introduction:** Diabetic neuropathy is the most prevalent complication of diabetes in the population and affects the life’s quality of patients. The painful symptoms are difficult to control, leading researchers to investigate alternative treatment strategies. Few studies has investigated the role of peripheral endogenous modulation of pain caused by exercise in this pain.

**Objective:** The aim of this study was to investigate peripheral activation of the opioid system in diabetes-induced hyperalgesia in a maximal progressive exercise session in Swiss mice.

**Methods:** This study was approved by the Animal Experimentation Ethics Committee (CEUA, Brazil). Diabetes was induced with a high fat diet consisting of 39% carbohydrate, 17% protein and 44% fat, totaling 4.27 kcal per gram of diet. The rats underwent 12 h of fasting to perform insulin resistance and glucose intolerance tests. Allodynia was measured according to the paw pressure test (Kawabata et al., 1992). To perform this protocol, a treadmill operated by an electric motor was used. After acclimation with the device, the mice were subjected to fatigue exercise, where the speed was increased at a rate of 1 m per minute every 3 min until fatigue (American Physiological Society, 2006), maintaining a constant inclination of fatigue. 5% Naloxone, a non-selective opioid receptor antagonist, was kept in the freezer in a stock solution dissolved in saline.

**Results:** Insulin resistance and glucose intolerance tests showed differences between groups. Insulin resistance (p = 0.0018) and glucose intolerance (p = 0.0065). Allodynia was observed in rats fed DHF were observed at the thirteenth week of treatment (p = 0.0322). There was a significant difference in nociceptive threshold in the groups submitted to the progressive maximal exercise protocol until the time fatigue (p = 0.0003) in relation to the groups kept at rest. There was a significant difference in nociceptive threshold following the progressive maximal exercise protocol for fatigue between the groups receiving naloxone (p < 0.0001)) and the groups receiving saline. The effects of naloxone were observed up to 60 min after injection, when the nociceptive threshold returned to baseline levels.

**Conclusions:** The progressive-maximal physical exercise protocol until fatigue, induces peripheral analgesia via the opioid system in the diabetic neuropathic pain model in Swiss mice fed 40% high fat diet.

### P9 Acute supplementation with flavanol-rich cocoa improve glycemic control after incremental treadmill exercise and decrease expression of TRB3 in liver and skeletal muscle on type 2 diabetic (T2DM) rats

#### Bruno Pereira Melo^1^, Pedro Henrique Madureira Ogando^1^, Aline Cruz Zacarias^1^, Joyce Camila Cruz de Oliveira^1^, João Gabriel da Silveira Rodrigues^1^, Elsa Heyman^2^, Romain Meussen^3^, Danusa Dias Soares^1^

##### ^1^Universidade Federal de Minas Gerais, Belo Horizonte, Brazil; ^2^Université de Lille, Lille, France; ^3^Vrije Universiteit Brussel, Brussels, Belgium

*Diabetology & Metabolic Syndrome* 2019, **11(Suppl 1):**P9

**Introduction:** Tribbles homolog 3 protein (TRB3) is a pseudo-kinase capable of binding to protein kinase B (Akt), inhibiting its phosphorylation, hindering glucose uptake in peripheral tissues. Physical exercise and flavanol consumption improvesglucometabolism, oxidative stress and vascular function, but the effects of this combination have been poorly investigated.

**Objective:** To verify the acute effects of flavanol-rich cocoa supplementation alone or associated to exercise on insulin, glucose and in TRB3 expression in liver and skeletal muscle of T2DM rats.

**Methods:** 64 Wistar rats (250 ± 10 g) had T2DM induced by consumption of high-fat diet (46%Fat; 43%CHO; 11%Pro) plus fructose diluted in water for 30 days. The rats were allocated into non diabetics (CON) and diabetic group (T2DM) and performed an incremental treadmill exercise test (EXE) or rested (SED), supplemented with flavanol (COCOA) or placebo (PLA, same substance without flavanol). The cocoa powder (NATUREX©, France) was diluted in water (1.8 ml kg^−1^) and administered one hour before the EXE via oral gavage (45 mg kg^−1^). The EXE consisted of running on a treadmill (Panlab^®^) at a speed of 10 m min^−1^, increased by 1 m min^−1^ every 3 min until fatigue. TRB3 expression was quantified by quantitative PCR real time and serum insulin concentrations by ELISA. Blood glucose levels were assessed before gavage, before and after EXE and during 60 min of recovery with a glucometer (Accu-chek Performa, Roche^®^).

**Results:** The physical performance parameters were not different between groups and situations (*p *> 0.05).T2DM rats supplemented with flavanol showed an attenuation on the elevation of blood glucose concentrations during recovery after exercise, with glucose values similar to CON supplemented with placebo (*p *= 0.001). In addition, both flavanol supplementation and exercise reduced insulin concentrations when compared to the placebo and non-exercise supplemented group (4.76 ± 0.57; 4.82 ± 0.34 vs. 7.15 ± 0.61 ng mL^−1^, respectively). When combined, exercise and flavanol reduced (*p *= 0.001) the expression of hepatic and skeletal muscle TRB3 in the T2DM group.

**Conclusion:** Acute supplementation of cocoa flavanol may help glycemic control after exercise in T2DM and in non-diabetic rats by diminishing hepatic glucose production and increasing muscle glucose uptake through reduction on liver and muscle TRB3 expression.

Financial support of Minas Gerais State Agency for Research and Development (FAPEMIG).

### P10 Adherence and persistence for dulaglutide (DU) vs. basal insulin (BI) in injection- naïve patients with type 2 diabetes (T2D): the dispel TM study

#### Anderson Leandro Couto Peres Ribeiro^1^, Reema Mody^2^, Qing Huang^3^, Maria Yu^4^, Xian Zhang^3^, Liya Wang^3^, Michael Grabner^3^, Hiren Patel^2^

##### ^1^Eli Lilly and Company, São Paulo, Brazil; ^2^Eli Lilly and Company, Indianapolis, USA; ^3^HealthCore, Inc., Wilmington, USA; ^4^Eli Lilly and Company, Toronto, Canada

*Diabetology & Metabolic Syndrome* 2019, **11(Suppl 1):**P10

**Introduction and Objectives:** Adherence and persistence are key considerations in patient-centric treatment selection for T2D management. The objective of this retrospective real-world study was to assess 1-year adherence and persistence using different measures among injection-naïve patients with T2D initiating DU vs. BI. **Methods:** A US claims database was used to identify patients with T2D initiating DU or BI between Nov’14–Apr’17 (index date = earliest fill date). Patients ≥ 18 years, with no claim for any antidiabetic injectable in the 6 months pre-index period (baseline), continuous enrollment and ≥ 1 HbA1c result at baseline and 1-year post-index were included. Two widely used measures for assessing persistence of injectables in the real-world were implemented. DU users were propensity-matched 1:1 to BI users.

**Results:** Matched cohorts (903 pairs) were balanced in baseline patient characteristics with mean age of 54 years. At 1-year follow-up, matched DU patients were significantly more likely to be adherent [PDC ≥ 80%, n (%)] than BI patients [516 (57.1%) vs. 262 (29%); p < .001). When measuring persistence as no gap between fills > 45 days [n (%)], more BI [605 (67.0%)] vs. DU [367 (40.6%)] patients discontinued their therapy but more BI [422 (69.8%)] vs. DU [141 (38.4%)] patients restarted their index therapy. More DU [519 (57.5%)] vs. BI [317 (35.1%)] patients discontinued based on the 90th percentile measure, and more DU [320 (61.7%)] vs. BI [126 (39.7%)] patients restarted their index therapy.

**Conclusion:** In this real-world study, DU demonstrated higher adherence than BI. Given the results, the most appropriate persistence measure may vary for different classes of antidiabetic injectables.

### P11 Adhesion of diabetic patients to dietary treatment

#### Ticiane De Oliveira Albuquerque, Alesson Silva Damasceno, Aldona Kubica^2^, Helena Alves De Carvalho Sampaio, Mayanne Iamara Santos De Oliveira Porto, Luiza De Carvalho Almeida, José Tercio Pereira De Carvalho, Carlos Cardoso Neto

##### Universidade Estadual do Ceará, Fortaleza, Brazil; ^2^Nicolaus Copernicus University, Torun, Poland

*Diabetology & Metabolic Syndrome* 2019, **11(Suppl 1):**P11

**Introduction:** Type 2 Diabetes Mellitus (T2DM) is a chronic disease of multifactorial nature, considered a public health problem, with high rates of morbidity and mortality.

**Objective:** To evaluate the adherence of diabetic individuals to dietary treatment.

**Methodology:** The study was conducted in the city of Fortaleza, in a Primary Health Care Unit (UAPS). The sample consisted of 37 users with diabetes, aged 18 years or older, both sexes and literate, with no previous history of heart atack or coronary surgery. In addition to people who did not meet the above criteria, pregnant women and those with cognitive problems that compromised their understanding of the interview topics were excluded. An adherence scale was applied, which is in the process of validation and contains 10 questions. The scale was developed in partnership with researchers from Brazil and Poland. Each item has 5 answer alternatives, with scores ranging from 0 to 4 on each item, with a maximum total of 40 points. The scale contained areas that included questions about knowledge of adequate nutrition, following the guidelines offered by professionals, discussing feeding with professionals and behaviors related to changing eating habits with or without professional consent. To classify the quality of adherence, the cutoff point of 75% was adopted, so that adherence was considered satisfactory if the individual reached at least 30 points. The study was submitted and approved by the Research Ethics Committee of the State University of Ceará, through PlataformaBrasil, CAAE 180546130.0000.5534. Results: Of the 37 patients, 34 were women. Low adherence was prevalent, 22 (59.5%) patients. This is a matter of concern, considering the importance of proper dietary management in the presence of T2DM.

**Conclusion:** The evaluated diabetic patients have low adherence to the oriented diet. The situation demands educational actions to promote the improvement of this adherence.

### P12 Adhesion of hypertense patients to dietary treatment

#### Anael Queirós Silva Barros^1^, Alesson Silva Damasceno^1^, Aldona Kubica^2^, Helena Alves De Carvalho Sampaio^1^, Mayanne Iamara Santos De Oliveira Porto^1^, Luiza De Carvalho Almeida^1^, José Tercio Pereira De Carvalho^1^, Carlos Cardoso Neto^1^

##### ^1^Universidade Estadual do Ceará, Fortaleza, Brazil; ^2^Nicolaus Copernicus University, Torun, Poland

*Diabetology & Metabolic Syndrome* 2019, **11(Suppl 1):**P12

**Introduction:** Systemic Arterial Hypertension (SAH) is a chronic multifactorial disease and constitutes a risk factor for the development of other cardiovascular diseases. Diet therapy is a non-drug treatment strategy for hypertension, but loses much of its effectiveness due to the low adherence of patients to nutritional recommendations.

**Objective:** To verify the adherence of hypertensive individuals to dietary treatment.

**Methods:** Validation clipping of the dietary adherence scale developed by researchers in Brazil and Poland. Data were collected at Primary Health Care Units (UAPS) in Fortaleza. A sample of 91 hypertensive patients, aged ≥ 18 years, both sexes, literate, with no previous history of heart atack or coronary surgery. Pregnant women with cognitive problems that compromised the understanding of the interview were excluded, as well as those who did not meet the above requirements. A 10-item dietary adherence scale was applied, with 5 alternatives and a score ranging from 0 to 4. Final score ranges from 0 to 40. The scale contained areas that included questions about knowledge of adequate nutrition, following the guidelines offered by professionals, discussing feeding with professionals and behaviors related to changing eating habits with or without professional consent. Satisfactory adherence was considered when 75% (≥ 30 points) were achieved. The study was submitted and approved by the Research Ethics Committee of the State University of Ceará, trough PlataformaBrasil, CAAE 180546130.0000.5534.

**Results:** 68.1% (62) of respondents had poor adherence. This situation puts patients at risk to health because it can prevent proper control of blood pressure levels.

**Conclusion:** There is a need for the development of educational actions that sensitize this public to adherence to dietary guidelines to improve the control of hypertension.

### P13 Adhesion to diabetes mellitus type 2 medical treatment

#### Vicente Rubens Reges Brito, Mariana Rodrigues da Rocha, Letícia Gonçalves Paulo, Patrícia Regina Evangelista de Lima, Rafaela Pereira Lima, Bruna Araújo Gomes, Mayla Rosa Guimarães, Ana Roberta Vilarouca da Silva

##### Universidade Federal do Piauí, Picos, Brazil

*Diabetology & Metabolic Syndrome* 2019, **11(Suppl 1):**P13

**Introduction and objectives:** Adherence to drug treatment is critical for a good diabetic patient response to therapy. Thus, this study aimed to evaluate drug treatment in people with type 2 diabetes mellitus.

**Methods and results:** Analytical study and cross-sectional, conducted with 78 users with type 2 diabetes mellitus, accompanied by five Family Health Strategies in the city of Picos-Piauí. Data were collected through a form concerning sociodemographic data and the Brazilian version of the Treatment Adherence Measure, which one is used for to evaluate the behavior patient regarding daily use of prescription drugs. Data were collected from February to April 2018 and analyzed using the descriptive and analytical statistics. The study was approved by the Research Ethics Committee of the Federal University of Piauí, according opinion n. 2.429.535. The results indicated the predominance of female (71.8%), had between 50 and 60 years of age (61.5%), brown (56.4%), married or cohabiting with a partner (66.7%), with 0 to 9 years of study (52.6%), attended public schools (82.0%), retired people (52.6%), with a habit of reading at least one textual source (65.4%), monthly income greater than one salary to three minimum wages (47.4%), economy class C2 (21.8%). In the sample selection, 43.6% of people have never forgotten to take diabetes control drugs, 35.9% were rarely careless with the hours of taking medications, 87.2% never forgot to take their medication because they felt better, 89.7% never forgot to take them because they felt worse, 94.8% never took more pills than the prescribed amount, on his own initiative, for feeling worse, 65.4% never interrupted treatment because they let their drugs finished, and 89.7% never stopped taking their medications for any other reason, unless the doctor’s recommendation. The mean score for adherence to medication treatment was 5.51 ± 0.491 (mean ± standard deviation) which reveals that 87.2% of the patients were considered adherent to the therapy.

**Conclusion:** Thus, conclude that the most of the participants in the sample had adherence to drug treatment. The study is of fundamental importance for health professionals, since it allows knowing the profile of users with diabetes and promoting actions that encourage their participation in treatment.

**Keywords:** Medication adherence; Diabetes Mellitus; Adherence to treatment.

### P14 Allogenic adipose derived mesenchymal stem cells and vitamin d supplementation in patients with recent-onset type 1 diabetes mellitus: a 3-month follow-up study

#### Débora B. Araujo de Pina Cabral^1^, Joana Rodrigues Dantas^1^, Leandra S. Baptista^2^, Carmen Lúcia Kuniyoshi Rebelatto^3^, Carlos Eduardo Barra Couri^4^, José Egídio Paulo de Oliveira^1^, Lenita Zajdenverg^1^, Melanie Rodacki^1^

##### ^1^Federal University of Rio de Janeiro, Nutrology and Diabetes Department, Rio de Janeiro, Brazil; ^2^National Institute of Metrology, Quality and Technology (Inmetro), Rio de Janeiro, Brazil; ^3^Core Cell Technology, Pontifical Catholic University of Paraná, Curitiba, Brazil; ^4^University of Sao Paulo, São Paulo, Brazil

*Diabetology & Metabolic Syndrome* 2019, **11(Suppl 1):**P14

Adipose tissue-derived stromal/stem cells (ADMSCs) and vitamin D has emerged as a potential treatment for Type 1 Diabetes (T1D) due to their intrinsic regenerative capacity and immunomodulatory properties.

**Objective:** To evaluate safety and efficacy of ADMSCs infusion from healthy donors and daily cholecalciferol (VitD) supplementation in patients with recent-onset T1D, after a 3-month follow-up.

**Methods:** This is a prospective, dual-center, open trial, phase II, in which patients with recent onset T1D received a single dose of ADMSCs (1x10^6^ cells/kg) and cholecalciferol 2000UI/day for 3 months. They were compared to controls with standard insulin therapy. Adverse events, C-peptide (CP) after mixed meal, insulin dose, HbA1c and CD45^+^CD3^+^CD4^+^FoxP3^+^ T-cells frequencies (flow cytometry) were measured at baseline (T0) and after 3 months (T3). Time in range (TIR) and glycemic variability were calculated by retrospective analysis of 72 h continuous glucose monitoring system (CGM).

**Results:** 13 patients were included (8 received ADMSCs + VitD or group 1 and 5 standard insulin therapy or group 2), with a mean age of 26.7 ± 6.1 years and mean T1D duration of 2.9 ± 1.05 months. Adverse events were transient headache (n = 8), mild local reactions (n = 7), tachycardia (n = 4), abdominal cramps (n = 1), thrombophlebitis (n = 4), scotomas (n = 2), central retinal vein occlusion (n = 1, complete resolution). After intervention, insulin requirement was lower in group 1 than in group 2 (0.22 ± 0.17 vs 0.61 ± 0.26 IU/kg, p = 0.01), respectively. Two patients in group 1 became insulin free, for 4 and 8 weeks each. At T3, group 1 had larger reduction of HbA1C (1.21 ± 0.18 vs 0.94 ± 1.28%; p = 0.04), larger area under the curve of CP (211.20 ± 100.42 vs 106.05 ± 47.25 ng/ml min;p = 0.05) and more glucose reading in TIR (89.4 ± 13.5 vs 57.50 ± 9.19%; p 0.02) than group 2. Moreover, at T3 all patients (n = 8) of group 1 were in honeymoon phase (insulin dose ≤ 0.5 IU/kg and HbA1c < 7.5%) vs none in group 2 (p = 0.01). There was an inverse correlation between VitD vs insulin dose (p = 0.01; r = 0.77) and HbA1C (p = 0.03; r = 0.67). CD4 + FoxP3 + had a negative correlation with HbA1C (p = 0.01; r = 0.71), SD (p = 0.01, r = 0.82) and MODD (p = 0.04, r 0.77), but not with MAGE (p = 0.77; r = 0.12).

**Conclusion:** Allogenic ADMSCs infusion + cholecalciferol without immunosuppression proved to be safe and may be an effective approach for the treatment of patients with recent onset T1D, but a larger follow up is still required.

### P15 Analysis of cost–benefit of the most consumed sweeteners currently

#### Ana Paula Varela Sanches^1^, Josilene Lopes de Oliveira^1^, Maíra Schuchter Ferreira^1^, Camila Neves Rodrigues^2^

##### ^1^Universidade Estadual de Campinas, Campinas, Brazil; ^2^Universidade Federal de Minas Gerais, Belo Horizonte, Brazil

*Diabetology & Metabolic Syndrome* 2019, **11(Suppl 1):**P15

**Introduction:** Sweeteners are products that have emerged, as an alternative to the use of sugar, it has been described for use by diabetic people or people who need sugar restriction by medical recommendation. The most consumed sweeteners are non-caloric, including aspartame, sucralose, sodium saccharin and steviol glycoside. With this great tendency to always follow the physical pattern that is imposed by society, many people end up changing their habits, and include in their routine the use of sweeteners. Comparing the price of these products in the market, people often end up opting for the product that has the lowest price. The consequences are that the lowest price can have adverse effects on other parameters. From the exposed data, the objective of this study was to verify the sweetener types that are most consumed today and to identify their cost–benefit.

**Methodology:** We searched for information regarding the categories of sweeteners present in the food market and compared the sweetener cost with the advantage of the product.

**Results:** We listed 23 types of sweeteners, including liquid and powders sweeteners, which can be used at high temperatures for recipe preparation, and those common, used day-by-day. Among them is cyclamate/Saccharin, that is a bitter taste sweetener, some studies have shown that it can have adverse effects on rats, but is widely used for its price, our research showed that this type of sweetener is among the cheapest. Sucralose is the intermediate sweetener, its structure is formed by two carbohydrate molecules, high sweetening power and under normal temperatures is not carcinogenic. However, when heated becomes harmful, can generate mutations, be absorbed and accumulated in the body. Aspartame is among the most expensive sweeteners, is made from two natural proteins, has no bitter taste and is more similar to sugar, compared to other sweeteners, no carcinogenic effect of this compound has been reported in experimental studies. However, it is the more expensive sweetener wich makes buying difficult, especially for people who make continuous use.

**Conclusion:** Currently we can find various types of sweeteners in the food market, but those with less adverse effects are usually more expensive and consequently less consumed.

### P16 Analysis of the efficacy of ginger in reducing glycemic levels of adults with type 2 diabetes

#### Gerdane Celene Nunes Carvalho^1^, Márcio Flávio Moura de Araújo^3^, José Cláudio Garcia Lira Neto^2^, Laise Maria Formiga Moura Barroso^1^, Vírginia Leyla Santos Costa Urtiga^2^, Fabiana Nayra Dantas Osternes^1^, Fabrícia Kelly Moura Dantas^1^, Marta Maria Coelho Damasceno^2^

##### ^1^Universidade Estadual do Piauí, Teresina, Brazil; ^2^Universidade Federal de Ceará, Fortaleza, Brazil; ^3^Fundação Oswaldo Cruz, Rio de Janeiro, Brazil

*Diabetology & Metabolic Syndrome* 2019, **11(Suppl 1):**P16

**Introduction and objectives:** Controlling type 2 diabetes mellitus (T2DM) has been a challenge, and factors such as insufficient knowledge about the disease or clinical inertia appear as strong obstacles to management and self-care. The urgency for more effective and low-cost solutions has made health policies directional, and researchers have begun to highlight the promising effect of ginger. Although widely used in cooking, ginger has been tested modestly in studies focused on reducing glycemic levels of people with diabetes. The aim of this study was to analyze the efficacy of ginger in reducing glycemic levels in adults with T2DM.

**Methods:** A randomized, double-blind, placebo-controlled clinical trial conducted of December 2017 to June 2018 in health units, in Picos-PI. Patients should be adults with T2DM, glycated hemoglobin (HbA1c) of 6–10%, and taking oral antidiabetic agents. Of the 229 patients recruited, only 142 were randomized into control group (CG) or experimental group (EG). In EG patients received capsules of 600 mg of ginger powder, and those of CG, 600 mg capsules of placebo. Both groups were instructed to take two capsules a day for 90 days, with the oral antidiabetic. Clinical and laboratory variables were collected at the baseline and after the intervention. In the analysis of the characteristics of the groups were used Mann–Whitney U test, and in the verification of the behavior of the numerical data, the Wilcoxon test, significance of 5%. The study was approved by the Research Ethics Committee of the State University of Piauí (2,248,450).

**Results:** Only 103 completed the study (47 in EG and 56 in CG). Most of the participants had a mean age of 58.64 years; they were female (69.9%), brown (54.4%), married (60.2%), diagnosed for 2–5 years of diabetes (40.8%), and a monthly income equal to or less than USD$243.00 (52.4%). CG patients had a 5% reduction in mean fasting blood glucose (185.23–175.98, p = .041), and 0.06% in HbA1c (8.36–8.29, p = .361). The EG patients had a 14.5% reduction in mean fasting blood glucose (203.60–174.04, p = 0.001), and a 0.26% reduction in HbA1c (8.40–8.14, p = .144).

**Conclusion:** Ginger seems to have aided in reducing glycemic levels, when in doses of 1.2 g/day, for 3 months. However, generalizations about the results cannot yet be made and more research is needed in different scenarios. Financial Support: this research was supported by the National Council for Scientific and Technological Development (CNPq).

### P17 ancestry but not self-reported color/race is associated with diabetic retinopathy in type 1 diabetes: a nested case–control study in Brazil

#### Deborah Conte Santos^1^, Laura Gomes Nunes de Melo^1^, Marcela Haas Pizarro^1^, Bianca Senger Vasconcelos Barros^1^, Carlos Antonio Negrato^2^, Luis Cristovão Porto^3^, Dayse Aparecida Silva^4^, Marilia Brito Gomes^1^

##### ^1^Universidade do Estado do Rio de Janeiro, Departamento de Medicina Interna, Diabetes, Rio de Janeiro, Brazil; ^2^Associação dos Diabeticos de Bauru, Bauru, São Paulo; ^3^Universidade do Estado do Rio de Janeiro, Laboratorio de Histocompatibilidade e Criopreservação (HLA), Rio de Janeiro, Brazil; ^4^Universidade do Estado do Rio de Janeiro, Laboratorio de Diagnostico DNA (LDD), Rio de Janeiro, Brazil

*Diabetology & Metabolic Syndrome* 2019, **11(Suppl 1):**P17

**Introduction:** The influence of genetic factors on the development and progression of diabetic retinopathy is still unclear. Previous studies showed controversial results.

**Objective**: We aimed to characterize the relationship between genomic ancestry and self-reported color-race with severe diabetic retinopathy in patients with type 1 diabetes belonging to a highly admixed population.

**Methods:** This study was a nested case–control based on data collected from a large cross-sectional, nationwide survey conducted in clinics from all 5 geographic regions of Brazil. For the present study, we included 414 individuals. Cases (n = 176) were considered if they had severe non-proliferative or proliferative diabetic retinopathy and controls (n = 238) were type 1 diabetes patients without retinopathy, matched for diabetes duration by range of 5 years. Indirect ophthalmoscopy was performed and individual genomic ancestry was inferred using a panel of 46 ancestry informative markers.

**Results:** The backward stepwise logistic regression analysis showed that African genomic ancestry (OR 3.9, p = 0.045), HbA1c (OR 1.24, p = 0.001), glomerular filtration rate (OR 0.98, p < 0.001) and hypertension (OR 2.52, p < 0.001) were associated with severe diabetic retinopathy after adjusting for clinical and demographic data. Self-reported color-race was not statistically associated with diabetic retinopathy.

**Conclusions:** Genomic ancestry as well as clinical variables such as hypertension, impaired glomerular filtration rate and poor diabetes control (HbA1c) was important risk factors for the development of severe diabetic retinopathy. Further studies are needed, especially in highly admixed populations, to better understand the role of genomic ancestry and possible genes that might be associated with the development and/or progression of diabetic retinopathy.

Finanial support was granted by FAPERJ and CNPq

### P18 Android-to-gynoid fat mass index and high calcium score are predictors of diabetic polineuropathy

#### Vaneza Lira Waldow Wolf, Ikaro Soares Santos Breder, Mauro Alexandre Pascoa, Gil-Guerra Junior, Otavio Rizzi Coelho-Filho, Andrei Carvalho Sposito

##### Faculdade de Ciências Médicas da Universidade Estadual de Campinas, Campinas, Brazil

*Diabetology & Metabolic Syndrome* 2019, **11(Suppl 1):**P18

**Introduction:** Diabetic polyneuropathy (DPN) is one of the most common complications in diabetic patients and is involved in risk amputation. Although it’s importance, full pathogenesis is not completely understood. The most important etiologic factors include diabetes duration, chronic hyperglycemia, age, hypertension, metabolic disorders and obesity.

**Objective:** To investigate the association between the main factors involved in diabetic polyneuropathy.

**Methods:** 77 patients aged 40 to 70 years-old with Type 2 Diabetes (T2DM) were submitted Michigan Score to evaluate the presence or absence of diabetic polyneuropathy (DPN or ADPN, respectively). Measures of Total Body, Android and Gynoid Fat Mass were performed by DXA. Calcium Score (CS) was done by cardiac computed tomography. Laboratory tests were performed at Fleury laboratory. Statistical analyses were performed using SPSS 20. Continuous data were compared by the Mann–Whitney U and Pearson’s χ2 tests. The Odds Ratio was obtained by binary logistic regression and p-values less than 0.05 were considered statistically significant.

**Results:** The mean age was 58.69 years-old, 57.40 years-old in ADPN group and 60.72 years-old in DPN group (p > 0.05). From the total, 30 patients (38.96%) presented DPN. Mean time of Diabetes was 9.55 years (95% CI 8.05; 11.05), without differences between the groups. Patients with DPN presented higher values of android/gynoid fat mass index (ADNP: 0.664; DPN: 0.838, 95% CI 0.775–0.915, p < 0.001). However, total body fat and Gynoid Fat were positively associated to ADNP (p = 0.034 and p = 0.034, respectively). Besides, CS > 100 was associate with DPN (p = 0.038). After regression tests, android/gynoid fat mass index maintained associated with DNP (OR 0.014, 95% CI 0.001–0.212, p = 0.002), while Gynoid Fat Mass was associated to protection against DNP (OR 1.53, 95% CI 1.05–2.23, p = 0.027).

**Conclusion:** Increased Android/Gynoid Fat Mass Index and CS > 100 are predictors of DNP in patients with T2DM.

### P19 Android-to-gynoid fat mass index is a strong predictor of high calcium score in patients with type 2 diabetes

#### Íkaro Soares Santos Breder, Vaneza Lira Waldow Wolf, Michele Santana, Otávio Rizzi Coelho Filho, Mauro Alexandre Pascoa, Gil Guerra-Junior, Andrei Carvalho Sposito

##### Faculdade de Ciências Médicas da Universidade Estadual de Campinas, Campinas, Brazil

*Diabetology & Metabolic Syndrome* 2019, **11(Suppl 1):**P19

**Introduction:** Both obesity and Calcium Score (CS) are known factors related to elevated cardiovascular mortality in diabetic patients. Although, BMI does not distinguishes neither body fat composition nor total fat tissue. We hypothesize that android/gynoid fat mass rate measured by Dual-energy X-ray absorptiometry (DXA) is a stronger predictor for elevated CS than BMI.

**Objective:** To evaluate if android/gynoid fat mass rate is better than BMI to predict higher CS.

**Methods:** 154 patients with 40 to 70 years-old with diagnosis of Type 2 Diabetes (T2DM) were submitted to DXA and CS. Women in fertile age were excluded. Agatston was categorized between lower or higher than 100. Statistical analyses were performed using SPSS 20. Association of body fat composition values with CS were performed using Spearman’s correlation coefficient.

**Results:** Mean age was 59.78 years-old, 61.59 in CS > 100 and 59.38 in CS < 100 (p = 0.100). Mean time of Diabetes was 7.594 years (95% CI 8.221–10.543), 7.0 years (95% CI 7.872–10.428) in CS < 100 and 9.709 (95% CI 7.511–13.342) in CS > 100 (p = 0.387). Patients with CS ≥ 100 presented higher values of android/gynoid fat mass index (0.620, 95% CI 0.597–0.655, p < 0.001), but not higher BMI (31.178, 95% CI 29.556–32.829, p = 0.266), android fat mass (3.460, 95% CI 3.034–3.886, p = 0.170) or total fat mass (30.627, 95% CI 27.791–33.464, p = 0.789).

**Conclusion:** Android/Gynoid fat mass index measured by DXA is the major value to predict higher CS when compared to BMI or another’s values from DXA.

### P20 Ankle-brachial index at assessment cardiovascular risk in diabetic patients

#### Adeline Brito Sales, Clara Simony de Sousa Santos, Tiago da Silva Nunes, Thayse Santos Barros, Daniel Matos Euzébio de Queiroz da Cruz, Bruno Matheus Souza Xavier, Marco Antônio Prado Nunes, Carla Raquel Pereira Oliveira

##### Universidade Federal de Sergipe, Aracaju, Brazil

*Diabetology & Metabolic Syndrome* 2019, **11(Suppl 1):**P20

**Introduction:** Diabetes Mellitus corresponds to a Cardiovascular Risk Factor of great importance in clinical practice, being Cardiovascular Diseases (CVD) the main causes of mortality associated with the diabetic population. In this context, the Ankle-Brachial Index (ABI) is presented as a simple and easy-to-perform tool capable of early detection of cardiovascular impairment in individuals. Thus, the present study aimed to evaluate the presence of alterations in ABI in diabetic patients.

**Methods:** For this, a cross-sectional study was conducted, in which clinical data of 293 diabetic patients followed in a public reference service in the city of Aracaju-SE were evaluated, according to ethics committee approval (4202.0.000.107-07). Based on the value of ABI, these were classified as definitive PAD (ABI ≤ 0.09), Borderline (ABI between 0.91 and 0.99), Normal Arteries (ABI 1.00–1.29) and Arterial Calcification (ABI ≥ 1.3). Categorical variables were analyzed using Pearson’s Chi square test or Fischer’s exact test, while numerical variables were analyzed by Mann–Whitney and Kruskal–Wallis, considering p ≥ 0.05 as significant.

**Results**: Of the patients evaluated, 51.19% had ABI value considered as altered, and of these, 48.8% corresponded to the Arterial Calcification group. The definitive PAOD, in turn, represents 13.5% of patients with altered ABI, being related to older age, longer DM and the presence of DM complications such as neuropathy, retinopathy and amputations. Finally, 3.07% of patients with altered ABI belong to the Borderline group, which, when compared to the other groups, had a lower average age, 48.8 years compared to 60, which may indicate an initial stage of definitive PAD.

**Conclusion:** Given these data, the importance of the use of ABI as a tool capable of early diagnosis of asymptomatic CVD in diabetic patients is observed. This is a low cost and easy applicability tool that can contribute to the reduction of morbidity and mortality population.

### P21 Antidiabetic, antioxidant, and anti-inflammatory effects of purified s-methyl cysteine sulfoxide in streptozotocin-induced diabetic rats

#### Licyanne Ingrid Carvalho de Lemos^1^, Matheus Anselmo Medeiros^1^, João Paulo Matos Santos Lima^1^, Camila Alexandrina Figueiredo^2^, Flavio Santos da Silva^3^, Bento João Abreu^1^, Karina Carla de Paula Medeirose^1^, Lucia Fatima Campos Pedrosa^1^

##### ^1^Universidade Federal do Rio Grande do Norte, Natal, Brazil; ^2^Universidade Federal da Bahia, Salvador, Brazil; ^3^Universidade Federal Rural do Semi-Árido, Mossoró, Brazil

*Diabetology & Metabolic Syndrome* 2019, **11(Suppl 1):**P21

**Introduction:** The use of medicinal plants and isolated substances plants have been discussed as therapeutic strategies in Diabetes mellitus (DM). S-methyl cysteine sulfoxide (SMCS) is a hydrophilic compound naturally found in plants known for possessing antidiabetic and antioxidant properties and effects in the morphological restorationof tissues. The study aims to explore the antidiabetic, antioxidant, and anti-inflammatory effects of SMCS and investigate histopathological changes in the hepatic and pancreatic tissues in streptozotocin (STZ)-induced diabetic rat models.

**Methods:** Twenty-five 90-day-old male Wistar rats (*Rattus norvegicus*) were divided into the following groups: non-diabetic rats control group (CG), STZ-induced diabetic rats (STZ-DB), and STZ-induced diabetic rats treated with SMCS (STZ-SMCS). SMCS (200 mg/kg) was administered daily by gavage for 30 days. After five days from the induction of DM, rats with glucose levels of ≥ 250 mg/dL were considered diabetic. Biochemical and cytokine analyses, catalase (CAT) and superoxide dismutase (SOD) activities assays and histopathological analysis of liver and pancreas tissues were performed. The local Committee on the Ethics of Animal Use and Care approved the study (no. 012.018/2017).

**Results:** The results were expressed as mean (standard error). STZ-SMCS group showed significant decrease in glycemia compared to STZ-DB [521 (9.3) mg/dL vs. 686 (13.9); p < 0.05]. The SCMS treatment significantly decreased VLDL [from 30.0 (5.45) to 10.0 (3.64); p < 0.01], and triglycerides levels [from 154.0 (24.8) to 54.0 (19.13); p < 0.01]. CAT (nmol/min/mg Prot) and SOD (UA/min/mg Prot) activities significantly increased in STZ-SMCS group compared to STZ-DB (both *p < 0.01); 5111.4 (681.4) vs. 2117.4 (326.9) and 409.9 (77.8) vs. 109.2, respectively. SMCS also mitigated the damage in pancreatic islets, enabling them to recover their morphology and improved hepatic glycogen depletion. Although SMCS failed to reduce the levels of the pro-inflammatory cytokines IL-6 and IL-1β, it caused an increase in IL-10.

**Conclusions:** SMCS can improve the metabolic profile of DM, relieve oxidative stress in the liver, and assist with the inflammatory response. Thus, these findings suggest their potential therapeutic properties.

**Financial support:** Coordination for the Improvement of Higher Education Personnel-CAPES (Finance Code 001).

### P22 Are patients with type 1 diabetes on insulin regimens of multiple daily injections being adequately treated?

#### Breno Carvalho Cirne de Simas, Marcelino Bernardo de França Neto, Jihane Paiva de Carvalho, Camila Pereira Carvalho, Flora Tamires Moura Bandeira, Felipe Costa de Albuquerque, Maria de Fátima Paiva Baracho, André Gustavo Pires de Sousa

##### Universidade Federal do Rio Grande do Norte/Hospital Universitário Onofre Lopes, Natal, Brazil

*Diabetology & Metabolic Syndrome* 2019, **11(Suppl 1):**P22

**Introduction:** Type 1 Diabetes (T1D) requires insulin replacement in the most physiological fashion achievable. In those with T1D on Multiple Daily Injections (MDI) insulin regimens, the most adequate daily dosing proportions comprise of 40 to 50% Basal Insulin (BI) and 50 to 60% Prandial Insulin (PI). Insulin analogues (IA) are safer, more efficient than Human Insulin (HI), and should be preferred for those with T1D. Also, patients should be instructed to determine mealtime dosing based on carbohydrate counting and correction factors to achieve better glycemic control.

**Objective:** To characterize a cohort of patients with T1D and evaluate the adequacy of their insulin therapy.

**Methods:** We conducted a cross sectional study in a cohort of patients with T1D assisted at an endocrinology clinic at an university hospital. Data was extracted from patients’ last visits and included glycated hemoglobin (A1c), diabetes related complications, body mass index (BMI), type of insulins and doses used, and whether patients were instructed on carbohydrate counting and correction factors to calculate PI dosages. We considered the proportion of insulin dosages adequate if PI daily dose represented 50 to 60% of total daily dose.

**Results:** Our sample included 58 patients (58.6% female), mean age 24.8 ± 7.6 years, and median disease duration 8.0 years. Mean A1c was 9.12 ± 2.22%, mean BMI was 23.4 ± 3.9 kg/m^2^, 24.1% had chronic kidney disease, 12.7% had retinopathy. Regimens included only HI in 38.6%, only IA in 54.4% and mixed regimens in 7.0% of patients, with a mean total dose of 0.78 ± 0.33U/kg. The median proportion of the total daily dose constituted of PI was 44%. In 27.6% of patients proportion was adequate, in 65.5% and 6.9% there was BI and PI predominance, respectively. Only 24.1% of patients were instructed on using correction factors to determine prandial insulin dosages and only 10.3% performed carbohydrate counting. A1c was lower in those who used correction factor (mean difference 1.76%, 95%CI 0.4–3.1%, p = 0.012). A1c was not significantly different according to performance of carbohydrate counting (p = 0.11), types of insulin (p = 0.19), nor adequacy of PI proportion (p = 0.14).

**Conclusions:** Only approximately half the patients were on analogues, on quarter were using adequate proportions, one quarter used correction factors and a tenth performed carbohydrate counting. Increasing the use of these tools on insulin regimens might improve glycemic control.

**Financial support:** None

### P23 Are there differences in maternal–fetal outcomes according to the trimester when gestational diabetes is diagnosed?

#### Martha Camillo Jordão, Filipe Dias de Souza, Rosiane Mattar, Bianca de Almeida Pititto, Sergio Atala Dib, Patricia Medici Dualib

##### Universidade Federal de São Paulo, São Paulo, Brazil

*Diabetology & Metabolic Syndrome* 2019, **11(Suppl 1):**P23

**Introduction:** Gestational diabetes mellitus (GDM) is a significant public health problem due to associated perinatal morbidity and long-term metabolic risk. According to the IADSG criterion, based on the findings of the Hyperglycemia and Adverse Pregnancy Outcome (HAPO) study, the diagnosis of early GDM occurs in the first trimester when fasting blood glucose (FBG) is ≥ 92 mg/dl and previous diabetes mellitus (DM) if FBG is ≥ 126 mg/dl. However, there are still few studies verifying differences in the prevalence of maternal and fetal outcomes in pregnant women diagnosed with early GDM.

**Objective:** To compare fetal-maternal outcomes among women with diagnosis of GDM in the 1st versus 2nd and3rd trimesters of pregnancy.

**Methods:** 801 pregnant women diagnosed with GDM (IADPSG criteria) were evaluated at a tertiary public service from 2007 to 2018. The women were stratified by: those diagnosed with DM in the 1st trimester (FBG ≥ 92 mg/dl, n = 128) and those who had the diagnosis in the 2nd or 3rd trimester (OGTT: fasting ≥ 92 and/or 1 h ≥ 180 and/or 2 h ≥ 153 mg/dl, n = 673). Age, BMI and HbA1c data, and maternal (specific gestational hypertension, preeclampsia and cesarean delivery) and fetal outcomes (jaundice, respiratory distress, ICU stay, hypoglycemia and malformations) were compared between groups by Student t test or Chi square test, p < 0.05.

**Results:** Maternal age, BMI and HbA1c were not different between groups. Prevalence of maternal outcomes and fetal outcomes also did not differ between the two groups. In addition, pregnant women diagnosed with DM in the first trimester were subdivided into: FBG ≥ 126 mg/dl (previous DM, n = 69) and FBG ≥ 92 and < 126 mg/dl (GDM, n = 59). Those with previous DM had higher HbA1c (6.1 ±0.9% vs 5.5 ±0.5%, p < 0.01) compared with GDM, respectively, but there was no difference regarding the prevalence of maternal and fetal outcomes.

**Conclusions:** Our results show that the gestational age at which the diagnosis of GDM was made did not influence the occurrence of maternal–fetal outcomes. Therapeutic approach of GDM patients in a tertiary service with an interdisciplinary team may be a better prognostic factor. However studies with a larger group of pregnant women comparing the two blood glucose values ​​(≥ 126 mg/dl and ≥ 92 mg/dl) for the diagnosis of GDM in the first trimester of pregnancy with respect to maternal–fetal outcomes are required.

**Financial support:** CAPES.

### P24 Assessment of hypoglycemia in non-diabetic patients in a high complexity service in southeastern Brazil

#### Maisa Monseff Rodrigues da Silva^1^, Anne Elise Alexandre^1^, Maria Amelia Aquino Montenegro de Andrade^1^, Marina Guedes Carvalho^1^, Raoni Pardi Moreira^1^, Patricia Moreira Gomes^1^, Maria Cristina Foss de Freitas^2^, Matheus Pereira Salgado^1^

##### ^1^Divisão de Endocrinologia, Dpt de Medicina Interna do HC FMRP-USP, Ribeirão Preto, Brazil; ^2^Docente da Disciplina de Endocrinologia e Metabologia da Faculdade de Ribeirão Preto – USP, Ribeirão Preto, Brazil

*Diabetology & Metabolic Syndrome* 2019, **11(Suppl 1):**P24

**Introduction:** Hypoglycemia, defined by the Whipple triad, is uncommon in individuals without diabetes mellitus and requires careful evaluation.

**Objective:** To analyze cases of hypoglycemia in hospitalized patients between January 2000 and June 2019 at a right complexity hospital in the state of Sao Paulo, Brazil.

**Method:** This is a retrospective study of 40 cases of hypoglycemia through medical records the patients had a previous diagnosis of hypoglycemia in a health unit on admission, hypopituitarism, leukemia, hepatic, renal and adrenal failure were ruled out blood glucose, insulin, proinsulin and c-peptide were collected. 70% of patients (28) underwent a 72-h prolonged fasting test.

**Results:** 57.5% of the patients were women; the mean age was 57 years. Reported symptoms, were loss of consciousness (60%), seizures (37.5%), sweating (57.5%), tremors (47.5%), visual clouding (40%), mental confusion (50%), palpitations (27.5%) and nausea (10%). Improvement of symptoms with diet occurred in 55% of cases. The mean time to diagnosis was 28.7 months. 19 patients had hypoglycemia while performing supervised prolonged fasting test, with an average of 16 h for its occurrence. Laboratory hypoglycemia was confirmed in 32 patients (80%), with a mean glycemia of 42.6 mg/dl and insulin of 60.2 uu/ml. The diagnoses were: 14 cases of insulinoma, 4 cases of primary islet hyperplasia (hpi), 4 cases of factitious, 4 cases of endogenous hyperinsulinemia without insulinoma image, 2 due to inadvertent use of sulphonylurea, 2 after bariatric surgery, 2 reactive hypoglycemia, 1 of hypoglycemia secondary to igfii production by tumor (liver metastasis of hemagiopericytoma) and 1 of persistent hyperinsulinemic hypoglycemia of childhood. 2 patients lost follow-up, 3 had no confirmed hypoglycemia. Thirteen patients with insulinoma and four with hpi (1 in another service) were operated. The insulinomas were located: 7 in the head (53%), 2 in the tail (15%), 1 in multiple locations (7%), 2 in the body (15%) and 1 still awaiting pathology report.

**Conclusion:** Prolonged supervised fasting helped to rule out factitious hypoglycemia. In cases of endogenous hyperinsulinemia hypoglycemia was achieved within a few hours of fasting. Neuroglycopenic symptoms were more prevalent than adrenergic, probably due to the long term symptoms. The literature reports a homogeneous distribution of insulinomas in the pancreatic segments. In our series, we observed a higher prevalence in the head of the pancreas.

### P25 Assessment of risk of falls, static and dynamic balance in individuals with type 2 diabetes mellitus

#### Douglas Sousa Matos^1^, Maria Carolina Simões Vieira de Melo^1^, Alice Sá Carneiro Ribeiro^2^, Diana de Andrade Silva^1^, Danilla Maria do Nascimento^1^, Hélida Cristina Cirilo da Silva^1^, Izabelly Karina Santos Rodrigues^2^, Silvia Regina Arruda de Moraes^1^

##### ^1^Universidade Federal de Pernambuco, Recife, Brazil; ^2^Universidade Estácio de Sá, Recife, Brazil

*Diabetology & Metabolic Syndrome* 2019, **11(Suppl 1):**P25

**Introduction:** The symptomatic complication of higher incidence in the type 2 diabetes mellitus (DM2) is peripheral neuropathy, found in 50% of individuals over 60 years of age. Peripheral neuropathy leads to sensory and motor deficits which, in turn, result in changes in gait and postural balance, as well as increased risk of falls (Diabetologia, vol.53, pp. 458–466, 2010, JRRD, vol 49, pp. 333–338, 2012).

**Objectives:** To evaluate the static and dynamic balance, as well as the risk of falls in individuals with DM2, regardless of the presence of diabetic neuropathy.

**Methods:** A cross-sectional study with a sample of 33 individuals of both sexes, aged 45 years or over, diagnosed for DM2 for at least 3 years. The evaluations of the postural balance and the risk of fall were performed with the Biodex Balance System SD in the Postural Stability Test (BBS-PS), Limits of Stability Test (BBS-LOS) and Fall Risk Test (BBS-FR) modalities. The study was approved by the Ethics and Research Committee under CAAE: 84511518.8.0000.5208.

**Results:** The mean age was 62.33 years (± 7.90), predominantly female (28/33 individuals), Body Mass Index 30.32 (± 4.51) and diabetes time 11.93 years (± 8.12). The mean values obtained from the BBS-PS analysis were: OSI (overall stability index) = 1.2 (± 1.05), APSI (anterior–posterior stability index) = 1.39 (± 0.98), LRSI (medial–lateral stability index) = 1.05 (± 0.50); of BBS-LOS: OSLI (overall stability limits index) = 19.94 (± 8.51); of BBS-FR = 2.03 (± 0.90).

**Conclusion:** The results of BBS-PS and BBS-FR are in agreement with the findings in the literature for healthy elderly individuals with the same mean age. However, differences in sample size and methodology were verified when compared to the present study. In regard to the BBS-LOS result, this was lower than expected reaching only 19.94/65 points. Therefore, the was a change in the dynamic balance of the analyzed sample. The generalization of the results of this study is limited due to the small sample size employed.

### P26 Association between functionality and handgrip strength of diabetic patients

#### Auristela Duarte de Lima Moser, Glenda Naila de Souza, Bruna Isadora Thomé, Fernanda Cury Martins Teigão, Maria Isabel Barboza Silveira, Jennifer Cristina Rabbers Vasconcelos, Cristina Pellegrino Baena

##### Pontifícia Universidade Católica do Paraná, Curitiba, Brazil

*Diabetology & Metabolic Syndrome* 2019, **11(Suppl 1):**P26

**Introduction:** The term Diabetes Mellitus (DM) describes a metabolic disorder that, in the long run, affects the muscular system and functional capacity. The functionality components include the biopsychosocial aspects of the individual’s life. Tracking these effects, including all spheres of functional capacity, is critical because they may influence self-care and management of DM.

**Objective:** To identify the association between functional capacity and handgrip strength in patients with DM.

**Method:** Quantitative observational and cross-sectional research. Held in an outpatient clinic of a high-complexity university hospital in Curitiba, Paraná, Brazil, with diabetic individuals over 20 years, following the protocol of the Report on Strengthening Observational Studies in Epidemiology (STROBE). The following protocols were applied: functionality score, World Health Organization Disability Assessment Schedule (WHODAS 2.0); Time Up Go functional mobility test (TUG), with association of motor and cognitive tasks; and measurement of Handgrip Strength (FPM). Epi Data Entry software was used for data tabulation and SPSS 21 for statistical analysis; Spearman’s test was used for correlations. The study was approved by the Research Ethics Committee under number 3030.003.

**Results:** The study included 168 people, with an average age of 59.38 years ± 13.23, diagnosed with DM for 12.01 years ± 9.81. The functionality score was 21.36% ± 17.17 indicating a low level of disability. The performance of functional mobility was unsatisfactory, the average time to perform the TUG was 11.89 s ± 4.92 and when associated with the cognitive and motor tasks, there was an increase of 1.81 s and 3.96 s respectively. The average of FPM was 32.85 kg/F ± 9.78, slightly below normal. Positive correlation was found between all variables related to functional capacity and FPM: WHODAS 2.0 and FPM (r = -0.296, p = 0.000); TUG and FPM (r = -0.384, p = 0.0000003); TUG associated with motor task and FPM (r = -0.252, p = 0.001); TUG associated with cognitive task and FPM (r = -0.343, p = 0.00001).

**Conclusion:** Functionality and muscle strength are interdependent and relevant dimensions for diabetic individuals. Within the sphere of functionality, the functional mobility associated with the motor task showed greater fragility, which reflects the difficulty of this population to have greater functional independence. Therefore, they should be considered in order to improve care in high complexity outpatient clinics.

### P27 Association between high glycemic variability and hba1c, hypoglycemic and hyperglycemic episodes and carbohydrate counting in adults with type 1 diabetes from a Brazilian Public Tertiary Hospital

#### Thais Barbarini Seabra Brasil^1^, Andrei Carvalho Sposito^2^, Walkyria Mara Gonçalves Volpini^3^, Elizabeth João Pavin^4^

##### ^1^Internal Medicine Postgraduate Program, Faculty of Medical Sciences, University of Campinas, Campinas, Brazil; ^2^AtheroLab, Cardiology Division, Faculty of Medical Sciences, University of Campinas, Campinas, Brazil; ^3^Neuroimmunology Unit, Biology Institut, University of Campinas, Campinas, Brazil; ^4^Endocrinology Division, Department of Internal Medicine, University of Campinas, Campinas, Brazil

*Diabetology & Metabolic Syndrome* 2019, **11(Suppl 1):**P27

**Introduction and objective:** The focus of *type 1 diabetes* (T1D) treatment is to maintain stability, both in mean blood glucose levels and glycated hemoglobin (HbA1C) rates, as well as in daily glycemic variability (GV). GV allows the current assessment of glycemic control and therapeutic adequacy. This study aimed to identify the GV values and their relationships with HbA1c, personal data, clinical and laboratory parameters, comorbidities and chronic complications of adult patients with T1D followed in a Brazilian Public Tertiary Hospital.

**Methods**: Patients aged ≥ 18 years and previously diagnosed with T1D for at least 6 months were included. All received face-to-face guidance to perform at least three seven-point glycemic profiles (on three consecutive days), totaling 21 blood glucose tests. After this monitoring, the patients returned to the clinic to download the monitor data, used for GV calculation, expressed as the standard deviation (SD) of the glycemic average, in mg/dL. For statistical analysis, Spearman correlation and Mann–Whitney test were used; significance level adopted of *5%*. CEP 80881317.7.0000.5404.

**Results**: Of the 40 patients studied, 70% were women, aged 37.1 ± 11.6 years (mean ± SD), T1D duration of 23.5 ± 9.2 years and HbA1c = 8.7% ± 1.8%. Comorbidities present: microvascular complications (77.5%), dyslipidemia (55%), arterial hypertension (40%) and primary autoimmune hypothyroidism (22.5%). The GV found was 69.1 ± 27.2 mg/dL (target < 50 mg/dL), presenting a positive correlation with: HbA1c (p < 0.0001), cholesterol levels (p = 0.0461) and triglycerides (p = 0.0460), episodes of hypoglycemia < 70 mg/dL (p = 0.0346) and hyperglycemia > 180 mg/dL (p < 0001) and negative with the percentage of blood glucose within target (70–180 mg/dL) (p < 0001). Patients who did not count carbohydrates had higher GV compared to those who did (78 mg/dL vs 57 mg/dL, respectively, p = 0.0127).

**Conclusion:** The patients with T1D treated in this public service presented high GV, associated with glycemic instability. Training of the medical staff and patients is required to be aware of the meaning and implications of this indicator, when assessed by systematic monitoring prior to consultation. Thus, strategies that improve the quality of glycemic control, such as carbohydrate counting therapy and pre-prandial blood glucose corrections can be accelerated.

**Financial support:** CAPES and FAEPEX.

### P28 Association between physical activity practice and lipid profile in metabolically healthy adult women

#### Flávio Weverton de Sousa, Patricia Pinho Cardoso, Fábia Karine de Moura Lopes, Mariana Pimentel Gomes Souza, Nathália Bernardo Marinho Leite, Lorena Taúsz Tavares Ramos, Virgínia Oliveira Fernandes, Renan Magalhães Montenegro Júnior

##### Universidade Federal do Ceará (UFC), Fortaleza, Brazil

*Diabetology & Metabolic Syndrome* 2019, **11(Suppl 1):**P28

**Introduction:** The impact of regular physical activity on health is well established in previous studies, reporting that exercise is an important in dependent factor in reducing the risk of morbidity and mortality. In this context, blood lipid levels are strongly correlated with cardiovascular diseases risk. It has been well established in previous studies that regular physical activity improves blood lipid profile by increasing High Density Lipoprotein (HDL) and lowering Low Density Lipoprotein (LDL) and Triglyceride (TG). Thus, high LDL cholesterol levels concomitantly with low HDL cholesterol levels leads to coronary heart disease progression, increasing the risk of death.

**Objective:** To investigate the association between physical activity practice and the lipid profile of adult women.

**Methods:** This is a cross-sectional study approved by the Ethics Committee (CPEA: 3.123.755), conducted with metabolically healthy adult women, aged 18 to 45 years, who attended the Clinical Research Unit at a hospital of reference in Fortaleza-Ceará, from October 2018 to June 2019. Weight, height and physical activity data were collected. Blood was collected for biochemical analyzes of Total Cholesterol (TC), LDL, HDL and TG. To verify the association between physical activity variables and biochemical indicators the Wilcoxon test was used, considering as significant p < 0.05. The normality of the variables was verified by the Shapiro–Wilk test.

**Results:** The sample consisted of 112 patients with a mean age of 29.7 (± 6.7) years. The mean BMI was 26.7 (± 5.4) kg/m^2^, most of them in inadequate nutritional status (54.5%). Regarding the practice of physical activity, most did not performany exercise (71.4%) and, among these, 60.4% had inadequate BMI. Regarding the weekly frequency of physical activity, the majority (87.5%) practiced over 3 times, with a daily frequency found in only 6.25% of those who reported practicing. There was a significant positive correlation between HDL values and physical activity (p < 0.01). The TC, LDL and TG fractions did not present significant correlation.

**Conclusions:** Physical activity showed no significant correlation with TC, LDL and TG fractions, but showed a significantly linked to higher HDL levels. The results of this study suggest that both physical inactivity and nutritional inadequacy were significantly associated with lower HDL levels.

### P29 Association between the ankle movement amplitude and the blood flow of neuropathic diabetic patients

#### Catarina Clapis Zordão, Aline Gobbi, André Timóteo Sapalo, Gabriela de Carvalho, Elaine Caldeira de Oliveira Guirro

##### Faculdade de Medicina de Ribeirão Preto - Universidade de São Paulo, Ribeirão Preto, Brazil

*Diabetology & Metabolic Syndrome* 2019, **11(Suppl 1):**P29

**Introduction:** Diabetes Mellitus (DM) is a disease of high incidence, and one of the main health problems in the world. Individuals affected by the disease are predisposed to reduced tibiotarsal joint mobility and impaired blood circulation.

**Objective:** To evaluate the relationship between range of motion and lower limb hemodynamic indices in the tibiotarsal joint in individuals with diabetic neuropathy.

**Methods:** Forty volunteers of both sexes with a mean age of 61.45 ± 7.05, with type 2 diabetes mellitus and diabetic peripheral neuropathy were evaluated. Arterial blood flow was assessed by Doppler ultrasound and joint range of motion by digital goniometry. Data distribution was verified through the Shapiro–Wilk test, followed by Pearson’s or Spearman’s correlation coefficient to verify the association between the variables.

**Results:** A significant association was found between the following variables: dorsiflexion and pulsatility index of blood flow from the left dorsal artery (rs = 0.49).

**Conclusion:** The range of motion of the tibiotarsal joint is related to the metabolic impairment of the neuropathic diabetic individuals evaluated.

**Financial support:** “Fundação de Amparo à Pesquisa do Estado de São Paulo (FAPESP)”.

### P30 Association of calcium and Pth concentrations with insulin resistance in overweight adolescents

#### Ana Beatriz Rodrigues Pinheiro, Maria Eduarda Bezerra da Silva, Thatyane Oliveira Souza, Jéssica Bastos Pimentel, Adriana Leão de Miranda Ferreira, Ricardo Fernando Arrais, Adriana Augusto de Rezende, Severina Carla Vieira Cunha de Lima

##### Universidade Federal do Rio Grande do Norte, Natal, Brazil

*Diabetology & Metabolic Syndrome* 2019, **11(Suppl 1):**P30

**Introduction:** Insulin activity is a mechanism associated with intracellular calcium, a fundamental micronutrient in mediating insulin response in muscle and adipose tissues. Studies show the association between changes in mineral concentrations and increased insulin resistance (IR), caused by reduced GLUT4 transporter activity. Thus, irregularities in calcium homeostasis have a harmful potential to insulin signaling pathways and can be signaled as a possible marker for IR.

**Objective:** To identify the association between the HOMA-IR index, calcium concentrations and PTH dosages.

**Methods:** Cross-sectional study, approved by the Research Ethics Committee, involving overweight adolescents between 10 and 19 years old, who attended a pediatric endocrinology outpatient clinic. Anthropometric data were collected - weight and height to obtain BMI, and biochemical - PTH, fasting blood glucose and fasting insulin. The last two were used to apply the Homeostatic Model Assessment (HOMA-IR) index. cut-off point ≥ 3.0 for insulin resistance. To test the association between variables, we used Pearson’s Chi square test or Fisher’s exact test. The correlation of quantitative variables was tested using Pearson’s correlation. For all tests we used a confidence level of 95% and considered statistically significant p < 0.05.

**Results:** Of the 85 adolescents evaluated, 41 (48.2%) were female, with a mean age of 11.42 years (± 1.48). The following means were recorded for BMI 26.72 kg/m^2^ (± 3.57), HOMA-IR index 2.64 (± 1.34), calcium 10.73 (± 0.52) and PTH 40.21 (± 19.73). Of the total sample, 32% were characterized as insulin resistant. There was a statistically significant difference in the HOMA-IR index value between genders (p = 0.027) and an association between the HOMA-IR index and BMI (p = 0.006). The HOMA-IR index was positively correlated with BMI (p = 0.001; r = 0.469), PTH (p = 0.117; r = 0.171) and calcium (p = 0.360; r = 0.100). There was a negative correlation between PTH and calcium (p = 0.861; r = -0.019).

**Conclusion:** The HOMA-IR index was not associated with calcium and PTH, suggesting that changes in these variables are not good predictors of decreased insulin response. However, BMI was an excellent marker of insulin resistance. This study was financed in part by the Coordenação de Aperfeiçoamento de Pessoal de Nível Superior - Brasil (CAPES) - Finance Code 001.

### P31 Association of dbp gene polymorphisms (rs4588 and rs7041) with adult-onset type 1 diabetes mellitus in Euro-Brazilians

#### Ana Karla Bittencourt Mendes, Susan Weber de Souza, Bruna Rodrigues Martins, Louryana Padilha Campos, Mateus Santana Lopes, Thanise Pitelli de Nigro, Geraldo Picheth, Fabiane Gomes de Morais Rego

##### Universidade Federal do Paraná, Curitiba, Brazil

*Diabetology & Metabolic Syndrome* 2019, **11(Suppl 1):**P31

**Introduction:** It has been suggested association between type 1 diabetes and insufficient levels of vitamin D, as well as polymorphisms within genes related to vitamin D pathways. Vitamin D binding protein (DBP) is the major carrier of vitamin D and its metabolites in plasma. 1,25-Dihydroxyvitamin D is a potent immunomodulator that also increases the production and secretion of several hormones, including insulin. Variants of the vitamin D-binding protein (DBP) have been associated with type 1 diabetes (T1D) as well as with metabolic characteristics that predispose to T1D. Two SNP (rs4588 and rs7041) located in *exon* 11 of the DBP gene (4q.13.3) seams to influence vitamin D metabolism. **Objective:** To associate the genotypic and allelic frequencies of rs4588 and rs7041 DBP polymorphisms in healthy subjects (controls) and T1D patients with diagnosis after 18 years of age, both Euro-Brazilians.

**Methods:** The study was approved by the Research Ethics Committee of the UFPR Health Sciences Sector (CAAE 01038112.0.0000.0102). The T1D patients (n = 135) and controls (n = 145) classified using ADA (2019) criteria were genotyped using PCR- RFLP.

**Results:** The polymorphisms analyzed are in Hardy–Weinberg equilibrium. The DBP rs4588 was not associated to T1D. Allele frequencies between the groups, as well as the dominant model, showed significance (P = 0.029) for rs7041. Carriers of the C allele (CC + CA) in rs4588 polymorphism were associated with a significant increase (P = 0.027) in HDL-cholesterol levels in the T1D group. The genotype (GG + GT) of rs7041 polymorphism was associated to a decrease in the concentrations of 1,5 anhydroglucitol (1,5 AG), only in the healthy subjects.

**Conclusion:** The presence of the G allele of rs7041 confer protection and the T allele risk to T1D the disease in the study population. It was found association between the C allele in rs4588 with a significant increase in HDL-cholesterol levels in the T1D group and G allele in rs7041 with decrease 1,5 AG levels in the controls.

**Financial support:** CAPES; CNPq; PPG-Ciências Farmacêuticas/UFPR; Fundação Araucária.

### P32 Association of glucose intolerance with markers of endothelial dysfunction assessed by flow-mediated vasodilation

#### Fernanda Yuri Yuamoto, Klaus Werner Wende, Stella Maris Firmino, Alessandro Domingues Heubel, Erika Zavaglia Kabbach, Renata Gonçalves Mendes, Ângela Merice de Oliveira Leal, Meliza Goi Roscani

##### Universidade Federal de São Carlos, São Carlos, Brazil

*Diabetology & Metabolic Syndrome* 2019, **11(Suppl 1):**P32

**Introduction:** It is already known that DM-2 patients present suggestive signs of endothelial dysfunction, like the alteration of flow-mediated vasodilation (FMD). However, it is still poorly studied if individuals with glucose intolerance may already present alterations in FMD as an early marker of incipient endothelial dysfunction.

**Objective:** To investigate the presence of early markers of endothelial dysfunction manifested by altered FMD in individuals with impaired glucose tolerance (IGT).

**Methods:** Cross-sectional clinical study with 2 groups composition: IGT Group: that met glucose intolerance criteria according to the American Diabetes Association (ADA) and Normoglycemic Control Group (NGC): without glucose intolerance or diabetes. Patients of both group underwent clinical evaluation, anthropometric measurements, transthoracic echocardiography, carotid ultrasound, assessment of the degree of physical inactivity by the IPAQ score, SF-36 quality of life score, and FMD. The comparison between the groups was performed by Chi square test for categorical variables and t-test or Man-Whitney test for continuous variables. This study was approved by Brazilian Ethics Committee.

**Results:** The groups were homogeneous regarding baseline and clinical variables (N = 41). It was observed that the IGT group (N = 8) had a higher prevalence of obesity (p = 0.02) and physical inactivity (p = 0.003) and better quality of life in the limitation due to emotional aspects (p = 0.001). The percentage of FMD was higher in the NGC (N = 33) and lower in the IGT group after 2 min (p = 0.02).

**Conclusion:** Individuals with IGT have initial markers of endothelial dysfunction assessed by the lowest FMD percentage, in addition to a higher degree of physical inactivity and impaired quality of life. Financial Support: CNPQ/PIBIC.

### P33 Association of interleukin-17A/RA polymorphism in gestational diabetes mellitus

#### Antonio Carlos Queiroz de Aquino^1^, Cecília Rodrigues Lucas^1^, Kleyton Thiago Costa de Carvalho^1^, Emanuelle Bernandes de Oliveira^1^, Alícia Gabriela Gomes Silva^1^, Ronier de Oliveira^1^, Maria da Conceição de Mesquita Cornetta^2^, Janaína Cristiana de Oliveira Crispim Freitas^1^

##### ^1^Departament of Clinical and Toxicological Analyses, Federal University of Rio Grande do Norte, Natal, Brazil; ^2^Departament ofObstetrics&Gynaecology, Federal Universityof Rio Grande do Norte, Natal, Brazil

*Diabetology & Metabolic Syndrome* 2019, **11(Suppl 1):**P33

**Introduction:** Gestational Diabetes Mellitus (GDM) is a metabolic disorder with onset or usually found during the pregnancy and it is defined as hyperglycemia that does not characterize any type of preexisting diabetes. During the gestational period the immune system of pregnant women undergoes lots of adaptations to guarantee fetal development. To ensure a physiologically healthy gestation the balance between the Th1 and Th2 profile is of great importance. Recent studies have pointed to the participation of the cytokine IL-17 in the status of a healthy pregnancy, and its unbalance in the uterine microenvironment may imply unfavorable outcomes.

**Objective:** The study aims to associate single nucleotide polymorphisms (SNP) of IL-17A (rs2275913) and IL-17RA (rs4819554) genes and susceptibility to GDM.

**Methods:** A case–control study has been conducted. Total of 40 GDM patients and 40 healthy controls and were selected. SNP typing was performed on two groups (IL17A and IL17RA) polymorphisms by polymerase chain reaction (PCR) to detect genotype and allele frequencies. The project was approved by the Research Ethics Committee (protocol number: 2.631.092 on May 2, 2018, CAAE: 73305717.2.0000.5292).

**Results:** There were no statistically significant associations between IL-17A*rs2275913 (G/A) and IL-17RA*rs4819554 (A/G) polymorphisms and DMG susceptibility in our sample (P > 0.05). Nevertheless, IL-17A*rs2275913 ((AA/AG)) was associated with increased risk of DMG, with OR = 3.462 (95% CI = 0.3196–37.50), P  =  0.5996).

**Conclusions:** Preliminary, this case–control study of IL17A/RA SNPs in patients with DMG is showing that the IL17A/RA gene polymorphisms were not associated with the development of DMG. However, its a pilot study and these findings should be confirmed with a larger number of samples.

**Financial support:** Capes, CNPQ.

### P34 Association of low birth weight with beta cell and kidney functions in adults without diabetes

#### Julia Inês Fleitas Branda^1^, Bianca de Almeida Pititto^2^, Sandra Roberta Gouveia Ferreira^3^

##### ^1^School of Public Health, University of São Paulo, São Paulo, Brazil; ^2^Department of Preventive Medicine, Federal University of São Paulo, São Paulo, Brazil; ^3^Department of Epidemiology, School of Public Health, University of São Paulo, São Paulo, Brazil

*Diabetology & Metabolic Syndrome* 2019, **11(Suppl 1):**P34

**Introduction:** Low birth weight (LBW) is considered indicative of adverse in utero conditions during critical period of development. Epigenetic changes in the fetus have been associated with increased risk of some chronic diseases in adulthood. Associations of LBW with reduced beta cell and kidney functions in the Brazilian population were less investigated. We examined the association of birth weight in a large sample of non-diabetic adults from the ELSA-Brasil.

**Methods:** A cross-sectional analysis of 819 non-diabetic ELSA-Brasil participants who had birth weight self-reported was performed. LBW (< 2.5 kg) was analyzed as a categorical variable; characteristics of LBW neonates were compared with normal birth weight ones (NBW ≥ 2.5 kg). In models of linear and logistic regression analysis, associations of LBW with parameters of beta cell function and insulin resistance (fasting glucose, A1c, HOMA-b, HOMA-IR, HOMA-adiponectin, TyG and QUICKI index), blood pressure (BP) and kidney function (albumin/creatinine ratio – ACR, glomerular filtration rate and serum cystatin C) were assessed. Variables were log-transformed for analyses.

**Results:** Mean age was 45.5 ± 4.8yrs and did not differ between NBW and LBW groups. The LBW had higher BP and ACR than the NBW group. After adjustments for age, sex and body mass index LBW was significantly associated with higher systolic BP levels or with the presence of hypertension (OR 0.77, 95%CI 1.05–4.25), and with ACR adjusted for age, sex, body mass index and blood pressure (p = 0.03). No significant association between LBW and parameters of beta cell function or insulin resistance was found.

**Conclusions:** Associations detected in our study reinforced the hypothesis that LBW could increase the risk of hypertension and kidney dysfunction later in life. However, our findings regarding beta cell function and insulin sensitivity were unable to support similar impact on the risk of glucose disturbances. We suggest that more accurate parameters should be explored to deepen such investigation.

### P35 Association of pre-diabetes with incipient markers of cardiovascular injury

#### Stella Maris Firmino, Klaus Werner Wende, João Paulo Gregório, Alessandro Domingues Heubel, Erika Zavaglia Kabbach, Renata Gonçalves Mendes, Angela Mérice de Oliveira Leal, Meliza Goi Roscani

##### Universidade Federal de São Carlos- Ufscar, São Carlos, Brazil

*Diabetology & Metabolic Syndrome* 2019, **11(Suppl 1):**P35

**Introduction:** Diabetes Mellitus type 2 (DM-2) is responsible for about 90% of diabetes cases and its mortality is associated with micro and macrovascular complications. Individuals who are at risk for developing DM-2 are referred to as pre-diabetics or as impaired glucose tolerance (IGT). It is estimated that approximately 25% of individuals with pre-diabetes will develop DM-2 within three to 5 years. It is not clear in the literature the behavior of cardiovascular function of these patients. It is believed that there are early markers of cardiovascular and endothelial dysfunction in this population.

**Objectives:** To evaluate the presence of early markers of impairment in endothelial and cardiovascular function in pre-diabetic patients compared to normoglycemic individuals.

**Methods:** Cross-sectional clinical study composed of two groups: Pre-diabetics (PDM) - who met the criteria for PDM and are not taking hypoglycemic medication; and Normoglycemic Control (NGC): presented normal values of glycemia in all applied tests. Patients in both groups had no known cardiovascular disease and were matched for age, sex and body mass index. They underwent clinical and physical evaluation, echocardiography, assessment of carotid intima-media thickness (CIMT), arterial stiffness, quality of life questionnaire, and degree of physical inactivity. Statistical analysis: comparison of the groups by Chi square test for categorical variables and t-test for variables with normal distribution and Man-Whitney for variables with non-normal distribution. Associations between variables in the same group were assessed by linear regression or Pearson’s correlation test. This study was approved by Brazilian ethical Committee.

**Results:** The groups were homogeneous regarding baseline and clinical variables (N = 41). Greater association with dyslipidemia (p = 0.03) and sedentary lifestyle (p = 0.05) was observed in the PDM group (N = 15), as well as early signs of diastolic dysfunction due to impaired left ventricular relaxation (p = 0.003) and early signs of CIMT (p = 0.001) in the PDM group. Quality of life also showed greater impairment in this group (p = 0.03).

**Conclusion:** PDM individuals already have early markers of cardiovascular injury and impaired quality of life when compared to healthy individuals with similar age and gender.

**Financial support:** CNPQ/PIBIC, PPG-BIOTEC.

### P36 Beers-fick criteria: is it important in type 2 diabetes mellitus?

#### Ana Luísa Andrade De Oliveira^2^, Michele Karen Pereira Clementino^1^, Dyalle Costa E Silva^1^, Filipe Mota Freitas^1^, Lara Gomes Melo^1^, Matheus Evangelista Correia^1^, Priscila Soares Garcia^1^, Ana Mayra Andrade De Oliveira^1^

##### ^1^Universidade Estadual de Feira de Santana, Feira de Santana, Brazil; ^2^Pontifícia Universidade Católica

*Diabetology & Metabolic Syndrome* 2019, **11(Suppl 1):**P36

**Introduction and objective:** Type 2 Diabetes Mellitus (DM) is the most prevalent type of DM worldwide with insulin resistance in the background and age and obesity are significant risk factors. In this way, most cases of type 2 DM are component of Metabolic Syndrome, being associated with other diseases such as Sistemic Arterial Hypertension (SAH) and Dyslipidemia. In this context, this type of DM demands the use of polypharmacy for metabolic and comorbities control. However, the therapeutic plan to be adopted should be established according to the pharmacokinetic and pharmacodynamic of each drug, processes that are peculiar in the elderly due to changes in physiological functions, wich may decrease the efficacy and increase the adverse effects of these drugs. This study aims to evaluate the usefulness of the Beers-Fick Criteria in prescribing elderly patients with type 2 DM in polypharmacy in a tertiary health service.

**Methods:** This is a cross-sectional, quantitative and exploratory study, wich used data from medical records of patients from an outpatient clinic in Feira de Santana – BA. The study included all patients diagnosed with type 2 DM who were using more than one drug, older than 60 years and with scheduled from August 2018 to January 2019. For the analysis, the Beers-Fick Criteria was used.

**Results:** A total of 333 medical records were evaluated, of wich 123 met the criteria of the present study (80% were female; 68.53 ± 7 years). Besides DM, 51% had another disease, being SAH the most common (37%). 11.5% had DM complications, and retinopathy was the most frequent. About 68% used polypharmacy, and the average number of drugs used per person was 5.45 ± 2. Adopting the Beers-Fick Criteria, 19.5% used inappropriate drugs, wich were: amitriptyline (14 patients), glibenclamide (7 patients) and fluoxetine (3 patients). No patient was taking concomitant use of more than one inappropriate drug.

**Conclusion:** Thus it was found the use of drugs with potential for inadequacy in indiviuals with DM. This demonstrates the need for a more careful approach on the part of the prescriber, who must consider all conditions regarding the elderly patient, in order to achieve the therapeutic goals, since physiological changes characteristic of advanced age may impact on the functionality of the drug and in disease control.

### P37 Blood glucose predictive model after exercise session for physically active type 2 diabetics

#### Rodrigo dos Santos Rodrigues Alves, João Marcos D, Theo Victor Alves Soares do Rêgo, Ricardo Azevedo Cavalcanti Aragão, Maria Elizabeth Queiroz Holanda do Nascimento, Jonathan Nícolas dos Santos Ribeiro, Cláudio Barnabé dos Santos Cavalcanti, Denise Maria Martins Vancea

##### Universidade de Pernambuco, Recife, Brazil

*Diabetology & Metabolic Syndrome* 2019, **11(Suppl 1):**P37

**Introduction:** Physical exercise is part of diabetic treatment. Although beneficial, its hypoglycemic effect when combined with patient medications may potentiate hypoglycemia. Knowing in advance how much the blood glucose will drop as a result of physical exercise makes the prescription safer.

**Objective:** To develop a linear regression model to estimate blood glucose after physical training.

**Methods:** It was characterized as a development study. Four type 2 diabetics taking 850 mg or 500 mg Metformin participated in a supervised exercise program for diabetics linked to a public university in the Northeast. For the development of the analysis were considered the dose (DOSE) and time of medication use (DELTATEMP); BMI (IMC); pre (GLICEPRE) and post training (GLICEPOS) blood glucose; and the type of exercise performed (TTREINO). Strength training was performed twice a week with 02 sets of 15 to 20 repetitions with 60” rest, consisting of eight exercises for the main muscle groups until the “concentric failure”. The aerobic training was performed once a week for about 20′ of dance practice. For the development of the model a linear regression was performed and then the data were “normalized”. An interaction variable relating DOSE and DELTATEMP was used. Finally, only the set of variables that presented the best statistical result was used to compose the last analysis. It was adopted f ≤ 0.05 and the value of p ≤ 0.05.

**Results:** The analysis presented a value f = 0.00 and an r = 0.62. The set of independent variables in this sample presented the best statistical evidence for IMC (p = 0.00); GLICEPRE (p = 0.00); SEX (p = 0.00).

**Conclusion:** The regression model was effective for this sample because it found values close to blood glucose measurements of type 2 diabetics after a training session, so it can be used to more accurately prescribe physical exercise.

### P38 Blue diabetes November campaign: screening of undiagnosed diabetes mellitus in community pharmacies in Brazil

#### Cassyano Januário Correr^1^, Josélia Cintya Quintao Pena Frade^2^, Eliete Bachrany Pinheiro^2^, Wesley Magno Ferreira^2^, Janice Sepúlveda Reis^3^, Karla Melo^3^, Roberto Pantarolo^1^, Walter da Silva Jorge João^2^

##### ^1^Universidade Federal do Paraná, Curitiba, Brazil; ^2^Conselho Federal de Farmácia, Brasília, Brazil; ^3^Sociedade Brasileira de Diabetes, São Paulo, Brazil

*Diabetology & Metabolic Syndrome* 2019, **11(Suppl 1):**P38

**Introduction:** Brazil has high diabetes mellitus (DM) prevalence, reaching 8.7% of the population. It’s estimated that 6.6 million people in the country have not yet been diagnosed (IDF, 2017). Populational studies assessing the prevalence of high blood glucose levels in people with no previous diagnosis of DM are scarce.

**Objective:** To carry out the screening of participants without previous diagnosis of diabetes who present high glycemia or high risk for the development DM in Brazil.

**Methods:** A national cross-sectional study was conducted in November 2018, involving pharmacies from all over the country. People without previous diagnosis of DM, aged 20 to 79 years were invited to participate in the study. The capillary blood glucose was assessed and a validated questionnaire (Findrisc) was applied to the participants. Fasting glycemia ≥ 100 mg/dL or casual glycemia ≥ 140 mg/dL were considered high.

**Results:** A total of 977 community pharmacists from 345 cities participated in the study and 17,580 people were evaluated. The majority of encounters (78.2%) occurred in consulting rooms within retail pharmacies. The population consisted mainly of women (59.5%) and people aged < 45 years (47.9%). The prevalence of patients with high glycemia was 18.4% (95% CI: 17.9 - 19.0). The Brazilian region with the highest prevalence was the Midwest, with 24.6%, and the lowest prevalence was in the Southeast (16.5%). The majority of the participants was sedentary (68.3%) and 84.2% had no previous history of high glycemia. It was observed that 43% of the people did not eat vegetables or fruits in a daily basis and 30.8% are hypertensive. Regarding the family history of type 1 or 2 DM, 21.3% had grandparents, uncles or cousins and 36.7 had parents, siblings or children with DM diagnosis.

**Conclusions:** There is a high prevalence of people in Brazil with high levels of glycemia without diagnosis. This is the largest screening study of DM ever conducted in Brazil and its results are of great importance, since they can drive public health actions to this undiagnosed population.

**Keywords:** Diabetes; Prevalence; high glycemia.

### P39 Brazilian population at risk of developing diabetes in the next 10 years, according to Findrisc: results of blue diabetes November campaign 2018

#### Cassyano Januário Correr^1^, Wendel Coura-Vital^2^, Josélia Cintya Quintao Pena Frade^3^, Lúbia Guaima Nascimento^2^, Mônica Soares Amaral Lenzi^3^, José Vanilton de Almeida^3^, Hermelinda Cordeiro Pedrosa^4^, Walter da Silva Jorge João^3^

##### ^1^Universidade Federal do Paraná, Curitiba, Brazil; ^2^Universidade Federal de Ouro Preto, Ouro Preto, Brazil; ^3^Conselho Federal de Farmácia, Brasília, Brazil; ^4^Sociedade Brasileira de Diabetes, São Paulo, Brazil

*Diabetology & Metabolic Syndrome* 2019, **11(Suppl 1):**P39

**Introduction:** Population aging, urbanization and changes in lifestyle have increased the prevalence of chronic noncommunicable diseases, such as hypertension, diabetes mellitus (DM) and dyslipidemia. In this sense, it is important to estimate the number of people at risk of developing diabetes over the next 10 years.

**Objective:** To carry out the screening of participants without previous diagnosis of diabetes who present high risk for the development of DM in Brazil.

**Methods:** A national cross-sectional study was conducted in November 2018, involving pharmacies from all over the country. People without previous diagnosis of diabetes, aged 20 to 79 years, were invited to participate in the study. The capillary blood glucose was assessed and a validated questionnaire (Findrisc) was applied to the participants. Findrisc is a standardized tool that generates a score that estimates a person’s risk of developing diabetes in the next 10 years. The score may indicate a low risk (1 in 100 develops disease), mildly moderate risk (1 in 25), moderate risk (1 in 6), high risk (1 in 3) or very high risk (1 in 2).

**Results:** A total of 977 community pharmacists from 345 cities participated in the study and 17,580 people were evaluated. The majority of encounters (87.8%) occurred in consulting rooms within retail pharmacies. The population consisted mainly of women (59.5%) and people aged < 45 years (47.9%). According to Findrisc, 19.5% of the Brazilian population presented a high risk of developing diabetes in the next 10 years and 3.1% a very high risk. The Midwest and North regions present 21.1% of the population at high risk of developing diabetes. In addition, the Midwest region had the highest percentage of people at very high risk (3.5%), followed by the Northeast region (3.4%). On the other hand, populations of the South and Southeast regions had the highest percentage (22.7%) of people with low risk of developing diabetes in the next 10 years.

**Conclusions:** There is a high percentage of the Brazilian population at risk of developing diabetes in the next 10 years and this risk is higher in the Midwest and North regions. Actions are necessary in the coming years in order to avoid this situation.

### P40 Brazilian type 1 & 2 diabetes disease registry (binder): a snapshot of diabetes mellituS (DM) in Brazil

#### Antônio Roberto Chacra^1^, Rodrigo de Oliveira Moreira^2^, Maurício Aguiar de Paula^3^, Karla Fabiana S. Melo Cabral Fagundes^4^

##### ^1^Universidade Federal de São Paulo (UNIFESP), São Paulo, Brazil; ^2^Instituto Estadual de Diabetes e Endocrinologia do Rio de Janeiro (IEDE-RJ), Rio de Janeiro, Brazil; ^3^Sanofi, São Paulo, Brazil; ^4^Faculdade de Medicina da Universidade de São Paulo (FMUSP), São Paulo, Brazil

*Diabetology & Metabolic Syndrome* 2019, **11(Suppl 1):**P40

**Introduction and objectives:** BINDER is an ongoing observational study of DM. The aims are to describe the proportion of patients (pts) with target HbA1c (< 7.0% or an individualized target), as well as demographic characteristics and comorbidities, DM complications, hypoglycemic episodes, medication use, treatment discontinuations, and hospitalizations.

**Methods and results:** BINDER collects longitudinal medical data from the previous 6 months at each of five cross-sectional waves of collection. This is the analysis of the second wave, with data as of October 2017 from 250 sites in 43 Brazilian cities. Of 2296 pts included, 2111 (91.9%) had type 2 DM (T2D) and 185 (8.1%) had T1D. Overall, 56.2% of pts were female, the mean age was 60.9 ± 14.5 years, and the mean time since diagnosis was 11.5 ± 10.3 years. T1D pts were more often (56.6%) in the public sector, while 68.2% of T2D pts were in the private sector. The overall proportions of pts seen by endocrinologists, general practitioners and cardiologists were 63.2%, 30.6% and 6.3%, respectively, but there was heterogeneity across the country. The most common treatments for T2D were metformin (78.4%), sulfonylureas (31.4%), and insulin (28.3%, 69.6% of which NPH). Most T1D patients were on long-acting insulin analogues (52.2% vs 28.6% on NPH). Macrovascular complications were reported for 44.5% of pts with T2D and 8.3% of those with T1D. Conversely, microvascular complications were more frequent among T1D pts (58.8% vs 29.4%). The reported overall hospitalization rate was 2% (5.6% in T1D vs 1.7% in T2D). The mean overall number of reported visits with other specialists was 2.8 ± 1.3 per year. Only 706 pts (44.2%) had HbA1c below 7.0%, and this was more frequent in the private (49.2%) than in the public (32.8%) sector (p < 0.001) and among pts with T2D (46.7%) than T1D (18.8%). Any hypoglycemia event in the previous month was more frequently reported in T1D than T2D (35.8% vs 3.2%), with nocturnal events reported for 11.1% vs 1.4% of pts, respectively. Pts with hypoglycemia were less likely to reach the HbA1c target (21.0%) than those without hypoglycemia (46.1%, p < 0.001). The main reasons for drug withdrawal were lack of efficacy and hypoglycemia risk.

**Conclusions:** More than half of pts with DM in Brazil did not reach HbA1c targets, with only a third achieving targets in the public system. Hypoglycemia appears to be associated with lack of glycemic control and adherence to treatment. Study sponsored by Sanofi.

### P41 Brazilian type 1 & 2 diabetes disease registry (binder): a snapshot of the type 1 diabetes (T1D) scenario in Brazil

#### Karla Fabiana S Melo Cabral Fagundes^1^, Antonio Roberto Chacra^2^, Maurício Aguiar de Paula^3^, Tiago Bonfim Catanho da Silva^3^, Rodrigo de Oliveira Moreira^4^

##### ^1^Faculdade de Medicina da Universidade de São Paulo (FMUSP), São Paulo, Brazil; ^2^Universidade Federal do Estado de São Paulo (UNIFESP), São Paulo, Brazil; ^3^Sanofi, São Paulo, Brazil; ^4^Instituto Estadual de Diabetes e Endocrinologia do Rio de Janeiro (IEDE-RJ), Rio de Janeiro, Brazil

*Diabetology & Metabolic Syndrome* 2019, **11(Suppl 1):**P41

**Introduction and objectives:** BINDER is an ongoing observational study of diabetes mellitus. This sub-analysis describes results from patients (pts) with T1D.

**Methods and results:** BINDER collects longitudinal medical data from the previous 6 months at each of five cross-sectional waves of collection from 250 sites in 43 Brazilian cities. This analysis of the second wave includes data as of October 2017 from 185 pts with T1DM assessed (8.1% of the total in BINDER), 112 of whom female (60.5%). The mean age was 34.2 ± 12.5 years, and the mean time since diagnosis was 15.2 ± 9.5 years. Most (90.8%) pts were managed by endocrinologists, 56.6% of pts were from the public sector, and the mean number of visits with another specialist was 3.1 ± 1.7 per year. The most commonly prescribed types of insulin were long-acting insulin analogues (62.6%), fast-acting analogues (59.3%), NPH (25.8%), and regular insulin (15.4%). Monotherapy with NPH or long-acting insulin analogues was reported for 25% of pts. Self-monitoring of blood glucose (SMBG) was reported with a mean of 4.2 ± 2.1 times per day, but only 49.2% of pts reported performing SMBG more than once daily. In addition to insulin, 27.2% of pts were using some oral agent: 17.9% metformin, 4.9% SGLT2 inhibitor, 2.2% acarbose, 1.6% DPP4 inhibitor, and 0.5% pioglitazone. Macrovascular and microvascular complications were reported for 8.3% and 58.8% of pts, respectively. Only 27 patients (18.8%; 95% CI 12.4–25.1%) had an HbA1c < 7.0%. The private sector had numerically more pts achieving HbA1c target (22.9%) than the public sector (13.4%, p = 0.19). Any hypoglycemia in the previous month was reported for 87.6% of pts (35.8% of events symptomatic, 11.1% nocturnal, 8% severe, and 17.3%/15.4% documented as < 70 mg/dL/< 54 mg/dL). The mean number of nocturnal events in the previous 6 months was 3.1 ± 1.8 per pt. Pts with hypoglycemia in the previous month were numerically less likely to achieve HbA1c target (16.3%) than those with no hypoglycemia (19.0%, p = 0.81).

**Conclusions:** Approximately 80% of pts with T1D in Brazil (majority treated in the public system) did not reach HbA1c targets, despite the large proportion of pts using insulin analogues. Hypoglycemia is an important barrier to achieving HbA1c targets, but suboptimal use of insulins and SMBG could also underlie these results. The study was sponsored by Sanofi.

### P42 Brazilian type 1 & 2 diabetes disease registry (binder): a snapshot of the type 2 diabetes (T2D) landscape in Brazil

#### Rodrigo de Oliveira Moreira^1^, Karla Fabiana S. Melo Cabral Fagundes^2^, Maurício Aguiar de Paula^3^, Daniele Pereira Jardim^3^, Antonio Roberto Chacra^4^

##### ^1^Instituto Estadual de Diabetes e Endocrinologia do Rio de Janeiro (IEDE - RJ); ^2^Faculdade de Medicina da Universidade de São Paulo (FMUSP); ^3^Sanofi; ^4^Universidade Federal de São Paulo (UNIFESP)

*Diabetology & Metabolic Syndrome* 2019, **11(Suppl 1):**P42

**Introduction and objectives:** BINDER is an ongoing observational study of diabetes mellitus. This sub-analysis describes results from patients (pts) with T2D.

**Methods and results:** BINDER is planned to collect longitudinal medical data from the previous 6 months at five cross-sectional waves of collection from 250 sites in 43 Brazilian cities. This analysis of the second wave includes data as of October 2017 from 2111 pts with T2D assessed (91.9% of the total in BINDER), 55.8% of whom female. The mean age was 63.2 ± 12.1 years, and the mean time since diagnosis was 11.1 ± 10.3 years. Pts were more often (68.2%) in the private sector, and the proportions of pts seen by endocrinologists, general practitioners and cardiologists were 60.7%, 32.7% and 6.6%, respectively, with some heterogeneity across the country. The mean number of visits with another specialist was 2.7 ± 1.2 per year. Only 46.7% of pts had an HbA1c < 7.0%. The private sector had more patients achieving HbA1c target (51.0%) than the public sector (36.4%, p < 0.001). Of pts using oral antidiabetic agents, 9.1% had HbA1c ≥ 9%; of these, 55.7% did not receive any change in the prescription, and among those who received a new prescription, 23.1% had an insulin indication. Hypoglycemia in the previous month was reported for 5.9% of pts, with mean numbers of nocturnal events of 1.7 ± 1.1, symptomatic events (≤ 54 mg/dL) of 2.7 ± 1.9, and severe events of 1.8 ± 1.3. Pts with hypoglycemia in the previous month were less likely to achieve HbA1c target (25.5%) than those with no hypoglycemia (47.8%, p = 0.0016). Of the 178 patients with a medication withdrawal reported, 42 patients (23.6%) had it because of lack of efficacy and 26 (14.6%) because of hypoglycemia risk.

**Conclusions:** Approximately 50% of pts with T2D in Brazil did not reach HbA1c targets, with only 36% of patients achieving goals in the public system. Hypoglycemia appears to be associated with lack of glycemic control and adherence to treatment. The study was sponsored by Sanofi.

### P43 Brazilian version of “insulin delivery system rating questionnaire”: translation and cultural adapta

#### Raquel Cristina Lopes Assis Coelho^1^, Adriana Silvino Pagano^2^, Aleida Nazareth Soares^1^, Janice Sepúlveda Reis^1^

##### ^1^Instituto de Ensino e Pesquisa da Santa CAsa de Belo Horizonte, Belo Horizonte, Brazil; ^2^faculdade de Letras, Universidade Federal de Minas Gerais, Belo Horizonte, Brazil

*Diabetology & Metabolic Syndrome* 2019, **11(Suppl 1):**P43

**Introduction:** The Insulin Delivery System Rating Questionnaire (IDSRQ) is a measure of health-related quality of life (HRQOL) and treatment preference for insulin delivery systems in persons with type 1 (T1DM) and type 2 diabetes mellitus (T2DM). The aim of the study was to translate and cross-culturally adapt the IDSRQ for Brazilian users as well as evaluate the validation of selected psychometric aspects.

**Materials and methods:** methodological study carried out in the following stages: forward translation, synthesis, back-translation, assessment by Judge Committee, pre-test and validation. International guidelines for translation and cross-cultural adaptation of measurement tools were followed. The validation provided information about the reliability (internal consistency, test–retest) and the construct validity of the studied tool.

**Results:** Regarding content validation, the instrument performed well in the Judges’ assessment with a mean Content Validity Index of 0.87 (± 0.2). Pre-test step involved 30 T1DM in face to face discussions. The IDSRQ validation study involved 113 T1DM patients, 46% male, mean age 32.61 (± 12.59) years and mean age at diagnosis of diabetes of 17.51 (± 12.41). 27.4% were using vial and syringe; 61.1% using pen and 11.5% using insulin pump. 76.5% of the patients administer insulin > 5 times daily. NPH was the most used basal insulin (39.8%), followed by glargine U100 (34.5%). Lispro was the most used fast insulin, by 60.2% of the patients. The scale presented acceptable internal consistency (Cronbach’s alpha = 0.785).

**Conclusions:** The translated and cross-culturally adapted Brazilian Portuguese version may be used to assess HRQoL and treatment preferences for insulin delivery systems in T1DM Brazilian patients.

**Keywords:** Diabetes mellitus; Type 1; Quality of life; Insulin

### P44 Breastfeeding during the first year and related factors among women with gestational diabetes: the Linda Brasil-Study

#### Kadhija Abrahim Cherubini^1^, Maria Inês Schmidt^1^, Leony Morgana Galliano^1^, Juliana Silvani^1^, Patrícia Dualib^2^, Adriana Costa e Forti^3^, Michele Drehmer^1^

##### ^1^Universidade Federal do Rio Grande do Sul, Porto Alegre, Brazil; ^2^Universidade Federal de São Paulo, São Paulo, Brazil; ^3^Universidade Federal do Ceará, Fortaleza, Brazil

*Diabetology & Metabolic Syndrome* 2019, **11(Suppl 1):**P44

**Introduction:** Breastfeeding may reduce the risk of developing type 2 diabetes in women with previous gestational diabetes (GDM). Few studies have evaluated breastfeeding (BF) practices and related factors in women with GDM, a group of women that may have more difficulties to breastfeed.

**Objective:** To evaluate breastfeeding and factors associated with weaning during the first year following a pregnancy complicated with GDM among women participating in the LINDA-Brazil Study.

**Methods:** Multicentric cohort of women with GDM (n = 2,220) enrolled at tertiary prenatal care centers from Porto Alegre, Pelotas and Fortaleza, followed by telephone interviews. Socio-demographics, behavioral, clinical and nutritional data were obtained at enrollment and breastfeeding practices were obtained by phone interviews. Time to weaning and related factors were estimated by Kaplan–Meier analyses and Cox regression models.

**Results:** Their average age was 31.4 ± 6.3 years; 50.2% reported being of non-white race/color, 32.0% having a familiar income within 1–2 minimum wages, 39.5% having completed high school, 89.2% living with a partner, 92.6% being non-smoker and 46.0% being obese before pregnancy. Average gestational age at delivery was 38.3 (± 1.6) weeks, 63.9% had cesarean section and the average birth weight was 3.286 (± 554) grams. The probability of breastfeeding at one-year postpartum was 53.4%. White color/race (Hazard Ratio [HR] = 1.46, 95% CI [1.21–1.76], p < 0.001), smoking in pregnancy (HR = 1.68, 95% CI[1.28–2.20], p < 0.001), difficulties to breastfeed (HR = 1.49, 95% CI[1.22–1.88]; p < 0.001), milk or formula offered before sixth months (HR = 2.54, 95% CI[2.09–3.08], p < 0.001), living in Porto Alegre (HR = 1.58, 95% CI[1.16–2.16] p < 0.001) and Pelotas (HR = 1.76, 95% CI [1.20–2.58; p < 0.001) were associated with a higher risk of weaning at 1-year. Maternal age between 30 and 39 years was associated with lower risk of weaning at 1-year compared to younger women (HR = 0.73, 95% CI [0.63–0.90], p < 0.001).

**Conclusions:** At 1 year after delivery, about half of the women with GDM participating in the LINDA-Brazil Study were breastfeeding. White color, smoking during pregnancy, early introduction of cow milk or formula, and being less than 30 years, all favored weaning. Although results are encouraging, there is room for further improvement in guiding women with gestational diabetes.

### P45 Can TSH values influence body composition and risk of atherosclerosis in type 1 diabetes?

#### Anna Theresa de Alencastro Correa, Gabriel Maia Ribeiro dos Reis, Gil Fernando da Costa Mendes de Salles, Lenita Zajdenverg, Joana Rodrigues Dantas Vezzani, Patrícia de Fátima dos Santos Teixeira, Melanie Rodacki

##### Universidade Federal do Rio de Janeiro –UFRJ, Rio de Janeiro, Brazil

*Diabetology & Metabolic Syndrome* 2019, **11(Suppl 1):**P45

**Introdução:** Patients with type 1 diabetes (T1D) are a population at high cardiovascular risk and they have an increased risk of association with thyroid autoimmune disease. Hypothyroidism, even subclinical (HS), may influence body composition mainly due to the increase in adipogenesis influenced by higher levels of thyroid stimulating hormone (TSH). The positive association between atherosclerosis and subclinical hypothyroidism has already been demonstrated in studies using pulse wave velocity (PWV) evaluation.

**Objective:** To evaluate the association between TSH plasmatic levels and both body composition and atherosclerosis.

**Methods:** This is an observational, cross-sectional study conducted between March 2018 and May 2019. TSH values were obtained by medical records. Body composition was assessed by bioimpedance examination and evaluation of atherosclerosis by brachial ankle index (ABI). The ABI was calculated by the quotient of the lowest of the systolic blood pressure in each leg and the highest systolic blood pressure between the two arms. ABI values below 0.91 were considered strong predictors of atherosclerotic disease.

**Results:** 38 patients with T1D were evaluated. Their mean age and disease duration were 24  ± 31 years and 17  ± 6.72 years, respectively. The body fat percentage (BFP) was elevated in 56.1% of the patients, the waist-to-hip ratio (WHR) was elevated in 34.1% of the patients, and the basal metabolic rate (BMR) was reduced in 47.5% of the patients. There was no significant relationship among TSH values and BFP (P: 0.506), WHR (P: 0.267) and BMR (P: 0.401). ABI values were higher in those with TSH below 2.5 mU/l (1.048 ± .74) than others (0.9967 ± .86); P: 0.039.

**Conclusion**: TSH levels were not associated with body composition in patients with T1D. In this sample, individuals with TSH levels 2.5 mU/l had lower ABI, suggesting that they could be more prone to development of atherosclerosis. The study did not receive financial support, funds were used by the institution itself.

### P46 Cardiometabolic profile and response to clinical treatment according to onset of obesity in infancy and adolescence or adulthood

#### Marta Germano Prado^1^, Glaucia Carneiro^1^, Maria Teresa Zanella^1^, Bianca De Almeida Pititto^2^

##### ^1^Disciplina de Endocrinologia, Escola Paulista de Medicina, UNIFESP, São Paulo, Brazil; ^2^Departamento de Medicina Preventiva, Escola Paulista de Medicina, UNIFESP, São Paulo, Brazil

*Diabetology & Metabolic Syndrome* 2019, **11(Suppl 1):**P46

**Introduction:** Enhancing knowledge about factors that could influence the response of obesity to clinical treatment is desirable in order to improve the control of obesity.

**Objective:** To evaluate the relationship between the period of the onset of obesity in the cycle of life with cardiometabolic profile and response to clinical treatment in obese (body mass index, BMI ≥ 30 kg/m^2^) adults followed in an Obesity Outpatient Clinic.

**Methods:** This cross-sectional study enrolled 105 individuals, > 17 years old, both sex, who were stratified according to the period of life when the obesity begun: infancy/adolescence (group1); adulthood (group2). Clinical characteristics and weight loss response to clinical treatment were compared between groups by Student t test or Mann–Whitney and Chi square tests.

**Results:** Among 105 participants, 30% were from group1 and 70% from group2. Group 1 was younger at the beginning of obesity [12.0 (4.2) vs. 31.5 (10.5) years, p < 0.001] and at the time of the first visit [34.9 (9.6) vs. 47.7 (10.7) years, p = 0.02], but the groups did not differ regarding duration of obesity [20.4 (9.8) vs. 16.4 (9.6) years, p = 0.085]. Group 1 had higher body weight at the beginning of the treatment [125.9 (29.0) vs. 109.6 (17.7) kg, p = 0.01] and BMI [45.6 (7.6) vs. 39.6 (7.9) kg/m^2^, p = 0.001] than group 2, but there were no statistical significant differences in mean of blood pressure, plasma glucose and lipids concentrations or in frequencies of hypertension [group1 = 80% and group2 = 86%], diabetes [group1 = 23% and group2 = 38%], pre-diabetes [group1 = 33% and group2 = 28%] or dyslipidemia [group1 = 73% and group2 = 70%]. Both groups were similar regarding body weight loss during the period of treatment [− 2.2 (− 6.4 to 3.5) vs. − 2.6 (− 7.9 to 1.0) kg, p = 0.98]; important to notice that there were no differences in duration of follow-up [17 (13 to 32) vs. 18 (15 to 33) months, p = 0.76], use of medication (100%), psychological and nutritional assistance and level of physical activity.

**Conclusion:** Although individuals who started obesity in infancy/adolescence were younger at the time of treatment, they did not differ regarding cardiometabolic profile and weight loss compared to those who developed obesity during adulthood. The similar duration of exposure to obesity might has a role for the development of morbidities and response to treatment, but these results could also mean that obesity started in youngest people is more severe and more resistant to treatment.

**Financial support:** CAPES.

### P47 Cardiovascular risk in individuals with congenital generalized lipodystrophy through calcium coronary score

#### Bartira Miridan Xavier Cortez Rodrigues Rebouças Feijó, Roberto Moreno Mendonça, Eryvaldo Socrates Tabosa Egito, Josivan Gomes de Lima, Maria de Fátima Paiva Baracho

##### Universidade Federal do Rio Grande do Norte, Natal, Brazil

*Diabetology & Metabolic Syndrome* 2019, **11(Suppl 1):**P47

**Introduction:** Congenital generalized lipodystrophy (CGL) is a rare disease characterized by a defect in the triglycerides deposition in subcutaneous cellular tissue, causing insulin resistance. The literature describes several indications of atherosclerotic disease in this population, such as premature cardiac death, myocardial fibrosis and coronary vessel atherosclerosis. The calcium coronary score (CCS) is currently the best marker of subclinical atherosclerotic disease.

**Objectives:** To assess cardiovascular risk in individuals with CGL through CCS.

**Methods:** Patients were selected for regular outpatient follow-up by CGL and with no cardiovascular disease known. We report here the results of the first 13 patients. General clinical evaluation and laboratory evaluation were performed. The patients went through chest tomography under standardized protocol for CCS evaluation. The data was described in absolute values as there are no percentiles for the sample age range.

**Results:** The sample consists on 09 women and 04 men, ranging in age from 6 to 37 years, average 18.5 (± 9.7) years old, which six of the cases were under 11 years old, waist 73.71 cm (± 12.79) and body mass index (BMI) 19.17 (± 4.01). Three of them are hypertensive and four are diabetic. Laboratory data expressed on average (± standard deviation) were: glycated hemoglobin 6.2% (± 1.7), total cholesterol 171 mg/dl (± 102), LDL 85.7 mg/dl (± 23.4), triglycerides 170.25 mg/dl (± 127), HDL 31.07 mg/dl (± 8.85), GOT 28.84 mg/dl (± 8.9), GPT 46.41 mg/dl (± 34) and GGT 60.4 mg/dl (± 78.03). CCS was non-zero in three individuals (23.07%) of the population studied so far, with absolute values of 1.94, 0.77 and 294. These individuals are 20, 28 and 37 years old respectively and are hypertensive, being case with higher CCS also diabetic.

**Conclusion:** The study suggests that individuals with CGL have a higher prevalence of subclinical atherosclerotic disease under 45 years old than the general population, with consequently a probable increase in cardiovascular risk and death from all causes.

### P48 Characterization of women with gestational diabetes mellitus: using application attenuated total reflection Fourier transform infrared (ATR-FTIR) spectroscopy

#### Emanuelly Bernardes-Oliveira^1^, Daniel Lucas Dantas^2^, Emanuelly Marques Cardoso^2^, Antonio Carlos Queiroz-Aquino^3^, Kleyton Thiago Costa Carvalho^3^, Maria da Conceição de Mesquita Cornetta^4^, Kassio Michell Gomes Lima^2^, Janaina Cristiana de Oliveira Crispim^4^

##### ^1^Programa de Pós-Graduação em Desenvolvimento e Inovação Tecnológica em Medicamentos, UFRN, Natal, Brazil; ^2^Laboratório de Química Biológica e Quimiometria, Instituto de Química, UFRN, Natal, Brazil; ^3^Departamento de Análises Clínicas e Toxicológicas, UFRN, Natal, Brazil; ^4^Maternidade Escola Januário Cicco, UFRN, Natal, Brazil

*Diabetology & Metabolic Syndrome* 2019, **11(Suppl 1):**P48

**Introduction:** Gestational Diabetes Mellitus (GDM) is a metabolic imbalance that occurs in some women during gestation, leading the body to produce insulin resistance during gestations GDM affect around 5–15% of pregnancies and it considered a public health problem. Until now it isn’t known what promotes this metabolic profile in these women, therefore the importance of studying new methodologies to characterize them.

**Objective:** Propose an innovative methodology to characterize GDM using Attenuated Total Reflectance Fourier Transform Infrared (ATR-FTIR) biospectroscopy together with the Principal Component Analysis (PCA), technique constructing an unsupervised classification model for GDM and healthy (Control) groups.

**Methods:** The study was carried out on 100 pregnant females attending obstetrics and gynecology ambulatory Tertiary Hospital (Brazil). They were divided into 2 groups: GDM (n  =  50) pregnant females diagnosed with gestational diabetes and healthy group (n  =  50) pregnant females were not suffering from any glucose intolerance (control group), both groups were between 12 and 38 weeks of gestation. The study was approved by the Ethics Committee of the Federal University of Rio Grande do Norte, Natal, Brazil (CAAE No.: 73305717.2.0000.5292). All patients signed they the Informed Consent Form, later 8 ml of peripheral blood was collected. The samples were centrifuged and the plasma separated for biospectroscopy analysis. Plasma 3 µL (in triplicate) was inserted into the ATR-FTIR device coupled to unsupervised multivariate analysis PCA to acquire the fingerprint of the 900–1800 cm^−1^ region of interest, the Baseline and Savitzky-Golay Smoothing correction.

**Results:** The visual results provided by the PCA mathematical algorithm demonstrate a clear separation between the DMG and Control analyzed groups. Such differences are identified in the spectra PC1, PC2 and PC3 loadings, whit cumulative total variance of 91.90%, PCs both were able differentiate quite efficiently the GDM and control group. Mainly PC3 versus PC2, obtaining in the first quadrant of analysis 7.16%–16.56% respectively, as observed in the 3D plane, which best selected the groups.

**Conclusions:** ATR-FTIR is an innovative technique that characterizes GDM patients quickly, without use of reagents, with less impact on the environment and reducing the cost associated with the traditional method. In addition, pregnant women are not required to expose themselves to fasting hours and ingest glucose load.

### P49 Chronic kidney disease (ckd) and risk of mortality, cardiovascular (CV) events and severe hypoglycemia in type 2 diabetes (T2D): devote results

#### Paulo Roberto Rizzo Genestreti^1^, Aslam Amod^2^, Scott S. Emerson^3^, Steven P. Marso^4^, Darren K. McGuire^5^, Thomas R. Pieber^6^, Rodica Pop-Busui^7^, Neil R. Poulter^8^

##### ^1^Instituto do Coração - Hospital das Clínicas - Universidade de São Paulo, São Paulo, Brazil; ^2^University of KwaZulu, Natal, South Africa; ^3^University of Washington, Washington, USA; ^4^HCA Midwest Health, Overland Park, USA; ^5^University of Texas Southwestern Medical Center, Dallas, USA; ^6^Medical University of Graz, Graz, Austria; ^7^University of Michigan, Ann Arbor, USA; ^8^Imperial College London, London, UK

*Diabetology & Metabolic Syndrome* 2019, **11(Suppl 1):**P49

**Introduction:** T2D is associated with an increased risk of cardiovascular disease (CVD) and CKD. CKD is a known risk factor for major adverse cardiovascular events (MACE), all-cause mortality and hypoglycemia. This secondary, pooled analysis from DEVOTE examined whether baseline CKD stages were associated with an increased risk of MACE, all-cause mortality or severe hypoglycemia in T2D patients.

**Methods:** DEVOTE was a treat-to-target, randomized, double-blind trial in 7637 patients with T2D at high CV risk, treated once daily with insulin degludec or insulin glargine 100 units/mL. According to baseline CKD stages (CKD stage 2: n = 3118; stage 3: n = 2704; stages 4 + 5: n = 214), more patients had a history of CVD (CKD stages 3‒5), were older and had lower A1C vs those with normal kidney function (normal + CKD stage 1, n = 1486).

**Results:** Risk of MACE and all-cause mortality was significantly higher (*p *< 0.05) in those with a higher baseline CKD stage. There was a significantly higher rate of severe hypoglycemia for stages 3 and 4 + 5 vs stage 2 or normal + stage 1. There were no significant interactions between treatment and CKD stages. Comparisons between treatment groups by CKD stage mirrored those from the primary analyses.

**Conclusion:** Increasing severity of baseline CKD stages was associated with a higher risk of MACE, all-cause mortality and severe hypoglycemia in T2D patients at high CV risk

### P50 Clinical outcome assessment of the effectiveness of insulin degludec in real-life medical practice (CONFIRM): a comparative effectiveness study of degludec and insulin glargine 300 units/ml

#### Giulliana Parisi Rodrigues^1^, Joseph Tibaldi^2^, Steffen Haldrup^3^, Viktor Sandberg^3^, Michael Lyng Wolden^3^, Helena W. Rodbard^4^

##### ^1^Novo Nordisk Farmaceutica do BrasilLtda, São Paulo, Brazil; ^2^Fresh Meadows Diabetes and Endocrinology, New York, USA; ^3^Market Access, Novo Nordisk A/S, Søborg, Denmark; ^4^Clinical Research, Rockville, USA

*Diabetology & Metabolic Syndrome* 2019, **11(Suppl 1):**P50

**Introduction:** Insulin degludec (degludec) is a basal insulin with a duration of action > 42 h and a low day-to-day variability in blood glucose-lowering profile. Randomized controlled trials (RCTs) in adult patients with type 1 (T1D) or type 2 diabetes (T2D) have demonstrated that with equivalent glycemic control, degludec is associated with a reduced risk of hypoglycemia compared with other long-acting insulin analogs. Presently there are no fully disclosed RCT data comparing degludec with insulin glargine 300 units/mL (glargine U300).

**Objective:** The aim of the CONFIRM (Clinical Outcome assessmeNt of the eFfectiveness of Insulin degludec in Real-life Medical practice) study was to investigate the comparative effectiveness of degludec versus glargine U300 in insulin-naïve adult patients with T2D in routine US clinical practice.

**Methods:** CONFIRM is a non-interventional comparative effectiveness study following US patients through electronic medical records (EMRs) from the Explorys (IBM Watson Health™) database. EMRs from > 50 million patients were utilized. CONFIRM included insulin-naïve adult patients with T2D and inadequately controlled with oral antihyperglycemic drugs ± a glucagon-like peptide-1 receptor agonist at baseline. The primary endpoint was change in mean HbA1c from the date of basal insulin initiation until 180 days of follow-up. Secondary endpoints: change in rates of hypoglycemic episodes, change in proportion of patients with ≥ 1 episode of hypoglycemia, time-to discontinuation of initial basal insulin and end-of-study basal insulin dose.

**Results:** Change in HbA1c was significantly greater with degludec versus glargine U300. Rates of hypoglycemia were significantly lower with degludec vs glargine U300, and the proportion of patients experiencing hypoglycemia was significantly lower with degludec. Patients with glargine U300 had a 37% higher risk of treatment discontinuation vs degludec. Prescribed insulin doses were 9% lower with degludec vs glargine U300. Change in body weight was not significantly different in patients in both treatments.

**Conclusions:** In this study, we investigated the comparative effectiveness of degludec vs glargine U300. Results demonstrated significantly improved glycemic control at lower insulin doses and lower risk of discontinuation with degludec versus glargine U300. There was a significant reduction in both rates and proportions of patients experiencing hypoglycemia with degludec compared with glargine U300.

### P51 Clinical response (HBA1C ≥ 1% and/or body weight ≥ 5% reduction) to semaglutide by baseline HBA1C and body weight in the sustain program

#### Andre Luiz Bressan^1^, Victor França De Almeida^1^, Vanita R. Aroda^2^, Julie R. Larsen^3^, Signe Harring Østoft^3^, Rosangela R. Rea^4^, Danny Sugimoto^5^, Uwe Ziegler^3^

##### ^1^Novo Nordisk Framacêutica do Brasil, Araucária, Brazil; ^2^Brigham and Women’s Hospital, Boston, USA; ^3^Novo Nordisk A/S, Soborg, Denmark; ^4^Hospital de Clinicas da Universidade Federal do Parana, Curitiba, Brazil; ^5^Cedar Crosse Research Center, Chicago, USA

*Diabetology & Metabolic Syndrome* 2019, **11(Suppl 1):**P51

**Introduction:** The SUSTAIN clinical development program assessed semaglutide once weekly, a glucagon-like peptide-1 (GLP-1) analog, across the continuum of type 2 diabetes (T2D) care, including in drug-naïve subjects and those on a background of oral antidiabetic drugs and/or insulin. Semaglutide demonstrated superior reductions in HbA1C and body weight (BW) vs. placebo and comparators (sitagliptin, exenatide extended release, insulin glargine, dulaglutide).

**Objective:** This analysis assessed the proportion of semaglutide-treated subjects achieving clinically relevant responses (composite endpoint: ≥ 1% HbA1C reduction and ≥ 5% BW loss; individual components: ≥ 1% HbA1C or ≥ 5% BW loss) by baseline HbA1C and BW across SUSTAIN 1–5 and 7.

**Methods:** A post hoc analysis of Sustain 1–5 and 7.

**Results:** A consistently high proportion of semaglutide-treated subjects achieved clinically relevant responses (composite, 42.0%; ≥ 1% HbA1C reduction, 76.6%; ≥ 5% BW loss, 49.9%), regardless of baseline HbA1C and BW. Baseline HbA1C and BW did not affect the likelihood of achieving the composite and ≥ 5% BW endpoints. A ≥ 1% HbA1C reduction was significantly more likely to be achieved with higher baseline HbA1C (143% increased odds per 1% unit higher baseline HbA1C; p < 0.0001).

**Conclusion:** Semaglutide provides meaningful reductions in HbA1C and BW across a range of HbA1C and BW. Individuals with poorly controlled HbA1C were more likely to achieve a ≥ 1% HbA1C reduction.

### P52 Clinical response to semaglutide by baseline hba1c and body weight

#### Mariana Arruda Camara Ferreira da Silva

##### Novo Nordisk Brasil, Araucária, Brazil

*Diabetology & Metabolic Syndrome* 2019, **11(Suppl 1):**P52

**Introduction:** The SUSTAIN clinical development program assessed semaglutide once weekly, a glucagon-like peptide-1 (GLP-1) analog, across the continuum of type 2 diabetes (T2D) care, including in drug-naïve subjects and those on a background of oral antidiabetic drugs and/or insulin. Semaglutide demonstrated superior reductions in HbA1c and body weight (BW) vs. placebo and comparators (sitagliptin, exenatide extended release, insulin glargine, dulaglutide).

**Methods:** This post hoc analysis assessed the proportion of semaglutide-treated subjects achieving clinically relevant responses (composite endpoint: ≥ 1% HbA1c reduction and ≥ 5% BW loss; individual components: ≥ 1% HbA1c or ≥ 5% BW loss) by baseline HbA1c and BW across SUSTAIN 1–5 and 7.

**Results:** A consistently high proportion of semaglutide-treated subjects achieved clinically relevant responses (composite, 42.0%; ≥ 1% HbA1c reduction, 76.6%; ≥ 5% BW loss, 49.9%), regardless of baseline HbA1c and BW. Baseline HbA1c and BW did not affect the likelihood of achieving the composite and ≥ 5% BW endpoints. A ≥ 1% HbA1c reduction was signi_cantly more likely to be achieved with higher baseline HbA1c (143% increased odds per 1% unit higher baseline HbA1c; p < 0.0001).

**Conclusions:** Semaglutide provides meaningful reductions in HbA1c and BW across a range of HbA1c and BW. Individuals with poorly controlled HbA1c were more likely to achieve a ≥ 1% HbA1c reduction.

### P53 Compasso protocol in the promotion of self-care practices in type 2 diabetes mellitus: via telephone call

#### Jéssica Caroline dos Santos, Laura Barbosa Nunes, Heloísa de Carvalho Torres

##### Universidade Federal de Minas Gerais, Belo Horizonte, Brazil

*Diabetology & Metabolic Syndrome* 2019, **11(Suppl 1):**P53

**Introduction:** Diabetes Mellitus Type 2 (T2DM) is a chronic health condition associated with unhealthy behaviors. This requires the constant search for resources that assist the health professional in promoting self-care. The phone is considered one of these features. Considering this scenario, it is noted that there is already the instrument COMPASSO, validated protocol for telephone intervention that offers subsidies to the healthcare professional in relation to self-care practices.

**Objective:** To analyze the COMPASS protocol in the promotion of self-care practices in type 2 diabetes mellitus, via telephone call.

**Methods:** This is a descriptive study involving 253 people with T2DM treated at eight basic health units (BHU) in Belo Horizonte MG and participants of the program “Evaluation of the effectiveness of behavioral interventions aimed at diabetes self-care in Primary Care”. “Sociodemographic data were collected; glycated hemoglobin (reference value ≤ 6.5%); and the COMPASS protocol was applied. Data collection was by the esurv platform. Descriptive analysis was performed by calculating frequencies for categorical variables and measures of central tendency (mean and median), and dispersion (SD: standard deviation) for quantitative variables. All research ethics standards were met in accordance with National Health Council Resolution 466/12.

**Results:** An average age of 62 years was observed; 67.2% were female; 53.0% had a partner; 68.0% had even elementary school as a level of education; 45.5% were retired; and 71.9% had a monthly income of two minimum wages. It was found that the protocol is easy to apply and allows health professionals to monitor, plan and implement contextualized interventions to self-care practices. Telephone self-care analysis mitigates common structural and contact barriers faced by professionals, as well as by people with T2DM themselves. In addition, the application of COMPASS allowed to identify and work some barriers of self-care. During the application of the instrument, it was found that 40.2% had difficulty following the eating plan; 25.3% do physical exercise; 5.2% to make appointments; and 2.8% of taking the medications.

**Conclusions:** The COMPASSO protocol was viable for monitoring self-care practices via telephone call. Financial Support: CNPq 432824/2016; FAPEMIG APQ-03865-16.

### P54 Conditions that interfere with foot self-care in people with diabetes meliitus

#### Sônia Silva Alves, Angel Tamna Souza De Souza, Ellen Priscila Oliveira Passos, Francineide Pereira Da Silva Pena, José Luis Da Cunha Pena, Erika Tatiane De Almeida Fernandes Rodrigues, Adriane Stefanny Rocha Ribeiro, Jéssica Gomes Da Silva

##### Universidade Federal do Amapá- Unifap, Macapá, Brazil

*Diabetology & Metabolic Syndrome* 2019, **11(Suppl 1):**P54

**Introduction:** In the context of complications associated with Diabetes Mellitus (DM), the diabetic foot stands out, defined as a clinical situation in which lower limbs present ulcerations, deep tissue destruction and infections associated with diabetic neuropathy, induced by sustained hyperglycemia, with or without peripheral vascular disease. An estimated 50% to 75% of lower limb amputations are in people with diabetes and up to 50% of amputations are preventable. Ignorance of the diagnosis of DM contributes to increase the possibility of foot injury, and yet most people with DM are unaware of the diabetic foot event. Health education for these people is necessary so that they can learn and reflect on foot care.

**Objective:** To identify conditions that interfere with foot self-care in people with DM.

**Methods:** Study of qualitative approach, with the focus group technique - FG, having as location a basic health unit. Approved by the Ethics Committee of the Federal University of Amapá No. 2,430,811, CAAE: 80829617.8.0000.0003 9 people with DM participated in the Health Promotion Program for People with Diabetes Mellitus (PPSPDM). Guiding question (FG): What conditions can interfere with the performance of foot care? Data analysis was performed through thematic content analysis, elected due to the notion of theme, and is linked to a statement about a certain subject.

**Results:** As an analytical category: Conditions that interfere with foot care: it was built from the cataloging and coding of the FG speech, it was established 8 units of meaning, which were considered by the participants as the ones that most positively or negatively influence the achievement. foot care: forgetfulness (9), laziness (17), tiredness (4), haste (2), physical and motor difficulties (4), visual difficulties (3), factors related to foot aesthetics (3). After analysis, educational activities were performed as intervention strategies, offering clues on how to overcome the conditions evidenced in the units of analysis.

**Conclusions:** It can be seen that the conditions that influence full foot care are associated with the culture of lack of habit, that is, allowing time for effective foot care. The strategy by the FG methodology favored the intervention for learning, behaviors and changing attitudes for foot care of people with diabetes.

### P55 Conventional anthropometric indicators versus unconventional as cardiovascular risk predictors in diabetics

#### Synara Cavalcante Lopes^1^, Renan Magalhães Montenegro Júnior^1^, Francisca Diana da Silva Negreiros^1^, Lorena Taúsz Tavares Ramos^1^, Luís Felipe Viana Correia^2^, Nathalia Bernardo Marinho Leite^1^, Maria Yasmin Paz Teixeira^1^, Fábia Karine de Moura Lopes^1^

##### ^1^Universidade Federal do Ceará, Fortaleza, Brazil; ^2^Universidade de Fortaleza – UNIFOR, Fortaleza, Brazil

*Diabetology & Metabolic Syndrome* 2019, **11(Suppl 1):**P55

**Introduction:** Anthropometric indicators are used to measure central adiposity and contribute to the prediction of cardiovascular morbidity and mortality. The most commonly used anthropometric indicators to identify cardiovascular risk are Waist Circumference (WC) and Neck Circumference (NC). However, other less conventional measures may be used, such as Waist/Height Ratio (WHtR) and Conicity Index (CI).

**Objectives:** To compare the association between conventional and unconventional anthropometric indicators and the prevalence of cardiometabolic risk in diabetic patients.

**Methods:** Cross-sectional, quantitative and descriptive study. A total of 121 individuals of both sexes, treated at a reference endocrinology outpatient clinic in Fortaleza – CE, were included. WC was measured using cutoff points for cardiovascular risk ≥ 94 cm and ≥ 80 cm for men and women, respectively. For NC, values ​​ ≥ 37 cm were used for men and ≥ 34 cm for women. The cutoff point for the definition of central obesity was ≥ 0.5 for WHtR in both sexes. For the CI, the cutoff points ≥ 1.25 and ≥ 1.18 were adopted for men and women, respectively. The construction of the database was performed in Excel and statistical analysis was performed using the SPSS program, with significance p < 0.05. This study was accepted by the Research Ethics Committee of the Federal University of Ceará under no. 83521518.0.0000.5045.

**Results:** The mean age of the studied group was 53 ± 13.02 years. The mean BMI was 29.33 kg/m^2^, indicating overweight. According to WC, 85.8% and 87.3% of the individuals, respectively men and women, demonstrated risk for the development of cardiometabolic diseases. Regarding to unconventional indicators, WHtR identified 95.8% of the studied population at cardiovascular risk while CI detected 90.1%. The variable WC was positively associated with WHtR and CI (p < 0.001).

**Conclusions:** Cardiovascular risk analysis can also be measured by unconventional anthropometric indicators, which can be used for nutritional screening in clinical practice.

### P56 Conversation map: health education technology for the care of individuals with diabetes mellitus

#### Gemiliana Sombra de Oliveira Carvalho^1^, Francisca Diana da Silva Negreiros^1^, Silvana Linhares de Carvalho^1^, Tatiana Rebouças Moreira^1^, Samila Torquato Araújo^1^, Maria de Jesus Nascimento de Aquino^1^, Thereza Maria Magalhães Moreira^2^, Renan Magalhães Montenegro Júnior^1^

##### ^1^Universidade Federal do Ceará, Fortaleza, Brazil; ^2^Universidade Estadual do Ceará, Fortaleza, Brazil

*Diabetology & Metabolic Syndrome* 2019, **11(Suppl 1):**P56

**Introduction:** Alarmingly increasing prevalence rates of chronic diseases, such as diabetes mellitus (DM), have changed the epidemiological panorama worldwide. Health education is a strategy for healthcare system users with DM to better understand their condition, focused on health promotion. The Diabetes Conversation Map is an educational technology created and validated by the International Diabetes Federation which is based on playful and interactive illustrations.

**Objective:** To describe an intervention using an educational technology in health that consisted of a conversation map for the care of individuals with DM.

**Methods:** This is an experience report with 235 patients surveyed from August 2018 to March 2019 in a specialized outpatient clinic in Ceará state, Brazil. Diabetes education was supervised by a multiprofessional team and took place in groups with six to ten healthcare system users and their families. The activities were carried out in a reserved room prior to the medical appointment, once a week, with an approximate length of 45 min. Meeting minutes were taken and kept in the records of the healthcare facility. All ethical principles and guidelines concerning research with human beings were followed.

**Results:** The study maps generated the following topics: how the human body and diabetes work; healthy eating and physical activity; reaching goals with the use of insulin; understanding the various factors involved in diabetes control; and diabetes and foot care. Participants and facilitators formed a conversation circle, where the conversation map was exposed for viewing and discussion. During the interventions, there was greater involvement of participants with the topic “reaching goals with the use of insulin”. The exchange of experiences revealed lack of knowledge about the injection, transportation and conservation of insulin, type and amount of food intake, pathophysiology of the disease and the use of appropriate footwear. The time required to implement the maps can be considered a limitation of the tool, as the workshops were carried out before the participants’ medical appointments, which may have generated anxiety among them.

**Conclusions:** Educational practices provide a dynamic and empathic environment for the exchange of experiences, clarification of questions, and behavioral changes for diabetes control. The approach to healthy eating, insulin therapy and foot care should be strengthened to encourage self-care in this population.

### P57 Correlation between biomechanical changes, plantar pressure and balance of people with diabetic neuropathy

#### Juliana Vallim jorgetto, Mônica Antar Gamba, Denise Miyaki Kusahara

##### Universidade Federal de São Paulo- UNIFESP, São Paulo, Brazil

*Diabetology & Metabolic Syndrome* 2019, **11(Suppl 1):**P57

**Introduction:** Biomechanical changes, elevated plantar pressure points (PP) and imbalance lead to overload and loss of skin integrity in diabetic neuropathy (ND).

**Objective:** To evaluate the correlation between balance, biomechanical changes and plantar pressure in diabetic individuals.

**Methods:** Descriptive and cross-sectional study (11/2018 to 02/2019), with 84 individuals with ND. Inclusion criteria: individuals with DN, municipal public network, more than 5 years of diagnosis, both sexes and over 30 years. Variables were investigated: age, gender, time since diagnosis, fasting blood glucose values, glycated hemoglobin, biomechanical changes of the feet, balance and plantar pressure (BaroScan). The analysis was performed by descriptive and inferential statistics and significance level 0.05. The research was approved under opinion No. 2,695,704.

**Results:** Of the 84 individuals evaluated, 58 (69%) were women and 26 (31%) men, mean age 61.82 (± 13.94) years, mean diagnosis time 14 (± 9.71) years, blood glucose mean 159.62 (± 74) mg/dl and mean glycated hemoglobin 7.13 (± 0.94). The Berg Balance Scale showed a variation between 8 and 56 points (average 40.38 points - risk of falling). On physical examination of the feet, bone callosity was present in 61 (72.61%) in both, right 38 (45.24%) and left 38 (45.24%) bone prominence, claw toes, right foot 38 (45.24%) and left 42 (50%), hallux valgus right foot 32 (38.09%) and left foot 27 (32.14%). The mean PP on the right foot was 6.38 (± 1.61) kgf/cm^2^ (13.28 and 2.21) and left 6.08 (± 1.64) kgf/cm^2^ (10.93 and 1.64), and in the right foot 45 individuals presented PP peak above 6 kgf/cm^2^ and 41 in the left foot. No significant correlation was identified between balance, biomechanical foot changes and PP alone. Canonical correlation analysis with 2 sets of variables (right and left PP and Berg Scale versus number of right and left biomechanical changes) showed a statistically significant (0.507) moderate correlation (p = 0.002).

**Conclusions:** Bone callosity was more frequent and half of the evaluated individuals presented high plantar pressure peak. Bivariate analysis of the influence of plantar pressure on balance and biomechanical changes on balance was not statistically significant; however, when associated with plantar pressure and balance, a moderate correlation was found with the amount of biomechanical changes presented by individuals.

**Financial support:** (if applicable).

### P58 Correlation between handgrip strength and cognitive ability of elderly women with type 2 diabetes mellitus

#### Maria Elizabeth Queiroz Holanda do Nascimento^1^, Iago Vilela Dantas^2^, Gabriel Albuquerque Galvão^1^, Ricardo Azevedo Cavalcanti Aragão^1^, Nathália De Souza Melo Abath Barbosa^1^, Jonathan Nícolas Dos Santos Ribe^1^, Cláudio Barnabé Dos Santos Cavalcanti^1^, Denise Maria Martins Vancea^1^

##### ^1^Universidade de Pernambuco, Recife, Brazil; ^2^Universidade Federal de Pernambuco, Recife, Brazil

*Diabetology & Metabolic Syndrome* 2019, **11(Suppl 1):**P58

**Introduction:** Declines in strength and cognition levels are consequences of aging, which may be potentiated by type 2 diabetes. Decreases in strength patterns in the elderly population influence cognitive impairment, but the relation between these variables in the diabetic population is unknown.

**Objective:** To correlate handgrip strength with cognition levels of elderly women with type 2 diabetes mellitus.

**Methods:** This study was characterized as descriptive, cross-sectional and quantitative (Ethics Commitment: 34868714.4.0000.5207). Ten physically active elderly women with type 2 diabetes, with a mean age of 69.3 years ± 5.33 years, were included. Data were collected in a single meeting in the morning. To evaluate the handgrip strength, an electronic hand-held dynamometer was used. To assess cognition, the Mini Mental State Examination questionnaire was applied. Descriptive and inferential analyzes were performed using SPSS 21.0 for Windows software. For descriptive analysis, the mean, standard deviation and confidence interval were calculated. For analysis of the correlation between variables, the nonparametric Spearman test was used.

**Results:** From the data analysis, it was observed that there was no correlation (p ≤ 0.29/r = 0.31) between strength and cognition.

**Conclusion:** For elderly women with type 2 diabetes mellitus in this sample, strength and cognition were not correlated.

### P59 Correlation between waist and neck circumference in individuals with type 2 diabetes mellitus

#### Mariana Pimentel Gomes Souza, Nathália Bernardo Marinho Leite, Maria Yasmin Paz Teixeira, Fábia Karine de Moura Lopes, Lorena Taúsz Tavares Ramos, Francisca Diana da Silva Negreiros, Synara Cavalcante Lopes, Renan Magalhães Montenegro Júnior

##### Universidade Federal do Ceará, Fortaleza, Brazil

*Diabetology & Metabolic Syndrome* 2019, **11(Suppl 1):**P59

**Introduction:** Insulin resistance can be predicted by anthropometric measurements. Waist Circumference (WC) measurement is a fast and inexpensive method, but it has limitations, such as the need to remove clothes, supine position and the difficulty of measurement in obese individuals. Thus, Neck Circumference (NC) may be a less invasive and more practical alternative.

**Objective:** To investigate the correlation between WC and NC as methods for predicting insulin resistance in individuals with type 2 diabetes mellitus (T2DM).

**Methods:** This is a cross-sectional study conducted with adult patients diagnosed with T2DM followed at the Nutrition Outpatient Clinic of a referral hospital in Fortaleza-Ceará. Participants had weight and height measured. WC and NC were measured with an inelastic tape measure at the midpoint between the last intercostal arch and the iliac crest and at the midpoint of the neck, respectively. A sociodemographic questionnaire was applied to investigate income, scholarity, practice of physical activity and the presence of comorbidities. Statistical analysis of the data was performed using the R software. The Shapiro–Wilk test was used to assess the normality of quantitative variables and the Spearman correlation coefficient to correlate the variables. The level of significance was 5%. This study was accepted by the Research Ethics Committee of the Federal University of Ceará under no. 83521518.0.0000.5045.

**Results:** The sample consisted of 58 patients, predominantly male (55.2%), with a mean age of 59 (± 13) years and a mean body mass index of 29 (± 4.4) kg./m^2^. Most individuals had an income of 1 to 4 minimum wages (75%) and had attended high school (94.6%). Most did not practice physical activity (58.6%), had systemic arterial hypertension (77.6%) and hypercholesterolemia (56.9%). WC and NC presented average values of 95 (± 12.9) cm and 39 (± 9.2) cm, respectively. Significant correlation was observed between WC and NC measurement (p < 0.001).

**Conclusions:** WC measurement was significantly correlated with NC measurement. Thus, the result suggests that NC is an anthropometric indicator as effective as WC for screening of insulin resistance in patients with T2DM.

### P60 Could serum branched-chain amino acids (BCAA) contribute to the increased cardiovascular in postmenopausal women?

#### Marilia Fonseca^1^, Bianca De Almeida-Pititto^2^, Isabela M Bensenor^3^, Paulo A Lotufo^3^, Sandra R G Ferreira^1^

##### ^1^Department of Epidemiology, School of Public Health, University of São Paulo, São Paulo, Brazil; ^2^Department of Preventive Medicine, Federal University of São Paulo, São Paulo, Brazil; ^3^Internal Medicine Department, University of São Paulo, São Paulo, Brazil

*Diabetology & Metabolic Syndrome* 2019, **11(Suppl 1):**P60

**Introduction:** Postmenopausal woman has increased cardiovascular risk, not all explained by traditional risk factors. Recently, BCAA have been associated with several cardiometabolic abnormalities in both sexes. Independent association with menopause was scarcely investigated, which could contribute to the increased risk of postmenopausal women. We tested association of serum BCAA with menopausal status (pre- or post-menopause).

**Methods:** This is a cross-sectional analysis of baseline data of women from the Sao Paulo centre of ELSA-Brasil stratified according to menopausal status. Exclusion criteria included menopause < 40 years and non-natural cause. Traditional risk factors and NMR spectroscopy-determined BCAA (valine, leucine and isoleucine) were compared using Mann–Whitney or Chi squared test. Multiple linear regression (BCAA as dependent variable) was performed to test independent association with menopausal category, adjusted for several factors. Additional analysis included comparisons of BCAA across 4 groups by combining presence/absence of metabolic syndrome (MS) and menopausal status: pre-menopause MS−, pre-menopause MS+, post-menopause MS− and post-menopause MS+.

**Results:** A total of 2,258 women (50.7 ± 8.8) were included. Postmenopausal women were older and had higher blood pressure, glucose, lipids, BCAA levels and higher prevalence rates of cardiometabolic disturbances than pre-menopausal ones. In multiple linear regression, central obesity, diabetes, hypertriglyceridemia, low HDL-c (components of the MS) were significantly associated with higher serum BCAA when the entire sample or menopausal categories were considered, but menopause was not independently associated with BCAA. Comparisons of BCAA within MS− and MS+ groups showed no difference between pre- and postmenopausal women. However, when comparing premenopausal or postmenopausal women, each with and without MS, higher BCAAlevels were consistently found in MS+ groups.

**Conclusion:** Our finding did not support that menopause is independently associated with circulating levels of BCAA in ELSA-Brasil, but the classical components of the MS.

**Keywords:** Menopause; Women; Branched-chain amino acids; BCAA; Metabolic syndrome.

### P61 Cut-off point for homeostatic model assessment for insulin resistance (HOMA-IR) index to detect metabolic syndrome in overweight adolescents

#### Eduarda Pontes dos Santos Araújo^1^, Thatyane Oliveira Souza^2^, Jessica Bastos Pimentel^2^, Angélica Luiza de Souza Sales^2^, Karla Simone Costa Souza^3^, Ricardo Fernando Arrais^4^, Adriana Augusto de Rezende^3^, Severina Carla Vieira Cunha Lima^2^

##### ^1^Programa de Pós-Graduação em Ciências da Saúde, Universidade Federal do Rio Grande do Norte (UFRN), Natal, Brazil; ^2^Programa de Pós-graduação em Nutrição, Universidade Federal do Rio Grande do Norte (UFRN), Natal, Brazil; ^3^Departamento de Análises Clínicas e Toxicológicas Universidade Federal do Rio Grande do Norte (UFRN), Natal, Brazil; ^4^Departamento de Pediatria, Universidade Federal do Rio Grande do Norte (UFRN), Natal, Brazil

*Diabetology & Metabolic Syndrome* 2019, **11(Suppl 1):**P61

**Introduction and objective:** Obesity is a important risk factor for the development of metabolic complications such as Insulin Resistance (IR) and Metabolic Syndrome (MS). However, specific IR cut-off points for early detection of changes are not yet well defined. The present work aims to establish the best cut-off point of the HOMA-IR Index for the detection of Metabolic Syndrome in overweight adolescents.

**Methodology:** This is a cross-sectional study, approved by the Research Ethics Committee, involving overweight or obese adolescents aged from 10 to 18 years old attending at a Pediatric Endocrinology Clinic. Anthropometric data (weight, height, Body Mass Index and Waist Circumference), blood pressure and biochemical data (fasting glucose, fasting insulin, HDL-c and triglycerides) were collected and analyzed. The diagnosis of MS was made according to the International Diabetes Federation (IDF) criteria. The HOMA-IR Index (Homeostatic Model Assessment) was calculated and the following cut-off points for IR diagnosis were adopted: 2.5; 3.0; 3.16 and 3.8. Sensitivity and specificity were estimated for each cut-off point, having as the outcome the MS, from the ROC (Receiver Operating Characteristic) curve.

**Results:** A total of 121 adolescents with a mean age of 11.45 (1.52) were evaluated, mostly male (51.2%) and classified as obese (65.3%). The overall mean BMI was 26.56 (3.53), Insulin 11.79 (6.97) and HOMA-IR 2.75 (1.73). A frequency of 30.6% of MS was observed. The test accuracy determined by the area under the ROC curve was shown to be 77% (p < 0.001). The cut-off point with the highest AUC was 3.16 (AUC = 0.733; S = 62.2%; E = 84.5%, p < 0.001), followed by 3.8 (AUC = 0.723; S = 54, 1%; E = 90.5%; p < 0.001), 3.0 (AUC = 0.722, S = 62.2%, E = 82.1%, p < 0.001) and 2.5 (AUC = 0.643, S = 70.3%, E = 58.3%, p = 0.002).

**Conclusion:** For the study population, the HOMA-IR index may be a good predictor for detecting MS, and the cut-off point 3.16 was the best for overweight and obese adolescents.

### P62 Descriptive study about the characteristics of the patients with gestational diabetes mellitus followed by a SUS’S (SISTEMA ÚNICO DE SAÚDE) secondary health care

#### Cristina Figueiredo Sampaio Façanha2.3^123^, Victoria Sudario Alencar^1^, Desyrrê Gabrielly Marques Gondim1^2^, Rejane Belchior Lima Macedo2^2^, Adriana Costa Forti^2^, Emanuel De Araújo Pinheiro^2^, Dayana De Alencar Mesquita Lima^2^, Mariana Salles Ballalai^3^

##### ^1^Centro Universitario Christus; Faculdade de medicina, Fortaleza, Brazil; ^2^Centro Integrado de diabetes e Hipertensão do Estado do Ceará- CIDH, Fortaleza, Brazil; ^3^Universidade Federal do Ceará, UFC, faculdade de Medicina, Fortaleza, Brazil, Bolsista DO CNQP

*Diabetology & Metabolic Syndrome* 2019, **11(Suppl 1):**P62

**Background:** Gestational Diabetes Mellitus (GDM), defined as a glucose intolerance of variable degree detected during pregnancy, is currently one of the most frequent complications of pregnancy, and affects about 18% of pregnant women in Brazil. In addition to disease-related maternal and fetal morbidity, women with a history of GDM have an increased risk of developing type 2 DM after delivery. In our midst we have an ongoing study aiming to develop educational measures for the prevention of postpartum T2DM in women with GDM - “Diabetes Prevention in women with previous gestational diabetes: a multicenter study of intensive lifestyle changes: LINDA BRAZIL (Schmidt, Duncan et al. 2016).

**Objective:** To describe the status of glucose tolerance after delivery in a cohort of patients from the study LINDA-BRAZIL from Fortaleza.

**Methods:** Research based on the LINDA-BRASIL study’s data base, among the patients recruited in Fortaleza between 2014 and 2019.

**Results:** 1,160 pregnant women with GDM, aged between 18 and 45 years were followed between 2014 and 2019. Among the pregnant women followed up, 18% had not completed elementary school and 40.6% had completed secondary school. As for family income, 22.6% lived with less than minimum wage and 40% receive between 1 or 2 times the minimum wage. We observed thinness in 0.8% of pregnant women, eutrophic in 22.2%, 36% were overweight and 41% obese. Regarding treatment, we observed that dietary treatment was sufficient for 57.5% of pregnant women, 28% were treated with metformin and 14.5% with insulin. The abortion rate in our group was 1.7% (20 patients). At the end of pregnancy, 44.1% had postpartum TOTG indicating post-DMG diabetes in 33.9% of those treated with insulin during pregnancy and 3.2% among those not treated with insulin, being type 2 diabetes diagnosed in 7.5% of the pregnant women evaluated postpartum.

**Conclusions:** In this cohort with a significant number of women with GDM, where postpartum reclassification was higher than the average observed in our country, we observed a significantly higher risk of developing postpartum DM2 in patients who needed insulin to control diabetes during pregnancy. The need for insulin therapy may correspond to women with more severe disease, thus with less tendency to complete glycemic normalization after delivery. Therefore, we consider it important to reinforce the orientation regarding the importance of postpartum DM reclassification, especially in this high-risk group.

### P63 Diabetes consulting: an education-centered approach

#### Samila Torquato Araújo, Tatiana Rebouças Moreira

##### Universidade Federal do Ceará, Fortaleza, Brazil

*Diabetology & Metabolic Syndrome* 2019, **11(Suppl 1):**P63

**Introduction:** Diabetes education aims to incorporate the necessary tools to achieve treatment goals and encourage self-care. Diabetes consulting is a personalized support modality for patient-centered therapy that is focused on self-management education.

**Objective:** To describe the experience of implementing a diabetes consulting service with an emphasis on education.

**Methods:** This was a descriptive study, with an experience report, carried out from May to July 2019 in the city of Fortaleza, Ceará state, Brazil. This work describes the implementation of a nursing consultancy service for diabetic patients based on the seven self-care behaviors proposed by the American Association of Diabetes Educators. The proposal consisted of the following items: development of an action plan, budget planning, creation of a visual identity, social media study, elaboration of service protocols, acquisition of materials/supplies and construction of the service portfolio.

**Results:** In January 2019, two nurse educators, specialist in diabetes education, created the *DiaCare Diabetes Consulting and Coaching*. A total of 36 appointments were carried out, of which six were in-hospital visits and the others corresponded to scheduled home visits. Remote support was also provided. The educational approaches included coaching of newly diagnosed patients, safe preparation and application of insulin, measurement of capillary blood glucose, monitoring drug therapy compliance, prescription review, hypoglycemia/hyperglycemia management, assessment of risk foot and foot care interventions, technology consulting, sensor installation and reporting, technical advice, continuing education, and motivational group therapy.

**Conclusions:** Education-centered diabetes consulting is an innovative proposal to assist patients in a personalized way to cope with the multiple nuances of diabetes mellitus and encourage self-care and treatment compliance.

### P64 Diabetes distress and advanced glycation end products in type 2 diabetic patients

#### Jéssica da Silva Cunha Breder^1^, Cynthia de Moura Borges^2^, Thaina Silva Guinami^1^, Natália Wilcesky Tosini Neves^1^, Andrei Carvalho Sposito^2^, Maria Helena de Melo Lima^1^

##### ^1^Faculdade de Enfermagem da Universidade Estadual de Campinas, Campinas, Brazil; ^2^Faculdade de Ciências Médicas da Universidade Estadual de Campinas, Campinas, Brazil

*Diabetology & Metabolic Syndrome* 2019, **11(Suppl 1):**P64

**Introduction:** The term “Diabetes Distress” (DD) means diabetes-related emotional stress. Studies have shown that high emotional stress related to diabetes is associated with poor glycaemic control. Advanced glycation end products (AGEs) in skin predict long-term diabetic complications before and after glycated haemoglobin adjustment. Skin autofluorescence (SAF) is a validated non-invasive measure of AGEs.

**Objective:** We investigated the association of diabetes-related emotional stress and skin AGE levels in type 2 diabetic patients.

**Methods:** This was a cross-sectional study approved by the Research Ethics Committee (no. 89525518.8.1001.5404/2018). Diabetes Distress Scale (DDS) was used to assess diabetes-related emotional stress. AGE accumulation in the skin was measured by SAF (AGE Reader^®^). Data distribution was assessed by the Shapiro–Wilk test and means by unpaired Student’s t-test.

**Results:** Of the 81 patients with type 2 diabetes evaluated, 58 patients (71.6%) had an DDS score greater than or equal to 3. Among patients with a DDS score greater than or equal to 3, the mean AGE was higher (2.78) when compared to the group with DDS score less than 3 (2.49/*p* 0.0439).

**Conclusion:** Patients with elevated diabetes-related emotional stress have higher levels of AGEs.

### P65 Diabetic patients show more abdominal obesity than non-diabetic patients: multicentric study in the Northeast/Brazil

#### Luciana de Brito Gonçalves Nascimento^1^, Viviane Sahade^2^, Bernadete Weber^3^, Josilene Maria Ferreira Pinheiro^4^, Carla Hilário da Cunha Daltro^1^

##### ^1^Programa de Pós Graduação em Medicina e Saúde (PPgMS)/Universidade Federal da Bahia (UFBA), Salvador, Brazil; ^2^Escola de Nutrição/UFBA, Salvador, Brazil; ^3^Hospital do Coração (HCOR), São Paulo, Brazil; ^4^Hospital Universitário Ana Bezerra/Universidade Federal do Rio Grande do Norte (UFRN), Santa Cruz, Brazil

*Diabetology & Metabolic Syndrome* 2019, **11(Suppl 1):**P65

**Introduction:** Increase of mortality due to cardiovascular diseases (CVD) concerns public health. Studies show deleterious impact of diabetes mellitus (DM) in cardiovascular morbidity.

**Objective:** compare clinical- nutritional profiles of CVD with and without DM.

**Methods:** cross-sectional study with individuals with CVD, aged ≥ 45, who had been attended in 10 specialized ambulatories in CVD—in 8 states in the Northeast, from 2013 to 2015. The variables were: clinical story, lifestyle, DM, systemic arterial hypertension (SAH), dyslipidemia, biochemical exams, body mass index (BMI) and Metabolic Syndrome (MS), according to NCEP-ATP III. Overweight was also considered—BMI ≥ 25.0 and ≥ 28.0 kg/m^2^, in adults and elderlies respectively, and obesity ≥ 30.0 kg/m^2^—in both. Records of food intake came from a 24-h food recall by Nutriquanti^®^. Data came from simple and relative attendance, average and standard deviation or median (Md) and interquartile range (IQR). The groups, with and without DM were compared by using the following tests: t Student, Mann–Whitney and Chi square—Pearson.

**Results:** 647 individuals were evaluated, with mean age of 63.1 (9.3) years, mainly females (50.5%), elderlies (61.1%), sedentary (73.3%)–40.3% of them diabetic patients. When the groups with and without DM were compared, the first one had more women (55.6% vs. 44.4%, p = 0.026), used more medicines [Md: 5.0 (IIQ: 4.0–7.0) vs. Md: 4.0 (IIQ: 3.0–5.0); p < 0.001], showed increased BMI [Md: 28.7 (IIQ: 25.6–32.4) vs. Md: 26.8 (IIQ: 24.5–29.8) kg/m^2^, p < 0.001], CC [Md: 100.0 (IIQ: 91.0–106.1) vs. Md: 95.5 (IIQ: 88.0–101.5); p < 0.001], waist-to-height ratio (WHtR) [Md: 0.6 (IIQ: 0.6–0.7) vs. Md: 0.6 (IIQ: 0.5–0.6); p < 0.001] and conicity rate [Md: 1.35 (IIQ: 1.29–1.39) vs. Md: 1.32 (IIQ: 1.27–1.38); p = 0.004]. We found higher levels of triglycerides [Md: 139.0 (IIQ: 101.5–191.0) vs. Md: 127.0 (IIQ: 92.0–173.5) mg/dL, p = 0.010] and fasting glycemia [Md: 131.0 (IIQ: 106.0–173.5) vs. Md: 98.0 (IIQ: 90.0–107.0), p < 0.001], beyond a higher percentage of SAH (93.5% vs. 87.0%, p = 0.008) and MS (98.8% vs. 80.4%; p < 0.001). No statistical differences related to age, lifestyle variables and food intake were found.

**Conclusions:** Despite not having showed a difference regarding food intake, diabetic patients with CVD showed more abdominal obesity and MS than individuals without DM.

*Study supported by* PROADI-MS.

### P66 Diabetic retinopathy is a strong predictor of elevated calcium score

#### Michele Santana, Ikaro Soares Santos Breder, Vicente Hidalgo Fernandes, Fernando Rodrigo Pedreira Chaves, Cynthia Borges, Carlos Arieta, Otavio Rizzi Coelho-Filho, Andrei Carvalho Sposito

##### Faculdade de Ciências Médicas da Universidade Estadual de Campinas, Campinas, Brazil

*Diabetology & Metabolic Syndrome* 2019, **11(Suppl 1):**P66

**Introduction:** Advanced glycation end products (AGE) induces diabetic retinopathy (DR). Likewise, calcifications of vascular smooth muscle cells are mediated by AGE.

Thus, it is directly correlated with coronary calcium score (CAC), an useful tool to predict cardiovascular events. Our hypothesis is that patients with DR are at increased risk of elevated CAC throw a common pathway that involves AGE formation.

**Objective:** To investigate the association between DR and CAC and to estimate the mediating effect of AGE in patients with T2DM.

**Methods:** A total of 198 patients, mean age (59 ± 7 years), were consecutively evaluated, including a retinal biomicroscopy, cardiac computed tomography and autofluorescence of the skin (sAF) to measure the AGE. The degree of RD was classified as non-apparent RD (NADR), non-proliferative RD (NPDR) and proliferative RD (PDR).

**Results:** A gradual increase of AGE-sAF was observed between individuals with NADR (2.7 ± 0.5), NPDR (3.1 ± 0.3) and those with PDR (3.6 ± 0.7; p < 0.005). Likewise, CAC progressively increased from those with NADR (174 ± 396) to those with NPDR (284 ± 432) and with PDR (1361 ± 3256; p < 0.0001). DR was found in 14% of patients with CAC < 10 and 9% of those with CAC = 0. CAC ≥ 10 doubles the chance of DR (RR 2.12, 95% CI 1.04–4.36; p = 0.04) and CAC = 0 reduces the chance of DR in 72% (RR 0.28, 95% CI 0.10–0.77; p = 0.01).

**Conclusion:** Patients with DR presents higher values of CAC. This association probably is mediated by a common pathway related to AGE accumulation.

### P67 diets products in the food market that attend the proposals by Brazil food trends 2020

#### Ana Paula Varela Sanches^1^, Josilene Lopes de Oliveira^1^, Camila Neves Rodrigues^2^, Fernanda de Oliveira Araújo^3^, Maíra Schuchter Ferreira^1^, Ana Iris Mendes Coelho^3^, Eliana Carla Gomes de Souza^3^

##### ^1^Universidade Estadual de Campinas, Campinas, Brazil; ^2^Universidade Federal de Minas Gerais, Belo Horizonte, Brazil; ^3^Universidade Federal de Viçosa, Viçosa, Brazil

*Diabetology & Metabolic Syndrome* 2019, **11(Suppl 1):**P67

**Introduction:** The Increase purchasing power of the population, greater access to information, increased schooling, modification in family structure and population aging, between other factors, have led to changes in preferences and choices regarding the food to be consumed. To analyze market trends and food consumption in the country, was proposed Brazil Food Trends 2020. For this study, 5 food trends are used: (I) Sensoriality and Pleasure (II) Healthiness and Wellness (III) Convenience and Praticity (IV) Reliability and Quality (V) Sustainability and Ethics. An industry challenge is to invest in one trend without harming others.

**Objective:** To perform a survey of diets in the food market that attend the categories of “new food trends” proposed by Brazil Food Trends 2020.

**Methodology:** We searched for information regarding the categories of new food trends on food labels and on official food industry websites. 45 products were evaluated.

**Results:** From the total of 124 evaluated, 100% met the healthiness, 51.61% the sensoriality, this shows us that when a product has no added sugar, it tends to be healthier, not losing palatability, probably the industry makes use of other components to not lose this effect. The convenience item represented 28.22% of the analyzed products and we noticed that it was not as prominent in the diets foods, as we observed in the case of sensoriality. The quality/reliability was met in a reasonable number of foods (22.58%), which is interesting for the diabetic patient because he needs to be sure about the information on the labels. Sustainability was mentioned in 3.22% of products used, showing that this is not a major concern of the diets food industry. Only 2 foods met the five items addressed by the “Brazil food trends” proposal.

**Conclusion:** Diet products in the food market that primarily meet the Health and Sensitivity trend among the “New Food Trends” categories proposed by Brazil Food Trends 2020. For the manufacture of diet products that have all five trends, there is still a long way to go.

### P68 differences in HBA1C reduction between insulin glargine 300 U/ml (GLA-300) and insulin degludec 100 U/ml (IDEG-100) in adults ≥ 70 years of age with T2DM in the bright trial

#### Bernard Charbonnel^1^, Vanita R. Aroda^2^, Jukka Westerbacka^3^, Felipe Lauand^3^, Emmanuelle Boëlle-Le Corfec^3^, Alice Y. Cheng^4^, Julio Rosenstock^5^, Geremia B. Bolli^6^

##### ^1^Nantes University, Nantes, France; ^2^Brigham and Women´s Hospital, Boston, USA; ^3^Sanofi, Paris, France; ^4^Division of Endocrinology and Metabolism, University of Toronto, Toronto, Canada; ^5^Dallas Diabetes Research Center at Medical City, Dallas, USA; ^6^Perugia University Medical School, Perugia, Italy

*Diabetology & Metabolic Syndrome* 2019, **11(Suppl 1):**P68

**Introduction and objectives:** BRIGHT (NCT02738151) showed similar glycemic control, with relatively low hypoglycemia risk, with Gla-300 and IDeg, but less anytime (24 h) hypoglycemia with Gla-300 during the first 12 weeks, among adults with in insulin-naïve, uncontrolled type 2 diabetes (T2D). Given the increasing prevalence of T2D and the higher risk of hypoglycemia among the elderly, the current analysis of selected endpoints from BRIGHT describes results in the subgroups aged ≥ 65 years (pre-planned) and aged ≥ 70 years (post hoc).

**Methods and results:** In this open-label, 24-week, noninferiority trial, pts on different oral agents ± a GLP-1 receptor agonist were randomized to Gla-300 (n = 466) or IDeg (n = 463), titrated to a fasting self-measured plasma glucose of 80–100 mg/dL. Changes in Hb1Ac, rates of confirmed hypoglycemia (≤ 70 mg/dL), and percentages of pts with ≥ 1 hypoglycemic (≤ 70 mg/dL) event were computed and compared between Gla-300 and IDeg for the overall population, for pts aged ≥ 65 years (n = 333), and for those aged ≥ 70 years (n = 161). Hb1Ac reductions from baseline to week 24 were similar between Gla-300 and IDeg for the overall population and pts aged ≥ 65 years. There was a significant interaction (p = 0.0087) between age (< 70 vs ≥ 70) and Hb1Ac change, with a least-squares mean difference between Gla-300 and IDeg of − 0.34% (95% confidence interval: − 0.59%, − 0.10%) for those aged ≥ 70 and no significant difference between Gla-300 and IDeg among those aged < 70. Rate ratios for hypoglycemia events/pt-years and odds ratios for pts with ≥ 1 hypoglycemic event were not significantly different from 1.00 in any of the populations assessed, indicating similarities between Gla-300 and IDeg for these safety parameters within each population. Insulin dose increase was slightly greater with Gla-300 than with IDeg in the overall and ≥ 65 years populations, but similar in both treatment arms in the ≥ 70 years subgroup.

**Conclusions:** Gla-300 provided similar HbA1c reductions vs IDeg in elderly pts with T2D, and greater reductions vs IDeg in those aged ≥ 70 years, with no increased risk of hypoglycemia in this potentially frail population. The trial was sponsored by Sanofi.

### P69 Differences in HBA1C-lowering effect and hypoglycemia risk between GLA-300 and ideg according to renal function in the bright trial

#### Martin Haluzik^1^, Athena Philis-Tsimikas^2^, Zsolt Bosnyak^3^, Dirk Müller-Wieland^4^, Felipe Lauand^3^, Lydie Melas-Melt^5^, Julio Rosenstock^6^, Geremia B. Bolli^7^

##### ^1^Institute for Clinical and Experimental Medicine, Prague, Czech Republic; ^2^Scripps Whittier Diabetes Institute, San Diego, USA; ^3^Sanofi, Paris, France; ^4^University Hospital RWTH Aachen, Aachen, Germany; ^5^Ividata, Levallois-Perret, France; ^6^Dallas Diabetes Research Center at Medical City, Dallas, USA; ^7^Perugia Medical School, Perugia, Italy

*Diabetology & Metabolic Syndrome* 2019, **11(Suppl 1):**P69

**Introduction and objectives:** BRIGHT (NCT02738151) showed similar glycemic control, with relatively low hypoglycemia risk, with the second-generation basal insulin analogs Gla-300 and IDeg, but less anytime (24 h) hypoglycemia with Gla-300 during the first 12 weeks. This exploratory analysis of BRIGHT assessed HbA1c changes and confirmed hypoglycemia according to renal function.

**Methods and results:** BRIGHT was a multicenter, open-label, 24-week, noninferiority trial in insulin-naïve patients (pts) with uncontrolled T2D. Pts on different oral agents ± a GLP-1 receptor agonist were randomized to Gla-300 (n = 466) or IDeg (n = 463), titrated to a fasting self-measured plasma glucose of 80–100 mg/dL. Among pts eligible for the current analysis (n = 462 per arm), renal function was categorized as estimated glomerular filtration rate (eGFR) of ≥ 90 (n = 428), 60–90 (n = 337), and < 60 mL/min/1.73 m^2^ (n = 89). Anytime (24 h) confirmed hypoglycemia (≤ 70 mg/dL) was used to compute hypoglycemia events/pt-year and percentages of pts with ≥ 1 hypoglycemic event in each subgroup. The comparative efficacy and safety of Gla-300 and IDeg did not vary by age or T2D duration. However, significant heterogeneity of treatment effect across subgroups (p = 0.015) suggested greater HbA1c reduction with Gla-300 than IDeg among pts with eGFR < 60 mL/min/1.73 m^2^ (least squares mean change from baseline, − 0.43 [95% confidence interval (CI): − 0.74, − 0.12]), with no difference in hypoglycemia frequency in this subgroup. Conversely, there was less confirmed (≤ 70 mg/dL) hypoglycemia with Gla-300 vs. IDeg in pts with normal renal function, with comparable HbA1c reduction in this subgroup. Among pts with eGFR ≥ 90 mL/min/1.73 m^2^, there were 6.5 and 10.4 events/pt-years with Gla-300 and IDeg, respectively (rate ratio, 0.60; 95% CI: 0.45, 0.81), and the proportions of pts with ≥ 1 hypoglycemic event were 59.9% and 65.0% with Gla-300 and IDeg, respectively (odds ratio, 0.74; 95% CI: 0.50, 1.10).

**Conclusions:** Gla-300 appears to confer advantages over IDeg depending on renal function, with higher efficacy among pts with impaired renal function and less hypoglycemia among those with normal renal function, something that warrants further investigation. The trial was sponsored by Sanofi.

### P70 Difficulty in achieving lipid target in type 2 diabetes

#### Paula Ferreira do Rego Barros, Livia Furtado Leba, Marcela Martins Campos, Ronaldo Gama Pena, Cátia Cristina Silva Sousa Vergara Palma, Roberta Arnoldi Cobas, Raquel de Carvalho Abi Abib, Roselee Pozzan

##### Universidade do Estado do Rio de Janeiro, Rio de Janeiro, Brazil

*Diabetology & Metabolic Syndrome* 2019, **11(Suppl 1):**P70

**Introduction:** In recent years the guidelines for the prevention of cardiovascular disease (CVD) in diabetes (DM) have set lower lipid goals for patients with elevated risk. However, there are many barriers in achieving these goals, especially in public health system patients.

**Objective:** To investigate the characteristics of lipid management and the percentage of patients within reccomendedLDLc targets according to cardiovascular risk and the clinical factors associated with achieving these goals.

**Methodology:** Cross-sectional study, including data from medical records of patients with type 2 DM from March to June 2019. The criteria of cardiovascular (CV) risk and lipid goals were those of the 2017 SBD/SBC/SBEM Guideline. Results are presented as mean ± standard deviation and n(%). SPSS/IBM software version 25.0 was used for analysis.

**Results:** We evaluated 102 patients aged 61.8 ± 9.1 years, 51% female, with DM duration 17.6 ± 9.4 years, HbA1c 7.98% ± 1.58, BMI 30.8 ± 5.9 kg/m^2^, waist circumference 102.4 ± 13.8 cm. Overall, 8% were smokers, 86.3% had hypertension, 20.6% history of CVD and 31.3% family history of early CVD, 30% presented kidney dysfunction, 37% retinopathy/maculopathy, 38.5% peripheral neuropathy. Regarding CV risk, 20 (19.6%) patients were classified as very high, 81 (79.4%) high and 1 (0.98%) intermediate/low. Of them, 92.2% used statin (67% simvastatin, 20.2% atorvastatin, 12.8% rosuvastatin). Lipid goal achievement was observed in 32.3% of overall patients and innone (0%) at low/intermediate risk, 37% at high risk and 15% at very high risk. Sixteen percent of the high risk and 35% of the very high risk patients are using the recommended dose of statin. Patients who did not reach LDLc targets were mostly women (39.2% vs 28.4%; p = 0.041), smokers (8.0 vs 0%; p = 0.043), had higher A1c (8.17 ± 1.62 vs 7.55 ± 1.43%; p = 0.002) and systolic blood pressure (145.3 ± 21.1 vs 135 ± 20; p = 0.022). No difference was observed regarding age, duration of DM, BMI, waist circumference, presence of microvascular diabetes complications.

**Conclusion:** In our sample of type 2 DM almost all were classified as very high and high CV risk despite only 15% and 37% of them reaching the proposed targets for LDL-c for CV prevention, respectively. Women, smokers and patients with worse glycemic and pressoric control were more likely to be out of recommended targets for LDL-c. This may mean less adherence to treatment as a whole. Theses patients need better attention and vigilance.

### P71 Discontinuation of insulin therapy postpartum in a patient with a previous diagnosis of nesiodioblastosis

#### Marina Cutrim Magalhães Vieira, Potira Almeida Gurgel de Azevedo, Bárbara Karolyne Pereira Sousa, Matheus Trinconi Cunha, Letícia Jácome Pereira, Mariana Vilela Pereira, Cecilia Kauffman, João Eduardo Nunes Salles

##### Irmandade da Santa Casa de Misericórdia de São Paulo, São Paulo, Brazil

*Diabetology & Metabolic Syndrome* 2019, **11(Suppl 1):**P71

**Report of case:** A 21-year-old patient diagnosed with nesidioblastosis at 2 years of age when pancreatectomy was performed, removing 95% of pancreas. Started using a night dose of NPH insulin at 10 years old and became pregnant at 18 years old using 8 units of NPH insulin in the morning and 2 grams of Metformin. During pregnancy 2 units of Lispro insulin was added before each main meal. At time the HbA1C was 6%. After the baby was born in an uncomplicated pregnancy, at 39 weeks, the patient had repeated episodes of hypoglycemia and no longer needed insulin for about 180 days. Nowadays patient is using basal-bollus regimen to control hyperglycemia, 28 units of NPH insulin and 30 units of regular insulin per day. The currently HbA1C is 10/2%.

**Discussion:** Nesidioblastosis, a rare cause of endogenous hiperinsulinemic hypoglycemia described mainly in newborns, refers to neoformation of islets of Langerhans from pancreatic duct epithelium. The long-term therapeutic approaches are directed toward decreasing insulin secretion. This may be accomplished pharmacologically or surgically. Near-total pancreatectomy is associated with high rates of insulin-dependent diabetes mellitus and exocrine pancreatic insufficiency. Little is known about the management and long-term glycaemic control of patients with diabetes after pancreatic surgery. In this case the patient lost almost her total pancreas mass, but after giving birth paciente had new hypoglycemia episodes. The main hypothesis for this postpartum glycemic control is the possibility of remaining pancreatic beta cell hyperplasia, probably stimulated by increased growth hormones in pregnancy, such as GH.

**Conclusion:** It is very important that cases of nesidioblastosis be followed for life and more studies should be done to verify why there is a proliferation of beta cells still capable of producing insulin.

Informed consent to publish had been obtained from the patient.

### P72 Does using a glucose meter integrated to an app mobile improve adherence in patients with insulin-dependent diabetes mellitus in a multiple daily injections regimen?

#### Joao Eduardo Nunes Salles^1^, Felipe Martins de Oliveira^1^, Cecília Kauffman Rutenberg Feder^1^, Maria Fernanda Ozorio de Almeida^1^, Mariana Vilela Pereira^1^, Maria Thereza Teixeira de Almeida Fagundes Alves^2^, Sônia Aparecida Dias Garcia^2^, Ligia Dinara Donizeti Reis^2^

##### ^1^Irmandade Santa Casa de Misericórdia de São Paulo, São Paulo, Brazil; ^2^Associação dos Diabéticos de Ourinhos (ADO), Ourinhos, Brazil

*Diabetology & Metabolic Syndrome* 2019, **11(Suppl 1):**P72

**Introduction:** Glycated hemoglobin (A1C) has been used to estimate treatment adherence in diabetes mellitus (DM). Recently, mobile apps have showed an important role in helping this adherence. Finally, there is a growing interest in self-reported adherence questionnaires, such as DSMP (Diabetes Self-Management Profile).

**Objectives:** To compare patients under a multiple daily injections (MDI) insulin regimen using Johnson & Johnson app OneTouch Reveal^®^ with another group of subjects under continuous subcutaneous insulin infusion (CSII) therapy.

**Methods:** 75 patients who regularly attend, in Ourinhos-SP, Brazil, were selected for a parallel, open, nonrandomized, two-arm clinical treatment trial: an intervention (use of OneTouch Reveal^®^ app) group containing 50 patients (half under simple MDI and half under carbohydrate counting (CC)) and a control group of 25 CSII users. Inclusion criteria: diagnosis of type 1 or LADA (Latent Autoimmune Diabetes of the Adult) DM, regular use of ultra-slow and/or ultra-fast insulin analogues, and CC and CSII for at least 3 months. A1C was collected and DSMP was applied at the beginning and at the end of 12 weeks. The outcome of this analysis was the combined finding of absolute reduction of A1C values and increase in total DSMP score between initial and final evaluations.

**Results:** At the end of the study, 65 individuals remained. There was an average variation of 4.7 points between initial and final DSMP values with statistically significant (p-value = 0.0132) increase in this score for the group who used OneTouch Reveal^®^ app when compared to control group (CSII). There was a mean variation of 0.411 from initial and final A1C values with a relevant reduction (p-value = 0.08) for patients using the mobile app when compared to control group. At a p-value = 0.06, the proportion of patients who achieved the combined DSMP score increase plus A1C reduction was 23% higher in the intervention group.

**Conclusion:** Using OneTouch Reveal^®^ app improves both objective (A1C) and subjective (DSMP) treatment adherence parameters for insulin-dependent DM patients compared to standard treatment (CSII). Devices OneTouch Select Plus Flex^®^ were donated by Johnson & Johnson company to the patients in this research.

### P73 Double type 1 diabetes mellitus and type 2 diabetes mellitus have the same degree of insulin action in vivo and relationship to cardiovascular risk factors

#### Luana Aparecida de Lima Ramaldes, Daniela Lino Maltez Bezerra, Patricia Medici Dualib, João Roberto de Sá, Sérgio Atala Dib

##### Disciplina de Endocrinologia da Escola Paulista de Medicina - Universidade Federal de São Paulo, São Paulo, Brazil

*Diabetology & Metabolic Syndrome* 2019, **11(Suppl 1):**P73

**Introduction:** The overweight and obesity prevalence is increasing globally and consequently the incidence of type 2 diabetes mellitus (T2DM). Currently about 1/3 of individuals with Type 1 Diabetes Mellitus (T1DM) are overweight or obese and are denominated as Double Diabetes (DD). However, the overall characteristics of this group are still not well defined.

**Objective:** To evaluate the insulin sensitivity of healthy individuals, with DD and T2DM and to compare their clinical and metabolic characteristics.

**Methods:** Eleven patients with DD compatible characteristics were recruited, termed as T1DM and at least two of the following criteria: systemic arterial hypertension (defined by NCEP), overweight or obesity (WHO), dyslipidemia (NCEP) and clinical signs of peripheral insulin resistance. Also 19 patients with a recent diagnosis of T2DM according to ADA criteria were recruited and 8 healthy individuals. The insulin tolerance test, plasma glucose disappearance rate (K_ITT_), age, sex, waist-hip ratio, BMI, presence of hypertension, family history of T2DM were evaluated for the three groups, and the total cholesterol and fractions were evaluated only in the T2DM and DD groups. Statistical analysis were performed using the software SPSS20, t-test, Chi square test and ANOVA; p < 0.05. The study was approved by the ethics committee.

**Results:** In the group of DD 54.5% sex ♀, family history of T2DM 54.5% age 22.4 ± 4.6y (mean ± SD), BMI: 29.0 ± 2.7 kg/m^2^, waist-hip ratio. hip 0.86 ± 0.07, HbA1c 8.1% ± 0.64 total cholesterol 180.0 ± 35.9 mg/dl), HDL 55 ± 16.1 mg/dL, HAS 18%. In the T2DM group 68.4% sex♀, family history of T2DM 73.7%. age 55.3 ± 5.3 a, BMI 29.56 ± 2.6 kg-m^2^, waist-hip ratio 0.974 ± 0.05, HbA1c 4.9% ± 1.0; total cholesterol 229.0 ± 74.2 mg/dL, HDL 37.3 ± 9.1 mg/dL, 18% HAS. In the group of healthy subjects 87.5% sex ♀, family history of T2DM 0% age 45 ± 7, BMI 30.1 ± 2.6 kg/m^2^, waist-hip ratio 0.93 ± 0.08, HbA1c 3.4% ± 0.33, HAS 0%. The K_ITT_ was the same for DD and T2DM (2.44 + 1.31%/min vs 2.47 + 1.10%/min; ns) and different in the healthy group (3.99 ± 0, 82%/min p < 0.001). The other variables studied were also similar between the DD and T2DM (ns) groups.

**Conclusions:** DD individuals presented a similar level of insulin action in vivo and cardiovascular risk factors (CRV) compared to T2DM and insulin action lower than healthy individuals. Considering the differences in age groups, the study suggests an early acquisition of CRV in the DD. CAPES financial support.

### P74 Drug by molecular hybridization: an analysis in silico using a dipeptidyl peptidase-4 inhibitors molecule (linagliptin) and a sulfonylurea (glicazide)

#### Luís Jesuino de Oliveira Andrade^1^, Ana Paula Rodrigues dos Santos^2^, Alcina Maria Vinhaes Bittencourt^3^, Gabriela Correia Matos de Oliveira^4^

##### ^1^Departamento de Saúde - UESC - Ilhéus, Brazil; ^2^Residência Clinica Médica - Santa Casa de Itabuna - Itabuna, Brazil; ^3^Faculdade de Medicina - UFBA - Salvador, Brazil; ^4^Faculdade de Medicina - FTC - Salvador, Brazil

*Diabetology & Metabolic Syndrome* 2019, **11(Suppl 1):**P74

**Introduction and objective:** Molecular hybridization (MH) is a strategy of rational design of a new drug, based on the adequate combination of drugs of the different substances with the aim of to produce a chimera more effective in relation to the parent drugs and less side effects. Dipeptidyl peptidase-4 (DPP-4) is related with the degradation of GLP-1 and GIP hormones, increasing the production and release of insulin. Thus, hybrid compounds of DPP-4 inhibitors with other antidiabetic drugs can be formulated for evaluation. The aim of the study was to demonstrate in silico by MH the production of a hybrid molecule from a DPP-4 inhibitor molecule (Linagliptin) and a sulfonylurea (Glicazide), in order to suggest new proposal of an oral antidiabetic for treatment of type 2 diabetes mellitus (T2DM).

**Method and results:** We hybridized in silico the structure of two drugs, using as search tool of structures the website Protein Data Bank archive, 2RGU (Crystal structure of complex of human DPP4 and inhibitor) and 4ZFC (Crystal structure of AKR1C3 complexed with glicazide), generating 1 molecule that include the parent drugs. The PDB code: 4ZFC was docked computationally to the active site of PDB code: 2RGU. Structural analyses of the 3D generated model of the new molecule showed that molecule is constructed with two chains with the co-crystallized ligand in each chain.

**Conclusion:** The generation of a molecule by MH in the described in silico experiment with interaction of a sulfonylurea and a DPP-4 inhibitor may be a qualitative therapeutic option of an oral antidiabetic for treatment of T2DM.

**Keywords:** Antihyperglycemic agents; Drug design; Molecular hybridization.

### P75 Dulaglutide (DU) has better glycemic effectiveness vs basal insulin (BI) in injection naïve patients with type 2 diabetes (T2D): the dispel TM study

#### Anderson Leandro Couto Peres Ribeiro^1^, Reema Mody^2^, Qing Huang^3^, Maria Yu^4^, Xian Zhang^3^, Liya Wang^3^, Michael Grabner^3^, Hiren Patel^2^

##### ^1^Eli Lilly and Company, São Paulo, Brazil; ^2^Eli Lilly and Company, Indianapolis, USA; ^3^HealthCore, Inc., Wilmington, USA; ^4^Eli Lilly and Company, Toronto, Canada

*Diabetology & Metabolic Syndrome* 2019, **11(Suppl 1):**P75

**Introduction and objectives:** The 2018 ADA-EASD consensus report recommends GLP-1 RAs over BI as the first injectable medication in most patients with T2D. The objective of this US retrospective observational study was to compare 1-year real-world glycemic effectiveness among patients with T2D initiating DU vs BI.

**Methods:** Patients ≥ 18 years with T2D initiating DU or BI between Nov’14–Apr’17 (index date = earliest fill date), and no claim for any antidiabetic injectable in 6 months pre-index period (baseline), continuous enrollment and ≥ 1 HbA1c result 6 months pre-index and 1-year post-index were identified from a US claims database. DU users were propensity-matched 1:1 to BI users.

**Results:** The pre-matching mean baseline HbA1c for DU cohort (n = 1,103) was 8.4% vs 9.9% for BI cohort (n = 3,193). Matched cohorts (903 pairs) were balanced in baseline characteristics with mean HbA1c: ~ 8.6%, mean age: 54 years, SGLT2 inhibitor use: 24% and DPP-4 inhibitor use: ~ 38%. At 1-year post-index, 11% of DU cohort used BI and 10% of BI cohort used GLP-1 RAs; DU patients used less rapid-acting insulin (2% vs 16%) and DPP-4 inhibitors (24% vs 39%) and more SGLT2 inhibitors (34% vs 23%) vs BI patients. For the matched cohorts, change (mean; SE) in HbA1c levels from baseline was significantly greater in the DU (− 1.12; 0.05) vs the BI cohort (− 0.51; 0.05) (p < 0.01). HbA1c level was reduced by ≥ 1% or decreased to < 7% in significantly more number of patients in the DU (65.6%) vsthe BI cohort (45.3%) (p < 0.01). Among patients with baseline HbA1c > 9%, change (mean; SE) in HbA1c levels was significantly greater in the DU (− 2.11; 0.10) vs the BI cohort (− 1.52; 0.10) (p < 0.01). Similar observations were made in patients aged ≥ 65 years.

**Conclusion:** This real-world study, patients with T2D initiating DU demonstrated significantly greater and clinically meaningful HbA1c reduction compared to those initiating BI.

### P76 Educational intervention for the prevention of diabetic foot in a center of medical specialties

#### JoseiKarly Santos Costa Motta^1^, Alexandra Dias Moreira^1^, Suelen Rosa de Oliveira^1^, Ângelo Gustavo Venâncio de Lima^2^, Helem Sena Ribeiro^1^, Aleida Nazareth Soares^1^, Tatiane Géa Horta Murta^1^

##### ^1^Instituto de Ensino e Pesquisa da Santa Casa de BH, Belo Horizonte, Brazil; ^2^Escola de Enfermagem da Universidade Federal de Minas Gerais, Belo Horizonte, Brazil

*Diabetology & Metabolic Syndrome* 2019, **11(Suppl 1):**P76

**Introduction:** Diabetes mellitus is defined as a metabolic disorder, which affect people in any age group in a progressive and degenerative way. In Brazil, diabetes occupies the highest incidence of hospitalizations and mortality due to amputations of lower limbs as a consequence of neuropathy or diabetic foot.

**Objective:** To evaluate the efficacy of an educational intervention on behavioral changes for the prevention of diabetic foot in people with diabetes mellitus treated at a medical specialty center in the city of Belo Horizonte, Minas Gerais.

**Methods:** Quasi-experimental study. The data collection was carried out in the period from August to November of 2018, in three phases: the first, face-to-face and the second and third via telephone contact. In the first phase, a face-to-face interview was conducted using a questionnaire with sociodemographic questions and self-care practices with the feet. Still in this step was used the “Healthy foot guide: foot care for people with diabetes”. In the second and third phases the same questionnaire was reapplied and the guidelines on preventive foot care were reinforced. For data analysis, the McNemar and Wilcoxon tests were used.

**Results:** It was found that 75% of the participants were female, with a mean age of 44.8 ± 17.6, 46.8% were single, 62.5% had low level of schooling, the majority were 68.8% diabetes mellitus type 2. The use of closed and soft shoes (p = 0.012 and p = 0.006) and self-examination of the feet (p = 0.039) were statistically significant after 15 and 45 days of intervention. The median score before the intervention was 4 points. After 15 days this score increased to 5 and was maintained after 45 days, being this increase statistically significant (p < 0.005).

**Conclusions:** The educational intervention was effective for the changes in behaviors aimed at the prevention of diabetic foot.

### P77 Effect of cecropia pachystachya aqueous extract treatment on the maternal outcomes of diabetic rats

#### Beatriz Martins Holtz, Cristielly Maria Barros Barbosa, Vanessa Liones da Costa, Catarina Freitas Oliveira, Linne Stephane Furtado, Maysa Rocha de Souza, Rafaianne Queiroz de Moraes-Souza, Gustavo Tadeu Volpato

##### FisioTox - UFMT/CUA, Araguaia, Brazil

*Diabetology & Metabolic Syndrome* 2019, **11(Suppl 1):**P77

**Introduction and objectives:** Several medicinal plants are used without criteria by the population for the diabetes treatment. There are no studies to prove the effects of *Cecropia pachystachya*, especially administered in the gestational period. The objective of this study was to evaluate the maternal effects of rats treated with *C. pachystachya* during diabetic pregnancy.

**Methods:** Diabetes was induced on the first day of life of newborn female Wistar rats by subcutaneous injection with Streptozotocin or citrate buffer (control animals). On the 110th day of life, the Oral Glucose Tolerance Test (OGTT) was performed to confirm diabetes. Subsequently, these rats were mated and randomized into 4 experimental groups (n = 12 animals/group): Control (C)—non-diabetic rats treated with vehicle (water); Treated (T)—non-diabetic rats treated with *C. pachystachya* extract; Diabetic (D)—diabetic rats treated with vehicle; Diabetic Treated (DT)—diabetic rats treated with the plant. The plant extract was administrated by gavage throughout pregnancy (dose of 200 mg/kg). The OGTT was performed on the 17th day of pregnancy. On the 21st day of pregnancy, rats were anesthetized, and blood was collected for biochemical evaluations. A laparotomy was performed, and the kidneys, spleen, liver, heart and pancreas were removed for weighing, and the uterus with its contents was analyzed for maternal reproductive evaluations.

**Results:** The area under the curve (AUC) of the OGTT was higher in the Diabetic (16857 ± 3531.8 mg/dL*120 min) and Diabetic Treated (13817 ± 1995 mg/dL*120 min) groups than in the C group (9576 ± 1464 mg/dL*120 min), but the DT group presented a reduction of the AUC compared to the group D. There was an increase in the relative weight of the pancreas and spleen of the DT group compared to the C. The embryo loss after implantation was increased in the DT group (11.7%) in relation to others groups (C = 2.6%; T = 5.7%; D = 5.5%).

**Conclusion:** The administration of the aqueous extract of *C. pachystachya* during the pregnancy of diabetic rats improves glucose uptake, but causes in embryonic losses. Therefore, the use of this plant during pregnancy should be done with caution until further studies are conducted to determine if its beneficial effects outweigh the deleterious effects.

**Financial support:** CAPES/FAPEMAT.

### P78 Effect of high vitamin d doses on diabetic retinopathy in patients with type 1 diabetes mellitus

#### Sávio Diego do Nascimento Cavalcante, Bruna Eduarda Peres Maciel Castro, Maria Clara Neres Iunes de Oliveira, Lorena Vilhena de Moraes, Ana Carolina Contente Braga de Souza, Márcia Costa dos Santos, Karem Miléo Felício, João Soares Felício

##### Universidade Federal do Pará, Belém, Brazil

*Diabetology & Metabolic Syndrome* 2019, **11(Suppl 1):**P78

**Introduction:** The role of vitamin D (25 (OH) D in diabetic retinopathy (RD) or diabetic macular edema (ME) is not yet clear in the literature, despite studies showing promising results on the beneficial effect of vitamin D at microvascular level that have demonstrated the improvement of microalbuminuria after vitamin D replacement in type 1 diabetic individuals (DM1).

**Objective:** To evaluate the impact of 25(OH)D supplementation in diabetic retinopathy, on patients with T1DM.

**Methods:** We performed a cohort study, approved by ethics committee, in which 44 patients with T1DM were assessed. Anthropometric measures, serum levels of 25(OH)D, HbA1c, urinary albumin/creatinine ratio and a simple retinography were evaluated in the first visit. Next, all patients were divided, according to 25(OH)D levels, in two groups. One group, with 25(OH)D 30–60 ng/dl, received 4000 UI/day cholecalciferol supplementation, while the other, with 25(OH)D levels < 30 ng/dl, received 10000 UI/day. Both groups took the medication for 12 months, after when, they were re-assessed in the same parameters evaluated in the first visit.

**Results:** The prevalence of RD before vitamin D supplementation (N = 40) was 65% (N = 26) and after supplementation (N = 22) was 50% (N = 11). The sensitivity and specificity of vitamin D serum levels as a predictor of diabetic retinopathy showed a low accuracy for its diagnosis, performing a cut-off point of 24.5 ng/dL vitamin D, with sensitivity of 57.1% and specificity of 69.2%. 03 patients have improved ME after supplementation, between that was observed an increase of vitamin D in all patients without any variation of glycated hemoglobin, that suggesting a possible effect of vitamin D supplementation on ME likely due to its anti-inflammatory effect.

**Conclusion:** We found no association between the basal levels of 25(OH)D and DR. The low prevalence of DR in our casuistic may have posed a difficulty for our results. Moreover, we encountered an improvement on ME, after 25(OH)D supplementation, independent to changes on HbA1c. This result may indicate a short-term anti-inflammatory effect of 25(OH)D on macular edema. To confirm our findings, double blinded, randomized clinical trials should be conclusively assess the effects of 25(OH)D on the ME of patients with T1DM.

### P79 Effect of nutritional intervention on elderly people knowledge of diabetes

#### Glenda Figueiredo Reis, Ricardo Barsaglini da Silva Leite, Helem Sena Ribeiro

##### Ensino e Pesquisa Santa Casa de Belo Horizonte, Belo Horizonte, Brazil

*Diabetology & Metabolic Syndrome* 2019, **11(Suppl 1):**P79

**Introduction:** Education in diabetes is one of the strategies to reduce the prevalence of complications and to promote self-care and management of the disease by the patient. The main purpose of this study was to evaluate the effects of the nutritional education in groups of elderly with Diabetes Mellitus Type II (DM2).

**Methods:** The intervention was performed collectively in a Medical Specialties Center from a hospital in Belo Horizonte. The sample calculation (5% significance level and 80% sample power) included all the ambulatory elderly patients (n = 88) who agreed on signing the TCLE. These patients were evaluated before and after nutritional interventions. First, they were presented to the new food pyramid. On a second moment, there was an approach about myths and truths supported by the SBD Guidelines through an interactive dynamic. Social demographic and clinical data were collected and a validated questionnaire was applied (Brazilian Version of the Questionnaire about the knowledge scale on Diabetes) in the beginning of the study and also after 3 months, in order to evaluate the participants’ knowledge through the number of right answers. The Wilcoxon and McNemar statistical tests were used. Outcome: 27 individuals were evaluated (18 women and 9 men) with an average age of 66.8 ± 5 years old. Most of the participants had completed Elementary school, were married and retired. The biggest part of them (66.7%) had a monthly income of up to two minimum wages. Almost half of the participants reported use of oral hypoglycemiant; all of them used insulin e 88.9% presented related diseases. More than 90% had been diagnosed with diabetes for more than 5 years ago. There was a statistical difference of the knowledge (pre *versus* post-intervention) related to the normal range of glucose in the blood (p = 0.031), the self-administration of insulin (p = 0.039), the awareness about what a kilo-unit weight is, the equivalent replacement of foods (p < 0.001) and the carbs replacement (p = 0.035). After grouping the questions by subjects similarity, there was also a statistical difference related to the general knowledge about diabetes (p = 0.003), treatment (p = 0.008) and nutritional care (p < 0.001).

**Conclusion:** The effect of the nutritional education in the elderly with DM2 was efficient, there was a significant improvement in knowledge after the intervention, reinforcing the importance of the awareness about diabetes in this population.

### P80 Effect of SGLT-2 inhibitors on glycemic control: real life data at an outpatient endocrinology clinic

#### Breno Carvalho Cirne de Simas^1^, Marcel Catão Ferreira dos Santos^1^, Lúcia Helena Coelho Nóbrega^1^, Marina Gabinio de Araújo Pontes^3^, Gabriel Guedes da Cunha^1^, Beatriz Andrade Brandão^2^, Luan Samy Xavier Dantas^1^, Josivan Gomes de Lima^1^

##### ^1^Universidade Federal do Rio Grande do Norte/Hospital Universitário Onofre Lopes, Natal, Brazil; ^2^Universidade Potiguar; ^3^Universidade Federal de Pernambuco, Recife, Brazil

*Diabetology & Metabolic Syndrome* 2019, **11(Suppl 1):**P80

**Introduction:** Among new agents approved for the treatment of type 2 diabetes (T2D), Sodium-Glucose Cotransporter 2 inhibitors (SGLT2i) are now considered the standard of care given their potential for cardiovascular and renal benefit. Their effect on glycemic control was modest in clinical trials. Whether they perform similarly in real-life is unclear.

**Objective:** To describe the effect of SGLT2i empagliflozin and dapagliflozin in real life settings.

**Methods:** We retrospectively searched clinical data from a cohort of patients with T2D assisted at an outpatient endocrinology clinic. Patients would be included if they initiated either empagliflozin or dapagliflozin after a registered visit and had follow-up data including at least Fasting Plasma Glucose (FPG) and glycated hemoglobin (A1c). The final date of follow-up was either the date with the lowest A1c after and SGLT2i was initiated or the date a new antidiabetic drug or insulin was prescribed. We assessed whether there were effects on A1c, Systolic Blood Pressure (SBP), LDL-c, and body weight. Student’s T test or Mann-Whitnney test were performed to calculate p-values if variables were parametric or non-parametric, respectively. We stratified patients by A1c at baseline < 7%, 7–8%, 8–9% and > 9%, and performed Kruskal–Wallis test to assess whether A1c reduction was increased in those with higher baseline A1c.

**Results:** Our final sample included 92 patients (57.6% female), 80.4% were on dapagliflozin, and mean age was 62.6 ± 8.8 years. At baseline, mean FPG was 130.2 ± 30.0 mg/dL, body weight was 80.5 ± 13.6 kg, LDL-c was 99.4 ± 36.8 mg/dL, median A1c was 7.1% (5.4–10.9), median SBP was 120 mmHg (95–160). Median follow-up was 20.5 weeks. There was a statistically significant A1c reduction (median reduction 0.3%, p < 0.001). Median reduction on A1c was 0.2% in the lower strata, 0.4% in the middle-lower strata, 1.0% in the upper-middle strata and 1.0% in the higher strata (p < 0.001), and did not differ according to drug prescribed (p = 0.992). SBP was significantly reduced (median reduction of 9.00 mmHg, p < 0.001). We observed a mean reduction of 2.58 kg (95% CI 2.06–3.10, p < 0.001) in bodyweight. Also, LDL-c was slightly increased (median increase of 11.4 mg/dL, p < 0.001).

**Conclusions:** Dapagliflozin and empagliflozin modestly improved glycemic control and other metabolic parameters. Improvement seems to be greater in those with poor control.

**Financial support:** None.

### P81 Effectiveness of a physical effortless test to predict maximal oxygen uptake in individuals with type 2 diabetes mellitus

#### Denise Maria Martins Vancea^2^, Claudio Barnabé dos Santos Cavalcanti^2^, Jonathan Nícolas dos Santos Ribeiro^3^, Thiago Borges Madureira Sabino^1^, Nathalia de Souza Melo Abath Barbosa^2^, Ricardo Azevedo Cavalcanti Aragão^2^, Theo Victor Alves Soares do Rêgo^2,^ Raphael Castro de Lima Ramos^2^

##### ^1^Universidade Federal de Pernambuco - UFPE, Recife, Brazil; ^2^Universidade de Pernambuco - UPE, Recife, Brazil; ^3^Instituto de Ciências Biológicas - UPE, Recife, Brazil

*Diabetology & Metabolic Syndrome* 2019, **11(Suppl 1):**P81

**Introduction:** Maximal oxygen uptake (VO2 max) is a valid clinical measure of great importance in assessing the cardiorespiratory system of individuals with type 2 diabetes mellitus (DM2). Poor cardiorespiratory fitness is a predictor of cardiovascular disease, morbidity and all-cause mortality in this population. The diabetics, in addition to reduced cardiorespiratory fitness, also present other chronic complications, such as autonomic and peripheral neuropathies, which contraindicate the performance of tests with physical effort. However, few studies have compared different prediction models, with and without physical effort, to evaluate the VO2 max of individuals with T2DM.

**Objective:** To verify the effectiveness of a test without physical exertion to predict the maximal oxygen uptake of individuals with DM2.

**Methods:** This research was characterized as descriptive. Ten trained type 2 diabetic women, with a mean age of 63.5 years ± 10.4 years, members of a supervised exercise program for diabetics at a Northeastern Public University, participated in this study. Two indirect tests were used for VO2 max prediction, the Rockport Fitness Walking and the N-EX NASA/Johnson Space Center from the University of Houston. The Rockport Fitness Walking test was performed on an athletic track. The diabetics women walked 1,609 meters in the morning. The N-EX NASA/Johnson Space Center test was applied by the researcher before the Rockport Fitness Walking test. Statistical analysis used multiple comparisons using Fisher’s smallest difference method and the Wilcoxon nonparametric test, being considered p ≤ 0.05. The study was approved by the institutional ethics committee nº 34868714.4.0000.5207.

**Results:** There was no significant difference between the N-EX NASA/Johnson Space Center and the Rockport Fitness Walking tests to predict the maximal oxygen uptake of the participants in this study (VO2 max = 21 ± 8.2 ml/kg/min versus 16.4 ± 6.2 ml/kg/min; p ≤ 0.27).

**Conclusions:** The N-EX NASA/Johnson Space Center test was effective for predicting maximal oxygen uptake without physical exertion for diabetics in this sample and can be a useful tool for diabetics contraindicated to exercise testing.

### P82 Effectiveness of insulinapp protocol in a university hospital in São Paulo - Brazil

#### Toyoshima MTK^1^, Moraes JRA^2^, Kondo RH^1^, Brandes PHR^1^, Paiva EF^1^

##### ^1^Serviço de Medicina Hospitalar, Hospital das Clinicas, Faculdade de Medicina da USP, São Paulo, Brazil; ^2^Serviço de Endocrinologia e Metabologia, Hospital das Clinicas, Faculdade de Medicina da USP, São Paulo, Brazil

*Diabetology & Metabolic Syndrome* 2019, **11(Suppl 1):**P82

**Introduction:** Hyperglycemia in hospitalized patients is associated with poor clinical outcomes, which includes increase on length of stay, infection risk, ICU admission and mortality. Despite its importance, adherence to protocols for management of inpatient hyperglycemia (IH) is still limited. Complexity of insulin protocols, clinical inertia and fear of hypoglycemia are main obstacles for optimal blood glucose (BG) control. Implementation and adherence to IH protocol is an indicator of hospital service quality. Thus, InsulinAPP is a computer-based protocol system that was developed to assist in managing IH by standardizing and simplifying subcutaneous insulin orders, particularly among healthcare providers who are not used to IH management. Most in-hospital glycemic control studies have used insulin analogues (glargine, detemir, lispro, aspart, glulisine). Few studies compared insulin analogues with human insulin (NPH and Regular insulin) in hospital setting and they have been shown equivalent. Human insulins are cheaper and better suited to the Brazilian public health system. We aimed to evaluate effectiveness of the InsulinAPP protocol in a public hospital in Brazil.

**Methods:** Retrospective medical record review was performed and approved by local ethics committee. 100 DM patients were admitted to the Hospital Medicine ward (November 2018–July 2019) and received either bolus + correction, basal-bolus or basal-plus insulin regimen with NPH and R insulin, according to InsulinAPP protocol. Data such as average BG, frequency of hypo and hyperglycemia during the first 10 days of protocol were collected. Descriptive statistical analysis was conducted.

**Results:** We evaluated medical records of 100 patients (60% men) with previous DM (92 previously known and 8 newly diagnosed DM), mean age 60.7 ± 18.6 years and mean A1C 8.5 ± 2.6%. 2275 BG were evaluated, 72% ranging 70–179 mg/dL, 42 hypoglycemia (< 2%) and 596 (26%) hyperglycemia (BG ± 180 mg/dL). Mean BG before the protocol was 202.7 ± 79.9 mg/dL (median 194 mg/dL) and dropped to 160.6 ± 57.5 mg/dL (median 152.2 mg/dL) on the first day. Mean BG remained falling in the first 3 days and was stable thereafter, with 144.5 ± 42.5 mg/dL (median 133.7 mg/dL) on the tenth day. Mean BG curve over time in our analysis was similar to studies with insulin analogues.

**Conclusions:** InsulinAPP protocol using human insulin was effective and safe for in-hospital glycemic control with significant improvement in BG mean and low hypoglycemia rate. Hospital glycemic control with significant improvement in BG mean and low hypoglycemia rate.

### P83 Effects of antioxidant treatment on renal morphology of diabetic rats

#### Hilary Barros de Carvalho, Natan Reyges Castro da Purificação, Melyna Soares de Souto, Lizandra Gabriela da Silva Leite, Edson Rafael de Sousa Araújo, Marcelo José Santiago Lisboa, Ohara Tereza da Silva Pacheco, Naianne Kelly Clebis

##### Universidade Federal do Rio Grande do Norte - Laboratório de microscopia celular e Tecidual/DMOR, Natal, Brazil

*Diabetology & Metabolic Syndrome* 2019, **11(Suppl 1):**P83

**Introduction:** Diabetes mellitus (DM) causes increased oxidative stress, endothelial cell proliferation with decreased capillary blood flow (ischemia) and increased the lipid peroxidation that is associated with renal dysfunction. Antioxidant use can balance the redox system by minimizing DM damages.

**Objective:** This research aimed to evaluate the morphological and quantitative changes in the diabetic kidney as well as the effect of antioxidant treatment on these parameters.

**Methodology:** Twenty male rats (n = 5) were divided into normoglycemic and diabetic groups without treatment (N and D) and treated with antioxidants (NT and DT). For induction of DM, 35 mg/kg body weight of streptozotocin was used. The experimental therapy was composed by quercetin (100 mg/kg body weight), l-glutamine 1% and E vitamin 1% administered by gavage for 60 days. Body weight, water and food consumption, final glycemia, glomerular density and glomerular morphometry were analyzed in 3 µm-thick sections stained with SPA (Schiff Periodic Acid). Fisher’s test was used for statistical analysis (p < 0.05).

**Results:** DM caused a reduction in body weight in animals D and DT compared to others, although there was an increase in water and food consumption in these animals, as well as plasmatic glycaemia level, which was not reversed by antioxidant treatment. Glomerular density (glomeruli/mm^2^) was similar between all groups. No differences in glomerular and tuft area (μm^2^) were identified in the analyzed groups. However, there was an increase in glomerular capsule thickness (μm) in NT compared to N, as well as an increase in urinary space (μm2) in D and NT compared to N, and a reduction in urinary space in DT group compared to the other groups.

**Conclusions:** The experimental model of DM did not lead to glomerular loss and morphometric changes suggestive of glomerulosclerosis. Morphometric changes were observed just in the urinary space. Thus, new models of induced DM and treatment should be tested to elucidate an action of this pathology in the kidneys.

**Keywords:** Diabetic nephropathies; Antioxidant therapy; Kidney; Histology.

### P84 Effects of cardiovascular biofeedback training in glycemic control and mental health on type 2 diabetes pacients

#### Estela Batista Santos, Tainá Maria de Souza Vidal, Victor Franklyn de Oliveira, Silvia Regina Arruda de Moraes

##### Universidade Federal de Pernambuco, Recife, Brazil

*Diabetology & Metabolic Syndrome* 2019, **11(Suppl 1):**P84

**Introduction and objectives:** Some factors such as anxiety, depression and stress levels may lead to a mis control of diabetes *mellitus 2* (T2D) causing complications that induce a decrease in the quality of life of individuals with this disease. The objective of this study was to evaluate whether a training using a cardiovascular *biofeedback* software would influence the control of levels of anxiety, depression, stress and glycemic control, in addition to the quality of life of Individuals with T2D.

**Methods:** It’s a series of 9 cases being 5 of them women and 4 others men submitted to a training with cardiovascular *biofeedback* (CARDIOEMOTION; developed by NPT Neuropsicotronics LTDA) for 8 Weeks, 3 times a week with 50-minute sessions. All volunteers were sedentary, T2D for more than 3 years, with an average age of 59.220 ± 7.04 years. The individuals answered to Beck’s depression and anxiety inventories, LIPP stress symptoms and the quality of Life Questionnaire (DQOL) before and after the training. Blood tests for the analysis of glycosylated hemoglobin was also performed before and at the end of the training. The present study was approved by the Ethics Committee on Research with Human Beings of UFPE (CAAE: 72119317.7.0000.5208).

**Results:** The participants obtained statistically significant difference in glycemic control (9.42 ± 2.99 × 7.86 ± 2.48 with P = 0.035). There were no statistically significant differences in the analysis of the inventories and questionnaire. However, reductions in the percentages of the sample from 33.3% to 11.1% were observed regarding the classification of the mild level of anxiety and 11.1% to 0% for the classifications of mild and severe depression levels. Regarding the LIPP scale analysis, a decrease in stress incidence was verified in the resistance phase from 66.6% to 33.3% of the sample studied. A reduction in the scores of the DQOL in 11.1% of the population studied was also observed, which is related to a better perception of quality of life.

**Conclusion:** Training with *Biofeedback* cardiovascular showed a positive effect on glycemic control and it seems to suggest that there are benefits on some aspects of mental health, such as anxiety symptoms, depression, stress and an increase in the level of quality of life of individuals with diabetes mellitustype 2.

### P85 Effects of creatine supplementation on the redox state of diabetic rats renal tissue, induced for estreptozotocina

#### Meline Gomes Gonçalves, Matheus Anselmo Medeiros, João Paulo Matos Santos Lima

##### Universidade Federal do Rio Grande do Norte, Natal, Brazil

*Diabetology & Metabolic Syndrome* 2019, **11(Suppl 1):**P85

**Introduction:** Creatine is a nutritional supplement that has been aim of research in various pathologies, with wide possibility of clinical applications. Studies showed the effect of creatine on increasing glucose uptake by elevating GLUT-4 receptor expression in metabolic disorders such as Type II Diabetes Mellitus, however, there are no studies reporting the effect of creatine on glycemic metabolism of Type I diabetes.

**Aim:** Therefore, the aim of this study is evaluate the effect of creatine supplementation in diabetic rats induced by streptozotocin (STZ).

**Methods:** 32 *Wistar* rats were separated into 4 groups: (C) animals without diabetes and without creatine supplementation (n = 8), (CCr) animals without diabetes and with creatine supplementation (n = 8), (D) diabetic animals induced with STZ without supplementation (n = 8), (DCr) creatine-treated STZ-induced diabetic animals (n = 8). The following evaluations were performed: clinical, biochemical, as well as the analysis of redox state parameters in the renal tissue of the animals. The experimental protocol was approved by the Animal Use Ethics Committee of the Federal University of Rio Grande do Norte, number 030.025/2017.

**Results:** In fasting glucose, diabetic groups showed significant hyperglycemia compared to control groups, but the DCr group showed a significant improvement over group D. In the quantification of urea and alanine transaminase, diabetic groups showed a significant increase. Compared to the control groups, however, the DCr group showed a significant improvement over group D in both parameters. The enzymatic activity of catalase, superoxide dismutase and glutathione peroxidase was significantly low in group D compared to the control groups, but the DCr group had stabilized activity similar to the control groups in all enzymes. In the quantification of hydrogen peroxide content, group D showed significantly higher levels compared to the control groups, but the DCr group had stabilization similar to the control groups in this parameter.

**Conclusions:** Creatine may improve the metabolic profile in DM and stabilize redox state parameters in this condition. These results support creatine supplementation as a potential therapeutic adjunct agent.

**Financial support:** CAPES & CNPq.

### P86 Effects of mesenquimal stem cell infusion on the skeletal muscle in diabetes mellitus model

#### Genoveva L. F. Luna, Stella Maris Firmino, Yisel C. Estrada-Bonilla, Meliza G Roscani, Fernanda F. Aníbal, Angela M. O. Leal

##### Universidade Federal de São Carlos- Ufscar, São Carlos, Brazil

*Diabetology & Metabolic Syndrome* 2019, **11(Suppl 1):**P86

**Introduction:** Diabetes mellitus (DM) is a major public health problem due to the high prevalence, and diabetic neuropathy (DN) is the most common chronic complication. Hyperglycemia damages neurons, leading to a sensorimotor disorder. The stem cell therapy is an attractive strategy for various complications of diabetes, among them ND, since they have the capacity to differentiate into other cell types and tissues, including muscle, and the ability to modulate the neurotropic factors to the nerves. However, mechanistically, this process is poorly understood.

**Objectives:** The present study aimed to evaluate the effects of Mesenchymal Stromal Cell (MSCs) transplantation on motor function and collagen organization in type 1 diabetes mellitus rat skeletal muscles.

**Methods:** Male Wistar rats were randomly assigned to 3 groups: Control (C), Diabetic (DM) and Diabetic treated with MSCs (DM-MSCs). Diabetes was induced by Streptozotocin (50 µg/kg). The MSCs were isolated from tibia and femur bone marrows. Ten weeks after DM induction, DM-MSC group received 4 weeklyi.p. injections of MSCs (1 × 106). Ten weeks after MSC transplantation, motor performance was evaluated by the Rota Rod test and the anterior tibial (TA) muscles were collected for morphometric analysis and quantification of collagen by birefringence by polarizing microscopy analysis. This study was approved by Brazilian ethical Committee.

**Results:** Motor performance of the DM group was significantly reduced when compared to the C group and increased significantly in the DM + MSCs group. The TA muscle mass and the muscle fiber cross sectional area were significantly reduced in the DM and DM + MSCs groups compared to the C group. The connective tissue increased in the DM group compared to the C group and decreased in the DM + MSCs group. The percentage of collagen birefringence of the connective tissue of the TA muscle decreased significantly in the DM group when compared to the C group and increased in the DM + MSCs group. Motor performance was positively correlated with collagen birefringence and negatively correlated with percentage of connective tissue.

**Conclusions:** The results indicate that MSCs transplantation improves both motor function and the collagen macromolecular organization of skeletal muscle in type 1 DM.

**Financial support**: CAPES, PPGBIOTEC-UFSCAR.

### P87 Effects of vitamin d supplementation among individuals with metabolic syndrome: a pilot study

#### Séphora Louyse Silva de Aquino^1^, Aline Tuane Oliveira da Cunha^2^, Severina Carla Vieira Cunha Lima^1^, Karine Cavalcanti Maurício Sena-Evangelista^1^, Josivan Gomes Lima^1^, Lucia Fatima Campos Pedrosa^1^

##### ^1^Universidade Federal do Rio Grande do Norte, Natal, Brazil; ^2^Universidade Potiguar, Natal, Brazil

*Diabetology & Metabolic Syndrome* 2019, **11(Suppl 1):**P87

**Introduction:** Vitamin D deficiency has been associated negatively with components of metabolic syndrome (MetS), such as fasting glycemia, arterial hypertension, and triglycerides. Nevertheless, it is uncertain whether supplementation with vitamin D is responsive to improving components of MetS because of controversial results.

**Objective:** To evaluate the effects of vitamin D supplementation in individuals with MetS.

**Methods:** In this pilot study, seventeen middle-aged and older adults with MetS and vitamin D deficiency [25-hydroxyvitamin D (25OHD) < 20 ng/mL] received vitamin D3 oral supplementation for 5 months. Firstly, the individuals received a loading dose of 50 000 IU once a week for 8 weeks (t_1_), followed by a maintenance dose of 7 000 IU once a week for 12 weeks (t_2_). We measured serum concentrations of 25(OH)D, clinical, biochemical, nutritional, and sun exposure parameters at baseline (t_0_) and the end of each period (t_1;_ t_2_). Serum concentrations of 25(OH)D were assessed using the chemiluminescent assay. Man-Whitney test was used for statistical testing significance. Continuous variables were expressed as median (interquartile interval). The local Research Ethics Committee (CAAE n. 13699913.7.0000.5292) approved the study.

**Results:** 25(OH)D concentrations (ng/mL) increased significantly (t_0_ vs. t_2_ p < 0.0001); median t_0_ = 19.0 (18.5; 19.4); t_2_ = 36.5 (27.15; 46.4). Additionally, there were significant decrease in PTH (pg/mL) (t_0_ vs. t_2_; p = 0.043); median t_0_ = 56.7 (55.4; 58.1); t_2_ = 43.9 (32.4; 56.9) and albumin-corrected calcium concentrations (mg/dL) (t_0_ vs. t_2_; p = 0.012); median t_0_ = 7.4 (7.1; 7.8); t_2_ = 7.1 (6.9; 7.2). The sun exposure score was significantly higher only when compared between t_0_ and t_1_ (p = 0.024); median t_0_ = 16.0 (8.0; 19.0); t_1_ = 22.6 (14.0; 29.5). No significant differences in changes of triglycerides, HDL-c, fasting glycemia, waist circumference, blood pressure, and vitamin D intake among individuals were observed during the evaluated periods (p value  >  0.05).

**Conclusions:** Weekly vitamin D3 supplementation for 5 months significantly increased 25(OH)D concentrations; however, no improvement in MetS components was observed.

**Financial support:** National Council for Scientific and Technological Development (CNPq), Brazil; grant no. 471761/2013-3.

### P88 efficacy and safety of semaglutide 0.5 MG vs dulaglutide 1.5 MG once weekly in type 2 diabetes: a post hoc analysis of sustain 7

#### Victor França De Almeida^1^, Richard E. Pratley^2^, Vanita R. Aroda^3^, Theis Gondolf^4^, Thomas Hansen^4^, IldikoLingvay5^5^, Jörg Lüdemann^6^, Trine Vang Skjøth^4^

##### ^1^Novo Nordisk Ltda, São Paulo, Brazil; ^2^AdventHealth Orlando, Translational Research Institute for Metabolism and Diabetes, Orlando, USA; ^3^Brigham and Women’s Hospital, Boston, USA; ^4^Novo Nordisk A/S, Søborg, Denmark; ^5^University of Texas Southwestern Medical Center, Dallas, USA; ^6^Diabetes-falkensee, Diabetes Centre and Centre for Clinical Studies, Falkensee, Germany

*Diabetology & Metabolic Syndrome* 2019, **11(Suppl 1):**P88

**Introduction:** Semaglutide and dulaglutide are glucagon-like peptide-1 receptor agonists for the treatment of type 2 diabetes (T2D). In SUSTAIN 7, an international, open-label, parallel group trial, adults with inadequately controlled T2D were randomized (1:1:1:1) to once-weekly subcutaneous semaglutide or dulaglutide at low (0.5 vs. 0.75 mg) or high (1.0 vs. 1.5 mg) doses. Semaglutide provided superior glycemic control and reductions in body weight at both low and high doses.

**Objective:** to compare the effects of semaglutide low (0.5 mg) vs dulaglutide high (1.5 mg) dose at week 40 and this comparison was implemented to reflect options available in clinical practice and to ensure a thorough assessment of clinical efficacy and safety.

**Methods:** A post hoc analysis of the efects of semaglutide low (0.5 mg) vs. dulaglutide high (1.5 mg) dose at week 40, using statistical methods in published prespecied analyses. Subjects mean age was 56 years, HbA 8.2%, diabetes duration 7.4 years; 77% were white.

**Results:** Eficacy data show similar glycemic control and blood pressure, and greater weight loss, for semaglutide 0.5 mg vs. dulaglutide 1.5 mg. Frequency and severity of adverse events (AEs) were similar for semaglutide and dulaglutide, including gastrointestinal (GI) AEs (nausea: 23% vs. 20%, respectively; vomiting: 10%, each treatment). Premature discontinuation due to GI AEs occurred in 5% of subjects in each arm.

**Conclusion:** Semaglutide 0.5 mg showed similar improvements in glycemic control and greater weight loss vs. dulaglutide 1.5 mg at week 40, while showing similar tolerability in subjects with T2D.

### P89 Efficacy of ideglira vs basal-bolus therapy in subjects with type 2 diabetes in dual vii by baseline characteristics

#### Tatiana Martins Benvenuto Louro Berbara^1^, Liana K. Billings^2^, Yuksel Altuntas^3^, David C. Klonoff^4^, Nikolaos Tentolouris^5^, Randi Gron^6^, Natalie Halladin^6^, Esteban Jódar^7^

##### ^1^Novo Nordisk Brasil Ltda, São Paulo, Brazil; ^2^NorthShore University HealthSystem/University of Chicago, Evanston, USA; ^3^University of Health Science, Şişli Hamidiye Etfal Training and Research Hospital, Istanbul, Turkey; ^4^Mills-Peninsula Medical Center, San Mateo, USA; ^5^National and Kapodistrian University of Athens, Medical School, Athens, Greece; ^6^Novo Nordisk A/S, Soborg, Denmark; ^7^University Hospital Quirónsalud, Madrid, Spain

*Diabetology & Metabolic Syndrome* 2019, **11(Suppl 1):**P89

**Introduction:** In 506 subjects with type 2 diabetes uncontrolled on metformin and basal insulin, the DUAL VII trial (NCT02420262) demonstrated that a once-daily injection of insulin degludec/liraglutide (IDegLira) was non-inferior to multiple injections of basal–bolus therapy (insulin glargine 100 units/mL + insulin aspart before each main meal) for A1C reduction, and was associated with a significantly lower risk of hypoglycemia.

**Methods:** This non-prespecified post hoc analysis examined whether the efficacy results persisted across sub-groups for six different variables and evaluated whether benefits seen in the trial were applicable across a broad patient population.

**Results:** After 26 weeks of treatment, there was no significant difference in A1C reduction between IDegLira compared with basal–bolus in any baseline characteristic group (A1C, BMI, age, diabetes duration, total daily insulin dose and fasting plasma glucose). Additionally, there was no significant interaction between treatment and baseline sub-group for any of the baseline characteristics.

**Conclusions:** In conclusion, the A1C lowering was comparable between once-daily IDegLira vs. basal– bolus therapy after 26 weeks of treatment, irrespective of baseline characteristics, which underscores the general application of these findings to a broad patient population.

### P90 Efficacy of semaglutide vs dulaglutide across baseline HBA1C in sustain 7

#### Raquel Cristina Lopes Assis Coelho^1^, Richard E. Pratley^2^, Juan P. Frias^3^, Harish Kumar^4^, John Petrie^5^, Andrea Navarria^6^, Morten Abildlund Nielsen^7^, Wolfgang E. Schmidt^8^

##### ^1^Novo Nordisk Farmacêutica do Brasil, São Paulo, Brazil; ^2^Florida Hospital Translational Research Institute, Orlando, USA; ^3^National Research Institute, Los Angeles, USA; ^4^Centre for Endocrinology and Diabetes, Amrita Vishwa Vidyapeetham, Kochi, India; ^5^Institute of Cardiovascular and Medical Sciences, University of Glasgow, Glasgow, Scotland; ^6^Novo Nordisk A/S, Søborg, Denmark; ^7^Novo Nordisk A/S, Aalborg, Denmark; ^8^Department of Medicine I, St. Josef-Hospital, Ruhr-University Bochum, Bochum, Germany

*Diabetology & Metabolic Syndrome* 2019, **11(Suppl 1):**P90

**Introduction:** Semaglutide, a new GLP-1 analog for T2D, showed significant and clinically meaningful HbA1c and body weight (BW) reductions across the SUSTAIN clinical trial program.

**Methods:** This post hoc analysis of the phase 3b SUSTAIN 7 trial evaluated once-weekly subcutaneous semaglutide 0.5 mg vs dulaglutide 0.75 mg and semaglutide 1.0 mg vs dulaglutide 1.5 mg by baseline (BL) HbA1c subgroups in subjects with T2D.

**Results:** At week 40, improvements in HbA1c and BW were similar or favored semaglutide vs dulaglutide across subgroups (p-value for interaction: HbA1c, p < 0.03; BW, p > 0.05); estimated treatment effects were similar or favored semaglutide. More subjects with BL HbA1c > 9% achieved HbA1c targets with semaglutide vs dulaglutide.

**Conclusions**: Semaglutide was associated with similar or greater HbA1c and BW reductions vs dulaglutide in all subjects regardless of BL HbA1c.

**Keywords:** Semaglutide; Type 2 diabetes.

### P91 Elaboration and cultural adequacy of the instrument “impact of the diagnosis of type 1 diabetes in the family environment of children and adolescents”

#### Márcia Salomão Ramos, Lívia Pinto e Fróes, Sônia Maria do Carmo Maulais, Janice Sepúlveda Reis

##### Santa Casa de Belo Horizonte, Belo Horizonte, Brazil

*Diabetology & Metabolic Syndrome* 2019, **11(Suppl 1):**P91

**Introduction and goals:** Diabetes type 1 (DM1) in childhood and adolescence impacts the family’s routine. Children’s chronic illness can trigger several family conflicts: job abandonment, financial imbalance, reduction or interruption of leisure time, overload of the primary caregiver and disruption that affects the whole family.

There are no elaborated and culturally adapted tools to evaluate this kind of impact in the family daily life, which could also help the health professionals to direct educational actions on diabetes. The aim of this study was to create and culturally adapt an instrument to assess the impact of the diagnosis of DM1 in the family environment of children and adolescents.

**Method:** Methodological study, consisting of three stages: 1) Preparation of the instrument, based on national and international guidelines of diabetes and bibliographic review on the subject. Shortly after, evaluation by a committee of specialists, consisting of endocrinologist, psychologist and linguist, originating the first version (V1); 2) The V1 was sent by the platform e-surv to a committee of judges, made up of professionals with experience in attending families of children and adolescents with DM1, who evaluated the clarity and relevance of the items. Based on the evaluation, the content validity index (CVI) was calculated. The suggestions of the judges’ which were considered relevant to the adequacy of the components of the instruments were accepted, originating the second version (V2). 3) Face-to-face test with the families of children and adolescents with DM1 (Version 3 – V3).

**Results:** The V1, according to the evaluation of the committee of judges, obtained a high score in relation to clarity and relevance, with a CVI of 0.97. The application of the face-to-face tests showed that only two questions presented difficulty in understanding. After the adjustments were done to this application, the V3, formed by 18 questions (final instrument), was considered suitable to the target audience.

**Conclusion:** The instrument was considered culturally appropriate to evaluate the impact of the diagnosis of DM1 in the family environment of children and adolescents.

**Keywords:** Diabetes Mellitus Type 1; Family; Surveys and questionnaires

### P92 Elaboration and validation of educational material for foot care in people with diabetes

#### Ângelo Gustavo Venâncio De Lima, Alexandra Dias Moreira, Tatiane Gea Horta, Rosimeire Fernandes de Oliveira, AgmaLeozina Viana, Suelem Rosa de Oliveira, Maria Regina Calsolari Pereira de Souza, Janice Sepúlveda Reis

##### Ensino e Pesquisa da Santa Casa de Belo Horizonte, Belo Horizonte, Brazil

*Diabetology & Metabolic Syndrome* 2019, **11(Suppl 1):**P92

**Introduction:** Diabetic foot, one of the most feared complications of diabetes mellitus (DM), is responsible for prolonged hospitalizations, lower limb amputations, disability, early retirement and preventable deaths. In the context of diabetes education, the use of printed educational materials is common practice in the Public Health System, with reading, discussion, and training of skills used as additional tools in individual and/or group teaching. The contribution of these materials to health promotion depends on the forms of communication involved in the elaboration processes.

**Objective:** Develop, adapt and validate an educational material for foot care in people with DM.

**Methods**: Methodological study developed in the following steps: definition of the theme and target audience, bibliographic survey, preparation of the material, readability analysis, knowledge and linguistic assimilation capacity, validation by committee (24 judges, with a minimum degree of specialist, including health and linguistic professionals), through the e-Surv online platform (with calculation Content Validity Index—CVI), discussion with experts and final validation by the target audience. This study was approved by The Local Research Ethics Committee.

**Results:** The educational material, after the analysis of the judges committee, the interdisciplinary team and the evaluation after application in target audience, was finished with 12 pages, containing written messages and illustrations. The material presented three versions, resulting in a Content Validity Index (ICV) of 0.95 for the final version. The target audience suggested, for a better understanding of the information, changes in 07 pictures, 05 written messages and the inclusion of a phrase with foot care message. The material was applied successively until there were no new suggestions for changes.

**Conclusion:** The educational material for foot care in people with diabetes is considered culturally appropriate and validated.

### P93 Elaboration and validation of practical guidelines on preparation and application of insulin

#### Stephanie Santos Siqueira^1^, Suelem Rosa de Oliveira^1^, Janice Sepúlveda Reis^1^, Alexandra Dias Moreira^2^, Heber Augusto Lara Cunha^3^, Tatiana Géa Horta Murta^1^

##### ^1^Instituto de Ensino e Pesquisa da Santa Casa de Belo Horizonte - IEP Santa Casa BH, Belo Horizonte, Brazil; ^2^Universidade Federal de Minas Gerais - UFMG; ^3^Faculdade IPEMED de Ciências Médicas, Belo Horizonte, Brazil

*Diabetology & Metabolic Syndrome* 2019, **11(Suppl 1):**P93

**Introduction:** Insulin use by wrong methods is unsafe and interferes in the achievement of good metabolic control, a fundamental requirement to protect against complications caused by Diabetes Mellitus (DM). While recognizing education as an essential part in the diabetes treatment, the instructions regarding preparation and the correct technique of insulin application are a key issue to be adressed. It is well known that educational materials, when well elaborated and validated, make possible the explanation of topics related to DM in a comprehensible way.

**Objective:** To develop, adapt and validate an educational material on the steps to be followed in the application of insulin through pens and syringes.

**Methods:** Methodological study developed in the following steps: definition of the theme and target audience, bibliographic survey, elaboration of the material, determination of readability and learnability, validation by committee of judges through the e-Surv online platform, discussion among experts, validation by the target audience and final discussion between experts. The Research Ethics Committee of the respective institution approved this study.

**Results:** The clarity of sentences was evaluated, and the educational material reporting insulin administration through the pens obtained an avarage CVC of 0.98 and the syringe material of 0.94. From the judges invited via e-Surv, six analyzed the pens material and eight the syringes material, suggesting textual and image adaptations. In the face-to-face test, the patients considered the material valid and useful, noting that 26 people evaluated the volume of the pens and 26 people the volume of syringes. All suggestions were discussed among experts and led to the creation of the final version of the material.

**Conclusions:** The material has been prepared, adapted and validated, and can be used to clarify doubts or to remind the routine of insulin application. It can also help to guide the health professional in the lack of instruments for demonstration.

### P94 Elaboration, adaptation and validation of a booklet about the demystification of the diabetes student diet

#### Stephanie Araújo Oliveira Rezende^1^, Débora Bonhen Guimarães^1^, Luiz Henrique Diniz Miranda^1^, Adriana Silvana Pagano^2^, Luciana Ferreira Valadares^1^, Tatiane Géa Horta Murta^1^, Janice Sepúlveda Reis^1^, Helem Sena Ribeiro^1^

##### ^1^Ensino e Pesquisa Santa Casa de Belo Horizonte, Belo Horizonte, Brazil; ^2^Faculdade de Letras da - FALE - UFMG, Belo Horizonte, Brazil

*Diabetology & Metabolic Syndrome* 2019, **11(Suppl 1):**P94

**Introduction:** Diet, including meals and snacks in the school environment, is a key part of treating children and adolescents with diabetes (DM). Family and school are responsible for the transmission and construction of knowledge; and strategies such as the educational materials make it possible to disseminate information to this population.

**Objective:** To describe the process of elaboration, adaptation and validation of a booklet on the demystification of the diet in schools of the students with DM.

**Methods:** Methodological study started with the definition of the theme and the target audience, and bibliographic survey. The preparation of the educational material was made from the determination of readability and comprehensibility (from the Flesch Readability, Flesch Kincaid, and the Coleman Liau Index formulas). In the next phase, the booklet was evaluated by a committee of judges through the e-Surv online platform and the subsequent calculation of the level of agreement by the Content Validity Coefficient (CVC). After the judges’ suggestion, a discussion was held between experts in the area and the educational material was validated by the target audience through face-to-face testing. The last phase was a final discussion among experts to accept or not the suggestions made. This study was approved by The Research Ethics Committee of the respective institution.

**Results:** The booklet obtained an average CVC of 0.91. Of the invited judges, 11 evaluated and provided suggestions that involved the removal and restructuring of text, as well as the insertions and exchange of images. In face-to-face tests, conducted with 12 participants, the booklet was evaluated positively. The suggestions were analyzed according to the relevant literature and discussed among experts. The final version has 38 two-sided pages, entitled: “Demystifying the Feeding of Students with Diabetes in Schools”, also containing an annex with the main laws that determine the distribution of school meals, to let students know their rights, and a detachable special meal request model.

**Conclusions:** The educational material was elaborated, adapted and validated from the point of view of content and relevance. Its use by teachers, school staff and other caregivers of children or adolescents with DM will contribute to the acquisition of knowledge and demystification of issues related to food in the school environment.

### P95 Employment of the baropodometer as a supporting in the detection of peripheral neuropathy in asympomatic individuals

#### Neidmar Da Mata, Rayanne Tojal De Carvalho, Kaline Karla Garcia De Araujo, Luana Régia Ribeiro De Araújo, Tahysa De Souza Costa, Maria Antonia Ferreira Gomes

##### UnP, Natal, Brazil

*Diabetology & Metabolic Syndrome* 2019, **11(Suppl 1):**P95

**Introduction:** Thebaropodometer is a tool used to assist in the study of body balance through the analysis of plantar force. Uses software to produce similar to podoscope images that print on graphics. This technique provides data of high diagnostic value, identifying early plantar changes that may early reflect chronic diseases. Neuropathy is known to be one of the diseases that contribute to plantar ulcers and 50% of the diabetic population has asymptomatic neuropathy. High plantar pressure with loss of protective sensation precedes ulcerations, resulting in serious complications and even leg amputations. Therefore, active search should always be performed early to prevent ulcerations.

**Objective:** To analyze the presence of peripheral neuropathy in asymptomatic individuals through neurological examination and the use of the baropodometer.

**Methods:** This is a quantitative cross-sectional study with a sample of 121 individuals of both sexes, aged between 20 and 60 years, evaluated from 2017 to 2018, at the Integrated Health Center of Potiguar University in Natal/RN. Having been approved by the ethics committee with CAAE 6605.9617.0.0000.5296. Data were collected using a semi-structured instrument and clinical observation, using pain, thermal sensitivity, vibration perception and aquileus reflex neurological tests. TSS (Total Symptom Score) and NDS (Neuropathy Disability Score) scores and numerical scales were used as diagnostic criteria to assess the frequency and intensity of symptoms. Tests were applied using monofilament and walking on the Electronic Baropodometer. Considering that silent loss of sensitivity is the major risk factor for foot injuries and the risk threshold is best identified by the absence of sensation using 5.07 monofilament, which gives a pressure of 10 g.

**Results:** In the neurological evaluation 25% of the individuals presented severe neuropathic symptoms, 43% without signs, 5.7% with moderate to severe signs and 30.57% reported feeling severe pain. Peripheral changes were of low significance, but will serve as a basis for reevaluation of these patients and identification of possible disease evolution.

**Conclusion:** With this research it was possible to identify the need for early revaluation of individuals, especially those with diabetes to identify the presence of peripheral neuropathy and its possible evolution.

### P96 Empowerment of healthcare professionals through a validated diabetes educational program

#### Claudia Mauricio Pieper^1^, Denise Franco^2^, Graça Camara^3^, Sonia Castilho^4^, Maristela Bassi Strufaldi^4^, Tarcila Ferraz Campos^4^, Maria Gabriela Cavicchioli^4^, Fernanda Castelo Branco^4^

##### ^1^Pós-Graduação em Endocrinologia da PUC-Rio, Rio de Janeiro, Brazil; ^2^Centro De Pesquisa Clínica (Cpclin), São Paulo, Brazil; ^3^Hospital Alemão Oswaldo Cruz (SP), São Paulo, Brazil; ^4^ADJ-Diabetes Brasil, São Paulo, Brazil

*Diabetology & Metabolic Syndrome* 2019, **11(Suppl 1):**P96

**Introduction:** Rappaport defines empowerment “as a process by which people gain mastery over their affairs”. In health education, this increase in power is not a means to dominate or change others, but rather a means to affect change. Empowering health care professional to affect change in diabetic patients can impact more than specific health behaviors.

**Objective:** To demonstrate the importance and validity of a Diabetes Education Program in the empowerment of health professionals in Brazil.

**Methods:** This Diabetes Education Program for Health Professionals was validated 11 years ago. It consists of theoretical and practical classes based on the PBL methodology, aiming at greater student participation and offering educational tools so that at the end of the 40 h, they are able to do a diabetes project aiming to help the patients they attend.

**Results:** 37 qualification courses were conducted in 26 Brazilian states, empowering health professionals from public (985) and private (679) units. A total of 1766 health professionals were trained: 685 (38.78%) nurses, 485 (27.46%) nutritionists, 185 (10.47%) doctors, 141 (7.98%) pharmacists, 90 (5.09%) psychologists, 43 (2.43%) physical education teachers and other health professionals, 135 (7.64%). The health professionals who participated in this Qualification Coursed did a pre and a post test on diabetes, with an 85% success rate at the end of the course. Satisfaction assessments were conducted on the program and course, which results were: 73% rated excellent, 25% Good, 2% regular and O % poor. A total of 957 projects were carried out by trained health professionals, many of them with good results in the patient population in which they were implemented according to the authors’ reports.

**Conclusions:** This qualification program has been demonstrating results consistent with its diabetes training goals of health professionals who took the course where it is used as a tool for empowerment. Empowerment is more than an intervention or strategy to help people make behavior changes to adhere to a treatment plan.

### P97 Endogenous modulation of diabetic neuropathic pain is exercise intensity dependent

#### William Valadares Campos Pereira, Douglas Lamounier de Almeida, Igor Dimitri Gama Duarte, Sérgio Henrique Sousa Santos, Thiago Roberto Lima Romero

##### Departamento de Farmacologia, Instituto de Ciências Biológicas, ICB-UFMG, Belo Horizonte, Brazil

*Diabetology & Metabolic Syndrome* 2019, **11(Suppl 1):**P97

**Introduction:** Opioid systems play an important role in endogenous modulation of diabetic neuropathic pain. Due to the lack of reports on exercise control variables and endogenous modulation in neuropathic diabetic pain, we investigated whether this modulation is dependent on exercise intensity.

**Objective:** The aim of our study was to evaluate if different physical exercise intensities can differently modulate the diabetic neuropathic pain.

**Methods:** The maximum progressive fatigue exercise protocol was used to measure: 80% and 40% of the maximum speed used in continuous exercise protocols. To combine different intensities of physical effort, we used the work calculation (τ), where the animals developed different speeds and times, but with the same final physical effort. The work calculation was performed in each exercise protocol, using the formula τ = F·Δs, to calculate forces parallel to the displacement, due to the fact that the protocols used in this work do not predict inclination (0% inclination). After an interval of 7 days, the animals were submitted to the above speeds and performed the exercise at continuous speed.

Naloxone was used immediately before protocols were performed at different intensities and the nociceptive threshold was measured when Naloxone peaked.

**Results:** In the control group, high intensity exercise induced higher analgesic response than moderate intensity exercise (p = 0.0325). When mice received 100 µg of naloxone, we observed a reduction in high-intensity exercise-induced analgesic response (p = 0.0061), but the naloxone dose was not sufficient to counteract the analgesic effect of the exercise protocol. In the neuropathic group, it was observed that high intensity exercise induced a significant increase in nociceptive threshold compared to moderate intensity exercise in saline-injected rats (p = 0.0001). When mice were injected with 100 µg naloxone, a reduction in exercise-induced high intensity nociceptive threshold was also observed, but the naloxone dose was not sufficient to counteract the analgesic effect of the exercise protocol (p = 0.0045). The same dose of Naloxone injected in mice undergoing continuous moderate exercise was able to completely oppose endogenous pain modulation.

**Conclusions:** Exercise intensity plays a key role in endogenous modulation of diabetic neuropathic pain and the opioid system has an important peripheral role in controlling the impulses ascending from the periphery.

### P98 Epidemiological profile of diabetic foot admissions by the Brazilian unified health system in the Northeast region from 2009 to 2018

#### Márcio Ribeiro Melhor^1^, Jair de Souza Braga Filho^1^, Victor Costa Araújo^2^, Rafael Rodrigues Oliva^3^, Ananda Pires Bastos^1^, Maria Luiza Nogueira de Barreiros Gavazza^1^, Gabriela Flor Martins^1^, Bianca Aparecida Colognese^1^

##### ^1^Universidade Federal da Bahia (UFBA), Salvador, Brazil; ^2^Universidade do Estado da Bahia (UNEB), Salvador, Brazil; ^3^Universidade Salvador (UNIFACS), Salvador, Brazil

*Diabetology & Metabolic Syndrome* 2019, **11(Suppl 1):**P98

**Introduction:** The diabetic foot is a chronic complication of diabetes mellitus (DM) whose diagnosis is mainly clinical, and therefore is easy to do and has low cost. However, it may present complications with negative outcomes, ranging from limb loss to death. Considering that about 25% of diabetics will be affected by the diabetic foot, a huge number of hospital beds is destined to these patients, possibly encumbering and congesting the healthcare system.

**Objectives:** To evaluate the epidemiological profile of diabetic foot admissions, as well as data such as the financial resources invested in the treatment of this complication and the mortality rates in the past 10 years.

**Methods:** This study performed an epidemiological analysis of the hospital admissions because of diabetic foot in the Northeast region, from January 2009 to December 2018. For this evaluation, secondary data from the section of “Hospital Information System (SIH/SUS)” were used. They were: number of admissions annually, mean number of days hospitalized, mortality, total cost and mean cost per patient.

**Results:** Altogether, 51.070 admissions were analyzed. These admissions were related to a total cost of R$ 28.648.416,80; mean cost per patient of R$ 550,58; 7.82 days of permanence hospitalized; 1961 deaths (mortality index of 3.84%). From 2009 to 2018, there was an increase of 309% in the total number of admissions (2011 to 8221), 380% in the total cost (R$ 1.045.040,63 to R$ R$ 5.017.368,36), 139% in the total of deaths (99 to 237) and 17,4% in the mean cost per patient (R$ 519,66 to R$ 610,31); and a reduction of 70% in mortality (4.92 deaths/year to 2.88) and 18% in mean days of permanence (9.2 to 7.8).

**Conclusion:** According to the results of this paper, we found a significant increase in the number of admissions, in the total cost of admissions and in the number of deaths, in the last 10 years, though a reduction in the annual mortality and mean number of days hospitalized was also observed. Thus, our health system should evaluate problems and advances related to these data and improve prevention and promote healthcare for this diabetic population, in order to avoid increases in the the number of admissions by diabetes itself and its implicated chronic complications.

### P99 Epidemiology and prevalence of diabetic retinopathy in a population assisted at world diabetes day in Presidente Prudente-SP

#### Ana Beatriz Foleto Brait^1^, Carolina de Castro Rocha Betônico^1^, Guilherme Pereira de Oliveira Matos^1^, Maria Eduarda Vilela Rodrigues da Cunha^1^, Ricardo Bernardes Filho^2^, Marcelo Hosoume^2^

##### ^1^Faculdades Adamantinenses Integradas, Adamantina, Brazil; ^2^Hospital Oftalmolaser, Presidente Prudente, Brazil

*Diabetology & Metabolic Syndrome* 2019, **11(Suppl 1):**P99

**Introduction:** Diabetic retinopathy (DR) is one of the most important microvascular complications of diabetes mellitus (DM), and is a leading cause of irreversible visual impairment in the world. Early detection and timely treatment of DR are imperative to prevent or delay diabetic related blindness.

**Objective:** To identify the prevalence of diabetic retinopathy in its different stages, and the presence of *maculopathy and* cataract of a DM population assisted at the World Diabetes Day Campaign (24th of November of 2018) in Presidente Prudente-SP and their epidemiological characteristics.

**Methodology:** Descriptive cross-sectional and population study was performed based on the collected data at diabetes campaign. The participants with DM were referred for routine eye examination, in addition to binocular indirect ophthalmoscopy to define the prevalence of maculopathy, cataract and DR classified as non-proliferative DR (NPDR) or proliferative DR (PDR) at its different stages.

**Results:** 943 participants fulfilled the inclusion criteria, 57.5% female, aged 18–91 years, with a predominance of 61–70 years (32.9%). 551 (58.4%) of the participants had DM and 439 (84.2%) of them *were referred to perform ophthalmologic exam at the campaign*. A quarter of these patients (25.5%) had their capillary blood glucose measured (mean 129 mg/dl ± 50.9) during the event. Among the patients referred to ophthalmologic exam, the majority (55.5%) had *never* undergone an *eye* examination for DR screening before, 69.2% had hypertension and 61.7% reported monitoring capillary blood glucose daily. Retinal examination found absence of DR in 72.2% (DR), 3.1% impossible to classify and the presence of DR in 24.6% classified as: 46.3% mild non-proliferative DR (RNP); 21.3% moderate DRNP; 17,5% severe DRNP; 3.7% proliferative retinopathy (PR) without previous laser treatment; 6.5% PR with signs of previous laser treatment and 4.6% had PR and vitreous hemorrhage. Eye evaluation revealed 7% of maculopathy, 28.9% had cataracts, mostly mild, and 53% had intraocular lenses.

**Conclusion:** Despite the elevated prevalence of DR, the study could demonstrate that a great number of people with DM had *never ever been* referred to an ophthalmologist for DR screening. Systematic screening for DR on campaigns is important to identify patients who need referral to a specialist and for designing, and evaluating public health prevention programs.

### P100 Evaluation of clinical and laboratory parameters of insulin resistance in adolescent girls with polycystic ovary syndrome

#### Isabel C. S. Bouzas, Daniela C. Bouzas, Samária A. Cader, Núbia Mattos Silva, Rebeca B. M. Cavalcante, Caroline A. G. Mello, Ana Beatriz W. Tavares, Lenora M. C. S. M. Leão

##### Universidade do Estado do Rio de Janeiro, Rio de Janeiro, Brazil

*Diabetology & Metabolic Syndrome* 2019, **11(Suppl 1):**P100

**Introduction:** Polycystic ovary syndrome (PCOS) is a complex disorder that encompasses a broad spectrum of reproductive and metabolic disorders, associated with elevated serum androgens levels and reduced insulin sensitivity. Metabolic disorders, such as obesity, dyslipidemia and glucose intolerance (GI) are quite frequent and may be instituted early, increasing the prevalence of type 2 diabetes mellitus (DM2) and metabolic syndrome (MS).

**Objective:** To compare clinical and laboratory parameters related to insulin resistance (IR) in Brazilian adolescents with and without PCOS.

**Methods:** Cross-sectional study involving 67 PCOS adolescents diagnosed according to *Rotterdam criteria* and 72 adolescents without irregular menstrual cycles and hyperandrogenism, all with gynecological age > 2 years. A *p* value < 0.05 was considered statistically significant.

**Results:** Age (15.3 ± 1.4 vs 15.3 ± 1.4 years) and body mass index (30.2 ± 8.3 vs 27.7 ± 7.7) were not statistically different, but waist circumference (95.5 ± 16.9 vs 89.4 ± 16 cm), diastolic blood pressure (77.5 ± 12.4 vs 73.2 ± 11.2 mmHg) and percentage of *acanthosis nigricans* (71.6% vs 48.6%) were statistically higher in the PCOS group. SHBG was statistically lower (31.7 ± 16.4 vs 51.6 ± 30 nmol/L), whereas total testosterone (389 ± 290 vs 238 ± 177.5 pg/mL) and androstenedione (2427.7 ± 1385.7 vs 2040.8 ± 1385.7 pg/mL) were statistically higher in PCOS adolescents. Fasting glucose (88.1 ± 8.7 vs 85 ± 7.9 mg/dL), fasting insulin (27.3 ± 19.6 vs 15.2 ± 9.2 µUI/mL), HOMA IR (6 ± 4.45 vs 3.15 ± 1.86), post-oral glucose tolerance test (OGTT) glucose (102.5 ± 24.9 vs 93.5 ± 20.3 mg/dL), post-OGTT insulin (159.6 ± 157.3 vs 67.6 ± 58 µUI/mL) and triglycerides (107.7 ± 52.7 vs 86.1 ± 42 mg/dL) were statistically higher in PCOS adolescents. QUICK (0.307 ± 0.029 vs 0.333 ± 0.033), fasting glucose/insulin ratio (4.91 ± 3.81 vs 8.07 ± 5.45) and HDL-cholesterol (44.8 ± 9.4 vs 49.8 ± 10 mg/dL) were statistically lower in the PCOS group. MS, according to International Diabetes Federation criteria, was diagnosed in 41.8% of PCOS girls vs 4.2% of non-PCOS girls (p < 0.0001).

**Conclusion:** Our results demonstrated that obese and overweight adolescents with PCOS present a higher prevalence of IR when compared to BMI-matched *non*-*PCOS* adolescents. These findings reinforce the need for a careful evaluation of clinical/laboratory markers of IR in PCOS girls and suggest that assessment of post-OGTT insulin levels may be a useful tool in this investigation.

### P101 Evaluation of comorbidities and clinical outcomes of nine patients with lipodystrophy treated in an University Hospital

#### Isabella Carvalho Oliveira, Dandara Sampaio Leão De Carvalho, Raissa Carneiro Rezende, Guilherme Borges De Andrade, Monike Lourenço Dias Rodrigues, Daniela Pultrini Pereira De Oliveira Viggiano, Daniela Espindola Antunes, Estela Muszkat Jatene

##### Universidade Federal de Goiás, Goiânia, Brazil

*Diabetology & Metabolic Syndrome* 2019, **11(Suppl 1):**P101

**Introduction and objective:** The lipodystrophy syndromes are a rare and heterogeneous group of diseases distinguished by the lack of peripheral adipose fabric which leads to a nutrient storage deficiency and the deposit in ectopic organs. Consequently, the patient shows severe insulin resistance and metabolic abnormalities. The lipodystrophies are classified in partials and generalized, which can be congenital or acquired. This study has the objective of describing nine cases of Lipodystrophy being treated in an University Hospital.

**Method:** A descriptive study of a series of cases.

**Results:** Nine cases of Familial Partial Lipodystrophy were identified. All the patients were women, with an average age of 42.7 years old. According to the body mass index, one patient was eutrophic; one was overweight; five with class 1 obesity; one with class 2 obesity and one with class 3 obesity. According to the comorbidities, “Diabetes Mellitus (DM)” was present in 77.7% of the cases, Hepatic Steatosis (77.7%), Hypertension (88%), Neuropathy (33%), Nephropathy (55%), Ophthalmopathy (22%), Dyslipidemia (100%), Metabolic Syndrome (77%). Pancreatitis wasn’t found in none of the patients. Two out of seven patients with DM show a late diagnosis of Lipodystrophy (after 21 years of DM). The other patients were simultaneously diagnosed with diabetes. The two patients with Lipodystrophy and that didn’t have DM, one showed a normal glycemic curve and the other showed Glucose Intolerance. Both have positive family history for Lipodystrophy with the syndrome appearing in grandmothers, mothers and aunts. Hypertriglyceridemia was present in 88% of the cases and Low HDL cholesterol in 55%. In general, the patients with hypertriglyceridemia used to keep high levels of triglycerides even though they were under treatment. A patient started the Dialytic Therapy when she was 52 years old, after 14 years of DM. Three patients were using Pioglitazone, a drug that provenly reduces the insulin peripheral resistance and it’s capable of improving the fat peripheral distribution. The biggest limiter for its use was the cost itself.

**Conclusion:** Lipodystrophy still is underdiagnosed in the population, including diabetics, and its early diagnosis is important for a more severe monitoring of the disease and also to track other metabolic conditions. The phenotype is very important for the diagnosis, mainly when mutation tests aren’t available.

### P102 Evaluation of flash glucose monitoring system technology as a resource to detect hypoglycemic events bringing better glycemic control in type 2 diabetic patients

#### Raquel Donadel Kroth^1^, Ana Cláudia Borges Do Carmo^1^, Renata Francioni Lopes Zappalà^2^, Mirella Hansen De Almeida^1^, Elaine Maria Dos Santos Gomes^1^, Jéssica Ramos Tavares^1^, Bruna Cardoso Borges^1^, Ana Eduarda Vieira Moerbeck^1^

##### ^1^Serviço de Endocrinologia do Hospital Central da Aeronáutica (HCA) - Rio de Janeiro, Brazil; ^2^Endocrinologia Força Aérea Brasileira, Rio de Janeiro, Brazil

*Diabetology & Metabolic Syndrome* 2019, **11(Suppl 1):**P102

**Introduction:** Brazil occupies the 4th position in the world ranking of people with diabetes according to data from the International Diabetes Federation (IDF). Inadequate glycemic control and high glycemic variability are important factors in the development of micro and macrovascular complications. The use of technologies to improve glycemic control helps in detecting hypoglycemia, brings convenience and minimizes morbidity and mortality in this population.

**Objective:** To analyze the incidence of hypoglycemia and glycemic control in patients with type 2 diabetes (DM2) using the flash glucose monitoring system (FGM).

**Methods:** A retrospective cross-sectional study was carried out to evaluate 100 sensors installed between 04/01/2018 and 07/31/2019 in 58 DM2 patients using FGM. Each FGM sensor was used for a period of 10 to 14 days subcutaneously and were analyzed using the ambulatory glucose profile software (AGP) the estimated A1C and percent target time (TIR) (target: 70–180 mg/dL), time above and below target, number of hypoglycemia and percentage of scans over the period.

**Results:** The age of the patients ranged from 45 to 87 years, with 50% women and 50% men. It was observed that the estimated A1C average was 6.7%, with an average of 10.63 daily scans per period in the evaluated sensors. Estimated A1c > 7% was found in 35% of sensors and estimated A1C ≤ 7% in 65% of sensors. Considering that the ideal TIR should be above 70%, 58% of patients were within target and 31% were above target. Hypoglycemic events ranged from 0 to 28 episodes per period. When the capture percentage was ≥ 80%, the average event of hypoglycemia was 6.74 events per period, when the capture was < 80%, there was an average of 3.10 events; the Student t-test was 0.001 showing statistical significance. In the overall evaluation, 38% of patients with hypoglycemia (< 70 mg/dL) had the duration greater than 4% of the time evaluated. The average of hypoglycemia in 21 patients who used more than one sensor during the study period dropped from 9.66 to 4.14 per period.

**Conclusion:** New forms of glycemic monitoring, such as FGM, help minimize glycemic variability, identify hypoglycemia, and reduce time spent on events. The use of specific metrics of these new technologies brings better glycemic control in diabetic patients, in addition to the traditional HbA1c, and may minimize the risk of micro and macrovascular complications in the future.

### P103 Evaluation of glycemic control in patients with type 1 diabetes with flash continuous glucose monitoring

#### Matheus Cisneiros Silva de Oliveira, Guilherme Andrade Nascimento, Thayanne Karolyne Andrade, Maria de Lourdes Passos Machado, Lysandro Pinto Borges, Naira Horta Melo, Karla Freire Rezende, Carla Raquel Pereira Oliveira

##### Universidade Federal de Sergipe, Aracaju, Brazil

*Diabetology & Metabolic Syndrome* 2019, **11(Suppl 1):**P103

**Introduction:** Use of new technologies has contributed to the clinical and therapeutic management of diabetes mellitus, and in the improvement of metabolic control. Patients with type 1 diabetes (DM1), due to the high glycemic lability, require more frequent self-monitoring, usually performed through digital puncture. In Aracaju-SE, necessary supplies are available and patients clinically followed in specialized outpatient clinics, even users of unified health system (SUS) can perform self-monitoring 3–5 times a day.

**Objective:** Evaluate the impact of subcutaneous flash glucose self-monitoring (FGM) on glycated hemoglobin (HbA1C) and insulin therapy in DM1 patients accustomed to conventional self-monitoring 3–5 times a day.

**Methods:** Prospective cohort study with 35 patients with DM1 (22 women, 31.7 ± 10.3 years, 60.9 ± 13.6 kg, 1.6 ± 0.08 m, BMI of 23.4 ± 5.04 m/kg^2^, more than 60% have DM1 diagnosis for over 10 years and 80% were SUS users). These patients used FreeStyle^®^ Libre, a FGM subcutaneous sensor, changed every 15 days by the healthcare team which performend clinical evaluation and insulin therapy adjustments for 3 months. HbA1C by HPLC (mg/dL) was dosed on the day of the first sensor application and the day of last withdrawal. Total daily insulin dose (TDID, U), sum of basal and prandial dose; total basal daily dose (TBD, U) and basal insulin percentage of TDID (%) were also evaluated before and after 3 months. Data were expressed as mean ± standard deviation, statistical analysis performed by IBM SPSS version 2.0 software through paired T test and considered significant p < 0.05. This study was approved by ethics committee (CAAE: 14555719.7.0000.5546).

**Results:** HbA1C decreased from 8.6 ± 1.2% to 7.9 ± 0.8% (p = 0.001). TDID did not change (44.1 ± 21U to 42.7 ± 21.5U; p = 0.346). TBD and basal insulin percentage decreased (26.5 ± 12.9U to 23.8 ± 12.1U; p = 0.007 and 60 ± 10% to 50 ± 10%; p = 0.001, respectively).

**Conclusions:** The exchange of conventional self-monitoring with multiple digital punctures by the use of a sensor promoted a reduction in HbA1C without increasing total insulin dose of patients with DM1 SUS users. Sensor informations promoted a improvement in basal bolus ratio. Support: Sensors provided by Abott.

### P104 Evaluation of knowledge and performance of community health workers about diabetes mellitus

#### Suzana Nunes Rabelo, Janice Sepúlveda Reis, Tatiana Teixeira de Miranda, Eduardo Maciel Cordeiro, Aleida Nazareth Soares

##### Ensino E Pesquisa da Santa Casa de Belo Horizonte, Belo Horizonte, Brazil

*Diabetology & Metabolic Syndrome* 2019, **11(Suppl 1):**P104

**Introduction:** The primary attention should be prepared to give support to a large number of patients with diabetes mellitus (DM). Thus, community health workers (CHW) should be able to effectively exercise their role in the prevention and control of diabetes and its comorbidities.

**Objective:** To evaluate the knowledge and the performance of CHW in the follow up of people with DM.

**Methods:** A cross-sectional study was performed using validated questionnaire (Diabetes-CHWs), with questions about sociodemographic profile, performance of duties and knowledge about diabetes and its comorbidities in Belo Horizonte, Minas Gerais, Brazil. 341 CHW were chosen randomly through stratified proportional sampling according to the health units. The normality was assessed by the Kolmogorov–Smirnov test. The association of socio-demographic profile and variables related to DM were assessed using the median correct score: Mann–Whitney and Kruskal–Wallis tests. Moreover, the associations between each item about knowledge in DM were evaluated using Chi square test. The level of significance was 5%. The software utilized was SPSS 20.0 version. The project was approved by the Research Ethics Committee of the responsible institutions.

**Results:** Most of CHW were female (92.54%), mean age 44.9 years, working in this function more than 6 years (88.17%) and with more than 8 years of schooling (86.89%). In their jobs, 58.1% assisted more than 750 people and 66.32% had difficulties to do home visits. The instrument has 41 items and each correct answer was considered as 1 point. The median of correct answers was 27 points (4–38). Regarding the CWH attributions in monitoring people with DM, 62.98% got at least one item wrong of the question. Regarding risk factors for DM, only 28.53% correctly answered the total of factors presented in the instrument. The percentage of correct answers was less than 34% regarding the minimum number of consultations/years with health professionals (doctor, dentist, nutritionist and foot evaluation). Longer CHW experience and skills training were associated with higher correct answer scores(p = 0.035; p = 0.040). There was a significant correlation between knowledge of CHW attributions, knowledge of the minimum number of consultations with physicians and training on DM (p < 0.05).

**Conclusion:** The CHW level of knowledge about DM is not enough to accompany people with DM and educational investment is required for these professionals.

### P105 Evaluation of peripheral neuropathy in diabetic patients in a specialized multidisciplinary care service

#### Barbara Franz Luvison^1^, Claudia Pinheiro Sanches^1^, Tatiane Coradassi^1^, Andressa Miguel Leitão^1^, Fernanda Nizar Dassoler^2^

##### ^1^Centro de Diabetes Curitiba - Hospital Nossa Senhora das Graças - Curitiba, Brazil; ^2^Hospital Pequeno Príncipe - Curitiba, Brazil

*Diabetology & Metabolic Syndrome* 2019, **11(Suppl 1):**P105

**Introduction:** Diabetes Mellitus is a chronic and progressive disease which culminates in microvascular and macrovascular complications. Distal polyneuropathy is one of the most prevalent complications in diabetic patients; however, it is still an underdiagnosed and undertreated condition, even in referral centers.

**Objective:** The aim of the study was to identify the prevalence of foot examination alterations and the presence of neuropathy in diabetic patients.

**Methods:** The present study followed a retrospective cross-sectional design of 149 patients, using as a strategy foot evaluation sheets from a diabetic foot prevention and treatment outpatient clinic in Curitiba. To compare quantitative variables, Student’s t-test or Mann–Whitney test were used. Categorical variables were analyzed according to Fischer test or Chi square test. P values < *0.05* were considered as statistically significant.

**Results:** 77 of the patients had peripheral neuropathy. Of these, *79.9%* had diagnosis of DM2, *16.1%* of DM1, *2.7%* LADA, and *1.3%* of pre-diabetes. From the 149 patients, 30.2% presented alteration in monofilament examination, 50.3% alteration in tuning fork test, 27.6% with alteration of Achilles reflex, and 5.8% with deviation in brachial ankle index. Regarding the comparison of distributions, there was a statistical significance regarding monofilament alteration and tuning fork test among type 2 or pre-diabetic patients compared to type 1 or LADA (p 0.012), as well as significance of the group associated with the profile. unfavorable for complications (p 0.002), along with the risk of deformity, ulcer or amputation neuropathy (p 0.043). It is also noted that 77.9% of patients did not receive guidance on foot care, and 73.6% never underwent the respective examination.

**Conclusion:** There was a significant prevalence of peripheral neuropathy and foot examination alterations in type 2 diabetes and pre-diabetic patients. In addition, there was an unsatisfactory rate of patients who underwent foot examination and guidance. Thus, we emphasize the importance of the use of preventive strategies by multidisciplinary teams for diabetic patients, in order to prevent and delay the evolution of neuropathy and optimize glycemic control.

### P106 Evaluation of psychological attitudes in type 2 diabetes mellitus of health professionals in primary

#### Joseane da Silva^1^, Heloísa de Carvalho Torres^2^, Daniel Nogueira Cortez^3^

##### ^1^Secretaria Municipal de Saúde de Divinópolis/MG, Divinópolis, Brazil; ^2^Universidade Federal de Minas Gerais, Belo Horizonte, Brazil; ^3^Universidade Federal de São João Del-Rei, Divinópolis, Brazil

*Diabetology & Metabolic Syndrome* 2019, **11(Suppl 1):**P106

**Introduction:** Diabetes Mellitus stands out as one of the four major non-communicable diseases worldwide with future estimates of increased prevalence. The complexity involved in the care of this condition makes its management still a challenge for health professionals and for the health system. In this context, Primary Health Care should be presented as a favorable scenario, with a multiprofessional approach and empowering posture. These actions are influenced by the attitudes that health professionals have regarding the clinical and psychosocial aspects that cover the condition of diabetes.

**Objective:** To identify the psychological attitudes in diabetes mellitus type 2 of primary health care professionals.

**Methods:** A cross-sectional study was carried out in a city in the central-western region of Minas Gerais, Brazil, with 56 professionals professionals of the area of ​​Health of superior level (doctors, nurses, dentists, pharmacists, physiotherapists and psychologists) of Family Health Strategies teams, using the Professional Attitudes Scale instrument in relation to DM (EAP-DM). This instrument stands out for evaluating a greater number of dimensions of DM type 2 care, such as the severity of diabetes, the autonomy of the person who has DM in the therapeutic decision process, the psychosocial impact of diabetes on the life of the person living with the condition and has scores ranging from 1 to 5. Frequency measurements were performed and after normality tests, the data were analyzed by the Mann–Whitney and Kruskal–Wallis tests. Measures were performed frequency and after normality tests, the data were analyzed by the Mann–Whitney and Kruskal–Wallis tests.

**Results:** All professionals presented positive attitudes regarding diabetes with scores above 3 for the applied questionnaire varying between 3.76 and 4.85. It should be emphasized that physicians and psychologists demonstrated less favorable attitudes than nurses and physiotherapists (p value < 0.05).

**Conclusions:** The findings reinforce the importance of reorienting the competencies of health professionals and an interdisciplinary approach.

### P107 Evaluation of skin temperature, sensitivity and vascular aspects of lower limbs affected by diabetic foot ulcers

#### Nathalia Cristina de Souza Borges, Mário Machado Perissini, Marcela Silva Carvalho, Luíza Rocha Soares, Elaine Caldeira de Oliveira Guirro, Maria Cristina Foss de Freitas, Rinaldo Roberto de Jesus Guirro

##### Faculdade de Medicina de Ribeirão Preto da Universidade de São Paulo, Ribeirão Preto, Brazil

*Diabetology & Metabolic Syndrome* 2019, **11(Suppl 1):**P107

**Introduction:** Diabetic foot ulcers have several complicating factors that affect the healing process. A good understanding and thorough evaluation are crucial to the establishment of adequate intervention strategies to prevent amputations.

**Objective:** Analyze skin temperature, sensitivity in the region of the ulcer and the ankle-brachial index of diabetic individuals with skin ulcers.

**Methods:** The present observational, cross-sectional study received approval from the institutional review board (certificate number: 2.724.659) and involved 30 male and female diabetic patients (age range: 45 to 62 years) with a diagnosis of peripheral diabetic neuropathy and chronic, uninfected plantar ulcers for at least four weeks (Grade I or II of the Meggitt-Wagner classification). The following were evaluated on a single occasion: mean skin temperature of the wound region (WST) and plantar (PST) bilaterally using infrared thermography; sensitivity around the wound using a digital esthesiometer; and vascular aspects in both lower limbs using the ankle-brachial index (ABI). The Shapiro–Wilk test was used to determine the distribution of the data. The t-test for independent samples was used for the comparison of PST on the foot with the ulcer and the contralateral foot. Cohen’s d was used to determine the effect size. Pearson’s and Spearman’s correlation coefficients were calculated to measure the strength of correlations between variables. The statistical analyses were performed with the aid of the SPSS version 17.0, with the significance level set to 5% (p < 0.05).

**Results:** Mean WST was 33.49 ± 0.68 °C. Mean PST was 31.69 ± 1.29 °C on the foot with the ulcer and 29.11 ± 1.66 °C on the foot without the ulcer. This difference was statistically significant (p < 0.05) and had a large clinical effect (d = -1.74). Mean sensitivity was 773.11 ± 194.98 grams. ABI values were normal: 1.08 ± 0.14 on the limb with the ulcer and 1.12 ± 0.13 on the limb without the ulcer. No significant correlations were found in these analyses (p > 0.05).

**Conclusion:** Mean plantar skin temperature was 2.58 °C higher on the foot with the diabetic ulcer compared to the contralateral foot in patients with no vascular alterations detectable by the ankle-brachial index. This difference in temperature may stem from the inflammatory process involved in the healing process.

### P108 Evaluation of the behavioral program in diabetes mellitus type 2: randomized clinical trial study protocol

#### Laura Barbosa Nunes, Jéssica Caroline dos Santos, Heloísa de Carvalho Torres

##### Universidade Federal de Minas Gerais, Belo Horizonte, Brazil

*Diabetology & Metabolic Syndrome* 2019, **11(Suppl 1):**P108

**Introduction:** Educational strategies are foundations for the prevention of complications and the promotion of self-care of this chronic condition, provided that the interventions are based on effective and reliable communication between patient and professional, resulting in reflective dialogue regarding self-care, as well as rescuing behavioral aspects, psychosocial and clinical. Given this fact, and the increasing use of technologies, a behavioral program was developed that addresses two methodological strategies, such as group and telephone interventions, which **aims** to describe the protocol of a clinical trial designed to evaluate the effect of the behavioral program on diabetes mellitus type 2.

**Methods:** This is a cluster randomized clinical trial conducted in six Basic Health Units of Belo Horizonte-MG. Each health unit was considered a cluster, and three units were allocated to intervention groups (117 participants) with application of the behavioral program and the other three in a control group (94 participants) who will receive usual services from health units, which total the participation of 211 people with DM2. The primary outcome is improvement in glycated hemoglobin by the twelfth month of intervention. While the secondary outcome concerns the modification of psychological attitudes and empowerment for self-care. All research ethics standards were met in accordance with National Health Council Resolution 466/12.

**Results:** The protocol was based on the Health Belief Model Theory and the Cognitive Social Theory, which includes the educational group, which will be used the contact time (hours), number of meetings per cycle, number of health professionals, approach with different themes: psychosocial aspects, eating patterns, physical exercise and medications. In contrast, the use of the telephone will strengthen interaction between the subjects involved, optimize time and reach a large number of users with work-related geographic, financial barriers. The intervention was adjusted according to the availability of the participant. The behavioral program will last 12 months.

**Conclusions:** This study will provide information on the effectiveness of a health-based behavioral program for self-care. In addition, the findings will improve our understanding of how telephone interventions can benefit many people with DM 2. Financial Support: CNPq 432824/2016; FAPEMIG APQ-03865-16.

### P109 Evaluation of the effects of antioxidant treatment on morphology, oxidative stress and cardiac inflammation in diabetic rats

#### Hilary Barros de Carvalho, Natan Reyges Castro da Purificação, Melyna Soares de Souto, Rayla Thais Oliveira Chavarria, Lucas Alexandre Araújo Lins, Andreza Nathalya Araújo de Miranda, Jayra Conceição Cândido da Silva, Naianne Kelly Clebis

##### Universidade Federal do Rio Grande do Norte - Laboratório de Microscopia Celular e Tecidual/DMOR, Natal, Brazil

*Diabetology & Metabolic Syndrome* 2019, **11(Suppl 1):**P109

**Introduction:** Diabetic cardiomyopathy (CM) is characterized by hypertrophy and cardiac fibrosis. It occurs by morphofunctional changes in the heart resulting from hyperglycemia that leads to increased oxidative stress and inflammation tissue.

**Objective:** The aim of this study was to evaluate the action of antioxidant therapy on the cardiac morphology of rats with diabetes mellitus (DM).

**Methodology:** 20 male rats were divided into (n = 5): normoglycemic (N); diabetics (D); normoglycemic treated with antioxidants (NT) and; diabetic treated with antioxidants (DT). DM was induced with 35 mg/kg body weight of streptozotocin. The treatment was performed with: quercetin (100 mg/kg body weight), l-glutamine 1% and α-tocopherol 1% for 60 days. Body weight, weight coefficient (heart weight/body weight − CP), SOD-1, GPx-1, IL-1β expression and amount of collagen fibers (Sirius red) in 3 µm-thick myocardial sections were analyzed. Statistical analysis was performed by Fisher’s test (p < 0.05%).

**Results:** There was a reduction in body weight in D and DT compared to normoglycemic animals with and without treatment. CP was higher in D and NT than in N, but was similar between D and DT. The expression of SOD-1 was higher in D than in N and no difference was observed between D and DT groups, and between N and NT. GPx-1 expression was higher in D and NT than N, but lower in DT than D. IL-1β expression was higher in D than in N, but similar between DT and D groups, and between NT and N. There was an increase in the deposition of collagen fibers in group D and NT compared to N, but in DT there was a reduction compared to D.

**Conclusions:** Antioxidant therapy did not reverse body weight loss and cardiac hypertrophy in DT. DM caused increased expression of SOD-1, GPX, IL-1β and collagen fibers in D, but DT treatment reduced oxidative stress (GPx-1) suggesting activation of the glutathione pathway leading to reduction of cardiac fibrosis, but the opposite was evidenced in NT, with increased GPx-1 and fibrosis, demonstrating that the use of antioxidants in normal condition is not indicated.

**Keywords:** Hyperglycemia; Endomyocardial fibrosis; Antioxidants; Ventricular remodeling.

### P110 Evaluation of the epidemiological profile and influence of diabetes mellitus’s duration in signs and symptoms of diabetic peripheral neuropathy in patients of a reference center

#### Vinicius Ribeiro Ferreira Mendonça Gonçalves, Lucia Henriques Alves da Silva, Camila Vicente dos Santos, Rosane Kupfer

##### Instituto Estadual de Diabetes e Endocrinologia Luiz Capriglione - IEDE/RJ, Rio de Janeiro, Brazil

*Diabetology & Metabolic Syndrome* 2019, **11(Suppl 1):**P110

**Background:** The diabetic peripheral neuropathy (DPN) is still barely diagnosed despite being one of the most prevalent microvascular complications secondary to Diabetes Mellitus (DM). Observational studies demonstrate that DM duration act as a risk factor for the development of DPN, although the actual data do not confirm its influence on severity of signs and symptoms.

**Objectives:** This study evaluate the epidemiological profile of a group of patientsin a Diabetic Neuropathy ambulatory of a Reference Center. Additionally, it verifies the influence of DM duration in the severity of signs and symptoms of DPN, quantified by scores (neurologic symptom score - NSS, pain analogical scale – PAS, and neurologic impairment score - NIS).

**Methods:** This is an observational, cross-sectional and retrospective study. It analyses the database of the Diabetic Neuropathy ambulatory of a Reference Center. The population of this study was composed of a total of 68 patients diagnosed with DPN, that were splited into two groups: the ones with less than 20 years of DM diagnosis (n: 32), and the ones with more than 20 years of DM diagnosis (n: 36). Epidemiological, laboratory and the scores of neuropathic signs and symptoms previously mentioned were analyzed and compared between the two groups. This study was approved by the Ethics Committee of our instituiton.

**Results:** The patient’s average age was 58.5 years, there was a greater prevalence of DM type 2 (82.4%), the DM average duration was 19,5 years and the average glycated haemoglobin was 8.1%, in both groups. The patients had a high prevalence of diabetic retinopathy (50%) and nephropathy (51%). The NSS and the PAS were classified as moderate, and the NIS was classified as low in the entire population. There was no difference in the occurrence of amputations and neurologic signs and symptoms between the two groups.

**Conclusions:** This population analysis showed that the patients already had moderate sypmtoms of DPN at the first evaluation of diabetic neuropathy (even with low occurrence of neurologic alterations), besides high prevalence of other microvascular complications. DM duration did not influenced in the severity of neuropathic signs and symptoms. However, the active screening and early diagnosis of DPN must be priorities to avoid severe and potentially irreversible clinical manifestations of the disease. We had our own financial support for this study.

### P111 Evaluation of the impact of an educational program for hospitalized patients on their diabetes knowledge

#### Flávia Coimbra Pontes Maia, Maria Regina Calsolari Pereira de Souza, Janice Sepúlveda Reis, Adriana Aparecida Bosco

##### Santa Casa de Belo Horizonte, Belo Horizonte, Brazil

*Diabetology & Metabolic Syndrome* 2019, **11(Suppl 1):**P111

**Introduction:** Diabetes mellitus (DM) is a chronic disease and a serious public health issue all over the world and despite improvements in medical knowledge, technological advances, and medications, only a small percentage of patients fulfill the therapeutic goals. Once patients are hospitalized for the treatment of acute conditions, despite the physical stress related to the seriousness of the disease and the emotional stress, this period represents a window of opportunity to assess and improve knowledge about the disease: time to attract the patient’s attention and to provide information about self-care. So far, there are no sufficient data permitting to assess the effectiveness of knowledge transmission during hospitalization in Brazil.

**Objective:** The aim of this study was to evaluate the impact of an educational program on the knowledge of patients with DM hospitalized for the treatment of any additional conditions.

**Methods:** Fifty hospitalized patients admitted for the treatment of diabetic complications or comorbidities in the largest public hospital of Minas Gerais, Brazil, were evaluated by the Diabetes Knowledge Questionnaire with questions about:basic physiology, insulin action, hypoglycemia, food groups and substitutions, techniques for insulin use, self-monitoring, and principles of diabetes care. It was applied individually to each patientbefore and 2 months after his participation in an interactive 60-minute class, including guidelines about diabetes, its management and complications. The questions were grouped into 6 knowledge domains to identify topics with more difficulties for the patients: diagnosis, diet, symptoms, diabetic complications, hypoglycemia, and diabetes control. This study was approved by Institutional Ethics Committee.

**Results:** The diabetes knowledge significantly improved after the educational program (p < 0.001), in the diet (p < 0.001), complications (p 0.009), hypoglycemia (p < 0.001) and control (p 0.02) domains, with no difference in the symptoms domain (p 0.152) or in relation to duration of the diagnosis (p = 0.071), sex (p = 0.160) or time since diagnosis (p = 0.456), but patients with type 1 DM had better scores than type 2 DM patients (p = 0.003).

**Conclusion:** Educational programs for hospitalized patients with DM can improve their knowledge about the disease and this period could be used as an opportunity to access the patient to the knowledge about the disease.

### P112 Evaluation of the impact of diabetes education and the medicament delivery on glycemic control and speckle tracking in patients with type 2 diabetes mellitus

#### Juliane Colombo Carrer De Macedo, Janice Sepúlveda Reis, Raldner Borges E Reges

##### Santa Casa de Belo Horizonte/MG - Ensino e Pesquisa, Belo Horizonte, Brazil

*Diabetology & Metabolic Syndrome* 2019, **11(Suppl 1):**P112

**Introduction:** Type 2 diabetes mellitus (TD2 M) is a chronic condition which requires special behaviors, self-care and knowledge. It is lack of control can lead to chronic complications, such as diabetic cardiomyopathy, defined as left ventricular dysfunction, and may affect quality of life. Therapeutic effectiveness depends on educational strategies for patients to have support and assume responsibilities. Some recent findings add knowledge in these intricate mechanisms and relate the dipeptidyl peptidase-4 inhibitors (DPP4i) with them.

**Objective:** To evaluate the control of the disease, cardiac dysfunction and quality of life in a population with uncontrolled TD2 M, non-adherent to treatment and with subclinical heart injury, before and after multi-professional follow-up, with regular supply of the DPP4i by a public hospital.

**Methods:** We selected 32 patients (18 women; age of 54.3 ± 5.4 years) with hemoglobin A1c > 7.0%, non-adherent by the Measurement of Adherence to Treatment (MAT), with asymptomatic cardiac dysfunction by two dimensional speckle tracking echocardiography (2DSTE) and quality of life impairment using Problem Areas in Diabetes (B-PAID) (Selection: M0). All patients used DPP4i throughout the study and after 3 months of initiation of the medication the laboratory tests were repeated (Pre educational intervention: M1) and the nutrition, psychology, nursing and physical education groups started for 4 consecutive weeks. After 3 months of the groups, all patients were reassessed by laboratory tests, 2DSTE and evaluation instruments (Pos educational intervention: M2).

**Results:** Comparing results in M0 and M2, we observed a significant improvement in treatment adherence (MAT: 2.5 ± 1.9 vs 5.7 ± 2.7; p < 0.001), quality of life (B-PAID: 62.4 ± 17.5 vs 19.4 ± 21.0; p < 0.001), subclinical cardiac dysfunction (2DSTE: 15.4 ± 3.0 vs -17.6 ± 3.4; p < 0.001), C-peptide (ng/ml) (2.0 ± 0.9 vs 2.8 ± 0.9; p < 0.001), sexual dysfunction (p < 0.001) and body weight/height^2^ (BMI) (kg/m^2^) (31.3 ± 5.9 vs 29.9 ± 5.6; p < 0.001). When compared results in M1 and M2, we observed a significant improvement in hemoglobin A1c (7.7 ± 0.9 vs 6.9 ± 1.2%; p < 0.001).

**Conclusions:** This study showed that regular medication and multi-professional diabetes education improved adherence to treatment, glycemic control, beta cell reserve, BMI, sexual dysfunction, asymptomatic heart injury and consequently quality of life.

**Financial support:** (Not applicable).

### P113 Evaluation of the knowledge of diabetes mellitus patients on the conservation of insulin pens and flasks

#### Ana Beatriz de Andrade Soares de Oliveira, Lorena Fortuna da Silva, Sávio Dias de Paula Mello, Marcela dos Santos Ferreira, Júlio Cesar Santos da Silva, Úrsula Pérsia Paulo dos Santos, Cristiane Rosa Magalhães, Fernanda Zerbinato Bispo Velasco

##### CEFET/RJ, Rio de Janeiro, Brazil

*Diabetology & Metabolic Syndrome* 2019, **11(Suppl 1):**P113

**Introduction and objectives:** Insulin is one of the forms of treatment of diabetes mellitus (DM), requiring a important care in its conservation so that its effect is guaranteed. Glycemic control also depends on maintaining insulin quality, requiring knowledge of the ideal way to conserve it. This study evaluated the knowledge of DM patients about insulin retention.

**Methods and results:** Descriptive and cross-sectional study, conducted in the first half of 2018. The target population were insulin-carrying diabetes mellitus patients using syringes or pens. A closed questionnaire containing questions about the conservation and packaging of the insulin bottle and applicator pen was constructed and applied. The subjects of the research were accessed through social networks, focused on the discussion of diabetes, and through direct contact in health institutions. Of the 244 patients treated, 82.3% were older than 18 years and 69.1% had type I DM. Regarding the insulin delivery device, 41.4% used a syringe, 41% applied pen and 17.7% forms. Regarding the pen in use, 83.3% store outside the refrigerator, 63.5% of which leave it outside the original case. Those who store the bottles of insulin in use in the refrigerator (75%), keep it on the shelves (36.4%), door (25%) and drawer of vegetables (13.6%). The sealed insulin bottles are kept in the shelves (47.8%), in the door (30.5%), in the vegetable drawer (21.7%). Regarding transport of insulin to work/school and beach/pool, 82.8% and 87.3% use some heat-conditioning container, respectively. The refrigerated container for transport between the drugstore and the home is used for 50% of the sample.

**Conclusion:** It was found that there are individuals who store the insulin pen in the refrigerator and those who leave the bottles in the refrigerator door, even though they are contraindicated, as well as those who don’t use any form of packaging in the insulin bottle. Through this result, which proposes a diagnosis of the knowledge of insulin users, it is possible for health professionals to update their health education practices, making it more effective for this population.

### P114 Evaluation of the metabolic control of chronically uncontrolled type 1 diabetes patients using the flash glucose monitoring followed by a quality of life and support program (PAQVIDA)

#### Carolina Piccoli, Marcia Puñales, Ana Maria Ferreira, Balduino Tschiedel

##### Instituto da Criança com Diabetes/Grupo Hospitalar Conceição, Ministério da Saúde - Porto Alegre-RS, Porto Alegre, Brazil

*Diabetology & Metabolic Syndrome* 2019, **11(Suppl 1):**P114

**Introduction:** Some studies suggested that technologies tools in diabetes as the continuous glucose monitoring system improves metabolic control and time in glucose range in type 1 diabetes patients (T1DM). The aim of the present study was to evaluate whether the use of the Flash glucose monitoring (Flash-GM) improves the metabolic control of patients with chronically decompensated T1DM (glycated hemoglobin - HbA1c ≥ 11.0% at the beginning of the program).

**Patients and methods:** Thirty-six T1DM adolescents were followed in a quality of life and support program (PAQVIDA) for chronically uncontrolled patients. HbA1c was performed at the first day of the Flash-GM, and 2 and 4 months after the intervention and compared with estimated HbA1c (eHbA1c) of the GM-Flash.

**Results:** The mean age of the group was 17.7 ± 2.8 years and 55.5% (N = 20) were female. Of the 36 adolescents, three (8.3%) refused to install Flash-GM, four (11.1%) gave up after the second month, four (11.1%) did not attend to the last evaluation and 25 (69.4%) completed the study. At the beginning, the HbA1c (N = 33) was 11.4 ± 1.9%, No differences were found between the HbA1c and eHbA1c after 2 months (10.9 ± 2.4% and 11.1 ± 1.9%, P = 0.773) or 4 months after the technology intervention (11.0 ± 2.5% and 11.1 ± 3.2%, P = 0.899). In addition, no differences were found between HbA1c at the onset, two (P = 0.256) or 4 months (P = 0.388). The time in range was very low (22.0%, interquartile interval—IQR: 6.5–37.0%) and 4.0 (3.0–5.0) scans per day were performed in the first 2 months and 19.5% (3.0–36.5%) the consecutive months with only 2.5 (1.5–5.0) scans per day.

**Conclusion:** Our results demonstrated that using the Flash-GM technology did not improve the metabolic control of chronically uncontrolled T1DM adolescents, and a high percentage of dropouts and poor adherence reflect the poor motivation and commitment in this age group even in patients followed by a diabetes support program, which involves education and psychological assistance.

### P115 Evaluation of vitamin b12 levels in diabetic patients with metformin

#### Guilherme Soares Borges, Patrícia Moreira Melo, André Gonçalves Da Silva, Isadora Noleto Barbosa, Gerlane Araújo Moura Luz

##### Universidade Federal do Piauí, Teresina, Brazil

*Diabetology & Metabolic Syndrome* 2019, **11(Suppl 1):**P115

**Introduction:** Metformin is the preferred initial medication for type 2 diabetes mellitus and may be associated with vitamin B12 deficiency.

**Objective:** To evaluate vitamin B12 levels and associated factors in diabetic patients using metformin for at least 6 months, and to investigate whether the chance of having a low vitamin level is greater in patients with metformin than in patients without metformin and analyze the presence of peripheral neuropathy symptoms among diabetics submitted to vitamin B12 dosing.

**Methods:** Cross-sectional, observational study. There were two groups, one with diabetics in use of metformin and a control group of diabetics without the drug. The association between vitamin B12 (normal or deficient) and age, sex, metformin dose, use of a proton pump inhibitor (PPI) and H2 antagonists in biguanide users was investigated, and also the symptoms of peripheral neuropathy between the participants.

**Results:** There were 135 participants, 11.71% with vitamin B12 deficiency and 12.59% with symptoms of peripheral neuropathy. There was no association between age, sex, metformin dose, use of PPIs and H2 antagonists with vitamin deficiency. There was no significance in the test for association between medication and hypovitaminosis.

**Conclusions:** The frequency of vitamin B12 deficiency was lower than in other health services and there was no evidence of association with the variables studied. A considerable number of patients reported symptoms of peripheral neuropathy. It has not been possible to establish whether the chance of having a low vitamin B12 level is greater in patients using metformin.

### P116 Exercise order alters glycemic response in type 2 diabetics: a case study

#### Valesca Nayara Silva e Silva, Camila Lima Barbosa, Jonathan Nícolas dos Santos Ribeiro, Iago Vilela Dantas, Rodrigo dos Santos Rodrigues Alves, Ricardo Hosanã Dias da Silva, Cláudio Barnabé dos Santos Cavalcanti, Denise Maria Martins Vancea

##### Universidade de Pernambuco, Recife, Brazil

*Diabetology & Metabolic Syndrome* 2019, **11(Suppl 1):**P116

**Introduction:** Aerobic and strength training are recommended for the control of diabetes, but it is not clear the effect of the sequence of these exercises in the same session for glycemic control of type 2 diabetics.

**Objective:** To evaluate blood glucose response of type 2 diabetics submitted to different training orders.

**Methods:** This study was characterized as a case study and was approved by Ethics Committee 007/09. Five women with type 2 diabetes mellitus (T2DM) attended a supervised exercise program for diabetics at a public university. They performed 12 combined training sessions (aerobic and strength in a row). The training program was divided into two moments: six sessions where the order was aerobic exercise followed by strength exercises (AS) and six sessions where strength exercises were followed by aerobic exercise (SA). Blood glucose samples were collected before, during and after each session. The parametric repeated measures ANOVA test was performed and the significance level of p ≤ 0.05 was adopted.

**Results:** There was a significant reduction in capillary blood glucose at AS order from pre to post intervention (169 vs 117.3 mg/dL; ∆ = − 52.6 mg/dL; p = 0.00). The SA order session was also capable to decreased glycemic levels from pre to post intervention (166.6 vs 118,4 mg/dL; ∆ = − 48.2 mg/dL; p = 0.00). In the intragroup analysis, the AS and SA had no significant difference (p = 0.68).

**Conclusion:** Both interventions were equally beneficial for glycemic control since they caused a reduction in capillary blood glucose without significant difference between the orders performed by the diabetics in this study.

### P117 Factors associated with dynapenia in older patients with type 2 diabetes

#### Roberta Arnoldi Cobas, Jefferson Teixeira Da Costa, Paula De Campos Calassara, Ana Luiza Nicoletti Dias, Roselee Pozzan, Luciane Pires Da Costa, Lucianne R M Tannus, Paulo Sergio Chagas Gomes

##### Universidade do Estado do Rio de Janeiro, Rio de Janeiro, Brazil

*Diabetology & Metabolic Syndrome* 2019, **11(Suppl 1):**P117

**Introduction:** Dynapenia is defined as loss of muscle strength or function and is associated with disability and metabolic disorders.

**Objective:** To investigate factors associated with dynapenia in older patients with type 2 diabetes (T2D).

**Methods:** Cross sectional study including patients with T2D older than 65 years evaluated for clinical parameters through structured questionnaire and medical records review. Muscle strength was evaluated by handgrip dynamometer Jamar and time on sit-to-stand chairs test. Muscle function was investigated by gait speed, Timed get up and Go test (TUG) and Short Physical Performance Battery (SPPB). Dynapenia was defined as presence of handgrip strength < 27/16 kg for men and women respectively or TUG time ≥ 20 s or gait speed < 0.8 m/s or SPPB < 8) using cut-offs from EWGSOP 2018. Muscle mass was evaluated by electrical bioimpedance. Results are presented as median [Interquartile range] and  %. SPSS/IBM22.0 was used for analysis.

**Results:** We included 77 patients, 46 (63%) women, age 73.8 ± 6.8 years, duration of diabetes 19.5 [10–25] years, A1c 7.9 ± 1.3%, BMI 27.8 ± 4.6 kg/m^2^. The presence of dynapenia was associated with history of bone fracture (45% vs 6%, p = 0.000) and cardiovascular event (30% vs 7.5%, p = 0.013) but not with presence of microvascular complications (retinopathy, nephropathy or neuropathy). In men, apendicular lean mass (ALM)/m^2^ was greater in patients without dynapenia compared to those with dynapenia (8.25 [7.47–8.68] vs 6.55 [6.37–7.45], p = 0.003) as well as ALM/BMI ratio (0.82 [0.77–0.92] vs 0.667 [0.645–0.829], p = 0.022) and skeletal muscle mass (30.2 [26.35–33.5] vs 22.6 [19.5–25.8], p = 0.001) but not ALM/body weight ratio (0.29 [0.26–0.31] vs 0.26 [0.25–0.29], p = 0.125). In women ALM/m^2^ was greater in patients without dynapenia compared to those with dynapenia (6.88 [6.39–8.03] vs 5.57 [5.14–5.98], p = 0.001) as well and skeletal muscle mass (21.55 [19.34–24.18] vs 18.1 [16.28–19.78], p = 0.005) but not ALM/body weight ratio (0.25 [0.21–0.29] vs 0.22 [0.21–0.24], p = 0.206) or ALM/BMI ratio (.0.62 [0.51–0.69] vs 0.52 [0.46–0.60], p = 0.092). Duration of diabetes and A1c levels were not different between patients with and without dynapenia.

**Conclusions:** The presence of dynapenia in older patients with T2D was associated with lower muscle mass parameters in men and women and history of bone fractures and cardiovascular disease but not with diabetes duration, glycemic control or presence of microvascular complications.

### P118 Feelings when we are on insulin pump: medical residents vs type 1 diabetes patients

#### Vanessa Araujo Montanari, Maria Gabriela Secco Cavicchioli, Paula Maria de Pascali, Tarcila Beatriz Ferraz de Campos, Vanessa Fujimoto Galves, Priscila Firmino Gonçalves Pecoli, William Ricardo Komatsu, Monica Andrade Lima Gabbay

##### Universidade Federal de São Paulo, São Paulo, Brazil

*Diabetology & Metabolic Syndrome* 2019, **11(Suppl 1):**P118

**Introduction:** Diabetes distress (DD) refers to an emotional state where people experience feelings such as stress, guilt, or denial that arise from the burden of diabetes management including counting carb, performing capillary blood tests as having frequent insulin shots at meals. Even among insulin pump users, when they have many these tasks facilitated, those with DD have poor metabolic control.

**Aims:** To compare insulin pump distress between non-diabetic medical endocrinology residents (NDMER) and type 1 diabetes mellitus (T1D) patients.

**Methods:** a questionnaire (10 questions) for adherence to insulin pump therapy was applied to 18 NDMER and 24 T1D patients from the Diabetes Outpatient Clinic. Patients has been using Accu- Chek Combo or Paradigm insulin pumps and NDMER answer the questionnaire after 3 days of pump using.

**Results:** NDMER (Age: 28.5/SD: 1.44) and T1D (Age: 29.33/SD: 6.59), and time insulin pump users (5.11/SD 3.70) years were studied. Comparison between T1D and NDMER answer shown: 1. *felt comfortable* (yes 60.9% vs 11.1%; p = 0.02); 2. *counting carbs is boring* (65.2% vs 60.1%; n.s); 3. *dressing the pump made they felt different from other people* (yes: 16.7% vs 83.3%; p = 0.001), 4. both groups *consider so much task during the meals* (56.5% vs 11.1%; p = 0.03), 5. *count carbs and give bolus 15* *min before the meals* (13% vs 44.4%; p = 0.03), 6. *the greatest CSII benefit was the comfort was bolus calculator* (76.6% vs 61.1%; p = 0.04), 7. 94.5% of T1D sad that *insulin pump brought relief and satisfaction;* 8. 88.9% NDMER sad *pump caused distress*, 9. 39.1% of T1D sad the *pump change the way they handle diabetes* and *it improved their diabetes selfcare*, finally 10. 50% NDEMR answered *they would consider more some patients complains*.

**Conclusion:** Tasks during the meals are the main causes of insulin pump distress in diabetics and non-diabetics. However, T1D considered it comfortable and beneficial to diabetes management. To NDEMR this experience might improve the contra transference during the medical relationship with these patients.

### P119 Fitness for healthy eating in the older adults with metabolic syndrome

#### Sara Franco Diniz Heitor, Nayara Cândida Gomes, Darlene Mara dos Santos Tavares

##### Universidade Federal do Triângulo Mineiro - UFTM, Uberaba, Brazil

*Diabetology & Metabolic Syndrome* 2019, **11(Suppl 1):**P119

**Introduction:** metabolic syndrome, which has a multifactorial and complex etiology, becomes more frequent with increasing age. In the management of this syndrome, an adequate diet is one of the treatments of choice because it has a direct effect on lipid profile, fasting and postprandial serum glucose levels and systemic blood pressure, due to reduced weight and visceral fat. Given the fundamental role that dietary aspects play, both in the individual components and in the prevention and control of the metabolic syndrome, and the knowledge that an inadequate diet is among the main factors that contribute to the emergence of this syndrome, it is necessary to develop research focusing this theme.

**Objectives:** to describe the sociodemographic and clinical characteristics of the older adults with metabolic syndrome; to determine the adequacy to the Food Guide 10 steps for healthy eating and to verify the influence of sociodemographic and clinical variables on the adequacy to the Food Guide.

**Methods:** study with quantitative, cross-sectional and analytical approach developed among 263 older adults with metabolic syndrome. Project approved by the Research Ethics Committee No. 950.675/2014.

**Results:** there was a predominance of female older adults (70.7%); 60├70 years (51.7%); 1├5 years of study (49.0%); income up to one minimum wage (62.4%); eutrophic (60.5%); independent for the basic activities of daily living (77.6%); dependent for instrumental activities of daily living (74.9%); without indicative of depressive symptoms (81.7%) and with positive self-rated health (50.2%). The most appropriate step was the consumption of salt (91.6%) and the least appropriate was the consumption of three portions of vegetables and fruits per day (4.6%). Adequacy to the Food Guide was not significantly influenced by education (p = 0.246); independence for basic activities of daily living (p = 0.249) and absence of indicative depressive symptoms (p = 0.113).

**Conclusion:** education, as well as independence in the basic activities of daily living and the absence of indicative of depressive symptoms did not influence the adequacy to the Food Guide. This study contributes to the characterization and knowledge of the eating habits of the older adults with metabolic syndrome and provides subsidies for the planning of health actions to improve the adherence of this population in search of reducing the risk of complications of already installed diseases.

**Financial support:** CAPES.

### P120 Food and nutritional education action in individuals with type 2 diabetes mellitus: knowledge of degree of food processing

#### Cinthia Fontes da Silva Santos, Milena Barbosa Corcino, Rafael Santos Oliveira, Dinayra Santos Pereira, Natalia Lohayne Dias Vasconcelos, Juliana de Souza Oliveira, Beatriz da Cruz Santos, Aline Rocha Reis

##### Universidade Federal de Sergipe, Aracaju, Brazil

*Diabetology & Metabolic Syndrome* 2019, **11(Suppl 1):**P120

**Introduction:** Diabetes mellitus (DM) is a metabolic disorder characterized by persistent hyperglycemia. The treatment of diabetes encompasses lifestyle changes and aims at metabolic control, that mainly involves adaptation to healthy food intake, and can be achieved with nutritional monitoring and/or educational actions. Food and Nutrition Education (FNE) actions using approaches from the Dietary Guidelines for the Brazilian Population as a basis may improve the understanding of individuals with diabetes regarding food choices based on the degree of food processing.

**Objective:** To evaluate the contribution of an FNE action in the consolidation of the concepts defined by the Dietary Guidelines for the Brazilian Population in a group of individuals with type 2 diabetes mellitus.

**Methods:** An FNE action was performed with 13 individuals with type 2 diabetes mellitus (T2DM) addressing the main concepts of the mentioned food guide. Questionnaires were applied in two moments: before (T0) and after FNE action (T1). The questionnaires contained 9 illustrated items containing 3 foods from each category: fresh, processed and ultra-processed. The foods contained in the questionnaire were meat, corn, peach, seasoned meat, pickled corn, peach in syrup, hamburger meat, corn chips and boxed peach juice. The answers were grouped into hits, errors and “could not answer”. The results were descriptive and presented in absolute and relative frequencies.

**Results:** The participants were mostly female (69.2%) and had a mean age of 65.0 ± 9.04 years. Regarding the food classification based on the mentioned guideline, the percentage of correct answers at T0 and T1 was 59.0 and 64.0%, respectively, which shows an improvement (5.0%) in the understanding of the degree of food processing after the FNE action. In addition, it was observed that the percentage of correct answers regarding foods classified as fresh remained the same (25.6%) at both times, while the hits regarding the processed foods were 17.9 and 15.4% at T0, and 20.5 and 17.9% at T1, respectively.

**Conclusion:** The FNE action has improved the understanding of food classification according to the Brazilian Dietary Guidelines, especially between processed and ultra-processed foods, that seem to be the largest source of doubt for this population and that may have an impact on unsatisfactory food choices for good metabolic control.

### P121 Food choices of adults with type 1 diabetes mellitus

#### Aline Leão Reis, Heloisy Andrea da Costa Brasil, Jeane Lorena Lima Dias, Talita Nogueira Berino, Daniela Lopes Gomes

##### Universidade Federal do Pará, Belém, Brazil

*Diabetology & Metabolic Syndrome* 2019, **11(Suppl 1):**P121

**Introduction:** Food choices are motivated by socio-cultural, biological and economic factors. Understanding these choices is critical for individualizing the nutritional treatment of people with type 1 diabetes (T1D) (Renner et al., 2012).

**Objectives:** To identify the effect of differents nutritional interventions on food choices of adults with T1D.

**Methods:** Participants were 20 to 40 years old, residents in Belém, with T1D for at least 1 year and agreed to sign the consent form. They answered to a Screening Protocol and The Eating Motivation Survey, that was developed by Renner et al. (2012) and validated for Brazil by Moraes and Alvarenga (2017), applied before (week 1, W1) and after interventions (week 12, W12), it is an instrument for assessing the motivations of eating choices, with 45 items on a Likert scale of never (1) to always (5), is divided into 15 dimensions and completed by the participants. The analysis was made from the sum of scores and intergroup comparison in W1 and W12. The subjects were randomized and distributed in control group (CG); group 1 (G1, intervention with healthy eating workshop and dietary prescription) and group 2 (G2, intervention with healthy eating, mindfulness and culinary workshops).

**Results:** Of 21 subjects, 5 were selected for research (CG = S1; G1 = S2, S3; G2 = S4, S5). The CG did not present relevant alterations in the scores. G2 [S4 = 123 (W1), 135 (W12); S5 = 69 (W1), 86 (W12)] had a higher score increase compared to G1 [S2 = 115 (W1), 118 (W12); S3 = 150 (W1), 145 (W12)], indicating for G2 that some dimensions gained strength as determinants in food choices after interventions. The strongest determinants for these choices were: Habits (S1), Liking (S2 and S5), Liking and Price (S3) and Necessity and Hunger, Health and Natural Issues (S4). Visual Appeal was identified as the least determining food choice by all subjects, accompanied by Social Image (S1, S3, S4 and S5), Emotions Control and Social Norms (S2 and S5) and Habit, Pleasure, Price, Natural Issues and Sociability (S5).

**Conclusion:** It is suggested that the combination of healthy eating guidelines, mindfulness and culinary workshops may influence more the determinants of food choices compared to the classic dietary prescription strategy associated with dietary guidelines.

**Financial support:** Coordination of Superior Level Staff Improvement.

### P122 Food industry adequacy: Brazil food trends

#### Josilene Lopes de Oliveira^1^, Ana Paula Varela Sanches^1^, Camila Neves Rodrigues^2^, Fernanda Oliveira Araújo^3^, Maíra Schuchter Ferreira^1^, Ana Iris Mendes Coelho^3^, Eliana Carla Gomes^3^

##### ^1^Universidade Estadual de Campinas, Campinas, Brazil; ^2^Universidade Federal de Minas Gerais, Belo Horizonte, Brazil; ^3^Universidade Federal de Viçosa, Viçosa, Brazil

*Diabetology & Metabolic Syndrome* 2019, **11(Suppl 1):**P122

**Introduction:** Studies show that consumers are constantly looking for healthy foods because they are conscious about relationship between food intake and health. Ensuring foods that provide healthy aging should be a major topic for research efforts for the next years. Evidence shows that certain foods have metabolic and regulatory function in the physiology of the human organism, through which they provide benefits beyond basic nutrition and can prevent disease or promote health. Observing these facts, the objective of this paper was to search for food information that fall within three categories of food trends on official food industry websites.

**Methodology:** For the research and data grouping, the foods were divided into 6 groups: (1) vegetables; (2) meat; (3) cereals; (4) milk and derivatives; (5) Vegetables; (6) oils, sugars and sweets. The categories of dietary trends analyzed were: health and wellness; sensoriality and pleasure; convenience and praticity.

**Results:** Our data show that the industries are more concerned to inform that the food is healthy. In all the groups we analyzed, this parameter presented high values, Legumes (57.1%); 2) Meat (47.6%); 3) Cereals (73.1%); 4) Milk and Derivatives (77.7%); 5) Vegetables (73%); 6) Oils, Sugars and Sweets (75%). Further than healthier products, consumers are also looking for more tasty foods, the category of sensoriality presented the second highest percentage (49.72%) among all products evaluated. Another good option is the products practical too, because in the current routine, the time for food preparation is shorter. With this in mind, the industry has also been investing in praticity, an interesting process, once we understand that some ways to reducing the time of food preparation makes it less healthy. The group of vegetables presented greater convenience and praticity (57%) when compared to the other groups, this shows the industry’s concern to attend the need of people who are on transition from a traditional diet to a diet with products ready for consumption or that require little dedicationfor their preparation, such as minimally processed vegetables or those already cooked or pre-cooked.

**Conclusion:** It is already possible to find products that attend the three categories that are part of the Brazil Food Trends proposal regarding new food trends, however, there is still a long way to go to increase the amount of food that comtemplate the characteristics of this proposal.

### P123 Fruits peel (industrial waste) extracts of *Passiflora edulis* Fo. Flavicarpa reduces glucose and improves the profile of renal and inflammatory markers in type 1 diabetes chronic model

#### Bárbara Cabral, Anderson Wilbur Lopes Andrade, Karla Simone Costa de Souza, Ony Araújo Galdino, Dáfiny Emanuele da Silva Marques, Gerlane Coelho Bernardo Guerra, Adriana Augusto de Rezende, Silvana Maria Zucolotto

##### Universidade Federal do Rio Grande do Norte, Natal, Brazil

*Diabetology & Metabolic Syndrome* 2019, **11(Suppl 1):**P123

**Introduction:** *Diabetes mellitus* (DM) has a high prevalence and constitute one of the most important risk factors for cardiovascular and kidney diseases. For DM type 1 there are few therapeutic options and many of them do not prevent the evolution of the disease. Thus, the aim of this study was to evaluate the effect of the extracts obtained of peel from *P. edulis* in association with insulin in type 1 diabetes chronic model.

**Methods:** For the evaluation of antidiabetic activity, the aqueous extract (AFA) and hydroethanolic extract (AFM) from *P. edulis* peel, were obtained. Chronic effect of extracts was evaluated by oral route in type 1 diabetes (T1DM), the animals were divided (n = 7) into: (i) control (healthy); (ii) diabetic control (T1DM); (iii) diabetic with insulinotherapy (DMT1I); (iv) diabetic with insulinotherapy + AFA 400 mg/kg (DMT1I + AFA) and (v) diabetic with insulinotherapy + AFM 400 mg/kg (DMT1I + AFM). The experimental DM was induced by an intravenous injection of streptozotocin and the duration of treatment was 60 days. The biochemical parameters analyzed were: glucose, plasma creatinine and urinary creatinine. The measurement of myeloperoxidase (MPO) was also performed in the heart and kidney. Values were expressed as mean ± standard error of mean, P < 0.05 (ANOVA and Tukey’s post-test).

**Results:** The findings showed that the treatment with the AFA extracts (50.6 ± 7.6 mg/dL, p < 0.05) and AFM (51.7 ± 6.2 mg/dL, p < 0.05) more insulin was able to significantly decrease the glycemia of diabetic rats when compared with the DMT1I group (101.3 ± 6.9, mg/dL). Plasma creatinine was decreased and urinary creatinine was increased with the treatment of AFA (0.36 ± 0.01; 35.3 ± 4.8 mg/dL, p < 0.05 respectively) and AFM (0.34 ± 0.01; 35.4 ± 6.3 mg/dL, p < 0.05 respectively), when compared to the group DMT1I (0.54 ± 0.06; 22.7 ± 2.2 mg/dL, respectively). In kidney was observed a significant decrease of MPO in the groups treated with AFA and AFM (p < 0.05), when compared to the group DMT1I. In heart only o AFM group (p < 0.05) presented a significant decrease of MPO, when compared to the DMT1I group.

**Conclusions:** Therefore, AFA and AFM extracts presented a promising biological effet as adjuvant to insulin treatment in T1DM, due to significantly decreases glucose level and the improve the profile of some kidney markers. In addition, the extracts was able attenuate the inflammatory process in kidney and heart. CEUA: (020.019/2017).

**Financial support:** CNPq.

### P124 Fruits peel of *Passiflora Edulis* Fo. Flavicarpa as a new adjunctive therapeutic agent in the prevention of diabetic nephropathy associated with type 1 diabetes mellitus

#### Ony Araujo Galdino^1^, Bárbara Cabral^2^, Dáfiny Emanuele da Silva Marques^3^, Flávio Santos da Silva^3^, Bento João da Graça Azevedo Abreu^3^, Marcela Abbott Galvão Ururahy^1^, Silvana Maria Zucolotto Langassner^2^, Adriana Augusto de Rezende^1^

##### ^1^Department of Clinical and Toxicological Analyses, Federal University of Rio Grande do Norte, Natal, Brazil; ^2^Department of Pharmacy, Federal University of Rio Grande do Norte, Natal, Brazil; ^3^Department of Morphology, Federal University of Rio Grande do Norte, Natal, Brazil

*Diabetology & Metabolic Syndrome* 2019, **11(Suppl 1):**P124

**Introduction:** Hyperglycemia caused by type 1 diabetes mellitus (T1DM) induces lipid peroxidation that modify cellular components and generate functional changes in the vascular endothelium. It is the cause of chronic complications such as diabetic nephropathy (DN), which is a main cause of chronic kidney disease in adults. Considering that adequate control of glucose levels prevents DN, *P. edulis* is a promising adjuvant therapeutic agent once it contains molecules such as flavonoids and polyphenols that inhibit GLUT1 activation and have antioxidant activity, reducing the kidney injury.

**Objective:** To evaluate the adjuvant therapeutic effect of *P. edulis* extract in the prevention of DN.

**Methods:** *P. edulis* fruit were collected and peel flour was extracted by decoction, filtered and lyophilized to obtain the aqueous extract (AFA). To evaluate the activity of the extract, the experimental T1DM model induced by streptozotocin in male Wistar rats was used (Ethics: 020.019/2017). Thirty-three rats were divided into 4 groups according to the treatment received: (1) control (C); (2) diabetic with insulin (DI); (3) diabetic with insulin + AFA 400 mg/kg (DI + AFA); (4) diabetic control (D). The duration of treatment was 60 days. After this period, the animals were euthanized and blood was removed for the evaluation of serum glucose, urea, creatinine, total cholesterol, triglycerides, HDL and LDL concentrations. The kidney was also removed for histological analysis by picrosirius red staining and total collagen was quantified.

**Results:** A significant reduction in serum glucose was observed in the DI + AFA group when compared to the DI and D groups (p < 0.001 for both). Serum urea and creatinine decreased in the DI + AFA group compared to the DI group (p = 0.011 and 0.039, respectively). A decrease in serum creatinine was also observed when comparing the DI + AFA group with the D group (p < 0.001). No significant changes in lipid profile were found. Regarding the histological analysis, there was a reduction in the percentage of collagen in the glomeruli and tubules in the DI + AFA group compared to the DI group (p < 0.001 and 0.001, respectively).

**Conclusions:** This study is a pioneer in showing the activity of *P. edulis* extract in ND, as well as presenting it as a possible adjunctive therapeutic alternative in the treatment of T1DM.

**Financial support:** CNPq.

### P125 Functional and mechanical hands assessment of physically active diabetic individuals and self-care as disorders prevention

#### Jennifer Felizardo Sodré^1^, Eduarda Pelisser Campos^1^, Isabela Duarte Machado^1^, Francieli Aparecida Meurer da Silva^1^, Ana Valéria de Souza^1^, Magnus Benetti^1^, Jefferson Luiz Brum Marques^2^, Claudia Mirian de Godoy Marques^1^

##### ^1^Santa Catarina State University, Health and Sports Science Center, Florianópolis, Brazil; ^2^Santa Catarina Federal University, Biomedical Engineering Institute, Florianópolis, Brazil

*Diabetology & Metabolic Syndrome* 2019, **11(Suppl 1):**P125

**Introduction:** Many considerations focus on various disorders and diseases afflicting diabetic individuals, but little attention is paid to the functionality of their hands.

**Objective:** To evaluate the functionality of the hands of physically active diabetic individuals and to provide guidelines regarding hands and wrists self-care.

**Methods:** Hands functionality was assessed in twenty-one diabetic mellitus type 2 individuals, men and women aged between 57 and 75 years, evaluating their physical appearance (presence of wounds, infections, deformities), range of motion of the wrists and fingers, types of sensibility (tactile and temperature), motor activity of the hand muscles (daily life activities and handgrip strength).

**Results:** Descriptive statistics showed that the most current alterations were the reduction of range of motion of fingers and wrists in 26.3% (5 of 19) of individuals was very altered, 26.3% (5 of 19) was reduced and, 47.3% (9 of 19) the range of motion was normal or with minimum alteration. In terms of the physical aspect of the hands 38% (8 of 21) showed hand callosities, 19% (4 of 21) joint deformities and, 14% (3 of 21) showed regions of infection or inflammation.

**Conclusion:** Although all individuals have shown to maintain the functionality of their hands for daily life activities, physically active diabetic individuals need continuous guidelines for hands as to prevent hands disorders and other diseases from developing, such as loss of sensibility and vascular alterations. This study is continuing for later comparisons followed by hands self-care guidelines.

### P126 Genomic ancestry and metabolic syndrome in type 1 diabetes in brazil

#### Bianca Senger Vasconcelos Barros^1^, Laura Gomes Nunes De Melo^1^, Marcela Haas Pizarro^1^, Deborah Conte Santos^1^, Luiza Harcar Muniz^1^, Dayse Aparecida Silva^2^, Luis Cristovao Porto^3^, Marilia Brito Gomes^1^

##### ^1^Universidade do Estado do Rio de Janeiro, Departamento de Medicina Interna, Diabetes, Rio de Janeiro, Brazil; ^2^Universidade do Estado do Rio de Janeiro, Laboratório de Diagnostico DNA, Rio de Janeiro, Brazil; ^3^Universidade do Estado do Rio de Janeiro, Laboratório de Histocompatibilidade e Criopreservação, Rio de Janeiro, Brazil

*Diabetology & Metabolic Syndrome* 2019, **11(Suppl 1):**P126

**Introduction:** Previous studies have investigated prevalence of metabolic syndrome in type 1 diabetic populations, with rates varying from 7 to 50%, depending on country and ethnicity. Usually participants come from homogenous populations, whether white, whether black. In admixed populations, such as Brazil, the color of the skin does not correlate well with genomic ancestry, which can modulate the expression of metabolic factors.

**Objective:** To study if genomic ancestry can influence the presence of metabolic syndrome in a type 1 diabetic population from Brazil.

**Methods:** A total of 1,698 patients with type 1 diabetes, from 14 public health centers, representative of Brazilian population was included. Participants were classified according to IDF criteria for metabolic syndrome and informed self-reported color-race. Analysis of 46 AIM-INDEL was performed to determine proportion of the three main genomic lineages (European, African e Amerindian). We compared groups with and without metabolic syndrome to determine predisposing factors. Self-reported color-race and genomic ancestry were included in two different models. The study was approved by the Ethics Committee of the coordinator center and of the other centers.

**Results:** We had complete data on 97.9% (n = 1662) of participants. Metabolic syndrome was present in 30.5%. In the first model, predisposing factors of metabolic syndrome were: female gender (OR 1.94, 95% CI 1.53–2.47, p < 0.001); age (OR 1.05, 95% CI 1.04–1.06, p < 0.001); lack of physical exercise (OR 0.79, 95% CI 0.63–0.99, p = 0.049); achantosis nigricans (OR 6.28, 95% CI 3.49–11.3, p = 0.02); and family history of type 2 diabetes (OR 1.37, 95% CI 1.05–1.78, p < 0.001). Self-reported color-race was not associated with metabolic syndrome. In the second model, predisposing factors were: female gender (OR 2.03, 95% CI 1.60–2.58, p < 0.001); age (OR 1.05, 95% CI 1.04–1.06, p < 0.001); achantosis nigricans (OR 6.37, 95% CI 3.51–11.6, p < 0.001); family history of type 2 diabetes (OR 1.37, 95% CI 1.05–1.78, p < 0.001); and European ancestry (OR 1.77, 95% CI 1.004–3.12, p = 0.048).

**Conclusions:** Predominant European ancestry may influence the presence of metabolic syndrome, in addition to traditional risk factors. Due to cross-sectional design of the study, we cannot stablish causal relationship. Further studies are necessary to confirm this association in other admixed populations.

**Financial support:** FAPERJ (1989.0246.5) and CNPq (563753/2010-2).

### P127 Genomic ancestry and self-reported color race in ckd in type 1 diabetes: a nationwide admixed sample of Brazilian patients

#### Marcela Haas Pizarro^1^, Deborah Conte Santos^1^, Laura Gomes Nunes de Melo^1^, Bianca Senger Vasconcelos Barros^1^, Luiza Harcar Muniz^1^, Luis Cristovão Porto^2^, Dayse Aparecida Silva^3^, Marilia Brito Gomes^1^

##### ^1^Universidade do Estado do Rio de Janeiro, Departamento de Medicina Interna, Diabetes, Rio de Janeiro, Brazil; ^2^Universidade do Estado do Rio de Janeiro, Laboratorio de Histocompatibilidade e Criopreservação (HLA), Rio de Janeiro, Brazil; ^3^Universidade do Estado do Rio de Janeiro, Laboratorio de Diagnostico DNA (LDD), Rio de Janeiro, Brazil

*Diabetology & Metabolic Syndrome* 2019, **11(Suppl 1):**P127

**Introduction:** Diabetic kidney disease is one of the most common microvascular complications in patients with type 1 diabetes. Historically, diabetic patients from ethnic groups, such as African-Americans, Hispanics, and Asians, have a higher chance of developing diabetic kidney disease when compared with Caucasians.

**Objective:** Evaluate the association between self-reported color race, genomic ancestry (GA), and the presence of chronic kidney disease (CKD), assessed by glomerular filtration rate and albuminuria in patients with type 1 diabetes.

**Methods:** This is a multicenter, observational, cross-sectional study with 1564 patients, conducted between August 2011 and August 2014 in 14 public clinics from 10 Brazilian cities. Race was evaluated as self-reported color race and GA (divided in European, African, and Amerindian). We used autosomal Ancestry Informative Markers to asses GA. We stratified patients into groups: normal renal function and CKD. Patients with normal renal function had a GFR >=60 ml/min and the absence of albuminuria. CKD was defined as a GFR < 60 ml/min and/or the presence of albuminuria. The study was approved by the ethics committee of our hospital and by the local ethics committee of each center.

**Results:** More patients self-declared themselves as black and brown in the group with CKD. The multivariate analysis did not find an association between any category of self-reported color race and the presence of CKD. Patients with CKD had a higher percentage of African (AFR) ancestry. However, a multivariate logistic regression between AFR ancestry and CKD did not confirm this association (p 0.064) after the adjustment for the confounding factors, such as Hba1c, hypertension, and social economic aspects. Patients with an AFR ancestry of 50% or higher had an association with CKD that did not persist after the multivariate analysis.

**Conclusions:** In our patients, from an admixed, multi-ethnic population, we did not find an association between self-reported color race, GA, and CKD. It is important to note that despite the fact that we didn’t find a significant p in the multivariate analysis concerning AFR ancestry and CKD, we found a narrow confidence interval (0.961–398) with an OR of 1.956. Further studies should be conducted to confirm the lack of association between AFR ancestry and CKD, especially from populations with higher African or Amerindian ancestries. Finanial support was granted by FAPERJ and CNPq.

### P128 Gestational diabetes mellitus: elaboration and validation of an e-book for self-care promotion

#### Fernanda Savoi Mendes^1^, Rosimeire Fernandes de Oliveira^1^, Alexandra Moreira Dias^2^, Janice Sepúlveda Reis^1^, Suelen Rosa de Oliveira^1^, Tatiane Géa Horta Murta^1^, Helem Sena Ribeiro^1^

##### ^1^Ensino e Pesquisa Santa Casa de Belo Horizonte, Belo Horizonte, Brazil; ^2^Escola de Enfermagem - Universidade Federal de Minas Gerais - UFMG, Belo Horizonte, Brazil

*Diabetology & Metabolic Syndrome* 2019, **11(Suppl 1):**P128

**Introduction:** The self-care of pregnant women with diabetes is of great importance since the glycemic control contributes to lower impacts on mother and child health.

**Objective:** the aim of this study was to develop and validate an *e*-*book* for pregnant women diagnosed with gestational diabetes mellitus (GDM) in order to promote self-care.

**Method:** this was a methodological study carried out in stages: definition of the theme and target patients, integrative literature review in Latin American and Caribbean Health Sciences (LILACS) databases, Nursing Databases (BDENF), Cumulative Index to Nursing & Allied Health Literature (CINAHL), Medical Literature Analysis and Retrieval System Online (MEDLINE); preparation of educational material; determination of readability and apprehensibility through the Flesch Reading Facility (FLF) test, the Flesh-Kincaid (FK) readability index and the Coleman Liau Index (CL); validation of the *e*-*book* by the judges’ committee through the *e*-*Surv* platform with subsequent calculation of the Content Validity Index (CVI); discussion with specialists between each stage; and validation of the *e*-*book* by the target patients through the face-to-face test.

**Results:** The final educational material entitled “Gestante Guide with Gestational Diabetes”, was composed of 32 pages with illustrations and explanatory text, according to the evaluation of 13 judges and obtained a mean CVI of 0.96 indicating clarity and relevance of the presented content. The material was adapted according to the suggestions and approval of the expert committee, made up of 6 professionals. The final text presented readability indexes FLF, FK and CL of 62.32, 4.43 and 11.96, respectively, being considered appropriate and clear to the target population. In response to the face-to-face test, the 10 interviewed pregnant women were satisfied with the material.

**Conclusion:** The *e*-*book* was prepared, adapted and validated according to the criteria of content and relevance. Its use by pregnant women diagnosed with GDM may contribute to their empowerment for better glycemic control and lower complications.

### P129 Gestational weight gain profile in women with gestational diabetes mellitus in a tertiary hospital

#### Fernanda Oliveira Braga, Raquel De Carvalho Abi-Abib, Carolina Alves Cabizuca, Elaine Ramalho Da Silva, Roberta Arnoldi Cobas, Roselee Pozzan

##### Universidade do Estado do Rio de Janeiro, Rio de Janeiro, Brazil

*Diabetology & Metabolic Syndrome* 2019, **11(Suppl 1):**P129

**Introduction:** The Institute of Medicine (IOM) recommends that pregnant women with pre-gestational body mass index (BMI) of 18.5 to 24.9 kg/m^2^ should gain 11 to 16 kg; those with BMI between 25.0 to 29.9 kg/m^2^ should gain 7 to 11.5 kg; and those with BMI ≥ 30 kg/m^2^ should gain 5 to 9 kg. Weight gain above or below recommendations is associated with adverse perinatal outcomes (ADOs). The aim of this study was to evaluate the factors associated with weight gain in pregnant women with gestational diabetes (GDM) and their association with ADOs.

**Methods:** This retrospective cohort study evaluated 129 pregnant women with GDM, followed at a tertiary hospitalfrom January 2014 to May 2019. Data were obtained from medical records. The pre-gestational BMI was calculated using the previous self- reported weight and the height obtained at the first visit. The final weight was obtained at the last visit (provided that it took place after the 36th week) or at admission for labor. The pregnant women were allocated into 3 groups according to adherence to weight gain recommendations: “below (G1), at target (G2) and above (G3) recommended”. The groups were compared in terms of income, educational status, ethnicity, gestational age at the first visit, treatment and ADOs (neonatal weight and neonatal hypoglycaemia).

**Results:** Of the 129 pregnant women evaluated, 39 (30.2%) had weight gain on target, 38 (29.5%) below and 52 (40.3%) above the recommendation. The weight gain was different according to the pre-gestational BMI classification (normal: 14.3 [8.1–17.4] kg, overweight: 9.9 [6.7–14.6] kg, obesity 1: 8.6 [4.6–14.0] kg, obesity 2: 7.9 [0–12.1] kg, obesity 3: 7.1 [4.2–11.0] kg; = 0.032). Family income was different among the groups studied (G1: 2.0 minimum wages [1.2–2.8], G2: 2.3 [1.8–3.6], G3: 1.7 [1.2–2 (G1: 12 [9–13], G2: 12 [11–12], G3: 11 [9–12]), as well as the number of years of study (p = 0.002 for comparison between G1 and G3). There was no difference between groups for the other parameters. There was a weak negative correlation between weight gain and years of school attendance (r = − 0.231, p = 0.009) and maternal age (r = − 0.218, p = 0.013).

**Conclusions:** Although the association between inadequate weight gain and greater risk of complications is already well established, only a minority of pregnant women in our center achieved what is recommended. Pregnant women with lower income and worse educational status seem to have more difficulty in achieving this goal.

### P130 Glycemic control in hypercaloric diet for hypertrophy

#### Felipe Silva Ferreira Mattos^1^, Barbara Oliveira Vieira^1^, Guilherme Silva Ferreira Mattos^2^, Lucas Silva Ferreira Mattos^3^

##### ^1^Centro Universitário de Caratinga (UNEC), Caratinga, Brazil; ^2^Centro Universitário Serra dos Órgãos (UNIFESO), Teresópolis, Brazil; ^3^Faculdade de Minas (Faminas), Belo Horizonte, Brazil

*Diabetology & Metabolic Syndrome* 2019, **11(Suppl 1):**P130

**Introduction:** Previous studies have shown that it is possible to control diabetes as long as it is combined with a good diet and following a doctor’s guidance. The disease can be controlled by prescribing a hypercaloric diet with a carbohydrate focus and plenty of exercise. Even with a higher intake of carbohydrate the patient did not show glycemic peaks or an alteration on glycated hemoglobin.

**Objective/goal/purpose:** To determine how a balanced diet and exercises associated with the FreeStyle Libre system can contribuite to the hyperglycemia control, weight loss and decrease of body fat.

**Method:** A patient with the objective of lean muscle gain and a decrease of body fat percentage was observed for a period of 4 months—10/18 a 02/19—by the nutrologist and endocrinologist by evaluating graphics from Libre and routine Bioimpedâncias. One to evaluate the control of glycemic index and the other to evaluate the gains and losses from the patient, but most of all to make sure it was the adequate treatment.

It was recommended to the patient that practices intense physical activity (patient practicing intense physical activity—met 6, 90 min duration, 6 times a week) 52 cal/kg/day, being 2.5 g/kg the protein requirement, 7 g/kg the carbohydrate requirement and the rest fat. The patient started the treatment with 72.8 kg and a body fat percentage of 18.7%, the diet was prescribed with 3786 kcal, being 182 g of protein, 509.6 g of carbohydrate and 113.27 g of fat.

**Result:** On the return for control on 12/13/2018 with A1c 5,7, LDL 147, weight 73,5, with the body fat percentage of 16,5%. Medication in use: Toujeo 26, Humalog (4 + 4+4 + 2) e Jardiance 25 mg. At the end of the treatment in February 2019 the patient showed an weight gain reaching 76.7 with a body fat percentage of 10.7%.

**Conclusion:** The hyperglycaemia in diabetics type 1 can be controlled as long as the right dosages utilizing the Libre equipment are followed. The equipment allows the patient to have access to the sugar index anywhere and anytime through a mobile facilitating the diabetes control. The improvement in the patient’s diet helped the overall weight gain with the exclusive loss of body fat and lean muscle gain. It’s valid to point out that the higher intake of carbohydrate did not change the previous treatment routine in any way.

Informed consent to publish had been obtained from the patient.

### P131 Glycemic control outcomes after a diabetes education program for type 1 diabetes patients

#### Ana Cristina Ravazzani De Almeida Faria^1^, Alexei Volaco^1^, Denise Beheregaray Kaplan^1^, Maria Augusta Karas Zella^2^, Denise Regina Baptista^3^, Luis Paulo Mascarenhas^3^, Nayanne Hevelin Dos Santos Oliveir^1^, Wiliam Coradin Cordeiro^1^

##### ^1^PUC PR, Curitiba, Brazil; ^2^Faculdade Evangelica Mackenzie de Curitba, Curitiba, Brazil; ^3^UFPR, Curitiba, Brazil

*Diabetology & Metabolic Syndrome* 2019, **11(Suppl 1):**P131

**Introduction:** Diabetes Mellitus (DM) is characterized by chronic hyperglycemia and is related to several complications. The incentive to diabetes self-care management is a fundamental tool in the diabetes treatment, because it empowers patients, improves adherence and drives to better glycemic control, minimizing the risk of complications.

**Objective:** To evaluate the glycemic control of patients with type 1 diabetes (DM1) enrolled in the State Insulin Analogues Supply Program before and after group diabetes education program.

**Methods:** DM1 patients enrolled in the State Insulin Analogues Supply Program were invited to participate in a three sessions diabetes education program performed by a multidisciplinary team. Data from 147 patients were collected: Glycated Hemoglobin A1c (HBA1c) from 1 year before to 1 year after the educational intervention. The HBA1c after the intervention was divided in HBA1c Post1, HBA1c Post 2, HBA1c post 3; whose intervals were about 3–4 months. The results were expressed as means. The Wilcoxon Test was applied to compare the averages.

**Results:** Of the 147 patients invited to the program, 75 attended at least two sessions and 57 to the three sessions. Data from patients who attended at least two sessions were analyzed. There was a reduction in the mean HBA1c of 1 year before compared with mean HBA1c of 1 year after the intervention from 8.8% to 8.5% (P < 0.032). When compared mean HBA1c pre intervention to HBA1c post 1, post 2 and post 3; there was a significant difference between the mean HBA1c pre and HBA1c Post 1 (8.8 × 8.4%) and Post 2 (8.8 × 8.5%) (p = 0.004 and 0.037 respectively), but not between the mean HBA1c pre and HBA1c Post 3 (8.8 × 8.7%) (p = 0.659).

**Conclusions:** This study demonstrated that a diabetes education program may be effective in improving glycemic control in DM1 patients. However, this effect is gradually lost in time, showing a need for continued education and a constant positive reinforcement in the feeling of encouraging and stimulating self-care in this population, seeking to prevent adverse outcomes and improvement in quality of life.

### P132 Glycemic impairment in chronic hepatitis c: does the vírus reduce b-cell function?

#### Camila Abdon^1^, Luciene Paes^1^, Amanda Kahwage^1^, Juarez Quaresma^2^, Fernando Flexa Ribeiro Filho^1^

##### ^1^Centro de Diabetes e Endocrinologia do Pará, Marabá, Brazil; ^2^Núcleo de Medicina Tropical - Universidade Federal do Pará

*Diabetology & Metabolic Syndrome* 2019, **11(Suppl 1):**P132

**Introduction:** In the course of chronic hepatitis C, extrahepatic manifestations are frequent, with important glycemic alterations, occurring approximately three times more than in the general population.

**Objective and methods:** To study this glycemic impairment in patients with Hepatitis C virus (HCV), we developed a cross-sectional and observational study with 72 patients living in State of Pará, Amazon region of Brazil, which has an endemic prevalence of HCV infection, both sexes, over 40 years of age, with a history of chronic infection by HCV and without co-infection with the Hepatitis B virus or HIV. Glycemic variables evaluated were fasting glycemia, glycemia after 2 h of overload with glucose, insulin, HbA1c, HOMA-B and HOMA-IR, relating them to aspects of liver disease. The fibrosis grade was estimated by the FIB-4 value (FIB-4 ≥ 3.25 vs < 3.25).

**Results:** Of the total of 72 participants, 52.8% were women. The mean age was 61.5 ± 8.4 years. Glycemic alterations were found in 40% of the patients: 26.4% were diabetic and 13.6% with pre-diabetes. The main genotype was type 1 (78%), followed by type 3 (22%). The glycemic variables behaved in a similar way among the genotypes. When we evaluated the glycemic profile according to the degree of fibrosis, patients with higher fibrosis (n = 17) presented a double prevalence of Diabetes Mellitus (41% vs 22%; p = 0.51) and higher 2 h glycemia (9.5 ± 4.5 mmol/L vs 6.8 ± 2.9 mmol/L, p = 0.028), however and in a contrary way than expected, caused by a lower HOMA-B (111.7 ± 63.4 vs 192.8 ± 182.4; p = 0.009), with equal levels of HOMA-IR, and without association with metabolic syndrome or obesity. Diabetic patients (n = 19) showed a tendency for a worse response to treatment, virological sustained response (SVR) was achieved in 80% of diabetics (vs 92.3% in non-diabetics, p > 0.05), especially those with genotype 3, that only 66% achieved SVR. Cirrhotic patients presented no more glycemic alterations than non-cirrhotic patients, however, diabetics showed a higher frequency of hepatocarcinoma compared to non-diabetics (10.5% vs 1.9%, p > 0.05). We conclude that the frequency of glycemic changes was high in the studied population, but without association with the traditional components of the metabolic syndrome and obesity.

**Conclusions:** HCV infection may compromise insulin secretion and that patients diagnosed with DM may require specific treatment because they present a poorer response to antiviral treatment and have greater chronic complications of the disease.

### P133 Glycemic level impairs blood pressure responses of diabetics submitted to exhaustion

#### Nathália De Souza Melo Abath Barbosa^1^, Iago Vilela Dantas^2^, Laryssa Guedes Da Silva Oliveira^1^, Amanda Renata Vicente Macedo^1^, Jonathan Nícolas Dos Santos Ribeiro^1^, Denise Maria Martins Vancea^1^, Cláudio Barnabé Dos Santos Cavalcanti^1^, Ricardo Azevedo Cavalcanti Aragão^1^

##### ^1^Universidade de Pernambuco, Recife, Brazil; ^2^Universidade Federal de Pernambuco, Recife, Brazil

*Diabetology & Metabolic Syndrome* 2019, **11(Suppl 1):**P133

**Introduction:** In normoglycemic individuals, blood pressure (BP) has a good relationship with physical exercise, varying according to the intensity and duration of the protocol. However, it is unclear whether blood glucose levels in any way alter the BP behavior of type 2 diabetics undergoing physical exertion.

**Objective:** To evaluate the relationship between different pre-exercise glycemic levels and the blood pressure response of type 2 diabetics during and after a maximal exercise stress test.

**Methods:** This quantitative and experimental study wasapproved by the Ethics Committee The subjects were allocated into four groups: non-diabetic, diabetic with glycemic values at the time of the test below 100 mg/dL, with blood glucose values between 100 to 200 mg/dL and diabetics with capillary blood glucose values above 200 mg/dL. The exercise stress test was performed in the morning. All subjects underwent the test using the modified Bruce protocol. Blood pressure was measured before, throughout the protocol and after the test and blood glucose was measured before and immediately after the test. Statistical analysis was performed using the SPSS for windows 16.0 program. The Shapiro–Wilk test was performed to analyze the normality of the data, where the Kruskal–Wallis test was chosen for nonparametric normality, adopting the significance level of p ≤ 0.05.

**Results:** The study included 67 type 2 diabetics of both sexes, aged 64.1 ± 1.9 years. It was observed that the group with glycemia above 200 mg/dL had a significantly increase in systolic pressure when compared to the other groups during all phases of the effort (178.2 ± 14.0 mg/dL vs 149.1 ± 14.4 mg/dL; 147.4 ± 13.7 mg/dL; 148.9 ± 11.1 mg/dL/p = 0.001) (193.1 ± 12.4 mg/dL vs 166.6 ± 10.5 mg/dL; 170.0 ± 10.0 mg/dL; 169.5 ± 10.1 mg/dL/p = 0.001) (204.2 ± 10.4 mg/dL vs 187.0 ± 8.6 mg/dL; 177.8 ± 18.4 mg/dL; 185.4 ± 10.3 mg/dL/p = 0.001), as in all recovery phases (192.3 ± 13.9 mg/dL vs 164.1 ± 10.3 mg/dL; 159.6 ± 16.5 mg/dL; 160.4 ± 16.7 mg/dL/p = 0.001) (175.2 ± 13.9 mg/dL vs 151.6 ± 12.8 mg/dL; 140.8 ± 8.6 mg/dL; 145.0 mg/dL ± 17.7 mg/dL/p = 0.001) (164.2 ± 10.4 mg/dL vs 134.5 ± 13.5 mg/dL; 132.5 ± 9.2 mg/dL; 133.5 ± 16.4 mg/dL/p = 0.001).

**Conclusion:** The main finding of the present study showed that diabetics with capillary blood glucose levels above 200 mg/dL before physical exertion may have an exacerbated rise in blood pressure during stimulation, as well as their impaired decrease at the end.

### P134 Glycemic variability before and after phototherapy in patients with type 1 diabetes mellitus and scleredema diabeticorum measured by continuous glucose monitoring system

#### Micaela Frasson Montero, Isabella Cristina Paliares, Mayra Zanon Casagrande, PatriciaMedici Dualib, Sergio Atala Dib, João Roberto de Sá

##### Escola Paulista de Medicina - Universidade Federal de São Paulo, São Paulo, Brazil

*Diabetology & Metabolic Syndrome* 2019, **11(Suppl 1):**P134

**Introduction:** ScleredemaDiabeticorum (SD) is a rare dermatological condition associated with chronic hyperglycemia and mainly described in type 2 diabetic patients (prevalence of 2.5%). It is characterized by skin thickening and tightening, with possible alteration of subcutaneous insulin absorption. Treatment includes intensive glycemic control, physical therapy, and recent studies suggest the use of phototherapy.

**Objective:** This study aims to evaluate the effect of phototherapy on glycemic variability in type 1 diabetes mellitus (T1DM) patients with SD through continuous glucose monitoring system (CGMS).

**Case report:** FSR, female, 33 years old, diagnosed with T1DM at 10 years old, on insulin since diagnosis, and long-standing poor glycemic control. At the age of 15, she noticed progressive and diffuse skin hardening, especially at the insulin application sites, with medication leakage after application. On physical examination BMI was 34 kg/m^2^, acanthosis nigricans in neck region, as well as diffuse skin thickening, especially in the neck, trunk, upper and lower limbs. The patient had high glycemic variability according to the CGMS, with time in range of 21.4% and estimated glycated hemoglobin (HbA1c) of 9.2%. Besides that, she reported asymptomatic hypoglycemia. It was hypothesized erratic absorption of subcutaneous insulin caused by the dermatopathy.

**Results:** The diagnosis of SD was confirmed by skin biopsy and the patient underwent 41 phototherapy sessions. Ten months later, the patient reported improvement of skin thickening and CGMS showed 48% of time in range and estimated HbA1c 7.8%, with reduction of hypoglycemic episodes.

**Discussion:** SD is a clinical and histopathological diagnosis, frequently ignored as a cause of glycemic decompensation in T1DM due to its lower occurrence. Its treatment lasts months, with frequent failure. Options such as phototherapy, radiotherapy, use of immunosuppressants and Tranilast have been reported only in case reports or series and retrospective studies. It was decided by phototherapy due to its lower morbidity.

**Conclusion:** The case reported illustrates the importance of investigating SD in patients with T1DM with high glycemic variability and skin hardening. Furthermore, it suggests phototherapy as an indicated treatment for SD with skin improvement and reduction of glycemic variability evaluated by CGMS.

Informed consent to publish had been obtained from the patient.

### P135 Health literacy and glycemic control in patients with diabetes: a tertiary care center study in Brazil

#### Luiza Harcar Muniz, Marilia Brito Gomes, Carlos Antonio Negrato, Paula de Campos Calassara, Ana Luiza Nicoletti Dias

##### Universidade Estadual do Rio de Janeiro, Rio de Janeiro, Brazil

*Diabetology & Metabolic Syndrome* 2019, **11(Suppl 1):**P135

**Introduction:** Diabetes is a chronic disease with a prevalence that varies widely throughout the world and is continuously increasing. Nowadays to reach and maintain a good glycemic control, is still a challenge in clinical practice, mainly among minorities. In Brazil, only 13.2% of patients with T1D [14] and less than 50% of patients with T2D presented HbA1c at goal.

**Objective:** The primary objective of our study was to determine which factors may influence health literacy (HL) in patients with type 1 diabetes (T1D) and type 2 diabetes (T2D), and the influence of HL on glycemic control.

**Methods:** This was a cross-sectional study with 347 patients (144 with T1D and 203 with T2D). Data were obtained from medical records and/or questionnaire. The short test of Functional Health Literacy (S-TOFHLA) was used to evaluate HL.

**Results:** More patients with T1D presented adequate HL [119 (82.6%) vs 87 (44.8%, p < 0.001)]. No difference was found in HbA1c levels according to S-TOFHLA. All T1D patients with HbA1c levels < 7.0% (53 mmol/mol) had adequate HL. A correlation between age and years of school attendance with S-TOFHLA score was observed in both groups. No correlation was observed between HbA1c levels and capillary glycemia at the beginning and at the end with all domains of S-TOFHLA. Although the p value of 0.07 found for DM1 patients who had Hb1Ac within standards and adequacy in S-TOFHLA is not statistically significant, it is probably clinically relevant because it was associated with a decrease in HbA1c of 0.5%.

**Conclusions:** A considerable number of patients with either T1D or T2D did not have adequate HL. In general, patients with T1D had a better performance. Overall, age and years of school attendance were the most important variables associated with better performance of S-TOFHLA. Patients with T1D who self-reported as White, that had more years of school attendance and presented an adequate HL had a better chance of reaching an adequate glycemic control. Finally, in addition to therapeutic regimens, an approach on diabetes management should also include patients’ HL evaluation along with psychological and social aspects.

### P136 Heart failure with preserved ejection fraction and atypical symptoms: differences among diabetic and non-diabetic patients

#### Francisco Alfredo Bandeira^1^, Marcela Resende Sarmento^1^, Elaine Karoline Costa da Silva^1^, Louise Rayra Alves Bezerra^1^, Jessica Myrian de Amorim Garcia^2^

##### ^1^Division of Endocrinology and Diabetes, Hospital AgamenonMagalhães, University of Pernambuco Medica, Recife, Brazil; ^2^Division of Cardiology, Hospital AgamenonMagalhães, Recife, Brazil

*Diabetology & Metabolic Syndrome* 2019, **11(Suppl 1):**P136

**Introduction:** The diagnosis of Heart Failure (HF) was present in less than 20% of patients recruited in cardiovascular outcomes studies with iSGLT2, but hospitalization for HF as a secondary outcome was as frequent as ischemic stroke (a component of the primary outcome). One possibility for this finding is that patients with diastolic dysfunction and elevated NT-proBNP without typical symptoms were not considered to have HF.

**Objective:** To evaluate echocardiographic findings and circulating NT-proBNP levels according to the presence of typical or atypical HF-related symptoms and metabolic changes in patients with and without DM.

**Methodology:** This is a cross-sectional cohort study with 78 patients (mean age 64.19 ± 13.85 years; BMI 27.58 ± 5.1 kg/m^2^; CA 92.1 ± 13.1 cm; SBP 136.1 ± 23.4 mmHg; DBP 83.9 ± 16.9 mmHg; estimated GFR: 77.5 ± 42.6 mL/min/1.73 m^2^; EF 53.2 ± 14.2%; final diastolic volume LV 53.3 ± 12.7 mL/m^2^; LV mass index 266.8 ± 155.3 g/m^2^; Left atrial volume index 42.2 ± 17.6 mL/m^2^; NT-proBNP 3699.7 ± 6574.5 pg/mL), of which 28 had HF with reduced EF and 50 had diastolic dysfunction, preserved EF and NT-proBNP > 125 pg/mL.

**Results:** Compared with the group of 28 patients with HF and reduced EF, the group with preserved EF had more DM (50.0% vs 21.4%; p = 0.013). Of the patients with preserved EF, 25 had DM and 25 did not have DM. The presence of diastolic dysfunction, NT-proBNP > 125 pg/mL and two or more atypical symptoms occurred in 58% of the preserved EF group without significant differences between DM and non-DM. Non-DM patients had more anorexia than DM patients: 32% vs 60%; p = 0.047. Although there were no significant differences regarding age, BMI, CA, DM patients had higher serum triglyceride levels than non-DM patients (311.2 ± 78.3 vs 123.5 ± 38.3 mg/dL; p = 0.033) and mean HbA1C of 7.85% ± 1, 8%. There was no significant difference between the group with reduced and preserved EF regarding age, gender and BMI.

**Conclusion:** A high prevalence of atypical symptoms in patients with diastolic dysfunction, preserved EF and increased blood NT-proBNP was found.

### P137 High diabetes distress was associated with increased glycemic variability, hyperglycemia, and ketoacidosis in adults with type 1 diabetes followed in a brazilian public tertiary hospital

#### Thais Barbarini Seabra Brasil^1^, Monica Sueli Vilela da Mota Silveira^1^, Walkyria Mara Gonçalves Volpini^2^, Elizabeth João Pavin^3^

##### ^1^Internal Medicine Postgraduate Program, Faculty of Medical Sciences, University of Campinas, Campinas, Brazil; ^2^Neuroimmunology Unit, Biology Institut, University of Campinas, Campinas, Brazil; ^3^Endocrinology Division, Department of Internal Medicine, University of Campinas, Campinas, Brazil

*Diabetology & Metabolic Syndrome* 2019, **11(Suppl 1):**P137

**Introduction and objective:** The management of type 1 diabetes (T1D) requires adherence to a complex daily and life-long treatment, as well as constant glycemic monitoring to achieve the recommended goals. Diabetes Distress (DD) may influence adherence to insulin treatment (IT) and glycemic control. This study aimed to identify possible associations between the scores obtained with the Type One Diabetes Distress Scale (T1DDS) Brazilian version, and the glycemic variability (GV) of adult patients with T1D followed in a Brazilian Tertiary Public Hospital.

**Methods:** The T1DDS questionnaire was applied to patients ≥ 18 years old, diagnosed with T1D for at least 6 months. It consists of 7 subscales that quantify the DD: S1: powerlessness, S2: management distress, S3: hypoglycemia distress, S4: negative social perception, S5: eating distress, S6: physician distress and S7: Family/friend distress. The patients were divided into two groups according to the DD levels: moderate and high (DDM/H) and low (DD/L). Morisky test was used for measure adherence to IT. All patients were instructed to perform 7-point glycemic profiles for 3 consecutive days, returning to download monitor data and calculation of GV, expressed as the standard deviation of the glycemic average in mg/dL. For statistical analysis, the following tests were used: Mann–Whitney, Chi Square, Fisher’s exact test and Spearman correlation. The adopted significance level was 5%. CEP 80881317.7.0000.5404.

**Results:** Of the 40 patients recruited, 70% were women, age: 37.1 ± 11.6 years, T1D time: 23.5 ± 9.2 years, HbA1c: 8.7 ± 1.8%, microvascular complications: 77.5% and DDM/H: 60%. GV: 69.1 ± 27.2 mg/dL (target < 50). Patients with higher S2 scores had higher GV (79.1 vs 57.9 mg/dL; p = 0.0282); higher mean blood glucose (352.9 vs 269 mg/dL; p = 0.0076) and more ketoacidoses per week in the last 2 months (0.68 vs 0.05; p = 0.0156). Higher S5 score were associated with lower body mass index (kg/m^2^), although classified as overweigth (25.5 vs 28.5; p = 0.0473).

**Conclusion:** DDM/H levels were high. There was an association between DD and T1DDS (S2) management and blood glucose instability, evaluated by the increase of the GV, more hyperglycemia and ketoacidoses. Psychological interventions to reduce DD, especially those related to self-management of T1D are fundamental, and may contribute to reduced GV, improving clinical outcomes and quality of life of this population.

**Financial support:** CAPES and FAEPEX.

### P138 High percentage of body fat in healthy non-obese young people

#### Larissa Luna Queiroz^1^, Patrícia Pinho Cardoso^1^, Luana Matos de Souza^1^, Flávio Weverton de Souza^2^, Myrella Messias de Albuquerque Martins^2^, Synara Cavalcante Lopes^2^, Renan Magalhães Montenegro Júnior^2^, Virgínia Oliveira Fernandes^2^

##### ^1^Programa de Pós Graduação de Saúde Pública -PPGSP/UFC, Fortaleza, Brazil; ^2^Universidade Federal do Ceará, Fortaleza, Brazil

*Diabetology & Metabolic Syndrome* 2019, **11(Suppl 1):**P138

**Introduction:** Increased cardiovascular risk such as metabolic syndrome (MS) has been attributed, especially in some populations with normal body mass index (BMI), to the peculiarities of body fat composition and distribution.

**Objetictive:** To evaluate anthropometric parameters, body fat percentage and MS parameters in non-obese women.

**Methods:** Cross-sectional and quantitative study, approved by the Ethics Committee (CAAE: 99568318.2.3001.5045), conducted with young women of childbearing age, non-obese, without comorbidities. Weight, height, BMI, waist circumference (WC) were measured, in addition to body composition by Biodynamic bioimpedance. Blood pressure was also measured and serum triglyceride, fasting glucose, total cholesterol (TC), and HDL cholesterol (HDL-c) levels were analyzed. The correlation between the variables was verified by the Spearman test, performed in the program Jamovi version 1.0.

**Results:** 156 volunteers were initially evaluated and the final sample consisted of 94 women, with a mean age of 29.4 years (± 7.14). The mean WC and BMI were 75.6 cm (± 7.96) and 24 kg/m^2^ (± 3.39) respectively. The percentage of body fat (PGC) was 31.8% (± 4.43), with a frequency of obesity by PGC of 68.5%. The mean systolic pressure (PS) was 117 mmHg (± 8.91) and the diastolic pressure 77.5 mmHg (± 8.65). As for laboratory parameters, the TC was 173 mg/dl (± 17.1), HDL-c 59.3 mg/dl (± 15.7), triglycerides of 86.4 mg/dl (± 74) and fasting glucose of 81.1 mg/dl (± 6.5). WC and PGC were negatively correlated with HDL (r = -− 0.5, p < 0.001) and (r = -− 0.4, p < 0.001) and positively with PS (r = 0.3, p = 0.003) and (r = 0.2, p = 0.04); and with CCe (r = 0.8, p < 0.001) and (r = 0.4, p < 0.001).

**Conclusions:** After all the research it was possible to conclude that in this population of healthy young, non-obese women with low WC values, a high frequency of body fat increase was observed by PGC. Under this circumstances it is important to extend our focus on BMI by considering other markers than WC, such as PGC or alternative anthropometric parameters in obese individuals, for early screening for metabolic obesity.

### P139 Hospitalizations for diabetes mellitus in the Brazilian unified health system from 2008 to 2019

#### Márcio Ribeiro Melhor, Mateus da Silva Santana, Eduardo Faria Soares de Magalhães, Rafael Costa Sarno Neves, Ananda Pires Bastos, Bianca Aparecida Colognese, Catherine Castelo Branco de Oliveira, Alexandre Machado Silva de Oliveira

##### Universidade Federal da Bahia (UFBA), Salvador, Brazil

*Diabetology & Metabolic Syndrome* 2019, **11(Suppl 1):**P139

**Introduction:** Diabetes mellitus (DM) is a severe health problem worldwide. In Brazil, the disease is still underdiagnosed and its complications represent one of the main death causes. It’s suggested that the study of hospitalization by DM in the Unified Health System (SUS) allows to reflect on its prevention and treatment policies in Brazil.

**Objective:** To describe, in Brazil and its regions, hospitalizations by diabetes *mellitus* in SUS between 2008 and May 2019 by quantity and hospitalization rates, quantity and death rates and in-hospital lethality rate.

**Methods:** This was an ecological study based on data from SUS’s Hospital Information System (SIH). Hospitalizations with diabetes mellitus as the main diagnose (categories E10 - E14 of the International Classification of Diseases, 10th Revision) and further hospital morbidity information from the SUS’s informatics department (DATASUS) were searched. For the hospitalization rates and death rates (by 10^4^ and 10^5^ people, respectively), population information from the Brazilian Geography and Statistics Institute (IBGE) was used.

**Results:** In total, 1.579.388 diabetes hospitalizations and 71.835 deaths were accounted for in Brazil. The North and Northeast regions were the only ones with raising hospitalization rates (20% and 17% respectively), where it was also noticed the highest hospitalization rates in 2018 (7.35/10^4^ people and 7.52/10^4^ people, respectively). The largest hospitalization rate reduction occurred in the Midwest region (40%) and the Southeast recorded the lowest rate in the studied period (5.23/10^4^ people). The Brazilian average hospital death rate was 3.17/10^5^ people, which, in 2018, showed a 12.5% reduction compared to 2008. In the regions, the biggest death rate comes from the Northeast (4.11/10^5^ people) and the smallest was from the Midwest (2.30/10^5^ people). The Northeast also had a bigger in-hospital lethality rate in that period (5.2%) when compared to the Midwest (3.0%), which was the smallest.

**Conclusions:** The data analysis showed the discrepancy between hospitalizations by DM in Brazil’s regions, especially in the Northeast region, which presented the highest death rate and in-hospital lethality rate and, along with the North region, was responsible for the biggest hospitalization rates in 2018. That difference emphasizes the necessity of a better population’s health coverage, especially in the most affected regions, to prevent disease progression, hospitalization and complications.

### P140 Hypercaloric diet effects on the reproductive outcome and fetal development of diabetic rats

#### Beatriz Martins Holtz^1^, Vanessa Caroline Araújo da Silva^1^, Alice Santos da Silva^1^, Andressa Silva Lourenço^1^, Thaigra de Sousa Soares^1^, Rafaianne Queiroz de Moraes-Souza^1^, Débora Cristina Damasceno^2^, Gustavo Tadeu Volpato^1^

##### ^1^FisioTox - UFMT/CUA, Araguaia, Brazil; ^2^LAPGO - UNESP/Botucatu, Botucatu, Brazil

*Diabetology & Metabolic Syndrome* 2019, **11(Suppl 1):**P140

**Introduction and objectives:** Diabetes during pregnancy causes to maternal–fetal complications. In addition, inadequate eating habits are the major factors for the development and progression of metabolic disorders. The objective of this study was to evaluate the maternal and fetal effects caused by mild diabetes and hypercaloric diet consumption before and during pregnancy.

**Methods:** Newborn female Wistar rats received citrate buffer (non-diabetic—ND) or streptozotocin (STZ) (diabetic—D) at the first day of life. At 90 days of life, Oral Glucose Tolerance Test (OGTT) was performed for inclusion and exclusion criteria of the experimental groups (n = 12 animals/group): ND with standard diet (ND) or with abnormal diet (NDA); Diabetic with standard diet (D) or with abnormal diet (DA). Experimental groups with abnormal diet received hyperlipid chow and water containing 5% sucrose. Subsequently, at 120 days of life, the rats were mated. On day 0 of pregnancy the Lee obesity index was performed, water and feed consumption, and corporal weight were weekly evaluated. On days 0 and 17 of pregnancy, these were submitted to OGTT. On the day 21 of pregnancy, the rats were anesthetized, and a laparotomy was performed for maternal reproductive evaluations. Visceral and periovarian fat were weighed. The fetuses were weighed and analyzed for anomalies analyses.

**Results:** The D and DA rats showed an increased area under the curve during the OGTT and pre and postimplantation losses in relation to the ND groups. The DA group presented increased caloric intake, visceral and periovarian fat, and reduced weight gain during pregnancy in relation to ND group. The offspring from the groups that received the hypercaloric diet presented a reduced fetal body weight, which is associated to intrauterine growth restriction. All groups showed a decreased percentage of fetuses without anomalies in relation to the ND group. The percentages of skeletal anomalies in the D and DA groups were higher than the ND. The association between diabetes and diet has increased visceral anomalies in relation to ND.

**Conclusion:** The glycemia and embryonic losses were exclusively caused by diabetes, but the abnormal fetal body weight was induced by diet. The association of these two factors changed the obesity biomarkers and fetal anomalies.

**Financial support:** CAPES/FAPEMAT.

### P141 I-SMART protocol on phone intervention: self-care practices in diabetes mellitus type 2

#### Laura Barbosa Nunes, Jéssica Caroline dos Santos, Heloísa de Carvalho Torres

##### Universidade Federal de Minas Gerais, Belo Horizonte, Brazil

*Diabetology & Metabolic Syndrome* 2019, **11(Suppl 1):**P141

**Introduction:** Type 2 diabetes mellitus (T2DM) is a chronic condition of multifactorial cause mainly associated with unhealthy lifestyle, such as physical inactivity and inadequate diet. The implementation of care plan is a self-management support strategy, being built according to individual preferences. The I-SMART protocol—smart plan for type 2 diabetes mellitus self-care phone intervention helps the user to choose what is important is to share goals and achievable actions.

**Objective:** To analyze the I-SMART protocol—smart plan in telephone intervention oriented towards self-care practices in type 2 diabetes mellitus.

**Methods:** Cross-sectional study conducted with 71 users with type 2 diabetes mellitus participating in the telephone intervention and linked to five Basic Health Units of Belo Horizonte, Minas Gerais, year 2019. Users received three telephone interventions in 6 months of follow-up. The I-SMART Protocol—My Smart Plan is comprised of six domains such as: motivation, specification, effort, scope, importance and time; and 11 questions. The protocol was applied through the e-suv platform via telephone call. The sociodemographic and clinical characteristics (Hb1Ac) were collected in a proper form through face-to-face interviews together with anthropometric variables (BMI). The data that made up the bank were grouped according to the frequency of responses and analyzed using absolute, relative (%), and central tendency (average) frequencies. All research ethics standards were met in accordance with National Health Council Resolution 466/12.

**Results:** The average age of the participants was 62 years old. Most were female (63.8%). The prevailing domain was the motivation that had as priority the follow-up of the plan to be fed (56.3%). Despite recognizing the importance of the continuity of the proposed plan, confidence in reach was lower, and the difficulties presented were associated with behavioral, psychosocial and cultural aspects (36.6%) and emotions (18.3%), such as sadness (14.1%) and anger (4.2%).

**Conclusions:** The I-SMART protocol oriented towards diabetes self-care practices favors the development of the care plan with short-term goals. Financial Support: CNPq 432824/2016; FAPEMIG APQ-03865-16.

### P142 IDEGLIRA (insulin degludec liraglutide combination) for the treatment of type 2 diabetes mellitus in real life

#### Walter Cardoso, Fábio Moura, Mirela Vasconcelos, Ana Carla Montenegro, Monica de Oliveira, Thais Gelenske, Erico Carvalho

##### IMIP - Instituto de Medicina Integral de Pernambuco, Recife, Brazil

*Diabetology & Metabolic Syndrome* 2019, **11(Suppl 1):**P142

**Introduction:** Achieving glycemic goals in the treatment of type 2 diabetic patients without causing harm or weight gain is challenging although several therapeutic modalities are available on the market. Combination of GLP-1 analogs with insulin analogs is a new option that may be useful in obtaining such outcomes.

**Objective:** To determine in a series of real life cases whether the clinical use of the IDegLira (Insulin Degludec Liraglutide combination) is feasible and effective in Brazilian patients.

**Methods:** A case series of 23 patients who started using IDegLira has been followed since introduction for 90 to 120 days. The patients were subdivided in three groups, based on previous treatment: oral drugs only, basal insulin plus oral drugs and multiple insulin doses. The starting dose was 10 units for patients without previous insulin use and 16 for those with previous insulin use. IDegLira dose was adjusted to achieve a fasting glycemia between 80 and 130 mg/dl. Clinical and laboratorial parameters were followed to determine effects on glycemic control, hypoglycemia risk, body weight and side effects. Hunger sensation was assessed through the hunger scale.

**Results:** At baseline, 58% were female, mean age was 62 and, 55% had been diagnosed with diabetes for over 10 years. 18/23 patients (78.2%) were able to maintain their medication; 3/23 (13%) stopped for financial reasons and 2/23 (8.7%) stopped for persistent nausea. The mean reduction in HbA1c was 0.75%, reaching 1.48% in patients with previous basal insulin use. Average weight loss was 5.3 kg, more pronounced in previous users of multiple insulin doses.. There was a 44% reduction in hypoglycemic events in patients previously treated with insulin, without increase in those on oral drugs only. Hunger sensation reduction occurred in 50% of patients. Average reduction in insulin dose was 18 units/day (2 units/day in those previously treated with basal insulin and 28 units/day in those treated with multiple doses).

**Conclusions:** In short term, IDegLira was effective in treating diabetic patients who had poor glycemic control. There was a reduction in HbA1c, frequency of hypoglycemia, body weight and hunger sensation. However, results were different due to previous treatment, with greater weight loss and reduction of hypoglycemia in patients previously treated with multiple insulin doses. Age, time since diagnosis and gender made no difference in the results. The smallnumberofpatientslimitstheconclusions.

### P143 Impact of A1c control on different insulin types and insulin regimens in patients with type 1 diabetes

#### Carlos Alexandre Rezende da Silva

##### Santa Casa de Misericórdia de São Paulo, São Paulo, Brazil

*Diabetology & Metabolic Syndrome* 2019, **11(Suppl 1):**P143

**Introduction:** Diabetes mellitus is not a single disease, but a heterogeneous group of metabolic disorders that presents hyperglycemia in common, resulting from defects in insulin secretion and/or action. In the treatment of type 1 diabetes (DM1), the use of insulin for glycemic control is essential. The classic Diabetes Control and Complications Trial (DCCT) study showed that intensive treatment of DM1 with 3 or more doses of insulin or continuous subcutaneous insulin infusion (CSII) was effective in glycemic control and decreased long-term microvascular complications.

**Objective:** To evaluate the glycated hemoglobin (A1C) in the different insulin therapy regimens (fixed dose regimen, flexible regimen, flexible carbohydrate and CSII regimen) and in the different insulin types (human insulin - NPH and regular -, use of some type of insulin analogue or CSII with exclusive use of insulin analogue).

**Methods:** This is an observational, nonrandomized cross-sectional study. Data were collected from patients with DM1 or LADA (latent adult diabetes mellitus). Data were collected from medical records of patients treated from March 2018 to August 2018 after approval of the project by the Research Ethics Committee of the Institution.

**Results:** The type of insulin prescribed was regular and NPH for 40.6% of the sample, with an average A1C of 9.0%, use of at least one type of insulin analogue 41% and an average A1C of 8.2% and CSII 16.3% of patients, with glycated hemoglobin of 7.8% and P = 0.017 and 95% CI when compared to the regular insulin/NPH and CSII therapies. When we evaluated the insulin therapy regimens and the influence on glycated hemoglobin, we observed that fixed insulin doses have A1C of 8.8%, flexible doses without carbohydrate counting, A1C of 9.0%, flexible doses with carbohydrate counting 8.0% and in CSII, 7.7% of A1c, being statistically significant (P = 0.037) the influence on A1C between the flexible and non-carbohydrate CSII insulin regimens.

**Conclusions:** Good glycemic control depends on new technologies, such as CSII, and analogues insulins that demand high costs in patient management and are not widely available in the Brazilian public health system.

### P144 Impact of diabetic distal polyneuropathy and diabetic cheiroartropathy in transplanted patients

#### Taynara Guedes da Silva^1^, Emanuel Davi Simões dos Santos^2^, Edson Bruno Vidal de Sousa^2^, Camylla Bandeira Miranda^2^, Cristiany Azevedo Martins^1^, Hortência Diniz Teixeira^2^, Taciana Benevides Rocha^1^, Daniela Gardano Bucharles Mont’Alverne^2^

##### ^1^Hospital Universitário Walter Cantídio - Universidade Federal do Ceará, Fortaleza, Brazil; ^2^Universidade Federal do Ceará, Fortaleza, Brazil

*Diabetology & Metabolic Syndrome* 2019, **11(Suppl 1):**P144

**Introduction:** The incidence of post-transplant DM, a secondary type of DM, has increased and ranges from 5 to 45%. However, the incidence of microvascular complications such as diabetic distal polyneuropathy (DPN) and diabetic cheiroarthropathy (DC) are not reported in the literature.

**Objective:** To verify the incidence of DPN and DC in patients undergoing kidney or liver transplantation.

**Methods:** A cross-sectional study of a quantitative approach was performed in kidney or liver transplant patients, regardless of gender, over 18 years. All participants were evaluated for age, gender, years of transplantation, time since diagnosis of DM, last HbA1c value. DPN was assessed by neuropathic symptom score (NSS) and neuropathic impairment score (NIS). The presence of the DC was obtained by the Prayer Sign test. Results were expressed as mean ± standard deviation and/or percentage. For associations, t-test was performed for independent samples, and for correlations, Pearson’s test or Chi square test was considered statistically significant when p ≤ 0.05.

**Results:** Sixty-two individuals were evaluated, of whom 37 (59.9%) underwent renal transplantation, most of them female (n = 32, 51.6%), with a mean age of 57 ± 11 years on evaluation. They had an average of 9 ± 5.5 years of transplantation and 5.7 ± 3.6 years of diagnosis of DM. The average HbA1c was 7.28 ± 1.8%. Of the 62 participants, 61.3% (n = 38) had PND, with 71% (n = 27) classified with DPN neuropathic pain, 15.8% (n = 6) with asymptomatic PND and 13.2% (n = 5) with painful PND. The prayer sign was present in 21 participants (33.9%). In the correlations, it was not observed influence of the years of transplantation or of the years of diagnosis of DM with the DPN or DC (p ≥ 0.05). The type of transplant also had no correlation with these variables (p ≥ 0.05). The prevalence ratio of those who have DPN to have DC in participants is 1.27.

**Conclusion:** A high incidence of DPN was observed in the participants. Although the incidence of DC was lower, it was still higher than described for patients with DM.

### P145 Impact of different bariatric surgical procedures on type 2 diabetes: results of one year follow-up

#### Mônica Raquel de Souza Aquino, Roberto Hangley Mendes Dantas Junior, Marcel Catão Ferreira dos Santos, Letícia Morais de Andrade, Victória Teixeira Leite, Vinicius Silva Costa, Daniel Coelho

##### Universidade Federal do Rio Grande do Norte, Natal, Brazil

*Diabetology & Metabolic Syndrome* 2019, **11(Suppl 1):**P145

**Introduction:** Bariatric and metabolic surgery is the most effective treatment for Type 2 Diabetes (DM2) among individuals who are affected by obesity and can result in remission or improvement in almost all cases. Techniques related to high diabetes remission rates (60–80%) include Roux-en-Y Gastric Bypass and Vertical Gastrectomy.

**Objective:** To compare weight loss and diabetes remission rates in patients operated by both techniques within a protocol.

**Methods:** It consists of a retrospective longitudinal study based on analysis of medical records of a tertiary hospital. Patients with DM2 were selected and had glucose and glycated hemoglobin (HbA1c) dosages in the pre-and post-operative period of 1 year. Comparisons were made between baseline results and 12 months after treatment. Statistical analyzes were performed with SPSS.

**Results:** A total of 97 patients were included in the study, 49 of whom underwent Roux-en-Y Gastric Bypass (GB) and 48 vertical gastrectomy (VG). The GB group before the procedure had fasting glucose [129 (106.5–150.0) vs. 99 (93.0–115.0) mg/dL, p < 0.001)] and HbA1c [7.1 (6.1–7.9) vs. 6.1 (5.5–6.7)%, p = 0.002)] higher in relation to the VG group. Furthermore, patients who were submitted to GB showed less HbA1c inferior to 7 (46.91% vs 89.6%, p < 0.001) and used more insulin (32.7% vs 4.2% p < 0.001). After 1 year, there was a statistically significant reduction in the IMC excess loss percentage in both techniques, weight [76.0 ± 19.83 vs. 79.1 ± 9.96 kg, p = 0.331)], IMC [29.49 (29.39–33.85) vs. 31.65 (24.40–34.55) kg/m^2^, p = 0.080), weight loss [28.9 (24.2–38.2) vs. 26.9 (20.5–33.5) kg, p = 0.092)]. However, on the opposite to the beginning of the study, the groups did not show differences regarding fasting glucose [88.0 (79.5–98.5) vs. 85.3 (77.0–94.8) mg/dL, p = 0.221)], HbA1c [5.4 (5.0–5.8) vs. 5.4 (5.0–5.7)%, p = 0.908)], percentage of patients who stopped insulin use (93.8% no BG e 100% na GV, p = 1.0), percentage of patients with HbA1c inferior to 7 (91.8% vs 97.9%, p = 0.362) and percentage of patients who reduced medication doses (97.8% vs 92.7, p = 0.344).

**Conclusions:** Surgical techniques are equally effective in type 2 DM remission and weight loss in 1 year, but these results support GB as the most indicated to achieve ideal weight.

### P146 Impact of switching from twice-daily (bid) basal insulin to once-daily (OD) insulin glargine 300 U/ml (GLA-300) in patients with type 1 diabetes (T1D)-phase 4 TOP1 trial, Brazilian cohort

#### Rosangela Roginski Rea^1^, Sérgio Atala Dib^2^, Maurício Aguiar de Paula^3^, Tiago Bonfim Catanho da SIlva^3^, Ana Cristina Truzzi^3^, Gustavo Akerman Augusto^4^

##### ^1^Serviço de Endocrinologia e Metabologia do Hospital de Clínicas da Universidade Federal do Paraná, Curitiba, Brazil; ^2^Universidade Federal de São Paulo (UNIFESP), São Paulo, Brazil; ^3^Sanofi, Votorantim, Brazil; ^4^CPQuali-SP, São Paulo, Brazil

*Diabetology & Metabolic Syndrome* 2019, **11(Suppl 1):**P146

**Introduction and objectives:** This study evaluated switch from BID basal insulin to OD Gla-300 therapy as part of a basal-bolus strategy.

**Methods and results:** The 28-week, multicenter top1 trial, conducted in Brazil, enrolled adults with T1D uncontrolled (HbA1c 7.5–10%) on BID basal insulin in combination with prandial rapid-acting insulin analogue for at least 1 year. Pts switched to Gla-300 in combination with a prandial rapid-acting insulin analog. The 123 pts (54.5% female) had a mean age of 37.0 ± 11.5 years, mean BMI of 26.3 ± 4.1 kg/m^2^, and mean T1D duration of 17.0 ± 9.5 years. Mean HbA1c and FPG at screening were 8.6 ± 0.7% and 201 ± 80.3 mg/dL, respectively. At baseline, the mean doses of basal insulin were 19.6 ± 10.4 U (AM) and 14.0 ± 7.1 U (PM), and 67% of pts were using insulin glargine 100 U/mL. The mean total dose of prandial insulin was 25.1 ± 13.2 U. There was no significant reduction in HbA1c from baseline to Week 24 (primary endpoint, p = 0.873). Fasting SMBG significantly decreased (175 ± 42 vs 156 ± 38 mg/dL from baseline to Week 24; p < 0.001), and significant decreases in glycemic variability (SMBG) were observed at various time points. There was a significant reduction from run-into the last 4 weeks on treatment in the proportion of pts with at least one hypoglycemic event (p = 0.025), as well as decreased numbers of hypoglycemic events/pt-year of any type, symptomatic, and confirmed ≤ 70 mg/dL (p = 0.036, 0.007, and 0.049, respectively), despite a minor increase in basal insulin dose (4.65 ± 0.9 U). The basal/prandial insulin dose ratio remained around 60/40%. There was a significant improvement between post-run-in and Week 24 in pt satisfaction as measured by the Diabetes Treatment Satisfaction Questionnaire total (24.8 ± 7.3 vs 30.7 ± 5.2; p < 0.001) and perceived hyperglycemia (4.0 ± 1.3 vs 3.0 ± 1.5; p < 0.001) scores, as well as significant decrease in concern with hypoglycemia and improved pt satisfaction with the number of injections.

**Conclusions:** In this population, switching from a BID basal insulin to an OD basal insulin, although not promoting significant reduction in HbA1c, brought significant reduction in SMBG and 8-point SMBG. These results were accompanied by a reduced incidence of hypoglycemia and increased pt satisfaction. The trial was sponsored by Sanofi.

### P147 Impact of the continuous insulin infusion system on quality of life and glycemic control of patients cared for in a federal district health unit

#### Álisson Duarte Martins, Brenda Miranda Aidar, André Neves Mascarenhas, Isabela Andrade Dutra de Resende, Nathália Ferigolo Trevisan Ribeiro

##### Escola Superior de Ciências da Saúde - Brasilia/DF, Brasília, Brazil

*Diabetology & Metabolic Syndrome* 2019, **11(Suppl 1):**P147

**Introduction:** Diabetes mellitus is a chronic and progressive disease characterized by high blood glucose levels. The Guideline Group has developed recommendations for the use of insulin in patients with type 2 diabetes for whom insulin is indicated and in the treatment of patients with type 1 diabetes. Thus, two methods of insulin administration are recommended, which is the regimen of insulin. multiple doses of insulin (MDI) or the Continuous Insulin Infusion System (SICI). The latter presenting, in recent years, better glycemic control and better quality of life for the patient.

**Objective:** To describe the impact of the Continuous Insulin Infusion System (SICI) on the quality of life and glycemic control of patients treated at a Health Unit of the Federal District.

**Methodology:** This is a cross-sectional and analytical study. The data analyzed refer to the use or not of ICS, quality of life of patients and glycated hemoglobin levels. Participants were divided into two groups, A and B, which belong to group A patients who use the SICI and group B those who do not use it, which is used as the study control group. For data collection, the Quality of Life Measure (DQOL) questionnaire, validated in Brazil, on quality of life was used. Statistical analysis was performed using the Statistical Package for Social Sciences-SPSS version 22. Data resulting from the application of the questionnaire were tested for normal distribution by the Kolmogorov–Smirnov test. Afterwards, Student’s t-test was applied to compare quantitative variables between the two groups.

**Results:** A total of 44 questionnaires were applied and 12 of them were answered by type 1 diabetic participants (DM1) who used the Continuous Insulin Infusion System (ICS), categorized as group A, and the others, 32, were answered. DM1 participants who do not use the device, categorized as group B. Group A obtained a higher index in the domain “Satisfaction” compared to group B, with p = 0.016, as satisfaction in time spent in controlling diabetes, apprehension that the diabetes causes in the family and flexibility in the diet. In the “Impact”, “Social/Vocational Concerns” and “Diabetes Concerns” domains, it was uncomfortable to have diabetes, lose your job and worry about passing out, with group B being more troubled and concerned about these issues. However, these three domains were not statistically relevant when comparing the groups, with p values > 0.05. Regarding glycated hemoglobin control, participants in group A obtained an average of 7.25% of glycated hemoglobin, with values ​​ranging from 6.0% to 9.1%, while those in group B obtained an average of 8.35%. with values ​​ranging from 6.1% to 12%, with p = 0.004.

**Conclusion:** The study patients who use the Continuous Infusion Pump have better quality of life and better control of glycated hemoglobin levels compared to those who do not use the device.

### P148 Impact of the kind of glycemic monitoring method at quality of life in patients with diabetes mellitus type1

#### Clara Simony De Sousa Santos, Pâmela Chaves De Jesus, Glauciane Freire Dos Santos, Lysandro Pinto Borges, Mariana Garcez Varela, Naira Horta Melo, Karla Freire Rezende, Carla Raquel Pereira Oliveira

##### Universidade Federal de Sergipe, Aracajú, Brazil

*Diabetology & Metabolic Syndrome* 2019, **11(Suppl 1):**P148

**Introduction:** Diagnosis of type 1 diabetes (DM1) demands lifestyle changes, multiple doses insulin injections or insulin pump and self-monitoring, which is conventionally performed by multiple digital punctures. These conditions, especially the latter, may impair health-related quality of life (HRQoL) in patients with T1DM.

**Objective:** Evaluate the impact of using a continuous glucose monitoring sensor at quality of life in DM1 patients users of unified health system (SUS) and adapted with conventional self-monitoring.

**Methods:** Prospective cohort study of 28 patients with DM1 SUS users (16 women, 31.5 ± 10.5 years, 1.62 ± 0.1 m, 61.1 ± 11.9 kg, BMI of 23.3 ± 4.5 kg/m^2^ and diagnosis of DM1 14 ± 8.3 years ago) answered a questionnaire to evaluate HRQoL, the DQOL-Brazil, before and 3 months after the use of FreeStyle^®^ Libre, a subcutaneous flash sensor, exchanged every 15 days. The questionnaire consists of 44 questions divided into 4 domains: satisfaction, impact, social/vocational concerns (SVC) and diabetes related concerns (DMRC). Answers range from 1 to 5, with 1 being the best rating. Questions were individually analyzed and calculated: Overall mean score (OMS); sum of the answers divided by the number of questions answered; and average scores of the 4 domains, following the same calculation. Data will be expressed as mean ± standard deviation, statistical analysis was performed in IBPM SPSS version 2.0 software using paired T test and considered significant p < 0.05. This study was approved by ethics committee (CAAE: 14555719.7.0000.5546).

**Results:** There was no change in OMS before and after using the sensor (2.52 ± 0.47 and 2.57 ± 0.66; p = 0.574, respectively), nor in the satisfaction (2.37 ± 0, 57 and 2.32 ± 0.71; p = 0.661), impact (2.59 ± 0.6 and 2.55 ± 0.73; p = 0.724), SVC (2.52 ± 0.87 and 2.73 ± 1.16; p = 0.291) and DMRC scores (2.81 ± 1.11 and 3.03 ± 0.92; p = 0.324). Only two questions showed significant improvement: satisfaction “with the time spent checking glucose levels” (3.11 ± 1.40 and 1.86 ± 0.89; p = 0.00) and “with current treatment” (2.07 ± 1.28 and 1.36 ± 0.62; p = 0.01).

**Conclusion:** The exchange of the glycemic monitoring method by multiple digital punctures to continuous monitoring sensor did not improve the quality of life of patients DM1 SUS dependent’s. However, they were more satisfied with the glycemic monitoring and treatment.

**Support:** Sensors provided by abott.

### P149 Impact of the structured care program for individuals with diabetes mellitus type 1 using a continuous subcutaneous insulin infusion (CSII) in Brazilian unified health system (SUS): 6 month follow up

#### Fernando Flexa Ribeiro Filho, Fernanda Laredo Dos Santos, Lara Gonçalves Ostuzzi, Raquel Almeida Cunha

##### Centro de Diabetes e Endocrinologia do Pará - CEDEPA, Belém, Brazil

*Diabetology & Metabolic Syndrome* 2019, **11(Suppl 1):**P149

**Introduction:** CSII is the most advanced form of insulin administration, but it requires structured education to provide the users with the necessary knowledge/skills and to support their motivation. With it advanced features, CSII offers significant advantages over the traditional therapy, but at higher costs. Individuals with diabetes may have misunderstandings about the capabilities of this therapy leading to negative emotional reactions. Structured diabetes education has been recognized as an integral component of diabetes therapy for decades and has been integrated into the treatment guidelines.

**Objective:** To evaluate the impact of the first semester after the Structured Education Program for CSII users, at a SUS Specialized Center.

**Method:** From September 2018 to March 2019, we followed the progress of 44 patients treated at a SUS Specialized Center in the Standardized Structured Education Program, specifically developed for CSII users, providing the necessary skills and knowledge for the effective use of this resource and dealing with Psychological barriers to enhance the beneficial effects of the therapy, based on a self-management approach that incorporates clinical, technological, and psychosocial components focused on global multidisciplinary educational follow-up, with the production of clinical and administrative reporting to assist the medical decision-making.

**Result:** 57.14% women, average age 23 years, ranging from 5 to 48 years, with 64.4% between 05 and 24 years. We found a variation of 3 and 40 and 1 and 35 years in age and time of diagnosis, respectively. The averages of the glycemic values in the target ranges (time in range—TIR) for values < 54 mg/dL ranged from 3.7 to 2.1%; 55–69 mg/dL ranged from 7.87 to 5.9%; 70–180 mg/dL ranged from 46.2 to 58.07; 181–249 mg/dL that ranged from 17.27 to 16.8; > 250 mg/dL ranged from 24.67 to 18.9. The mean A1C ranged from 8.6% (5.8% to 12.1%) to 7.7% (5.5 and 10.8%).

**Conclusion:** Reduction of 0.9% in A1C, with improvement of the TIR, increase in the range of 70–180 and decrease in the other ranges. Clinical benefits and cost-effectiveness are only possible with the care structuring, trained professionals and a skilled user to make appropriate decisions, managing problems, assessing and acting based on blood glucose. The education plays a relevant role in the therapy, particularly for CSII users. A constant monitoring is required to obtain lasting improvement.

### P150 Impact of type 2 diabetes on cognitive decline

#### Ana Cristina Ravazzani De Almeida Faria, Alexei Volaco, Clara Inacio De Paiva, Aline Maciel Gouveia, Launy Fraga Da Silva, Andre Mondin Nogueira, Cassio Slompo Ramos, Salma Ali El Chab Parolin

##### PUC Parana, Curitiba, Brazil

*Diabetology & Metabolic Syndrome* 2019, **11(Suppl 1):**P150

**Introduction:** Type 2Diabetes Mellitus (DM2), as well as dementia, are diseases of high prevalence, incidence, morbidity and mortality. Patients with DM2 have 50% higher risk of having dementia, which indicates that cognitive decline (CD) can be considered another complication of diabetes.

**Objective:** To Evaluate and quantify the prevalence of cognitive dysfunctions in a population of DM2 patients.

**Methods:** It is a cross-sectional observational study, with DM 2 patients from the outpatient clinic of a terciary hospital at Curitiba-PR. The exclusion criteria were: illiteracy, visual and/or auditory impairment, history of previous neurological diseases and/or use of medications that change cognition. Patients were submitted to anamnesis, physical examination, cognitive tests (Mini Mental State Exam, Semantic Verbal Fluency Test, Trail Making Test A and B and Word Memory Test) and PHQ-9 questionnaire (screening for depression symptoms). The medical records data were used to confirm the previous morbid history, use of medications and laboratory exams. The linear regression analysis of the cognitive tests was performed with all clinical and laboratory variables. The P values with significance level were < 0.05.

**Results:** 219 patients were enrolled, 135 (61.6%) female and 84 (38.4%) male. The mean age was 61.6 (± 10.1) years old and the mean diabetes duration was 13.4 (± 10.5) years. In a linear regression model analysis, a positive correlation was observed between the diabetes duration and the Trail Making Test A (p = 0.011), even when adjusted for age, schooling and history of severe hypoglycemia, as well as relation of the presence of macular edema and the Trail Making Test A (P = 0.015), even when adjusted for age, schooling, diabetes duration and history of severe hypoglycemia. For the Trail Making Test B, the linear regression model analysis showed a positive correlation with the history of severe hypoglycemia (P = 0.021), even when adjusted for age, schooling and diabetes duration. Other variables did not correlate with the cognitive tests in this sample.

**Conclusions:** Diabetes duration, history of severe hypoglycemia and presence of macular edema were markers of negative impact on the executive function evaluated by the Trail Making Test A and B in these DM 2 patients. These findings could interfere at self-care in this population. Because there is no specific treatment for cognitive dysfunctions so far, it is of extremely importance prevent diabetes in the general population to reduce and/or postpone the impact of DM on cognition.

### P151 Impacts of a diabetes clinical programme

#### Marcela Alves Teixeira Furtado^1^, Gladys Villas Boas do Prado Melo^1^, Camila Yumi Senaga^1^, Pedro Gabriel Melo Barros e Silva^3^, José Paulo Abi Jaudi^2^, Raquel da Silva Balduíno^3^, Viviane Aparecida Fernandes^3^, Valter Furlan^3^

##### ^1^Amil EspaçoSaúde Jardins, São Paulo, Brazil; ^2^United Health Group, São Paulo, Brazil; ^3^Hospital SamaritanoPaulista, São Paulo, Brazil

*Diabetology & Metabolic Syndrome* 2019, **11(Suppl 1):**P151

**Introduction:** Diabetes is a health issue that represents a significant impact for patients, health systems and job market. Besides, can cause loss in quality of life, disabilities and premature death. Therefore, building efficient strategies for prevention, diagnosis and treatment associated with education for self-care is essential to minimize the consequences of diabetes. Previous studies discuss forms of care approach but usually not associated with health costs.

**Objective:** Evaluate the clinical impact and use of health system before and after the inclusion in a Diabetes Clinical Programme from a Private Outpatient Care Institution in Sao Paulo, Brazil.

**Methods:** Evaluate patients before and after their inclusion in a Programme based on transdisciplinary care. In this regard, the time before and after was set as follows: Time 0 (T0): patient’s inclusion in the Programme; 6 months: comparison 6 months before and after T0; 12 months: comparison 12 months before and after T0. The indicators were: health expenditure, hospitalization, emergency room visits and glycated haemoglobin (A1c).

**Results:** Comparisons at 6 months shows reduction of 30.2% in heath expenditure (p < 0.05); whilst at 12 months the reduction drops to 9.4% (p < 0.05). Concerning the number of hospitalization per 1.000 patients included in the Programme, the 6 months data shows reduction of 35.4% (p < 0.01) and the 12 months period indicates decrease of only 18.6% (p < 0.05). Emergency room visits were down to 14.9% in 6 months (p < 0.01) and 9.5% in 12 months (p < 0.05). Considering A1c levels, both after 6 or 12 months, there was an increase of 27% of patients in the target defined by the patient’s physician which represents 50% of all patients. That was the most sustained index. The analysis of the data demonstrates the effectiveness of the programme indicating that more access to health care professionals and education represent greater treatment adherence. After 12 months the effect on the drop of results was reduced likely due to loss of strength in the initial approach that could have been kept by means of telemonitoring resources.

**Conclusion:** such programme, with transdiciplinaridade approach, clinical assistance and education have positive results in patients’ health and impact in health care expenses aggregating value in health care. However, it’s crucial to associate a way to maintain constant contact with patients in order to sustain efficacy.

### P152 Impairment on placental and fetus caused by high fat diet intake

#### Josilene Lopes de Oliveira, Ana Paula Varela Sanches, Bruna de Souza Lima, Maíra Schuchter Ferreira, Laís Angélica de Paula Simino, Josiane Erica Miyamoto, Marciane Ferreira Milanski, Letícia Martins Ignácio de Souza

##### Universidade Estadual de Campinas, Campinas, Brazil

*Diabetology & Metabolic Syndrome* 2019, **11(Suppl 1):**P152

**Introduction:** Nutritional status of mother can affect not only maternal metabolic adaptations in pregnancy, but can also program the fetus while interfering with individual gene expression, which can range from changes in biomolecular functions to permanent hormonal changes, since it happens during embryonic and fetal development, that is, a critical period where tissues and organs are being formed.

**Objectives:** To verify the effect of maternal weight gain on fetal placental development.

**Methodology:** Swiss female mice have been divided into two groups, the first has been received a control diet(CD), while the second received a high-fat diet(HF). The diets have been implemented throughout four weeks, before mating and until the 19th day of gestation when the 19 animals were euthanized. Before and during pregnancy, body weight parameters were evaluated, and placental and fetal parameters were euthanized.

**Results:** Pre-gestational weight gain correlated inversely with placental weight in group HF (p = 0.01) while there was no difference in CD, gestational weight gain was directly related to placental weight in group HF (p = 0.01) and CD (p = 0.001), these data show that diet influences differently the period before and during pregnancy, indicating possible impairment in placental development, when weight gain occurs before pregnancy.

Together with these results, our study also showed that the lower the placental weight, the lower the fetal weight in group HF (p = 0.001) and there was no change in CD. The positive correlation between placenta and fetus may show that the passage of nutrients was made differently according to the mother’s nutritional status/diet. Studies show that the weight of the fetus is lower when the placenta is smaller, this may be due to the fact that exposure to the inflammatory environment, which may cause placental insufficiency and decreased nutrient transport, consequently impairing fetal development. To verify possible changes in these parameters, we performed gene analysis through RNA-seq and found a difference in the expression of placental transporters, group HF differed from group CD in one glucose transporter isoform, two fatty acid transporter isoforms and six amino acid transporter isoforms, this confirms that nutrient passage may have occurred differently in groups CD and HF.

**Conclusion:** Excessive pre-gestational weight gain negatively affects the fetal outcome by causing impairment of placental development.

**Financial support:** CAPES.

### P153 Improved glycemic control with sulfonylurea in mody, after genetic diagnosis of a new mutation

#### Marilia Chaves Bernardo^1^, Mariana Fuganti De Souza^1^, Larissa Gava Ziviani^1^, Camila Vicente Dos Santos^1^, Lucia Henriques Alves Da Silva^1^, Roberta Magalhaes Tarantino^2^, Gabriella De Medeiros Abreu^3^, Rosane Kupfer^1^

##### ^1^Instituto Estadual de Diabetes e Endocrinologia Luiz Capriglione, Rio de Janeiro, Brazil; ^2^Universidade Federal do Rio de Janeiro, Rio de Janeiro, Brazil; ^3^Fundação Oswaldo Cruz, Rio de Janeiro, Brazil

*Diabetology & Metabolic Syndrome* 2019, **11(Suppl 1):**P153

**Introduction:** MODY (Maturity Onset Diabetes of the Young), the main type of monogenic diabetes, is secondary to an inherited mutation in a given gene, with a worldwide estimate prevalence of 2%. It is characterized by early onset, usually before 25 years of age, absence of insulin requirement in the early years (persistence of peptide C) and inheritance (predominantly autosomal dominant), and should compromise at least two generations of the same family, in addition to absence of insulin resistance markers. Mutations in the GCK and HNF1A genes are the most common types, but 14 types of MODY have been described so far. Sulphonylurea is the medication of choice when dietary therapy is insufficient to maintain normoglycemia.

**Case report:** A 49-year-old female patient with hypertension, dyslipidemia, overweight and a diagnosis of DM at 15 years of age, initially treated with oral antidiabetic agents, progressed to insulin therapy 10 years ago. She presents diabetic retinopathy, without other chronic or acute complications. His mother, brother and two children are diabetic. Their children diagnosed DM before 20 years of age and they don`t need insulin therapy. In the laboratory investigation, she presented C-peptide dosage of 2.4 ng/mL (VR > 0.8) and negative anti-GAD antibody. Based on the mentioned clinical criteria and strong family history, a HNF1A Sanger sequencing was performed and confirmed pathogenic mutation in exon 2. This is a nonsense mutation (p.Tir163Ter; c.489C > G) that creates a stop codon at position 163 of protein. The mutation was still seen segregating into one of the patient’s diabetic children. Because it has not yet been reported in the literature, the Mutation Taster program was used for analysis, which considered a pathogenic mutation, suggesting that the transcription factor HNF1A may have its function compromised by the presence of this variation. Sulfonylurea was introduced, with a reduction in total insulin dose (from 1.39 U/kg to 0.36 U/kg in 9 days) and an excellent glycemic control.

**Conclusion:** Genetic testing confirmed the diagnosis and made possible the discovery of a new pathogenic mutation. There are benefits in identifying MODY for patients and their families, leading to more specific therapy and enabling genetic counseling.

Informed consent to publish had been obtained from the patient.

### P154 Incidence and persistence of diabetic peripheral neuropathy after bariatric surgery

#### Otto Henrique Nienov, Fernanda Dapper Machado, Helena Schmid

##### Programa de Pós-Graduação em Ciências da Saúde: Ginecologia e Obstetrícia - UFRGS, Porto Alegre, Brazil

*Diabetology & Metabolic Syndrome* 2019, **11(Suppl 1):**P154

**Introduction:** Type 2 diabetes mellitus (T2DM) shows remission in more than 80% of subjects undergoing bariatric surgery (BS) but the impact of BS on the incidence and progression DPN is unclear.

**Objective:** To evaluate incidence or persistence of DPN in severe obese diabetic subjects submitted to laparoscopic BS.

**Methods:** In this prospective cohort study, 93 subjects with T2DM undergoing laparoscopic BS, Roux-en-Y gastric bypass (RYGB) or sleeve gastrectomy (SG), were evaluated for DPN by the Michigan Neuropathy Screening Instrument (MNSI) before and after 6 months of BS and divided according to presence (+) or absence (−) of DPN at baseline. Subjects with peripheral neuropathy from other causes like decompensated hypothyroidism, vitamin B12 deficiency and alcoholism, were excluded.

**Results:** The prevalence of pre-BS DPN was 34.0% (n = 18) and decreased significantly to 11.3% (n = 6) post-BS. There was no difference between RYGB and SG in DPN prevalence’s before (36.4% versus 30.0%, p = 0.861) and after 6 months of BS (7.5% versus 3.8%, p = 1.000). The prevalence of post-BS DPN was independently associated with higher fasting glycaemia (127.5 mg/dL versus 84.5 mg/dL; PR 1.018, 95% CI 1.005–1.030, p = 0.039). When we looked to the two groups, DPN (+) and DPN (−), from baseline to 6 months we saw: (1) for the DPN (+) group before BS (n = 18) the persistence of DPN (post-BS DPN) was 22.2% (n = 4) and, (2) for the DPN (−) group (n = 35) the incidence of post-BS DPN was 3.8% (n = 2).

**Conclusion:** DPN prevalence decreased after 6 months of BS; DPN incidence after BS was low.

### P155 Incidence and severity of diabetic polyneuropathy and diabetic cheiroartropathy in patients after renal and hepatic transplantation

#### Georgia de Melo Castro Gondim^1^, Emanuel Davi Simões dos Santos^2^, Edson Bruno Vidal de Sousa^2^, Camylla Bandeira Miranda^2^, Cristiany Azevedo Martins^2^, Hortência Diniz Teixeira^2^, Taciana Benevides Rocha^2^, Daniela Gardano Bucharles Mont’Alverne^2^

##### ^1^Hospital Universitário Walter Cantídio - Universidade Federal do Ceará, Fortaleza, Brazil; ^2^Universidade Federal do Ceará, Fortaleza, Brazil

*Diabetology & Metabolic Syndrome* 2019, **11(Suppl 1):**P155

**Introduction:** Microvascular complications of diabetes mellitus (DM), such as diabetic polyneuropathy (DPN) and diabetic cheiroarthropathy (DC), have increased. However, little is said about the incidence and severity of these complications in diabetic patients with kidney or liver transplanted.

**Objective:** To verify the incidence and severity of DPN and DC in diabetic patients who underwent kidney or liver transplantation.

**Methods**: A cross-sectional study of a quantitative approach was performed in kidney or liver transplantation patients, regardless of gender, over 18 years. All participants were evaluated for age, gender, years of transplantation, time since diagnosis of DM, last glycated hemoglobin (HbA1c) value. DPN was assessed by neuropathic symptom score (NSS) and neuropathic impairment score (NIS). The presence of cheiroarthropathy was observed by the Prayer Sign test. Results were expressed as mean ± standard deviation and/or percentage. For associations, t-test was performed for independent samples and, for correlations, Pearson’s Chi square was used. The test was considered statistically significant when p ≤ 0.05.

**Results:** 53 individuals were evaluated, of which 34 were men (64.2%), with a mean age of 59.3 ± 9.6 years, the mean DM years of 16.6 ± 8 years and mean transplant years of 4.7 ± 4.2 years. The mean HbA1c was 6.2 ± 2.6%, and most had undergone kidney transplantation, instead of liver (n = 35, 66%). Among the 53 participants, 83% (n = 44) had DPN, with 47.7% (n = 21) most specifically classified as DPN with neuropathic pain, 11.4% (n = 5) asymptomatic DPN, 25% (n = 11) DPN with risk of ulceration and 15.9% (n = 7) with painful DPN. The prayer sign was present in 29 participants (54.7%). In the correlations, it was not observed influence of the years of transplantation or of the years of diagnosis of DM in DPN or in DC (p ≥ 0.05). The type of transplant and age also did not correlate with these variables (p ≥ 0.05). The prevalence ratio of participants with DPN who also have DC was 0.78.

**Conclusion:** A high incidence of DPN was observed in participants, with more than 40% presenting severe DPN, with a higher risk of ulceration. Although the incidence of DC was lower than DPN, it was high, demonstrating a elevated functional limitation in the studied population.

### P156 Incidence of diabetes and related factors in the first 2-years postpartum among women with gestational diabetes-the Linda-Brasil study

#### Cristina Dickie de Castilhos^1^, Ruben Ladwig^1^, Cristina Figueiredo Sampaio Facanha^2^, Bianca de Almeida Pititto^3^, Lenita Zajdenverg^4^, Leony Morgana Galliano^1^, Bruce Bartholow Duncan^1^, Maria Inês Schmidt^1^

##### ^1^Postgraduate Program in Epidemiology, Hospital de Clínicas de Porto Alegre, UFRGS, RS, Porto Alegre, Brazil; ^2^School of Medicine, Unichristus, Fortaleza, CE, Fortaleza, Brazil; ^3^School ofPublic Health, Universidade de São Paulo, SP, São Paulo Brazil; ^4^Maternity School, Universidade Federal do Rio de Janeiro, RJ, Rio de Janeiro, Brazil

*Diabetology & Metabolic Syndrome* 2019, **11(Suppl 1):**P156

**Background:** Type 2 diabetes is growing in epidemic proportions and GDM is a strong risk factor for early development of diabetes among women.

**Objective:** To investigate the main predictors of diabetes development up to two years post-partum among women with recent GDM participating in the LINDA-Brasil cohort.

**Methods:** We recruited potential participants with GDM from prenatal care centers in Porto Alegre, Pelotas, Fortaleza, São Paulo and Rio de Janeiro to assemble the LINDA-Brasil cohort. Post-partum follow-up was carried out to assess the development of diabetes, ascertained by OGTT. We estimated relative risks and 95% CIs, and area under the ROC curve (AUC) for diabetes postpartum through logistic regression.

**Results:** We recruited 4846 women with GDM and 1955 underwent OGTT testing within 2 years postpartum. Mean (SD) pre-pregnancy BMI was 30.2 (6.5) kg/m^2^ and mean age, 31.7 (6.3) years; 891 (21.9%) used insulin during pregnancy, an additional 1310 (27.0%) oral hypoglycemic agents (OHAs). During follow-up, 242 (12%) developed diabetes. Factors associated with the development of diabetes at post-partum were: insulin use during pregnancy (RR 4.37; 95%CI 3.29–5.80); use of oral hypoglycemics during pregnancy (RR 2.66; 95%CI 2.01–3.53; one standard deviation change in 2 h plasma glucose at diagnosis (RR 1.93; 95%CI 1.67–2.23); one standard deviation change in fasting plasma glucose at diagnosis (RR 1.49; 95%CI 1.39–1.62); and one standard deviation change in pre-pregnancy BMI (RR 1.41; 95%CI 1.25–1.60). The inclusion of all these variables in the model yielded an AUC of 0.80 (0.753–0.846).

**Conclusions:** With simple clinical or administrative information we can effectively prioritize screening and counselling for diabetes prevention in the initial years following pregnancy complicated by GDM.

### P157 Increased expression of nephrin, podocin and wilms tumor 1 released in urinary extracellular vesicles in preeclampsia and diabetes mellitus

#### Karla Simone Costa de Souza^1^, Manuela Miguelia Bezerra da Silva^1^, Rita Nykassia Pinheiro Santos^1^, Tatiana Xavier da Costa^2^, Ricardo Ney Oliveira Cobucci^2^, Rand Randall Martins^3^, Adriana Augusto de Rezende^1^, Marcela Abbott Galvão Ururahy^1^

##### ^1^Department of ClinicalandToxicologicalAnalyses, Federal Universityof Rio Grande do Norte, Natal, Brazil; ^2^Maternity School Januário Cicco, Federal Universityof Rio Grande do Norte, Natal, Brazil; ^3^Department ofPharmacy, Federal University of Rio Grande do Norte, Natal, Brazil

*Diabetology & Metabolic Syndrome* 2019, **11(Suppl 1):**P157

**Introduction:** Patients with *Diabetes mellitus* (DM) and preeclampsia (PE) are at a higher risk of developing renal injury. This injury is characterized by occlusion of capillary lumens, glomerular endothelial swelling, and loss of podocytes. Over the last decade, evidence has increasingly spotlighted podocyte injury in DM and PE. Thus, evaluating the expression of podocyte-specific proteins, such as nephrin, podocin and Wilms Tumor 1 (WT1), in urinary extracellular vesicles (VEus), would contribute to identify potential biomarkers of kidney injury in DM and PE.

**Objective:** To evaluate the expression profile of nephrin, podocin and WT1 proteins released in VEus from patients with DM and PE, in order to investigate the potential role of these proteins as biomarkers of kidney injury.

**Methods:** From Jan. to Dec. 2018, 10 pregnant women with DM and PE (Case Group) and 10 normoglycemic and normotensive pregnant women (Control Group) were included in the study (Ethics Number: 1.942.794). Fasting blood samples were used to evaluate the patients’ overall metabolic status. First morning urine samples were collected to isolate uEVs by ultracentrifugation. Nephrin, podocin and WT1 expressions were evaluated by Western-blot. Nephrin, podocin and WT1 bands density were normalized by urinary creatinine.

**Results:** Increased nephrin/creatinine, podocin/creatinine and WT1/creatinine ratios were found in Case Group when compared to Control (p = 0.012; p = 0.015; p < 0.001, respectively). In the ROC analysis, high AUROC (area under the ROC) values for prediction of albuminuria were observed for nephrin/creatinine (AUROC = 0.813; p = 0.021), podocin creatinine (AUROC = 0.844; p = 0.011) and WT1/creatinine (AUROC = 0.979; p < 0.001) ratios. Patients’ overall metabolic status were compatible with the described for women with DM and PE.

**Conclusions:** This study is the first to show increased expression of nephrin, podocin and WT1 released in uEVs in patients with DM and PE. These results associated with the ones obtained in the ROC analysis suggest that nephrin, podocin and WT1 from uEVs are potential marker of kidney injury in DM and PE.

**Financial support:** CNPq.

### P158 Influence of diabetes mellitus on oxaliplatin induced peripheral sensory neuropathy in mice

#### Luiz Airton Paulino Soares^1^, Lus Mário da Silva Pereira^1^, Roberto César Pereira Lima Júnior^2^, Jonh Washington Cavalcante^1^, Anamaria Falcão Pereira^2^, Renata Bessa Pontes^2^, Paulo Roberto Carvalho de Almeida^2^, Mariana Lima Vale^2^

##### ^1^Hospital Universitário Walter Cantídio, Fortaleza, Brazil; ^2^Universidade Federal do Ceará, Fortaleza, Brazil

*Diabetology & Metabolic Syndrome* 2019, **11(Suppl 1):**P158

**Introduction:** Retrospective clinical studies have shown no significant statistical difference between diabetic and non-diabetic patients to present peripheral sensory neuropathy (PSN) using cumulative doses of oxaliplatin (OXL). These studies had limitations on the decreased number of patients investigated, difficulty to obtain a control group compatible with the treated group and exclusion of patients with initial degrees of neuropathy.

**Objective:** To verify whether a pre-existing condition of diabetes mellitus (DM) could influence the onset or course of OXL-induced PSN in mice.

**Methods**: DM and PSN were each induced with alloxan (50 mg/kg, iv) in a single dose and OXL (4.0 mg/kg, iv) twice weekly for 4.5 weeks in mice, respectively. The animals were evaluated weekly for 35 or 56 days, N = 8. Nociceptive tests (Von Frey and rota-rod), glycemia dosages and weight evaluation were performed. The tests were performed before and after the treatments of the animals weekly or biweekly. The experimental protocols were performed according to the guidelines of the animal use ethics committee of the Faculty of Medicine of the Federal University of Ceará and approved under number 27/2012.

**Results:** Alloxan (50 mg/kg, iv) was the best dose capable of inducing DM in animals without causing neuropathy or altering the reaction time in animals in the rota-rod. OXL (4.0 mg/kg, iv) was the best dose able to induce PSN in animals without altering the motor coordination of the animals in the rota-rod. The combination of treatments with alloxan and OXL induced DM with hyperglycemia and weight loss in animals significantly (p < 0.05) compared to the control and OXL groups. OXL treatment induced PSN from the 28th day in the animals. In addition, the combination of alloxan and OXL treatments in addition to anticipating the PSN from the 28th day to the 21st day also amplified the establishment of the PSN in the animals compared to the OXL group. Analysis of the pancreas of the animals treated with alloxan and combined with OXL showed a decrease in the number of islets of Langerhans besides the presence of areas with intense basophilia and acinar cells with basophilic cytoplasm and disarrangement of the glandular architecture.

**Conclusion:** It was demonstrated that mice with DM associated with alloxan-induced hyperglycemia both anticipated and amplified the development of PSN when treated with OXL. The present study renovates perspectives for the understanding of OXL-induced PSN in new studies with diabetic patients.

**Financial support:** CAPES.

### P159 Influence of diabetic distal polyneuropathy on ankle movement wide, quality of life and self-care in plantar ulcera patients

#### Cristiany Azevedo Martins^1^, Camylla Bandeira Miranda^1^, Hortência Diniz Teixeira^1^, Geyse Gomes de Oliveira^1^, Priscila Sampaio Silva^2^, Taynara Guedes da Silva^1^, Daniela GardanoBucharles Mont’Alverne^2^, José Carlos Tatmatsu Rocha^2^

##### ^1^Hospital Universitário Walter Cantídeo -UFC, Fortaleza, Brazil; ^2^Universidade Federal do Ceará, Fortaleza, Brazil

*Diabetology & Metabolic Syndrome* 2019, **11(Suppl 1):**P159

**Introduction:** Diabetic distal polyneuropathy (PND) can lead to trophic changes in the skin and osteoarticular structure of the foot, leading to the so-called diabetic foot. In addition, it is known that muscle weakness and decreased range of motion lead to a higher risk for the development of plantar ulcers in this population.

**Objective:** To verify the range of motion (ROM) of the feet, and its relationship with diabetic distal polyneuropathy (PND) quality of life and diabetes self-care in patients with plantar ulcer.

**Methods:** An observational, cross-sectional, comparative and quantitative study conducted at a Fortaleza Health Post in the first half of 2018. The PND Diagnostic Scale was applied, in addition to the Diabetes Quality of Life Measurement Questionnaire and the Health Activity Questionnaire. Self Care in Diabetes. The ankle and foot ROMs were evaluated using a manual goniometer for dorsiflexion, plantar flexion, inversion and eversion movements, and a descriptive percentage analysis was performed.

**Results:** Seventeen individuals diagnosed with PND were recruited, 47.1% men, with a mean age of 64 ± 10 years and disease duration of 18.6 ± 8 years. When comparing the ROMs found with predicted values, a reduction was observed in all movements (27% and 31% plantar flexion, 32% and 30% dorsiflexion, 34% and 36% inversion and 19% and 22% eversion, right and left, respectively). No correlation was found between ROM and signs and symptoms of PND, as well as quality of life and self-care.

**Conclusions:** A reduction in the feet joint amplitude values ​​was evidenced, being the inversion the most lost movement by the participants. The reduction in ROM seems not to influence the most severe signs and symptoms of PND, nor the quality of life and self-care of diabetic patients.

### P160 Influence of diabetic neuropathy on balance and mobility of patients with type 2 diabetes mellitus

#### Georgia de Melo Castro Gondim^1^, Francisca Janiele Ribeiro Tavares^2^, Pedro Miguel Afonso de Almeida e Silva^2^, Wescley Franco Lima^2^, Julia Maria Sales Bedê^2^, Ana Caroline Costa da Silva^2^, José Carlos Tatmatsu Rocha^2^, Daniela GardanoBucharles Mont’Alverne^2^

##### ^1^Hospital Universitário Walter Cantídio - Universidade Federal do Ceará, Fortaleza, Brazil; ^2^Universidade Federal do Ceará, Fortaleza, Brazil

*Diabetology & Metabolic Syndrome* 2019, **11(Suppl 1):**P160

**Introduction:** Diabetes Mellitus (DM) may lead to reduced functionality, sensitivity and muscle strength of the lower limbs in patients with diabetic polyneuropathy (DPN), a condition commonly found in this population. It is also known that diabetic patients show significant reduction in mobility and balance.

**Objective:** To evaluate the influence of DPN on balance and mobility of patients with DM type 2.

**Methods:** A cross-sectional study with a quantitative approach was performed in patients with type 2 DM, of both sexes, aged over 18 years. All participants were evaluated for demographic, anthropometric and clinical data, last fasting blood glucose values as well as last glycated hemoglobin (HbA1c) values, and the Diabetic Distal Polyneuropathy Diagnostic Scale, in which the Neuropathic Symptom Score (NSN) and the neuropathic impairment score (NIS) were evaluated. Balance and mobility were assessed by the Timed Up and Go (TUG) test. The reference values for TUG were compared with those found in the study. Results were expressed as mean ± standard deviation and/or percentage. For associations, t-test was performed for independent samples and, for correlations, Pearson’s Chi square test was performed, being considered statistically significant p ≤ 0.05. The research was approved by the Research Ethics Committee of the Federal University of Ceará, under opinion 2,251,159.

**Results:** 36 patients participated in the study, with a mean age of 62.4 ± 11 years and a predominance of females (n = 21; 58.3%). They had a mean age of diagnosis of 16 ± 8 years, with a last average glycemia of 189.7 ± 96 mg/dL, and a mean HbA1c of 7.88 ± 1.56%. The average time of TUG was 9.6 ± 3 s. Among the 36 patients, 31 (86%) had DPN. When comparing the TUG time with the presence or absence of DPN, no statistical difference was evidenced (p = 0.187). However, when comparing the TUG values with the reference values, a statistically significant reduction in the values found was observed (TUG predicted 8.5 ± 0.8 s; TUG found 9.6 ± 3 s; p = 0.032).

**Conclusion:** Patients with DM2 had higher TUG values than the expected for the population, and no influence of DPN on TUG values was observed.

### P161 Influence of diabetic polineuropathy on the functional capacity of diabetic individuals

#### Taynara Guedes da Silva^1^, Mirella Mac Links de Macedo^2^, Julia Maria Sales Bedê^2^, Ana Caroline Costa da Silva^2^, Débora Fidélis de Oliveira^2^, Pedro Miguel Afonso de Almeida e Silva^2^, José Carlos Tatmatsu Rocha^2^, Daniela GardanoBucharles Mont’Alverne^2^

##### ^1^Hospital Universitário Walter Cantídio - Universidade Federal do Ceará, Fortaleza, Brazil; ^2^Universidade Federal do Ceará, Fortaleza, Brazil

*Diabetology & Metabolic Syndrome* 2019, **11(Suppl 1):**P161

**Introduction:** Diabetes Mellitus (DM) can lead to diabetic polyneuropathy (DPN) that can interfere with the functionality of individuals.

**Objective:** To verify the influence of DPN on the functional capacity of diabetic individuals.

**Methods:** A cross-sectional study and quantitative approach was performed in DM-independent patients over 18 years of age. All participants were assessed for demographic (age, gender), anthropometric, clinical (time since diagnosis, last fasting blood glucose, last HbA1c values), reported lower limb pain (pain analogue scale), Scale for diagnosis of DPN where the neuropathic symptom score (NSS) and neuropathic impairment score (NIS) and the 6-minute walk test (6MWT) were evaluated. Results were expressed as mean ± standard deviation and/or percentage. T-test was performed to compare the value found with the predicted in the 6MWT. For the correlations, Pearson’s test was performed, being considered as statistically significant when p ≤ 0.05.

**Results:** Thirty patients with DPN participated in the study, being 90% (n = 27) type 2 DM, with a mean age of 60 ± 11 years and predominance for males (n = 16; 53.3%). They had a mean HbA1c of 8.1 ± 1.7%, with a mean daytime blood glucose of 175.8 ± 71 mg/dL. A statistically significant reduction of 26% was observed between the distance covered in the test and the distance expected by the participants (355.26 ± 98.41 meters, 480.10 ± 90.34 meters, respectively. P = 0.0001). Vital signs of heart rate and blood pressure remained within normal range at the end of the test. Correlations showed an inverse correlation between age and fatigue reported in the lower limbs at the end of the test (Borg lower limbs) (r = -,531 p = 0.003). In the other correlations, no statistical differences were evidenced.

**Conclusions:** It was found a reduction in functional capacity in diabetic patients with DPN. It was also observed that the older the age, the lower the fatigue in the lower limbs.

### P162 Influence of emotional distress on treatment adherence for diabetes mellitus

#### Reijane Mara Pinheiro Queiroz, Marta Maria França Fonteles, Nirla Rodrigues Romero, Vitoria Pessoa de Farias Cabral, joshua Levi Maia Magalhães, Lara Regis do Nascimento

##### Universidade Federal do Ceará, Fortaleza, Brazil

*Diabetology & Metabolic Syndrome* 2019, **11(Suppl 1):**162

**Introduction:** The emotional distress experienced by people with diabetes can influence adherence to pharmacological treatment, leading to difficulties in controlling the disease and risk of complications.

**Objective:** The objective of this study was to evaluate the influence of the emotional distress level in relation to medication adherence in two different treatment lines for diabetes mellitus.

**Methods:** A quantitative cross-sectional design carried out in a random sample of 157 participants with diabetes treated in primary care from March to June 2018. Emotional distress was measured using the Diabetes Distress Scale (DDS); adherence was measured by the Measurement of Adherence to Treatment instrument validated for oral antidiabetics (OADs; MAT_OAD_), and for Insulin (MAT_INSULIN_). The prevalence ratio (PR) and the Chi squared test (X^2^) between the variables were calculated to test the association between stress and adherence, considering statistical significance when p < 0.05. The study was approved by the Research Ethics Committee under opinion no. CAAE 86293118.3.0000.5054.

**Results:** All participants used OADs (N = 157). After applying the DDS and the MAT_OAD_ measuring instruments, it was observed that 17.9% (n = 5) presented “normal stress level” and low adherence, and 82.1% (n = 23) indicated “attention-level stress” with low adherence to oral treatment, thus demonstrating that emotional distress for people using OADs can be an important influencing variable in adherence to oral treatment (PR = 11.9 CI: 4.2–33.6, X^2^ = 28.8 (p < 0.01). In the same sample, 38 participants also used insulin and when applying the MAT_INSULIN_ insulin adherence instrument, it was observed that 23.6% (n = 9) of them did not adhere to treatment, 55.6% (n = 5) among those who did not adhere to treatment presented “normal stress level” and the remaining 44.4% (n = 4) presented “attention-level stress”. In the association analysis between stress and insulin adhesion, there was no difference in proportion (PR = 0.85 CI: 0.19–3.85; X^2^ = 0.04, (p = 0.8).

**Conclusion:** Stress levels can influence up to 11.9-fold adherence to treatment in people taking oral antidiabetic drugs. In addition, it was observed that participants in use of OADs have a higher prevalence of low adherence when it presents high stress level.

**Financial support:** *CAPES*, *CNPQ*, *PIBIC AND PREX*.

### P163 Influence of overweight in metabolic parameters in a cohort of patients with type 1 diabetes

#### Ana Carolina Andrade Mota, Bruno Fernandes de Sousa, Vinicius Silva Costa, Josivan Gomes de Lima, André Gustavo Pires de Sousa, Adriana Bezerra Nunes

##### Universidade Federal do Rio Grande do Norte, Natal, Brazil

*Diabetology & Metabolic Syndrome* 2019, **11(Suppl 1):**P163

**Introduction:** Approximately 30 thousand Brazilians live with type 1 diabetes mellitus (T1D). There is a consolidated relationship between type 2 diabetes mellitus (T2D) and other metabolic syndrome-related parameters since insulin resistance, classically reported in T2D, is one of the components keys of the syndrome. What has been increasingly studied is the role of insulin resistance in T1D and its subsequent relationship with metabolic syndrome.

**Objective:** To determine a correlation between T1D glycemic control and deleterious metabolic parameters.

**Methods:** It is a longitudinal associative study that has as participants patients with T1D, followed at an outpatient clinic of endocrinology of a University Hospital. The following patients’ data were collected: age, body mass index (BMI), glycated hemoglobin (HbA1c), fasting plasma glucose (FPG), total cholesterol (TC), high-density lipoprotein (HDL), low-density lipoprotein (LDL) and triglycerides (TG). It was used Pearson correlation coefficient (r) and for TG data, the Spearman correlation coefficient (rs). Statistics were pre-configured at a confidence interval (CI) of 95% and significance of p ≤ 0.05.

**Results:** The 67 patients with T1D included 38 females (56.7%) and 29 males (43.7%). The mean age was 24.7 ± 16.2 years and the mean age at diagnosis was 15 ± 7.5 years. The ethnic identification was based mostly on skin color: brown color with 51 patients (78.4%), followed by white population with 13 patients (20%) and the black color with 1 patient (1.6%). The mean values of BMI, HbA1c, FPG, TC, HDL, LDL and TG were respectively, 23.3; 9.2%; 214.5; 183.7; 47.9; 112.3; 111.3. According to statistical analysis, there is a correlation between higher BMI and higher FPG (95% CI; p < 0.045; r > -0.291) and higher BMI and higher HbA1c (95% CI; p < 0.04; r > -0.261). Also, there was a correlation between HbA1c and TG, in which poor glycemic control was associated with higher TG (95% CI; p < 0.007; rs < 0.396). However, there was not an association between BMI and TG (95% CI; p < 0.299; rs > 0.109) and neither between BMI and the HDL data (95% CI; p < 0.078; r > -0.234).

**Conclusions:** The present study establishes a correlation between increased BMI (overweight) and poor control of FPG and HbA1c in T1D. Also, poor glycemic control was associated with high levels of TG. In conclusion, deleterious metabolic parameters related to metabolic syndrome investigated in this study are associated with poor glycemic control in patients with T1D.

### P164 Influence of the time of diagnosis of type 1 diabetes mellitus on glycemic control of patients accompanied by a specialized outpatient clinic

#### Bruna Pereira Padilha, Daniella Almeida Brito, Jakeline Silva Santos, Leonardo Scandolara Junior, Adriana Paula da Silva, Elvi Cristina Rojas Fonseca, Heloisa Marcelina Cunha, Maria de Fátima Borges

##### Universidade Federal do TriânguloMineiro, Uberaba, Brazil

*Diabetology & Metabolic Syndrome* 2019, **11(Suppl 1):**P164

**Introduction:** Type 1 Diabetes Mellitus (DM1) is a chronic autoimmune disease that causes the destruction of beta-pancreatic cells responsible for insulin production. Individuals who do not adopt adequate therapeutic measures may be affected by progression of chronic complications, such as coronary microvascular disease and macrovascular disease, as well as the risk of acute complications such as hypoglycemia. With regard to the treatment, a vital aspect of the treatment is the consumption of balanced diets, insulin therapy, regular exercise and blood glucose monitoring.

**Objectives:** To establish a correlation between glycemic control and their time of diagnosis.

**Methods:** The study included 115 patients with DM1 assisted at the diabetes outpatient clinic of the city of Uberaba from May 2017 to March 2018. The patients were divided into 2 groups: children and adolescents (n = 59) and adults (n = 56). The study was approved by the Research Ethics Committee.

**Results:** No correlations were observed between the time since the diagnosis with the variables fasting plasma glucose (FPG), postprandial glucose (PPG), glycated hemoglobin (HbA1c) and fructosamine in the total patient sample. However, in the group of children/adolescents there were direct and significant correlations between the time of diagnosis with FPG (r = 0.375; p = 0.003); PPG (r = 0.489; p < 0.0001); HbA1c (r = 0.355; p = 0.006) and fructosamine (r = 0.350; p = 0.007). In the adult group, an inverse correlation with PPG was found (r = -0.289; p = 0.031).

**Conclusion:** While it did not occur in adults, the worsening of the glycemic control in children and adolescents with the progression of the disease may be related to factors, such as the inclusion of young people in the social environment, decreased active participation of family members in glycemic control, and increased hormonal influences. The influence of these factors must be considered in medical practice.

### P165 Insomnia, depression and other sleep disturbances in gestational diabetes

#### Mariana Salles Ballalai^1^, Victória Sudário de Alencar^2^, Cristina Figueiredo Sampaio Façanha^3^, Veralice Meireles Sales de Bruin^3^, Rejane Belchior Lima Macedo^4^, Adriana Costa Forti^4^, Thainá Lima Facó^5^

##### ^1^Universidade Federal do Ceará, Faculdade de Medicina; Bolsista CNPq, Fortaleza, Brazil; ^2^Centro Universitário Christus, Faculdade de Medicina, Fortaleza, Brazil; ^3^Universidade Federal do Ceará, UFC, Faculdade de Medicina; PPGMC, Fortaleza, Brazil; ^4^Centro Integrado de Diabetes e Hipertensão (CIDH) do Ceará, Fortaleza, Brazil; ^5^Centro Universitário Christus, Faculdade de Enfermagem, Fortaleza, Brazil

*Diabetology & Metabolic Syndrome* 2019, **11(Suppl 1):**P165

**Introduction:** Gestational diabetes (GDM) and sleep disturbances are frequent disruptors of pregnancy. Both conditions are related to depression and activation of the stress system, and in that way, associated with increased risk of poor outcomes, like pre-term birth. Among the sleep complaints related to pregnancy, poor sleep quality and insomnia, defined by difficulty initiating, maintaining or an unrefreshing sleep are the most important ones. Unfortunately, this issue has not been fully evaluated on GDM.

**Objectives:** This study aims to investigate the prevalence of insomnia in GDM pregnancies and its relationship with depression and other sleep disturbances.

**Methods:** A cross-sectional study, with a convenience sample interviewed 240 GDM pregnant women attending a public healthcare unity in the northeastern Brazil. Insomnia was defined by a score ≥ 15 in the Insomnia Severity Index (ISI) score. Sleep quality defined with Pittsburgh Sleep Quality Index (PSQI) score > 5. Daytime sleepiness with Epworth Sleepiness Scale (ESS) score ≥ 10; Depression with Edinburgh Postnatal Depression Scale (EPDS), validated in pregnancy > 12 and Fatigue, defined by Fatigue Severity Score (FSS) > 28. The study was approved by Instituto para o Desenvolvimento da EducaçãoLtda-IPADE ethic board, approval number 1.801.860. The statistical analysis was performed by software IBM SPSS Statistics.

**Results:** During the second half of pregnancy, 240 women with GDM, with mean age of 33.27 and mean BMI of 32.3 were evaluated. Poor sleep quality was found in 64.4% (PSQI:7.5 ± 3.7); insomnia in 16.4% (ISI:8.43 ± 5.8); Depression in 27.6% (EPDS:8.04 ± 6) Daytime sleepiness in 42.1% (ESS:9.8 ± 4.5) and Fatigue in 58.5% (35.13 ± 15). Short sleep was observed in 54.7%, with mean sleep duration of 6.4 ± 1.9. As expected, insomnia score was significantly related to PSQI score (p:0.000), Depression (P:0.000), daytime somnolence (p:0.000) and unexpectedly with Fatigue (p:0.001).

**Conclusion:** Sleep disturbances and depression were frequent conditions observed in these large group of GDM women. Insomnia was positively related with depression, poor sleep quality, daytime somnolence and Fatigue. As these conditions are associated with poor pregnancy outcomes on normal pregnancy, we suggest it might be important to investigate these conditions as a routine procedure during pre-natal visit of GDM patients.

### P166 Inspiratory muscle training on glucose control in type 2 diabetes: randomized clinical trial

#### Mariana Bruto de Pinto^1^, Patricia Martins Bock^1^, Andressa Silveira de Oliveira Schein^1^, Juliana Portes^2^, Raíssa Borges Monteiro^3^, Beatriz D^1^

##### ^1^Universidade Federal do rio Grande do Sul (UFRGS), Porto Alegre, Brazil; ^2^Centro Universitário Metodista (IPA), Porto Alegre, Brazil; ^3^Universidade do vale do Rio dos Sinos (UNISINOS), São Leopoldo, Brazil

*Diabetology & Metabolic Syndrome* 2019, **11(Suppl 1):**P166

**Introduction:** Inspiratory muscle training has been used in several clinical situations, and may be an alternative for individuals with type 2 diabetes mellitus who have difficulties in performing conventional exercises.

**Objective:** To test the hypothesis that inspiratory muscle training would improve glucose control and respiratory muscle function.

**Methods:** Thirty diabetic patients were randomly assigned to inspiratory muscle training (IMT) or placebo-IMT (P-IMT). Inspiratory muscle training was performed at 30% of the maximal inspiratory pressure (MIP) and placebo training was performed at 2% of the MIP, once daily, seven days/week/12 weeks. Data were analyzed *per protocol* and intention-to-treat. Fasting blood glucose and glycated hemoglobin (HbA1c) were primary outcomes, and respiratory muscle strength and lung function were secondary outcomes. The study was approved by the Ethical Commission (61986916900005327). The clinicalTrials.gov identifier is NCT 03191435, study release date:10/07/2017.

**Results:** Thirty patients were included, 73.3% women, 59.6 ± 10.7 years, HbA1c 8.7 ± 0.9%, glycemia 181.8 ± 57.8 mg/dL, 15 in P-IMT and 15 in IMT. Considering the subjects who performed more than 80% of the exercise sessions, the attendance rate was: IMT, 60%, and P-IMT, 80%. After 12 weeks HbA1c was 8.2% ± 0.3% and 8.7% ± 0.3%for IMT and P-IMT, respectively (p = 0.8). Fasting glycemia decreased in both groups without difference between them at 12 weeks. It was lower, however, in IMT at 8 weeks (170.0 mg/dL ± 11.4 mg/dL and 184.4 mg/dL ± 15.0 mg/dL for IMT and P-IMT, respectively; p < 0.05). Respiratory endurance time improved in IMT group (baseline: 325.9 s ± 51.1 s and 305.0 s ± 37.8 s; after 12 weeks: 441.1 s ± 61.7 s and 250.7 s ± 39.0 s for IMT and P-IMT, respectively; p < 0.05) while respiratory muscle strength and pulmonary function did not change. During the study adverse events reported were hyperglycemia, fatigue, dyspnea, dizziness, nausea, headache, and hypoglycemia, which were similar between groups.

**Conclusions:** Considering that glucose control, respiratory muscle strength and lung function do not improve in diabetic patients submitted to inspiratory muscle training at 30% of MIP, it should not be used as an alternative to other types of exercise in diabetes. Higher exercise intensities or longer training periods could determine better results.

**Support:** Fundo de Apoio à Pesquisa do Hospital de Clínicas de Porto Alegre (FIPE), grant 160615, CAPES, grant465518/2014-1.

### P167 Instrument for care for pregnant women with diabetes mellitus based on the sunrise model

#### Thaís Braga Meira^1^, Leila Rangel da Silva^2^, Renata Braga Meira^3^

##### ^1^Estadual de Diabetes e Endocrinologia Luiz Capriglione, Rio de Janeiro, Brazil; ^2^Universidade Federal do Estado do Rio de Janeiro, Rio de Janeiro, Brazil; ^3^Instituto Estadual de Hematologia Arthur da Siqueira Cavalcanti, Rio de Janeiro

*Diabetology & Metabolic Syndrome* 2019, **11(Suppl 1):**P167

**Introduction:** This study derives from a professional concern through a study conducted between April and October 2016 with pregnant women diagnosed with gestational diabetes mellitus, a study titled, relationships between culture and self-care of women diagnosed with gestational diabetes mellitus, the data showed that factors of pregnant women, such as inadequate eating habits, lack of physical activity, associated with insufficient or inadequate transmission of information by professionals, result in the lack of knowledge about their disease and self-care deficiency.

**Objectives:** To identify how cultural factors interfere with self-care of pregnant women with diabetes, to elaborate an instrument for nursing consultation based on the Leninger sunrise model, which focuses on self-care of pregnant women with diabetes.

**Methods:** This is an eminently qualitative study, whose research was intervention. Sixteen pregnant women were interviewed during the period from May to October 2017. The scenario was a secondary level Health Unit in the city of Rio de Janeiro. Data were collected through a questionnaire based on the cultural and social questionnaire of Leininger. In order to identify the social and cultural dimensions, we used as a theoretical basis the first level Sunrise Model by Madeleine Leininger and with regard to understanding the discourses of our subjects, we used the proposition of Bardin Content Analysis.

**Results:** Two major categories were coded: 1) Implications for newborn health arising from adherence to the treatment of pregnant women with diabetes mellitus 2) Culture x Self-care: diet as an essential element for the health and control of diabetes mellitus in pregnant women. The results demonstrate that nurses need to raise multiple factors that influence the expressions of cultural care and their meanings. The method used allowed approximations of the experiences of women with diabetes and the influence of the support network on self-care of these pregnant women.

**Conclusion:** The relationship in the context of a chronic disease between the nurse, the individual and their family has a fertile ground for the development of transformative educational actions with application of the instrument built from the results.

**Keywords:** Diabetes; Gestation; Diabetes Education.

### P168 Insulin action in vivo in double type 1 diabetes is associated with cardiovascular risk factors

#### Luana Aparecida de Lima Ramaldes, Sarah Simaan dos Santos, Patricia Medici Dualib, João Roberto de Sá, Sérgio Atala Dib

##### Disciplina de Endocrinologia da Escola Paulista de Medicina - Universidade Federal de São Paulo, São Paulo, Brazil

*Diabetology & Metabolic Syndrome* 2019, **11(Suppl 1):**P168

**Introduction:** Double Type 1 Diabetes (DT1D) is associated with overweight and insulin resistance (IR) in Type 1 Diabetes Mellitus (T1DM). Despite the increased prevalence in recent years this clinical entity is not so well characterized and its treatment has been similar to classic T1DM.

**Objective:** To evaluate the relationship between insulin sensitivity in vivo and cardiovascular risk factors in individuals with double Type 1 diabetes mellitus.

**Methods:** Eleven patients were recruited with characteristics compatible with DT1D (T1DM and at least two of the following criteria: Systemic arterial hypertension (defined by NCEP), overweight or obesity (WHO), dyslipidemia (NCEP) and clinical signs. These patients were submitted to insulin tolerance testing with calculation of plasma glucose disappearance rate (K_ITT_), evaluation of clinical and epidemiological characteristics and metabolic parameters associated with cardiovascular risk (glycated hemoglobin, lipids, uric acid, ferritin, ultrasensitive PCR). Statistical analysis were performed using the software SPSS20 and the Pearson and Spearman correlation coefficients were used with a p value < 0.05. The study was submitted and approved by the ethics committee.

**Results:** The patients were distributed in 5 ♂ and 6 ♀, age 22.3 ± 4.9 y (average + SD), family history of Type 2 Diabetes Mellitus 54.5%, time from diagnostic 13.5 ± 3.5 yr, BMI: 29.0 ± 2.75 kg/m^2^, HbA1c 8.1 ± 0.6%, insulin dose (U/kg/day) 0.9 ± 0.21, K_ITT_ 2.4 ± 1.3%/min, systemic arterial hypertension 18.2%, dyslipidemia 27.3%. There is a positive correlation in K_ITT_ for the female sex (r = 0.751 p = 0.008) and a negative K_ITT_ correlation with triglycerides (r = − 0.683 p = 0.02), ferritin (r =  − 0.682 p = 0.02) and plasma uric acid (r = − 0.674 p = 0.02).

**Conclusions:** Patients with DT1D have more than 50% of T2DM family history and a negative correlation between the *in vivo* insulin action degree and cardiovascular risk factors. CAPES financial support.

### P169 Insulin adsorption on salin bags and infusion set with and without PVC

#### Rogério Silicani Ribeiro, Thalita Barreira Modena, Ana Paula S Artilheiro, Natalia Mazini Ribeiro, Magda Tiemi Yamamoto

##### Hospital Israelita Albert Einstein, São Paulo, Brazil

*Diabetology & Metabolic Syndrome* 2019, **11(Suppl 1):**P169

Intravenous insulin is the therapy of choice for critically ill patients with hyperglycemia. However, insulin adsorbs to plastic tubing, which decreases the concentration of an insulin solution infused. Few studies have evaluated the loss of adsorbed insulin, the stability of solutions in equipment of different materials (with and without PVC).

**Objective:** To study “in vitro” insulin adsorption in different infusion set with or without PVC following current recommendations.

**Methodology:** Regular insulin concentration was measured in samples after dilution in saline (1UI/ml) before and after flushing in different volumes (10, 20, 30, 40, 50 and 60 milliliters of solution). The solution bag was connected to the infusion pump and infused at a rate of 1 ml/h. Samples were collected at 0, 1, 2, 4, 6 and 24 h. The experiment was done in duplicates, with and without PVC. The dosing method was ultra high performance liquid chromatography (UHLPC).

**Results:** There was a progressive reduction in insulin concentration in the PVC and non-PVC bags after the washout volumes were discarded, with a 40% and 20% reduction in the initial concentration in the PVC and non-PVC bottles. Analyzed at the end of the equipment line, the insulin concentration before and after 1, 2, 4, 6 and 24 h reduces to 50, 49, 39, 22, 19 and 17% of the expected concentration in the non-PVC and 70, 71, 62, 56, 45 and 41% in the PVC equipment.

**Conclusion:** Insulin adsorption causes a reduction in insulin concentration in the vial and at the end of the infusion set, being more pronounced in non-PVC sets.

### P170 Insulin degludec has lower hypoglycaemia risk than insulin glargine u100 in older people with type 2 diabetes

#### Tatiana Martins Benvenuto Louro Berbara^1^, Simon R. Heller^2^, J. Hans DeVries^3^, Carol H. Wysham^4^, Charlotte T. Hansen^5^, Melissa V. Hansen^5^, Brian M. Frier^6^

##### ^1^Novo Nordisk Brasi lLtda, São Paulo, Brazil; ^2^University of Sheffield, Sheffield, UK; ^3^University of Amsterdam, Amsterdam, The Netherlands; ^4^University of Washington/Multicare Rockwood Clinic, Spokane, USA; ^5^Novo Nordisk A/S, Søborg, Denmark; ^6^University of Edinburgh, Edinburgh, UK

*Diabetology & Metabolic Syndrome* 2019, **11(Suppl 1):**P170

**Introduction and objective:** Vulnerability to hypoglycemia increases with age. To further assess the safety of insulin in older patients, the risk of hypoglycemia was investigated post-hoc in the SWITCH 2 treat-to-target, 64-week, crossover trial.

**Methods:** Patients with T2D and high risk of hypoglycemia were randomized, double-blind, to either degludec or insulin glargine U100 ± OADs. The primary endpoint was the number of positively adjudicated severe (external assistance) or symptomatic hypoglycemic events (plasma glucose < 56 mg/dL) during the two 16-week maintenance periods.

**Results:** For patients ≤ 65 (n = 450) and > 65 (n = 270) years, baseline median [range] diabetes duration was 12.0 [1–40] vs. 15 [1–54] years, mean A1C was 7.7 vs. 7.4%, and mean eGFR was 87.0 vs. 63.7 mL/min/1.73m^2^. No significant differences in A1C reduction (degludec vs. glargine U100) were seen for patients ≤ 65 and > 65 years. During the maintenance period, degludec had a lower risk of hypoglycemia (overall/nocturnal symptomatic) vs. glargine U100 in patients ≤ 65 (31/43%) and > 65 years (30/41%). The number of severe hypoglycemia episodes was not significantly lower. The adverse event rate was 3.2 and 3.3 events/patient-year for ≤ 65 years and 3.5 and 4.1 events/patient-year for > 65 years, for degludec and glargine U100, respectively.

**Conclusions:** Degludec was safe and effective, and the frequency of hypoglycemia was lower than glargine U100 in patients ≤ 65 and > 65 years with T2D.

### P171 Insulin degludec/liraglutide (IDEGLIRA) is efficacious and safe in patients with type 2 diabetes (T2D) with normal, mild or moderate renal impairment: analyses from phase 3 trials

#### Emanuela Mello Ribeiro Cavalari^1^, Vanita R Aroda^2^, Bruce W Bode^3^, Jaime A Davidson^4^, Helena W Rodbard^5^, Andreas Andersen^6^, Anne Kaas^6^, Tina Vilsbøll^7^

##### ^1^Novo Nordisk, São Paulo, Brazil; ^2^MedStar Health Research Institute, Hyattsville, USA; ^3^Emory University School of Medicine, Atlanta, USA; ^4^The University of Texas Southwestern, Medical Center, Dallas, USA; ^5^Endocrine and Metabolic Consultants, Rockville, USA; ^6^Novo Nordisk A/S, Søborg, Denmark; ^7^Steno Diabetes Center Copenhagen, University of Copenhagen, Copenhagen, Denmark

*Diabetology & Metabolic Syndrome* 2019, **11(Suppl 1):**P171

**Introduction:** The DUAL clinical trial program investigated the efficacy and safety of IDegLira in various type 2 diabetes (T2D) populations.

**Objective:** The aim of this post hoc analysis was to confirm the efficacy and safety of IDegLira versus comparators in patients grouped according to their baseline renal function, using data from the DUAL I–V trials.

**Methods:** All trials were 26 weeks in duration; for DUAL I there was also a 26-week extension. Endpoints were compared for patients stratified according to their baseline renal function using the following groups: Normal, mild and moderate renal function (estimated glomerular filtration rates (eGFR) of ≥ 90, ≥ 60– < 90 and of ≥ 30– < 60 mL/min/1.73 m^2^, respectively).

**Results:** Across trials, the number of patients included with normal, mild renal impairment and moderate renal impairment were 944, 820 and 116 with IDegLira; 474, 358, and 58 with basal insulin; 294, 226 and 37 with GLP-1RA; and 60, 73 and 13 with placebo, respectively. Across trials, HbA1c reductions from baseline to end of trial were significantly greater with IDegLira versus comparators in all baseline renal function groups. Tests for treatment by subgroup interaction showed that there were no differences in the treatment effect across the renal groups in all trials expect for DUAL V, where there was a greater treatment difference in the moderate renal impairment group. Across baseline renal function groups, the rates of confirmed hypoglycemia with IDegLira were lower compared with basal insulin comparators, but higher than those with GLP-1RA or placebo. At end-of-trial, eGFR was unchanged compared with baseline for all patients regardless of treatment. Overall, the rates of adverse events and serious adverse events were similar for patients with normal, mild and moderate renal impairment across treatments.

**Conclusions:** This post hoc analysis showed that IDegLira resulted in significantly greater HbA1c reductions than basal insulin, continued GLP-1RA and placebo comparators in patients with T2D with normal, mild or moderate renal impairment. Across baseline renal function groups, hypoglycemia rates with IDegLira were numerically lower compared with basal insulin, but numerically higher than those with GLP-1RA or placebo. Overall, the results are consistent with those observed in patients with normal renal function and no safety concerns with IDegLira were identified in patients with mild or moderate renal impairment.

### P172 Insulin pen: a study of the effectiveness of outdoor storage

#### Ana Beatriz de Andrade Soares de Oliveira, Sávio Dias de Paula Mello, Lorena Fortuna da Silva, Marcela dos Santos Ferreira, Júlio Cesar Santos da Silva, Cristiane Rosa Magalhães, Fernanda Zerbinato Bispo Velasco, Úrsula Pérsia Paulo dos Santos

##### Centro Federal de Educação Tecnológica Celso Suckow da Fonseca, Nova Iguaçu, Brazil

*Diabetology & Metabolic Syndrome* 2019, **11(Suppl 1):**P172

**Introduction and objectives:** Insulin is a medicine of great importance for a good glycemic control of patients with Diabetes Mellitus (DM), mainly type 1. Insulin is presented in bottles that can be used by means of devices such as insulin pump, needle and syringe assembly and applicator pen, with the last one being the focus of this study. The objective of this research was to evaluate the relative forms of insulin pens in relation to their temperature during leisure activities.

**Methods and results:** This is a descriptive and cross-sectional research aimed at measuring the temperature of insulin-applying pens. The tests occurred during the period from April to January, in an approximate time of 12 h a day, in outdoors places, such as beaches and museums. Tests with various types of packaging resulted in the following results: Pen in the case of the manufacturer itself reached 32.8° C, at maximum ambient temperature of 36.1° C; pen stored in school-bag pouch with two flexible ice reached 26.9° C at maximum ambient temperature of 36.1° C; pen stored in a plastic container reached 34.2° C at maximum ambient temperature of 35.2° C; pen in thermal bag with two flexible ice reached 24.9° C at maximum ambient temperature of 35.7° C.

**Conclusion:** With this study, it is realized that on days of constantly high temperature, it’s necessary to have a form of packaging allied to a refrigerated device, such as flexible recycled ice, since both the manufacturer’s case and other storage containers voids were not enough to keep the pen at a temperature below 30° C, recommended by the Brazilian Ministry of Health and the Brazilian Diabetes Society. Through this research based on regular everyday events, it is possible to create knowledge, both for users of insulin pens and for health professionals, that can make their health education practices more effective for the population.

### P173 Insulin resistance and lipid profile in overweight adolescents

#### Ana Beatriz Rodrigues Pinheiro, Eduarda Pontes dos Santos Araújo, Maria Eduarda Bezerra da Silva, Angélica Luiza Sales de Souza, Jéssica Bastos Pimentel, Adriana Augusto de Rezende, Ricardo Fernando Arrais, Severina Carla Vieira Cunha Lima

##### Universidade Federal do Rio Grande do Norte, Natal, Brazil

*Diabetology & Metabolic Syndrome* 2019, **11(Suppl 1):**P173

**Introduction:** In insulin resistance (IR) there is an impairment of insulin action in peripheral glucose uptake, which is associated with a higher risk of metabolic complications, especially in obese individuals. The study aimed to evaluate the prevalence of IR and its association with lipid profile in overweight or obese adolescents.

**Methodology:** Cross-sectional study, approved by the Research Ethics Committee, involving overweight or obese adolescents aged 10 to 19 years old attending a pediatric endocrinology outpatient clinic. Anthropometric data (weight and height) were collected and analyzed to calculate Body Mass Index and Biochemicals (HDL-c, triglycerides, insulin and fasting glucose). The HOMA-IR Index (Homeostatic Model Assessment) was calculated and the cutoff point ≥ 3.16 was adopted to diagnose insulin resistance. Data were evaluated by descriptive statistics and Chi square test, Student T test and Pearson correlation were applied. For all tests a 95% confidence level was used and p-value < 0.05 was considered statistically significant.

**Results:** 124 adolescents were evaluated, with a mean age of 11.4 (1.52) years, most of them were male (52.4%). Overall BMI averages of 26.5 kg/m^2^ (3.59), fasting blood glucose of 94.10 mg/dl (7.43) and fasting insulin of 12.76 (11.89) were found. The prevalence of IR was identified in 32.3% of individuals, being more evident in females (p = 0.001). It was observed that adolescents with IR had higher mean fasting blood glucose (p = 0.001), HDL-c (p = 0.005) and triglycerides (p = 0.001) when compared to those without IR. There was an association between IR and low HDL-c concentrations (PR 1.28, CI 1.02–1.61), high triglycerides (RP 1.39, CI 1.11–1.74) and high blood glucose (RP 1.88, CI 1.14–3.07). A positive correlation was observed between IR and triglyceride values (r = 0.185, p = 0.038) and fasting glucose (r = 0.328, p = 0.001), and a negative correlation with HDL-c values (r = − 0.287, p = 0.001).

**Conclusion:** There was a high frequency of IR among overweight adolescents, with higher prevalence in females. Adolescents with IR showed alterations in serum HDL-c, triglycerides and fasting glucose levels. Thisstudywasfinanced in partbythe Coordenação de Aperfeiçoamento de Pessoal de Nível Superior - Brasil (CAPES) - FinanceCode 001

### P174 Interrelationship of visceral obesity, gluteofemoral obesity and periodontal disease in women

#### Angelo César Fernandes Jacomossi, Fernando Shiba, Doris Hissako Matsushita

##### Universidade Estadual Paulista “Júlio de Mesquita Filho”, Araçatuba, Brazil

*Diabetology & Metabolic Syndrome* 2019, **11(Suppl 1):**P174

**Introduction:** Previous studies indicate an association between obesity and periodontal disease, but did not consider individuals with gluteofemoral obesity in their analysis.

**Objective:** To evaluate the severity of Periodontal Disease (PD) in obese women considering the distribution of body fat.

**Methods:** 39 women were evaluated, 15 with visceral obesity (VOb), 10 with gluteofemoral obesity (GFOb) and 14 with normal weight were considered as control group (C). All were submitted to periodontal evaluation and scored according to the Community Periodontal Index (CPI). Anthropometric measurements and bioimpedanciometry were performed in order to discriminate the regionalization of body fat. A collect of blood sample was made for fasting dosage of glycemia and insulinemia for determination of Homeostatic Model Assessment-insulin resistance (HOMA-IR). The three groups were compared in relation to the severity of PD. Correlation analyzes were performed between CPI and the following parameters: Body Mass Index (BMI), waist circumference (WC), waist/hip circumference ratio (C/Q), percentage of visceral fat in relation to body weight (%GV) and HOMA-IR. **Results:** The mean HOMA-IR and CPI score was significantly higher (p = 0.0045) in the ObV group compared to the C group, but no difference was founded between the GFOb e C regarding these parameters. There was no statistical difference in the GFOb and the VOb. Positive correlation was found between the CPI score (p = 0.0173), C/Q (p = 0.0004), and WC (p = 0.0082).

**Conclusion:** The present study confirms previous literature data associating obesity and PD, but shows that the latter is more severe in visceral obesity than in subcutaneous obesity, suggesting a harmful role of chronically inflamed abdominal fatty tissue in the oral health of obese individuals.

### P175 Investigation of cystic fibrosis-related diabetes in patients of Rio Grande do Norte

#### Ana Cristina Vieira de Melo^3^, Heglayne Pereira Vital da Silva^1^, Clariscy Samantha Andrade da Costa Mello^1^, Karla Simone Costa de Souza^1^, João Felipe Bezerra^2^, Jussara Melo de Cerqueira Maia^3^, Vera Lúcia Gil da Silva Lopes^4^, Adriana Augusto de Rezende^1^

##### ^1^Department of clinical and toxicological analyses, Federal University of Rio Grande do Norte, NatalBrazil; ^2^Natalense Faculty of Education and Culture (FANEC), Natal, Brazil; ^3^Department of Pediatrics, Federal University of Rio Grande do Norte, Natal, Brazil; ^4^Department of Medical Genetics, School of Medical Sciences, State University of Campinas, Campinas, Brazil

*Diabetology & Metabolic Syndrome* 2019, **11(Suppl 1):**P175

**Introduction:** Cystic fibrosis (CF) is an autosomal recessive disease caused by mutations in cystic fibrosis transmembrane conductance regulator (*CFTR*). In infants with CF, pancreatic disease presents as exocrine pancreatic insufficiency but endocrine pancreatic function is also disrupted causing progressive hyperglycemia and Cystic Fibrosis-related diabetes (CFRD). CFRD is the most common extrapulmonary co-morbidity associated with FC, affecting 19% of adolescents and 40–50% of adults with this condition. CFRD shares clinical features of type 1 and type 2 diabetes, and is associated with more severe FC prognoses. There is a correlation between mutations in the *CFTR* and phenotypes in the pancreas and gastrointestinal tract. Patients homozygous for the most common mutation of *CFTR*, F508del, appear to present more severe phenotypes; however, due to the great variety in clinical presentation it is not always possible to evidence this correlation.

**Objective:** To evaluate the influence of mutations in the *CTFR* on the glycemic profile of CF individuals.

**Methods:** Thirty-one patients with a diagnosis of CF with ages between 1 and 37 years were studied. The Shwachman-Kulczycki score was used for clinical evaluation of the patients studied. Two measurements of fasting glucose and glycated hemoglobin were performed to assess the glycemic profile. Analysis of variants of the *CFTR* gene was performed by the capture of exons (*Nextera Exome Capture)* followed by sequencing with Illumina HiSeq.

**Results:** The mean glucose obtained at diagnosis was 84.8 ± 14.6 mg/dL and after one year was 94.9 ± 32.6 mg/dL. For glycated hemoglobin the means for the two dosages were 5.1 ± 0.5% and 5.7 ± 0.7%, in respectively times. The mean time of disease was 9 years. Only three patients were diagnosed as pre-diabetics, with disease duration of approximately 1½ years, 3 and 8 years, respectively. All these three patients present recessive homozygous for the F508 deletion as *CFTR* genotype; however, they had a mild clinical score. No patient was diagnosed as diabetic.

**Conclusions:** In the studied population of CF patients, it was not possible to evidence significant alterations in the glycemic profile or to correlate with the *CFTR* genotypes, which may be justified by the short disease time presented by the patients. Nevertheless, further studies assessing long-term development of CFRD and associating with *CTFR* genotypes are necessary for early treatment and prevention of an important FC complication.

### P176 Is diabetic ketoacidosis at the onset of type 1 diabetes associated with a worst prognosis?

#### Barbara Karolyne Pereira Sousa, Fernanda Nascimento Faro, Potira Almeida Gurgel de Azevedo, Marina Cutrim Magalhães Vieira, Mariana Vilela Pereira, Cecilia Kauffman, Nilza Maria Scalissi, João Eduardo Nunes Salles

##### Santa Casa de Misericórdia de São Paulo, São Paulo, Brazil

*Diabetology & Metabolic Syndrome* 2019, **11(Suppl 1):**P176

**Introduction:** Diabetic Ketoacidosis (DKA) is the initial presentation of type 1 diabetes (T1D) in 30–46% of cases. Some studies have demonstrated that DKA at diagnosis is related with worse glycemic control.

**Objective:** To evaluate if DKA at diagnosis of T1D predicts worse glycemic control and diabetes complications.

**Methods:** This cross-sectional study included patients from a T1D service at a tertiary hospital. Data was collected from medical records. Inclusion criteria were previous diagnosis of T1D, age ≥ 18 years old and available data about initial presentation of T1D. Association between history of DKA at the diagnosis and the following variables was evaluated: age at diagnosis, gender, family history of T1D, type of therapy, micro and macrovascular complications, C-peptide and glycated hemoglobin levels (HbA1c). SPSS 13.0 was used for statistical analysis.

**Results:** A total of 146 patients were included. Mean age was 16 ± 0.9 years at diabetes diagnosis and 35 ± 1.1 at data collection, with 18 ± 0.8 years of disease duration. A total of 61% were female, 37% had family history, 57% microvascular complications (33% retinopathy, 33% nephropathy, 25% neuropathy) and 8% macrovascular complications (3% heart attack, 2% stroke, 3% peripheral artery disease). Mean HbA1c was 8.5% ± 0.1. Human insulin therapy was being used by 47%, while 35% were receiving insulin analogs and 18% continuous subcutaneous insulin infusion (CSII). Carbohydrate counting was used by 48%. The prevalence of KAD at diagnosis was 50.7%. Chi square test shown no association between KAD and gender, family history of T1D, type of therapy, diabetes complications, C-peptide or glycemic levels. In contrast, age at diagnosis was inversely correlated to KAD (p < 0.01).

**Conclusion:** In this population, KAD at the onset of T1D was not correlated to worse glycemic control or diabetes complications. According to our review, just three studies investigated this association. Although they found that KAD at diagnosis was correlated to worse glycemic control, in two of them, the use of CSII neutralized this effect. Being in a service specialized in T1D, where almost half of patients were at carbohydrate counting and using insulin analogs or CSII, was a bias. This was the first to evaluate association between KAD at diagnosis and diabetes complications. The inversely correlation between age and DKA was in accordance to previous data. More longitudinal studies are necessary to corroborate these findings.

### P177 Is serum urea an indicator of insulin resistance?

#### Marcel Catão Ferreira dos Santos^1^, Natália Nóbrega de Lima^1^, Breno Carvalho Cirne de Simas^1^, Débora Nobrega de Lima^2^, Isabella Ferezini Oliveira de Sá^2^, Felipe Roham de Vasconselo Lima^1^, Kaio Vinícius Rodrigues de Souza^1^, Josivan Gomes de lima^1^

##### ^1^Universidade Federal do Rio Grande do Norte(UFRN)/Hospital Universitário Onofre Lopes(HUOL), Natal, Brazil; ^2^Universidade Federal de Pernambuco(UFPE), Recife, Brazil

*Diabetology & Metabolic Syndrome* 2019, **11(Suppl 1):**P177

**Introduction:** Serum Urea (SU) is widely used as a marker of renal dysfunction. Whether there would be a role for SU as a marker of Insulin Resistance (IR) is unclear. Animal studies have suggested that metabolic effects of IR and Diabetes Mellitus (DM) inhibit endothelial Nitric Oxide Synthase (eNOS) activity. The presence of DM and obesity led to an increase in SU associated with decreased activity of eNOS in mice. The same association has not been established in humans.

**Objective:** To assess if there is an association between higher SU and higher levels of serum glycemia or DM.

**Methods:** We retrospectively obtained data from patients who performed blood tests at a clinical analysis laboratory. Information obtained included age, sex, fasting plasma glucose (FPG), serum creatinine, SU, and whether the patient had confirmed DM diagnosis. Estimated Glomerular Filtration Rate (eGFR) was calculated using the Chronic Kidney Disease Epidemiology Collaboration equation. Since kidney failure presents with elevated urea and may also cause insulin resistance, we only included patients without Chronic Kidney Disease (CKD) and SU within the normal range. Subjects were divided into quartiles, according to SU. We performed Mann–Whitney’s Test and Chi Square test, with the hypothesis that patients at the highest SU quartile would have higher FPG and higher prevalence of diabetes, respectively, as compared with patients at the lowest quartile.

**Results:** Our sample included 1158 patients, 76.9% female. Mean age was 45.0 ± 13.5 years, median creatinine was 0.70 mg/dL (0.4–1.1), median SU level was 28 mg/dL (11–40), median eGFR was 107.8 mL/min/1.73 m^2^ (90.1–158.2) and median FPG was 86 mg/Dl (48–409), 24.4% had DM. In the 25th and 75th percentile, SU levels were 22 mg/dL and 33 mg/dL, respectively. Median FPG was 84 mg/dL (65–377) in the lowest quartile and 90 mg/dL (48–409) in the highest quartile (p < 0.001). Those in the upper quartile were more likely to have DM than those in the lowest SU quartile (OR 2.58, 95%CI 1.74–3.82, p < 0.001).

**Conclusions:** Our findings suggest that higher SU is associated with higher serum glycemia and risk of DM. Maybe SU could be used as a biomarker associated with IR. This might be explained by inactivation of eNOS. Whether SU has incremental value if added to other biomarkers of IR is uncertain, and therefore, its clinical value in this context is yet to be established.

**Financial support:** None

### P178 Knockdown of NHLH2 in arcuate nucleus promotes body weight gain, anxiety-depressive behavior and inflammation in adipose tissue in mice

#### Rodrigo Scarpari Carraro, Guilherme Nogueira, Joseane Morari, Vanessa Bóbbo, Nathália Dragano, Licio Augusto Velloso

##### Laboratory of Cellular Signaling, State University of Campinas (UNICAMP), Campinas, Brazil

*Diabetology & Metabolic Syndrome* 2019, **11(Suppl 1):**P178

**Introduction:** In several experimental models of obesity, there is a progressive loss of the hypothalamic capacity to regulate whole body energy expenditure and caloric intake. One of hypothalamic neuronal groups produces proopiomelanocortin (POMC), which is cleaved by the convertase 1 (PC1/3) giving rise to melanocyte stimulating hormone α (α-MSH). It is also known that Nhlh2 controls the mRNA expression of the PC1/3 gene by heterodimerizing with Stat3 and increasing its transcriptional activity. Thus, Nhlh2 may play an important role in the dynamics of energy expenditure, which places it as a potential target for pharmacological approaches aimed at treating obesity.

**Objective:** Evaluate physiological changes with site-specific inhibition of Nhlh2 in the arcuate nucleus of the mouse hypothalamus by lentivirus.

**Methods:** Eight-week-old male Swiss mice weighing approximately 30 g (CEUA: 4072-1) (n = 15 per group) were fed either conventional or a high-fat diet and underwent stereotactic surgery for lentivirus inoculation in the hypothalamus to decrease Nhlh2 expression. After 6 weeks, the expression of Nhlh2, POMC, PC1/3, markers of inflammation, as well as, anthropometric parameters and behavior were evaluated. We used Western Blot and PCR techniques to measure the amount of protein and mRNA from samples and microscopy images to analyze in adipose tissue. For the comparison of means between two groups, we applied Student’s t-test for independent samples.

**Results:** Lentivirus injection was efficient to promote the inhibition of Nhlh2 expression in the hypothalamus and was confirmed by real-time PCR. Upon Nhlh2 inhibition, mice showed an increase in body weight and food intake, reduction in oxygen consumption, production of carbon dioxide, as well as the reduction of the transcription of Nhlh2, PC1/3 and IL-6 in the hypothalamus. Furthermore, the knockdown animals increase the anxio-depressive behavior. In addition, in the brown and white adipose tissue, there was an increased transcription of inflammatory markers.

**Conclusion:** This study provides further evidence for the involvement of hypothalamic Nhlh2 in the control of whole body energy homeostasis. Further studies may reveal potential mechanisms of regulation of Nhlh2 that can be useful to treat obesity.

**Financial support:** FAPESP.

### P179 Knowledge and attitude regarding the diabetes treatment of type 1 diabetes patients before and after diabetes education program

#### Ana Cristina Ravazzani De Almeida Faria^1^, Alexei Volaco^1^, Denise Beheregaray Kaplan^1^, Deise Regina Baptista^2^, Maria Augusta Karas Zella^3^, Luis Paulo Mascarenhas^2^, Willian Coradin Cordeiro^1^, Nayanne Hevelin Dos Santos De Oliveira^1^

##### ^1^PUC Parana, Curitiba, Brazil; ^2^UFPR, Curitiba, Brazil; ^3^faculdade Evangelica Mackenzie do Parana, Curitiba, Brazil

*Diabetology & Metabolic Syndrome* 2019, **11(Suppl 1):**P179

**Introduction:** Diabetes Mellitus (DM) is a chronic disease that causes micro and macrovascular complications. Its prevalence has increased and the success of treatment is a challenge for its complex and chronic character. Health education programs show great allies in the role of control and prevention of complications and can reduce costs in public health.

**Objective:** To evaluate the degree of attitude and knowledge regarding the diabetes treatment of patients with Type 1 diabetes mellitus (DM1) before and after a group diabetes education program.

**Methods:** Patients enrolled in the State Public Program of Insulin Analogues Supply were invited to participate in a three session diabetes education program with multidisciplinary team. Data from 75 patients who attended at least two of the three educational sessions were analyzed. All of them filled the DSMP questionnaire (*Diabetes Self*-*Management Profile*) before and after the education program. The questionnaire assesses the degree of attitude and knowledge regarding the diabetes treatment in 5 domains: hypoglycemia, exercise, insulin, diet and glucose test.

**Results:** There was a significant improvement in the mean scores in the DMSP questionnaire when comparing the pre and post education program (p < 0.000). When evaluating the different questionnaire domains: hypoglycemia, diet and glucose test showed significant differences. Almost 30% of the patients self-monitored glycemia 1 or 2 times a day and 36% used a fixed insulin dose in the prandial period. Para comparação das médias foi utilizado o Teste Paramétrico T de Student e o valor p significante utilizado foi < 0.05.

**Conclusions:** The Diabetes Education Program applied improved the scores of attitudes and knowledges of patients regarding the diabetes treatment measured by the DMSP questionnaire. Differences in different domains results helps mapping strategies to reinforce posterior educational advices at specific aspects of the treatment.

### P180 Leisure-time physical activity level of women with previous gestational diabetes: linda-brasil cohort study

#### Leony Morgana Galliano, Letícia Ribeiro Pavão da Silveira, Pâmella Goveia, Paula Bracco, Maria Inês Schmidit

##### Universidade Federal do Rio Grande do Sul, Porto Alegre, Brazil

*Diabetology & Metabolic Syndrome* 2019, **11(Suppl 1):**P180

**Introduction:** Regular physical activity (PA) is important to prevent Diabetes Mellitus 2 (T2DM). Gestational Diabetes Mellitus (GDM) presented a higher risk of developing T2DM, but little is known about the practice of PA in this context.

**Objective:** Describe the level of leisure-time PA and associated factors 1 year after delivery of women who had GDM.

**Methods:** The LINDA-Brasil cohort study recruited pregnant women with GDM attended by the Unified Health System in prenatal care services in Porto Alegre, Pelotas and Fortaleza, from October 2014 to September 2018. Questionnaires including socioeconomic, clinical and nutritional data were applied during recruitment. One year after delivery, the PA level was collected via telephone contact through the International Physical Activity Questionnaire (leisure-time), considering the classification of low, moderate of high level. Descriptive data were presented by relative and absolute frequencies or means and standard deviation. Poisson regression with robust variance was performed to estimate prevalence ratios (PR). To assess factors associated with PA level, the following variables were used: age, race/color, education, income, parity and body mass index 1 year after delivery.

**Results:** 1452 women with GDM were evaluated. The average age was 31.5 ± 6.3 years, 53.7% reported white race/color and 38.0% had an income ≤ 2 minimum wages. Within 1 year after delivery, the mean body mass index was 30.8 ± 6.45 kg/m^2^, 28.7% were overweight and 43.4% were obese. Regarding the leisure-time PA, 91.4% were classified as low level, 8.3% as a moderate and high level for 0.3%. Among the associated factors, the prevalence of low PA was 7% higher in women with lower education compared to those with completed high school (PR = 1.07; 95%CI 1.01 to 1.13), even after adjustment for confounders. The other factors evaluated (age, race/color, income, parity and body mass index 1 year after delivery) were not statistically significant.

**Conclusions:** Most women who had GDM had low level of leisure-time PA, especially those with less education. Considering the potential benefits of PA in prevention of T2DM, it is essential to provide postpartum follow-up guidance for women who have had GDM.

**Financial support:** Brazilian National Council of Technological and Scientific Development and Eli Lily Non-Communicable Diseases Partners.

### P181 Liraglutide and semaglutide improve cardiovascular and renal outcomes across most body mass index categories in type 2 diabetes

#### Ana Carolina de Campos Godi^1^, Deepak L. Bhatt^2^, Lawrence A. Leiter^3^, C. David Mazer^3^, Darren K. McGuire^4^, Richard Pratley^5^, Bernard Zinman^6^, John Buse^7^

##### ^1^Centro de Pesquisa Clínica em Diabetes - Faculdade de Medicina de Marília (FAMEMA), Marília, Brazil; ^2^Brigham and Women´s Hospital Heart and Vascular Center, & Harvard Medical School, Boston, USA; ^3^St Michael´s Hospital and University of Toronto, Toronto, Canada; ^4^Division of Cardiology, University of Texas Southwestern Medical Center, Dallas, USA; ^5^Florida Hospital Translational Research Institute for Metabolism and Diabetes, Orlando, USA; ^6^Lunenfeld - Tanenbaum Research Institute, Mt. Sinai Hospital, University of Toronto, Toronto, Canada; ^7^University of North Carolina School of Medicine, Chapel Hill, USA

*Diabetology & Metabolic Syndrome* 2019, **11(Suppl 1):**P181

**Objective:** Whether cardiorenal benefits of liraglutide and semaglutide are consistent across body mass index (BMI) categories is unknown. We performed post hoc analyses on LEADER and SUSTAIN 6 data to evaluate cardiorenal efficacy by BMI groups in patients with type 2 diabetes (T2D) and high cardiovascular (CV) risk.

**Methods:** LEADER and SUSTAIN 6 were randomized CV outcome trials of liraglutide and semaglutide vs placebo in 9340 and 3297 patients, respectively, with T2D and high CV risk. The primary outcome was a composite of CV death, non-fatal myocardial infarction, or non-fatal stroke (major adverse CV events, MACE), with secondary outcomes including nephropathy measures (new or persistent macroalbuminuria, serum creatinine doubling, end-stage kidney disease or death from kidney disease). We evaluated the effect of liraglutide and semaglutide on these cardiorenal outcomes, stratified by baseline BMI groups. Hazard ratios (HRs) for treatment vs placebo were calculated using a Cox proportional hazards model with treatment and eligibility risk group as factors, adjusted for baseline characteristics related to cardiorenal risk within BMI groups.

**Results:** In LEADER, 9%, 29%, 32%, and 30% of patients had a baseline BMI of < 25 kg/m^2^, ≥ 25– < 30 kg/m^2^, ≥ 30 – < 35 kg/m^2^ and ≥ 35 kg/m^2^, respectively; for SUSTAIN 6, this was 8%, 28%, 33% and 31%. Baseline characteristics were mostly balanced within BMI groups. Both liraglutide and semaglutide improved MACE and nephropathy outcomes across most BMI groups vs placebo. Additionally, more weight loss was observed with liraglutide (< 25 kg/m^2^: −0.85 kg; ≥ 25– < 30 kg/m^2^: −1.93 kg; ≥ 30– < 35 kg/m^2^: −2.06 kg; ≥ 35 kg/m^2^: −3.25 kg; p-interaction: < 0.001) and semaglutide (< 25 kg/m^2^: −3.34 kg; ≥ 25– < 30 kg/m^2^: −3.09 kg; ≥ 30– < 35 kg/m^2^: −3.65 kg; ≥ 35 kg/m^2^: −3.99 kg; p-interaction: 0.09) vs placebo.

**Conclusions:** In LEADER and SUSTAIN 6, liraglutide and semaglutide improved CV and renal outcomes with no apparent systematic differences across BMI groups.

### P182 Liraglutide as add-on to sglt2 inhibitors in patients with inadequately controlled type 2 diabetes

#### Ana Carolina De Campos Godi^1^, Rosangela Réa^2^, Lawrence Blondie^3^, Lidia Belousova^4^, Ofri Mosenzon^5^, Pedro A Garcia-Hernandez^6^, Sunil M Jain^7^, Jalal Nafach^8^

##### ^1^Centro de Pesquisa Clínica em Diabetes - Faculdade de Medicina de Marília (FAMEMA), Marília, Brazil; ^2^Endocrinology and Metabolism Service (SEMPR), Universidade Federal do Paraná, Curitiba, Brazil; ^3^Ochsner Medical Center, New Orleans, USA; ^4^Almazov National Medical Research Centre, Saint Petersburg, Russian Federation; ^5^Division of Internal Medicine, HadassahHebrewUniversity Hospital, Jerusalem, Israel; ^6^Endocrinology Service, University Hospital, Monterrey, Mexico; ^7^TOTALL Diabetes HormoneInstitute, Indore, MadhyaPredesh, India; ^8^Dubai Diabetes Center, Dubai Health Authority, Dubai, United Arab Emirates

*Diabetology & Metabolic Syndrome* 2019, **11(Suppl 1):**P182

**Introduction:** Glucagon-like peptide-1 receptor agonists (GLP-1RAs) and sodium-glucose cotransporter-2 inhibitors (SGLT2is) reduce glycated hemoglobin (HbA 1c), but randomized controlled trial data on their combined use are limited.

**Objetive:** The LIRA-ADD2SGLT2i trial compared the effect on glycemic control of liraglutide 1.8 mg/day vs placebo as add-on to SGLT2i ± metformin in patients with type 2 diabetes (T2D) during 26 weeks.

**Methods**: In this phase 3b trial, patients with T2D on a stable dose of SGLT2i ± metformin and with HbA 1c 7.0–9.5% were randomized 2:1 to add either liraglutide 1.8 mg/day or placebo. Exclusion criteria included a history of diabetic ketoacidosis (DKA) while being treated with SGLT2i and/or estimated glomerular filtration rate < 60 mL/min/1.73 m 2. The primary endpoint was change in HbA 1c from baseline at 26 weeks; also assessed after 26 weeks were change in body weight, the proportion of patients achieving HbA 1c < 7%, and safety.

**Results:** Overall, 412 patients were screened, 303 were randomized and 280 (92.4%) completed treatment (92.1% with liraglutide, 93.0% with placebo). Baseline characteristics were balanced between treatment groups: mean HbA 1c 8.0%, mean body weight 91.1 kg, mean duration of diabetes 9.9 years. At week 26, the mean change in HbA 1c from baseline with liraglutide was 0.98% (n = 203) vs 0.30% with placebo (n = 100) (estimated treatment difference [ETD]: 0.68%, 95% confidence interval [CI]: 0.89, 0.48; p < 0.001). The mean change in body weight from baseline with liraglutide was -2.81 kg vs 1.99 kg with placebo (ETD: 0.82 kg; 95% CI: -1.73, 0.09, p = 0.077). In the liraglutide group, 51.8% of patients achieved HbA 1c < 7.0% vs 23.2% in the placebo group (odds ratio: 5.1, 95% CI: 2.67, 9.87; p < 0.001). Nausea was the most frequent AE, occurring in 26.2% of the liraglutide group and 6.0% of the placebo group. Similar incidences of hypoglycemic episodes were reported in both groups (8.9% with liraglutide vs 8.0% with placebo); none were severe. The proportion of patients reporting serious AEs was low in both groups (liraglutide 2.5% vs placebo 1.0%). There were no reports of acute renal failure, DKA, diabetic foot ulcers or amputations with liraglutide in combination with SGLT-2i.

**Conclusions:** In patients with T2D, the addition of liraglutide to SGLT2i therapy (± metformin) provided superior glycemic control vs placebo, and had a safety profile consistent with the known safety profile of both drug classes.

### P183 LixiLan-G: a randomized trial assessing switching to iglarlixi vs. continuation of daily or weekly GLP-1RA in T2D inadequately controlled by a GLP-1RA and OAD(S)

#### Lawrence Blonde^1^, Julio Rosenstock^2^, Stefano Del Prato^3^, Robert R. Henry^4^, Naim Shehadeh^5^, Elisabeth Niemoeller^6^, Elisabeth Souhami^7^, Vanita R. Aroda^8,9^

##### ^1^Department of Endocrinology, Ochsner Medical Center, New Orleans, USA; ^2^Dallas Diabetes Research Center at Medical City, Dallas, USA; ^3^University of Pisa, School of Medicine, Pisa, Italy; ^4^UC San Diego and VA San Diego Healthcare System, Center for Metabolic Research, San Diego, USA; ^5^Rambam Health Care Campus, Haifa, Israel; ^6^Sanofi, Frankfurt, Germany; ^7^Sanofi, Paris, France; ^8^MedStar Health Research Institute, Hyattsville, MD, USA; ^9^Brigham and Women’s Hospital, Boston, MA, USA

*Diabetology & Metabolic Syndrome* 2019, **11(Suppl 1):**P183

**Introduction and objectives:** Fixed-ratio combinations (FRCs) of basal insulin plus a GLP-1 receptor agonist (RA) offer simple administration of complementary injectable therapies for T2D. Effects of switching to a titratable FRC of insulin glargine lixisenatide (iGlarLixi) in T2D patients (pts) receiving GLP-1 RAs have been unknown. LixiLan-G (NCT02787551) assessed the effects of switching to a titratable FRC of iGlarLixi in T2D pts receiving GLP-1 RAs.

**Methods and results:** The randomized, open-label, 26-week trial, compared switching to iGlarLixi vs. continuing a GLP-1 RA in T2D pts with HbA1c 7‒9%, receiving a maximum tolerated dose of a daily (60% of pts [liraglutide QD, exenatide BID]), or weekly (40% of pts [dulaglutide, exenatide extended-release, or albiglutide]) GLP-1 RA with metformin ± pioglitazone ± sodium-glucose cotransporter 2 inhibitor. Adherence to randomized treatment was reinforced and monitored throughout the study. In both arms, mean age was close to 60 years, the mean duration of T2D was close to 11 years, mean BMI was close to 33 kg/m^2^, and the mean duration of GLP-1 RA use was 1.9 year. iGlarLixi (n = 257) provided greater HbA1c reductions than GLP-1 RA (n = 257), from 7.8% at baseline to 6.7% and 7.4%, respectively (least-squares [LS] mean difference [primary endpoint] ‒0.6% ± 0.1%; p < 0.0001). HbA1c < 7% was reached by 61.9% (iGlarLixi) vs 25.7% (GLP-1 RA) of pts (p < 0.0001), and the composite of HbA1c < 7% without documented symptomatic hypoglycemia (< 54 mg/dL) was reached by 56.7% (iGlarLixi) vs 25.3% (GLP-1 RA) of pts. The LS mean differences in fasting plasma glucose (‒30.1 ± 3.0 mg/dL) and 2-hour postprandial plasma glucose after a standardized breakfast meal (‒51.3 ± 5.2 mg/dL) also favored iGlarLixi (P < 0.0001 in both cases). Documented symptomatic hypoglycemia (0.25 vs < 0.01 events/pt-year), nausea (8.6% vs 2.3%), and vomiting (3.1% vs 0.8%) were nominally more frequent with iGlarLixi vs GLP-1 RA.

**Conclusions:** Switching to iGlarLixi can further improve glucose control for T2D pts receiving the maximum tolerated GLP-1 RA dose with oral antidiabetic(s). The trial was sponsored by Sanofi.

### P184 Long-term clinical outcomes are worse in brazilian young-onset type 2 diabetes

#### Renata Midori Hirosawa, Sergio Atala Dib

##### Centro de Diabetes, Escola Paulista de Medicina - UNIFESP, São Paulo, Brazil

*Diabetology & Metabolic Syndrome* 2019, **11(Suppl 1):**P184

**Introdution:** When diabetes develops in young adults, the adverse societal effects could be greater because of the presence of a chronic disease throughout patients’ working life. In young adults a spectrum of hyperglycemic disorders needs to be considered. Autoimmune diabetes may manifest as the classic acute-onset type 1 diabetes (T1DM) phenotype or, alternatively, may initially produce a clinical form of slowly progressive insulin-dependent diabetes, the latent autoimmune diabetes in adults (LADA). Furthermore, mutations in the HNF1A gene present with progressive hyperglycemia and the patients are prone to develop micro- and macrovascular complications with the same frequency as type 2 diabetes (T2DM). Evidence is accumulating that young-onset type 2 diabetes (YT2DM) has a more aggressive disease phenotype, leading to premature development of complications. To our knowledge, difference in the prevalence of diabetes complications between YT2DM, T1DM, T2DM, LADA and HNF1A- MODY have not been examined.

**Objective:** To evaluate long-term clinical outcomes in a Brazilian YT2DM group compared to T1DM, T2DM, LADA and HNF1A-MODY.

**Methods:** We abstracted clinical data from electronic medical, laboratory records in a University Diabetes Center. Five different groups were defined 1) YT2DM (age between 18 and 40 years at the diagnosis); 2) T2DM (> 40 years at the diagnosis) 3) T1DM; 4) LADA and 5) HNF1A-MODY. All patients have been diagnosed for at least 5 years. HbA1c, lipid profile and chronic diabetes complications was evaluated in according with standard methodologies.

**Results:** 157 patients were included, the age at diagnosis of YT2DM (n = 61), T1DM (n = 26), T2DM (n = 39), LADA (n = 12) and HNF1A- MODY (n = 7) were, respectively: 30.2 ± 5.2 y.o; 25.9 ± 6.4; 50.5 ± 8.4; 51.8 ± 12.9; 25.6 ± 11.1. The duration of diabetes was 20.0 ± 10.8 yr. (YT2DM), 16.2 ± 8.0 (T1DM), 17.3 ± 6.4 (T2DM), 19.5 ± 6.7 (LADA) e 24.7 ± 8.6 (HNF1A-MODY), p = 0.09. The BMI (kg/m^2^) was 31.9 ± 5.7 (YT2DM)), 24.0 ± 3.0 (T1DM), 28.9 ± 4.8 (T2DM), 24.9 ± 2.9 (LADA) and 26.6 ± 5.5 (HNF1A- MODY), p < 0.001. The HbA1c (%) was: 9.2 ± 1.9 (YT2DM), 8.5 ± 1.4 (T1DM) e 8.8 ± 1.6 (T2DM), 8.3 ± 1.1 (LADA) e 7.7 ± 0.6 (HNF1A-MODY), p = 0.07. Dyslipidemia prevalence among the groups was 78% (YT2DM), 15.8% (T1DM), 84.6% (T2DM), 66.7%(LADA) and 71.4%(HNF1A), p < 0.0001. YT2DM compared to the others groups showed a higher percentage of nephropathy (p = 0.0002) and neuropathy (p = 0.0004), and superiority trend in retinopathy (p = 0.05) and cardiovascular events (p = 0.08).

**Conclusions:** YT2DM compared to other etiologies of diabetes of young, with a similar age of onset, duration of diabetes, glycemic control and lipid profile as T2DM, LADA and HNF1A was more aggressive in relation to chronic diabetes complications. Metabolic control and screening for micro and macroangiopathies should be precocious and intensified in this new condition of type 2 diabetes.

### P185 Low birth weight is associated with lower bone mineral content in adulthood? results of the longitudinal adult health studY (ELSA-BRASIL)

#### Nayranne Hivina Carvalho Tavares^1^, Carolina Gomes Coelho^2^, Sandhi Maria Barreto^2^, Luana Giatti^2^, Larissa Fortunato Araújo^1^

##### ^1^Universidade Federal do Ceará, Fortaleza, Brazil; ^2^Universidade Federal de Minas Gerais, Belo Horizonte, Brazil

*Diabetology & Metabolic Syndrome* 2019, **11(Suppl 1):**P185

**Introduction:** Low birth weight (LBW) seems to be related to lower accumulation of bone mineral content (BMC) during life and may predispose to greater susceptibility to chronic diseases.

**Objective:** To investigate the association of birth weight with bone mineral content among adults and elderly participants in the Longitudinal Adult Health Study (ELSA-Brasil) and whether the association is different between men and women.

**Methods:** This is a cross-sectional study with 10,499 participants from the second wave (2012–2014) of ELSA-Brasil. The outcome variable was the amount of BMC in kilograms determined by electrical bioimpedance. The explanatory variable of interest was the self-report low birth weight (No and Yes: < 2.5 kg). The mean differences and their confidence intervals of 95% were estimated using linear regression models. Sequential adjustments were made by age, race/skin color, educational attainment, leisure physical activity, alcohol consumption diabetes, antidiuretic use, menopausal status (for women), weight and height. All analyzes were stratified by sex and performed in Stata 13.0. The research was approved by the National Commission for Research Ethics (CONEP) through the approval letter of No. 976/2006, by the Research Ethics Committee of the Federal University of Minas Gerais (COEP/UFMG) and by the ethics committees of the other institutions involved in the study. Informed consent was obtained from all individual participants included in the study.

**Results:** Most were female (54.86%), with a mean age of 55 years (SD ± 8.74). After adjusting for socioeconomic characteristics, health-related behaviors and health conditions, those who reported LBW had a reduction in mean BMC compared with those who reported adequate birth weight (men: − 0.23, 95% CI: − 0.29, -0.17; women: − 0.14, CI 95%: − 0.18, − 0.10). However, when adjusting for current weight and height, there was reduction in the magnitudes of the associations (men: − 0.04, 95% CI: − 0.13; − 0.06; women: − 0.01, CI 95). %: − 0.11, − 0.05).

**Conclusion:** LBW seems to influence BMC in adults and the elderly, but part of this relationship is mediated by current height and weight. The magnitude of the association was stronger in men compared to women. Technical and financial support from the Ministry of Health, Department of Science, Technology and Strategic Inputs, Department of Science and Technology, Ministry of Science and Technology, National Council for the Development of Science and Technology.

### P186 Low-intensity resistance training promotes better control of capillary blood glucose in type 1 diabetes than high intensity: a case study

#### Denise Maria Martins Vancea^2^, Cláudio Barnabé dos Santos Cavalcanti^2^, Jonathan Nícolas dos Santos Ribeiro^3^, Iago Vilela Dantas^1^, Thiago Borges Madureira Sabino^1^, Stefhany Rodrigues da Silva^2^, Emmerson Cristyan Ferreira Dantas^2^, Ricardo Hosanã Dias da Silva^2^

##### ^1^Universidade Federal de Pernambuco - UFPE, Recife, Brazil; ^2^Universidade de Pernambuco – UPE; ^3^Instituto de Ciências Biológicas - UPE, Recife, Brazil

*Diabetology & Metabolic Syndrome* 2019, **11(Suppl 1):**P186

**Introduction:** There is evidence that resistance training (RT) is capable of increasing glucose uptake in skeletal muscle, with effects on decreasing fasting glucose and glycated hemoglobin (HbA1c) in individuals with type 1 diabetes mellitus (DM1). Some studies have shown that higher intensity RT may be more effective in controlling diabetes than lower intensity RT. However, few studies have evaluated different intensities of RT in the glycemic response of individuals with DM1.

**Objective:** To evaluate the glycemic response of a type 1 diabetic submitted to resistance training at different intensities.

**Methods:** It was characterized as a case study with a crossover design. The study included a woman, brown, type 1 diabetic, trained, 26 years old and 22 years of diagnosis of the disease. The experimental sessions were held in a Laboratory of a Northeastern Public University in the morning. The maximum repetition test (1RM) was performed to evaluate the exercise intensities. The day after the 1RM test, the volunteer performed the 40% of 1RM RT session and, after 08 days of the first intervention, the 80% of 1RM session. Capillary glycemia was collected before, during and after the experimental sessions. A descriptive analysis of the variables was performed by Delta Variation (∆) at baseline, during and after the intervention. The study was approved by the institutional ethics committee nº 36498714.7.0000.5207.

**Results:** The 40% of 1RM resistance training presented higher glycemic fall after session (143 mg/dL versus 100 mg/dL; ∆ = − 43 mg/dL). The 80% of 1RM resistance training session also promoted a post-session glycemic fall (267 mg/dL versus 240 mg/dL; ∆ = − 27 mg/dL), but by delta variation the intensity at 40% of 1RM bigger fall.

**Conclusion:** The lower intensity resistance training promoted better capillary blood glucose decrease in this type 1 diabetic.

Informed consent to publish had been obtained from the patient.

### P187 Maternal consumpsion of high fat diet in pre-gestational period and fetal outcome

#### Ana Paula Varela Sanches, Josilene Lopes de Oliveira, Bruna de Souza Lima, Maíra Schuchter Ferreira, Lais Angélica de Paula Simino, Josiane Érica Miyamoto, Márcio Alberto Torsoni, Letícia Martins Ignácio de Souza

##### Faculdade de Ciências Aplicadas da Unicamp, Limeira, Brazil

*Diabetology & Metabolic Syndrome* 2019, **11(Suppl 1):**P187

**Introduction:** Maternal obesity brings several complications for both mother and fetus, including gestational diabetes. There is a complex relationship between maternal body weight and fetal-placental development. Placental changes, maternal weight gain seem to be determinant for the child’s health. Thus, the aim of this study was to investigate the influence of maternal obesity and its metabolism on fetal outcome.

**Methodology:** Female Swiss mice (CEUA 4547-1) were submitted to a high fat diet for 4 weeks and then subjected to mating remaining on the same diet until day E19 when euthanasia and sample collection occurred. Body weight and food intake were analyzed before and during pregnancy. Western blotting and Oil Red staining were performed. Data were analyzed by t-test or Pearson correlation and expressed as mean and standard deviation (n = 19).

**Results:** The hyperlipidic group presented higher caloric intake (p < 0.04) compared to the control, and consequently higher pre-gestational weight gain (p < 0.0005). Correlation analysis showed that the higher mother’s weight, lower the fetus is (p = 0.006; r = 0.3). This may be associated with the increase in inflammatory cytokines that occur in obesity, suggesting that this condition would cause placental insufficiency, decreased nutrient transport and intrauterine growth retardation. Panoramic images with “Oil Red” staining show greater labeling in obese mothers, which could indicate greater lipid incorporation into the tissue and, at a 10x increase, there appears to be greater dispersion of the labyrinth zone, with few juxtaposed nuclear markings compared to control animals. To evaluate some molecular parameters, we verified the proteostase pathways in the tissue by quantifying the proteins p62 and proteasome and both showed a tendency (not significant yet) to decrease in the HF group, when compared to the control, which could already indicate a UPS process failure to the detriment of the autophage process. In RNAseq analysis we found that the proteasome gene increased in the hyperlipidic group compared to the control, suggesting the involvement of some post-transcriptional mechanism once the protein tended to decrease.

**Conclusion:** Excessive pre-gestational weight gain contributed to lower fetal weight do due ectopic accumulation of fat what may have affected the efficiency of this tissue.

**Financial support:** FAEPEX.

### P188 Maternal consumption of soy protein isolated by wistar rats during lactation program improves the antioxidating profile and hyperinsulnemia in adult progeny

#### Maíra Schuchter Ferreira^1^, Alice Helena de Souza Paulino^1^, Mayara Medeiros de Freitas Carvalho^1^, Poliana Guiomar de Almeida Brasiel^2^, Kacia Mateus^2^, Maria Lucia Pedrosa^1^, Aline Silva de Aguiar^2^, Sheila Cristina Potente Dutra Luquetti^2^

##### ^1^Universidade Federal de Ouro Preto, Ouro Preto, Brazil; ^2^Universidade Federal de Juiz de Fora, Juiz de Fora, Brazil

*Diabetology & Metabolic Syndrome* 2019, **11(Suppl 1):**P188

**Introduction:** Increased oxidative stress is associated with several changes in the body, including hyperglycemia. Studies show positive effects of soy protein isolate (SPI) on oxidative stress, acting in the prevention of chronic diseases, like diabetes and cardiovascular disease. However, because it contains phytoestrogens in its composition, the safety of its consumption has been questioned at critical stages of development (pregnancy and lactation).

**Objective:** To evaluate the effects of maternal consumption of SPI during lactation on the antioxidant and glycemic profile of progeny at weaning and in adulthood.

**Method:** The study was conducted after approval by Ethics Committee (018/2014). Lactating rats were housed in cages with 6 male puppies and divided into 2 groups: Casein Control (C): received casein feed (20% protein); Soy Protein Isolated (SPI): received PIS-based feed (20% protein). At weaning, pups/litter, randomly separated, started to receive commercial feed until 150 days. Were evaluated: food intake (FI), body weight (BW), blood glucose, insulinemia, liver activity of superoxide dismutase (SOD), catalase (CAT), glutathione peroxidase (GPx) and glutathione reductase (GR), content of oxidized glutathione (GSSG) and reduced (GSH), redox index (GSH/GSSG), lipid peroxidation (TBARS) and serum estradiol (mothers). Analyzes were performed by t-student (p < 0.05).

**Results:** SPI mothers presented sporadic increase AI, without changing MC, TBARS reduction, without changing the other parameters. The offspring did not change the AI and MC. At weaning, offspring SPI presented increase of GPx, GR, GSH/GSSG, indicating reduction of oxidative stress, no change in glycemic metabolism. In adulthood, offspring PIS showed reduction of TBARS, indicating reduction of oxidative stress, without altering antioxidant enzymes, however, presented hyperinsulinemia without blood glucose modification.

**Conclusion:** Maternal consumption of SPI in lactation programmed positive effects on oxidative stress, reducing in weaning and adulthood, corroborating studies that correlate its consumption in the elimination of free radicals, however, showed hyperinsulinemia in adulthood. Thus, in relation to the antioxidant profile it has positive effects on adult progeny, however, in relation to glycemic metabolism may present non-beneficial alterations, therefore, the consumption of SPI in critical phases of development should be cautious. Financial Support: CAPES, CNPq, PROPP/UFOP.

### P189 Maternal–fetal outcomes of pregnant women with t1 dm using continuous subcutaneous insulin infusion or multiple daily injections during pregnancy

#### Juliana Ogassavara^1^, Sergio Atala Dib^1^, Rosiane Mattar^2^, Patricia Medici Dualib^1^, Bianca De Almeida Pititto^3^

##### ^1^Disciplina de Endocrinologia, Escola Paulista de Medicina, UNIFESP, São Paulo, Brazil; ^2^Disciplina de Obstetrícia, Escola Paulista de Medicina, UNIFESP, São Paulo, Brazil; ^3^Departamento de Medicina Preventiva, Escola Paulista de Medicina, UNIFESP, São Paulo, Brazil

*Diabetology & Metabolic Syndrome* 2019, **11(Suppl 1):**P189

**Introduction:** As the diagnosis of type 1 diabetes mellitus (T1DM) occurs at an earlier age, women of reproductive age are affected. It is known that pregnant women with pre-existing diabetes have an increased risk of maternal–fetal complications. Regarding treatment of T1DM, continuous subcutaneous insulin infusion (CSII) has several advantages compared to multiple daily injections (MDI), but data about the best option during pregnancy is limited.

**Objectives:** This study aimed to compare the maternal–fetal outcomes of pregnant women with T1DM followed in a referral service using CSII or MDI during pregnancy.

**Methods:** The study included 172 pregnant women with T1DM in follow-up at a DM and Gestation Service, from 2008 to April/2019. Data were collected from the participants’ medical records. Continuous variables were represented as mean (standard deviation) and categorical variables as percentages (n). Variables of interest (Student’s t-test or Chi Square test) were compared between groups: CSII *versus* MDI, p < 0.05.

**Results:** Among the 172 pregnant women evaluated, 20% (35) used CSII and 80% (137) MDI, with a mean age of 25.5 (5.9) years. Mean levels of lipid profile variables and pre-gestational BMI were similar in the 2 groups, while hypothyroidism was more prevalent in CSII users [31.4% (11) vs. 12.8% (17), p = 0.008] than MDI users. There was an improvement in HbA1c throughout gestation in the 2 groups, but there was no difference in HbA1c values at the first trimester of pregnancy [8.7 (1.8) vs. 8.7 (1.6)%, p = 0.92] and at the 3rd trimester [7.2 (1.2) vs. 7.0 (0.8)%, p = 0.57] and in frequency of cesarean delivery [90 (27) vs. 73 (73)%, p = 0.08] comparing CSII vs. MDI respectively. There was no statistical difference in the prevalence of preeclampsia and neonatal complications (neonatal death, hyperbilirubinemia, intensive care unit admission, hypoglycemia and malformation). There was also no difference in mean of birth weight and of gestational age at birth [34 (3.8) vs. 36 (2.5) weeks, p = 0.07] comparing CSII vs. MDI groups.

**Conclusion:** There was no difference in maternal–fetal outcomes between the pregnant women with T1DM who used CSII *versus* MDI in this referral service, considering the profile of these women at the beginning of pregnancy. Previous glycemic control and reason for the use of CSII should be addressed in future studies to improve the knowledge about the effect of this therapy during pregnancy.

**Financial support:** CAPES.

### P190 Menopause per se is associated with calcium deposition in coronary arteries: results from the ELSA-Brasil

#### Marilia Fonseca^1^, Bianca De Almeida-Pititto^2^, Isabela M Bensenor^3^, Paulo A Lotufo^3^, Sandra R G Ferreira^1^

##### ^1^Department of Epidemiology, School of Public Health, University of São Paulo, São Paulo, Brazil; ^2^Department of Preventive Medicine, Federal University of São Paulo, São Paulo, Brazil; ^3^Internal Medicine Department, University of São Paulo, São Paulo, Brazil

*Diabetology & Metabolic Syndrome* 2019, **11(Suppl 1):**P190

Menopause *per se* is associated with calcium deposition in coronary arteries: results from the ELSA-Brasil.

**Introduction:** Coronary artery disease is a major cause of mortality worldwide. CT-determined calcium in the coronary arteries (CAC) has been shown to predict cardiovascular events. Menopause deteriorates cardiometabolic risk profile but its independent association with structural arterial changes was less investigated. We tested independent association of menopause with presence of calcium in the coronary arteries in women from the ELSA-Brasil.

**Methods:** This is a cross-sectional analysis of baseline data of women from the Sao Paulo centre of ELSA-Brasil. Exclusion criteria were menopause < 40 yrs, non-natural cause of menopause and hormone replacement therapy. They were stratified by menopausal status (pre- or post-menopause) and divided according to the presence of CT-determined CAC (CAC = zero or > zero for any calcium detectable). Comparisons were performed using Mann–Whitney or Chi squared test. Association of CAC > zero (dependent variable) with menopause (independent variable of major interest) was tested using multiple logistic regression, age, smoking, central obesity, hypertension, diabetes, dyslipidaemia, use of lipid-lowering drugs and cardiovascular disease.

**Results:** 2,169 women were included in this analysis. Those with detectable CAC were older (58.0 ± 8.0 vs 48.2 ± 7.7 years, p < 0.001) and had higher prevalence of obesity, hypertension, diabetes, dyslipidaemia and cardiovascular disease than women with CAC = zero. In crude analysis, a significant association of menopause with CAC > zero was found [OR 7.85 (95%CI 5.89–10.45)]. After adjustments for confounders in multiple logistic regression, the association was attenuated but menopause was still independently associated with CAC > zero [OR 1.55 (95%CI 1.04–2.33)].

**Conclusion:** Association of menopause with CAC > zero in a large sample raised the question if reduced hormonal levels could contribute to calcium deposition in coronary arteries independently of classical risk factors present in menopausal women.

**Keywords:** Menopause; Women; Coronary artery calcium; CAC; ELSA-Brasil.

### P191 Mesenchymal stem cells and low intensity ultrasound on the recovery of fracture of diabetic rats

#### Ingrid Marianne De Freitas Santos^1^, Cybelle Da Silva Nery^2^, Victor Franklyn De Oliveira^1^, Sara Emanuely Veríssimo Santos^1^, Tamires Do Nascimento^1^, Hélida Cristina Cirilo Da Silva^1^, Tânia Shi^1^, Silvia Regina Arruda De Moraes^3^

##### ^1^Departamento de Fisioterapia, Universidade Federal de Pernambuco - UFPE, Recife, Brazil; ^2^Programa de Pós-graduação em Biotecnologia/RENORBIO, Universidade Federal de Pernambuco - UFPE, Recife, Brazil; ^3^Departamento de Anatomia, Laboratório de Plasticidade Neuromuscular - UFPE, Recife, Brazil

*Diabetology & Metabolic Syndrome* 2019, **11(Suppl 1):**P191

**Introduction and objectives:** The diabetes mellitus affects the bone tissue, promoting alterations on osteogenesis processes, causing osteopenia and increasing fracture fragility. The bone marrow mesenchymal stem cells therapy (BM-MSCs) and the pulsed low-intensity ultrasound therapy (US) are an alternative to increase the healing process. The aim was to assess the effects of BM-MSCs associated with US on mineral bone density (MBD) and biomechanical parameters.

**Methods:** 77 *Wistar* rats were distributed in: Placebo Control Group (PCG, n = 11), Placebo Diabetic Group (PDG, n = 11), US Control Group (UCG, n = 12), US Diabetic Group (UDG, n = 12), Cell therapy Control Group (CtGCP, n = 7), Cell therapy Diabetic group (CtDGP, n = 8), Cell therapy and US Control Group (CtCGU, n = 8) and Cell therapy and US Diabetic Group (CtDGU, n = 8). At 60 days of age, the animals were induced to experimental diabetes through Estreptozotocin, and at 74 days of life, an open fracture of the right femur was performed. In the Cell therapy group, a suspension of cells was administrated into the medullary canal of the femur, after the fracture. From the first until the 24th post-cirurgical day, the treatment with the SONOPULSE ultrasound was initiated, 7 times per week, 20 min per day, pulsed modality, 1 MHz frequency, 100 Hz pulse repetition frequency, 2 ms pulse duration – 1:5 ratio, 20% duty cycle and 0.5 W/cm^2^ intensity. Then, the femurs were collected analysis of MBD and biomechanical properties.

**Results:** the BMD showed increased values on the animals from groups (CtCGP, CtDGP, CtCGU, CtDGU) when compared with their respective controls (PCG—p = 0.002 e PDG—p < 0.001). Concerning the biomechanical parameters, there was no difference in maximal force. However, it was observed an increase of cross sectional area (p < 0.001) and reduction on Maximum tension (p ≤ 0.03) and Elastic modulus (p = 0.041) on CtCGP and CtDGP in relation to their controls (PCG and PDG) and reduction of Maximum deformation of CtCGP, CtDGP, CtCGU and CtDGU in relation to their controls (PCG and PDG).

**Conclusion:** the combined use of BM-MSCs and US therapy was effective on promoting an increase of MBD of femurs supposing an early bone repair. On the other hand, in relation to the assessed biomechanical parameters, there was no differences between groups concerning the association of both therapies.

### P192 Metabolic and nutricional profile of diabetic chronic kidney patients in hemodialysis program

#### Aniette Renom Espineira^1^, Murilo Tudeia Sousa e Silva^2^, Arelly Bethania Fonseca Barbosa^2^, Isabella Aparecida Lopes Andrade^2^

##### ^1^Fundação Santa Casa de Misericórdia de Franca, Franca, Brazil; ^2^Faculdade de Medicina. Universidade de Franca (UNIFRAN), Franca, Brazil

*Diabetology & Metabolic Syndrome* 2019, **11(Suppl 1):**P192

**Background:** The prevalence of Chronic Kidney Disease has substantially increased and Diabetes Mellitus (DM) is overcoming Hypertension as underlying cause. Knowing the clinical and nutritional profile of diabetic subjects in hemodialysis treatment is essential for professionals that take care of these patients.

**Objectives:** To describe the clinical, metabolic and nutritional features of diabetic patients and to compare them with non-diabetics patients in a hemodialysis population.

**Methods:** A population based case–control study in 219 chronic kidney patients in hemodialysis program at the Dialysis Center of Santa Casa de Franca, São Paulo, Brazil. Data was collected from medical records. The studied variables were: albumin, ferritin, serum calcium, serum phosphorus, Parathyroid hormone (PTH), calcium x phosphorus product, Total Cholesterol (CT), Low Density Lipoprotein (LDL), High Density Lipoprotein (HDL), triglycerides, Body Mass Index (BMI), lean mass, fat mass, waist circumference (WC) and Vascular Calcification Kaupilla Score. Continue variables are expressed in mean and standard deviation, and categorical variables in frequency and percentage. Mann–Whitney test was used to compare means. Fisher and Chi Square tests were used to determine association between categorical variables. A type 1 error of 5% was assumed. Analyses were done using GraphPad Prism 7.0 program.

**Results:** DM was the main underlying disease (37.9%) and was a comorbidity in 21.9%. Diabetic group presented higher triglycerides levels (140.4 ± 86.5 mg/dL) and higher body fat mass (31.7 ± 11.5%) than non-diabetics (113.9 ± 67.0 mg/dL; 26.2 ± 11.9%, respectively). Serum phosphorus and albumin levels were lower in diabetics (4.7 ± 1.4 mg/dL; 3.9 ± 0.5 md/dL, respectively) than in non-diabetics (5.1 ± 1.4 mg/dL; 4.1 ± 0.3 mg/dL, respectively). There was an association between DM and suppressed plasmatic PTH level (א^2^ = 7.9; p = 0.01). There were no differences in Kaupilla score (3.3 ± 5.3 vs. 2.6 ± 4.7) and plasmatic ferritin level (433 ± 318 vs. 455 ± 366 mg/d) between diabetics and non-diabetics patients, respectively.

**Conclusions:** Diabetics patients in hemodialysis programs seem to present a worse nutritional status and a higher risk to have PTH level below target value. Although atherosclerosis is a known condition associated with DM, in the dialysis population vascular calcification in diabetics subjects is similar when compare with non-diabetics.

### P193 Metabolic profile evaluation in hepatitis c patients treated with direct-acting antivirals

#### Sérgio Elias Estefan Junior, Paula Fernanda Rodrigues de Mattos Cardoso, Cintia Marques dos Santos Silva, Carlos Eduardo Brandão Mello, Márcia Helena Soares Costa

##### Universidade Federal do Estado do Rio de Janeiro - UNIRIO, Rio de Janeiro, Brazil

*Diabetology & Metabolic Syndrome* 2019, **11(Suppl 1):**P193

**Introduction:** The implementation of direct antiviral agents (DAAs) in the treatment of hepatitis C has allowed a significant increase in cure rates (SVR) in patients with this disease. Recent studies have shown that a large proportion of these patients have concomitant hepatitis C-based fatty liver disease and changes in the various metabolic parameters and metabolic syndrome.

**Objective:** To assess the metabolic profile of patients with chronic HCV hepatitis before starting DAA therapy.

**Methods:** Cross-sectional study of thirty five genotype 1 HCV hepatitis patients with laboratory tests for metabolic variables (fasting glucose, glycated hemoglobin, insulin and lipid profile), liver markers (TGO, TGP, Gama GT) and calculation of liver fibrosis scores (FIB4; APRI; NAFLD SCORE) in addition to anthropometric data collection (BMI, waist-hip ratio). Transient Hepatic Elastography (THE) (fibroscan) was also performed for staging the degree of hepatic fibrosis with controlled attenuation parameter (CAP) to assess hepatic steatosis. To correlate liver fibrosis scores (FIB4; APRI; NAFLD SCORE) with THE, Pearson’s correlation coefficient was used.

**Results:** In the series of selected patients, the median age was 64 years, BMI 24.90 kg/m^2^ and waist-hip ratio was 0.83 for women and 0.92 for men; 19 of the individuals (54.2%) had dysglycemia, 7 diabetic (20.0%) and 12 pre-diabetic (32.2%). The median insulin resistance index (HOMA-IR) in 20 patients evaluated was 3.19; Regarding lipid profile, 51.4% had some pattern of dyslipidemia; of the individual evaluated by THE: 36.0% had hepatic steatosis (S1 = 20.0%; S2 = 12.0%; S3 = 4.0%) with a CAP median of 194 dB/m and 41.1% had some degree of liver fibrosis (F2 = 20.5%; F3 = 5.8%; F4 = 14.7%) with a median of 6.75 kPa. The scores had a correlation of 0.79 (FIB4); 0.66 (APRI); 0.56 (NAFLD SCORE) in relation to THE, with medians of 2.019 (FIB4); 0.576 (APRI); -0.284 (NAFLD SCORE).

**Conclusion:** The result of our study is in agreement with studies in different parts of the world that described an association between 13% and 33% in patients with chronic HCV and diabetes. Thus, it is evident the need for adequate follow-up for screening and control of fatty liver disease associated with metabolic causes in these patients after the sustained virologic response.

### P194 Metabolic syndrome and diabetes mellitus

#### Carla Simone Moreira de Freitas^1^, Aleida Nazareth Soares^2^

##### ^1^Fundação Cristiano Varella - Hospital do Câncer de Muriaé, Muriaé, Brazil; ^2^Instituto de Ensino e pesquisa da Santa Casa de BH, Belo Horizonte, Brazil

*Diabetology & Metabolic Syndrome* 2019, **11(Suppl 1):**P194

**Introduction:** Prostate cancer (PCa) is the second most common cancer among men in Brazil. Recently, several studies have hypothesized a relationship between PCa and metabolic syndrome (MS).

**Objectives:** To evaluate the metabolic syndrome in patients with prostate cancer and to correlate with the risk classification according to Gleason scores.

**Method:** Retrospective observational study conducted with 3885 men with prostate cancer treated from 2005 to 2018. Metabolic Syndrome is a set of diseases based on insulin resistance or diabetes mellitus, associated with hypertension and dyslipidemia. Comparison of Gleason and Metabolic syndrome by Chi square test. 5% significance level and software used SPSS 20.0

**Results:** Seven hundred and three patients (18.1%) had metabolic syndrome. Most patients (43.03%) had Gleason score <= 6 (low risk), 22.88% Gleason 7 (intermediate risk) and 34.08% score > = 8 (high risk). There was a significant association between Gleason score and metabolic syndrome (p < 0.001). Patients with Gleason > = 7 (intermediate and high risk) have a higher proportion of metabolic syndrome (49.7%) compared to those with low risk (7.9%).

**Conclusions:** This study evidenced the association between metabolic syndrome and higher Gleason values and, consequently, more aggressive diseases with worse prognosis.

### P195 Mindfulness: an intervention study in patients with type 1 diabetes mellitus

#### Jeane Lorena Lima Dias, Heloisy Andrea da Costa Brasil, Aline Leão Reis, Talita Nogueira Berino, Daniela Lopes Gomes

##### Universidade Federal do Pará, Belém, Brazil

*Diabetology & Metabolic Syndrome* 2019, **11(Suppl 1):**P195

**Introduction:** Mindfulness is defined as the ability to bring attention to the present moment, without judgment or criticism, with an openness and curiosity attitude.

**Objectives:** To identify if the mindfulness intervention contributes to changes in awareness of automatic behaviors in patients with type 1 diabetes.

**Methods:** Participants were 20 to 40 years old; residents of Belém; at least 1 year of diagnosis; agreed to sign a consent form. The subjects responded to the protocols: 1) Screening Protocol; 2) Mindful Attention Awareness Scale (MAAS): Applied before (week 1, W1) and after intervention (week 12, W12), was created by Brown e Ryan (2003) validated by Barros et al. (2015), that measures individual differences in the frequency of mental states and consciousness over time. It was used a Likert scale ranging from 1 (almost always) to 6 (almost never), all the answered values were added, the mindfulness score can range from 15 (minimum level) to 90 (maximum level). The groups were randomized into: control group (CG) and intervention group (IG), which went through a mindfulness training (mind stabilization, body scanning and chocolate meditation), then, they were instructed on the application of mindfulness in daily life, relating this to diabetes. Study approved by the Ethics Committee, number of approval 3.232.967.

**Results:** Of the 21 subjects (S), 5 were selected (CG: S1, S2, S3; IG: S4, S5). It was observed that IG [S4: 60 (W1), 65 (W12); S5: 55 (W1), 78 (W12)] had an increase in the score compared to CG [S1: 71 (W1), 64 (W12); S2: 55 (W1), 40 (W12); S3: 56 (W1), 52 (W12)], which had a decrease. In the CG, all members reduced the score about “walking without paying attention”, S1 showed a decrease in the scores for “distraction from activities”, “not acting consciously” and “forgetfulness”. About IG, the highest scores for all members were to “focus on the aim”, “talk and do something else” and “drive on autopilot”; S5 indicated that was eating her meals carefully, while S4 had an increased perception of eating after the interventions.

**Conclusion:** A punctual mindfulness intervention has contributed to increase the level of mindfulness to automatic behaviors. Financial Support: Coordination of Superior Level Staff Improvement.

### P196 Modulation of placentary transcriptome of high fat diet mice

#### Maíra Schuchter Ferreira, Josilene Lopes de Oliveira, Ana Paula Varela Sanches, Bruna de Souza Lima, Josiane Erica Miyamoto, Lais Angélica de Paula Simino, Adriana Souza Torsoni, Letícia Martins Ignácio de Souza

##### Universidade Estadual de Campinas, Limeira, Brazil

*Diabetology & Metabolic Syndrome* 2019, **11(Suppl 1):**P196

**Introduction:** Epigenetic changes in the genome of the fetus, induced by environmental factors during the critical periods of development, have a profound impact on gene expression and organism programming even in the absence of the stimulus that started this process. Chronic diseases, epidemic and self-perpetuation, like obesity, have been increasingly related to perinatal periods. Thus, the placenta emerges as an important link between the maternal and fetal environment during pregnancy, may represent an important influence on progeny metabolism.

**Objective:** To investigate, globally, placental transcriptome modulation of animals submitted to the high fat diet during the pre-gestational and gestational phases.

**Methods:** The study was conducted after approval by Ethics Committee (4547-1) Swiss female mice were divided into two groups: one received control diet (CD) and the other high fat diet (HF) for 4 weeks before mating and until the 19th day of gestation, when euthanasia was performed. According to weight gain during treatment HF animals were divided into prone (HFP) and resistant (HFR) to obesity. RNA was extracted from the placenta of the animals and transcriptome analysis was performed by sequencing RNA-seq: transcription alignment was performed by HISIT2 software, mapping by StringTie software using Merge, and assembly transcript expression was calculated using the kallisto software For statistical difference were considered transcripts that presented adjusted *p*-value < 0.05 and 2.5 times expression for upregulation or downregulation.

**Results:** Expression of 36265 transcripts in the placenta was identified. When comparing CD with HFR, 480 transcripts had upregulation and 436 transcripts had downregulation. In contrast, the results of the comparison with HFP showed 774 transcripts with upregulation and 541 transcripts showed downregulation. When comparing HFRxHFP, only 5 transcripts had upregulation and 2 transcripts had downregulation. The genes with the highest differential expression (50-fold increased or reduction) are mainly related to regulation of plasma cholesterol homeostasis, regulation of thyroid hormone inactivation during embryological development, modulation of transcription by chromatin NDA compression and regulation of activity of calcium channels.

**Conclusion:** Consumption of high fat diet during the pre-gestational and gestational period differently modulated placental transcriptome in obese or resistant mothers.

**Financial Support:** CAPES.

### P197 Molecular and clinical update on the Brazilian Monogenic Diabetes Study Group (BRASMOD)

#### Renata Peixoto Barbosa^1^, Luis E. Calliari^2^, Renata Pires Dotto^3^, Felipe Crispim^3^, Luciana Ferreira Franco^3^, Regina S. Moises^3^, André Fernandes Reis^3^, Fernando de Mello Almada Giuffrida^1^

##### ^1^Universidade Federal de São Paulo (UNIFESP), São Paulo, Brazil e Universidade do Estado da Bahia (UNEB), Salvador, Brazil; ^2^Faculdade de Medicina da Santa Casa de Misericórdia de São Paulo, São Paulo, Brazil; ^3^Universidade Federal de São Paulo (UNIFESP), São Paulo, Brazil

*Diabetology & Metabolic Syndrome* 2019, **11(Suppl 1):**P197

**Introduction:** Maturity-Onset Diabetes of the Young (MODY) is a heterogeneous group of monogenic forms of diabetes, commonly misdiagnosed as either type 1 or type 2 diabetes. Universal access to molecular diagnosis is still not feasible in most populations. Therefore, specific clinical data according to genetic cause can help devising cost-effective molecular diagnosis strategies.

**Objective:** to update the clinical and molecular profile of a Brazilian registry of MODY cases.

**Methods:** 337 patients (151 families) with clinical characteristics of MODY (proband age below 35 years old first degree relative with diabetes, negative islet auto-antibodies) were analyzed, 27 newly described in this report. Patients underwent Sanger sequencing of glucokinase (GCK) and/or hepatocyte nuclear factor 1 homeobox A (HNF1A) genes. Continuous variables were analyzed by ANOVA (Bonferroni correction) and expressed in mean + SD/median [interquartile range]. Categorical variables were tested with Fisher’s exact test and expressed in percentages. Individuals were divided in groups: GCK, HNF1A, and NM (no mutations in both genes).

**Results:** In total 36 families with GCK mutations, 19 with HNF1A and 103 with no mutations in both genes were identified. Seven families with GCK mutations have been newly identified, with 2 novel mutations. According to ACMG guidelines, Ser54Gly has uncertain significance and Ile130Val is likely pathogenic. The following clinical diferences were observed among probands in groups GCK × HNF1A × NM: age at diagnosis (10 ± 7 × 21 ± 7 × 23 ± 9 years old, p < 0.001); BMI at diagnosis (19 ± 2 × 22 ± 4 × 24 ± 4 kg/m^2^, p = 0.006 GCK × NM); HbA1c (6.3 ± 0.4 × 6.7 ± 1.1 × 8.0 ± 2.5%, p = 0.001 GCK x NM); and triglycerides (57[45–76] × 81[62–125] × 103[71–180] mg/dL, p < 0.001 GCK × NM); presence of hypertension (0 × 31 × 37%, p < 0.001); and insulin use (7 × 14 × 55%, p < 0.001).

**Conclusions:** Novel GCK mutations are common in the Brazilian population. Ongoing study of this genetic cause of hyperglycemia contributes to genotype/phenotype annotation in the Brazilian population, which can assist in devising cost-effective strategies for molecular screening in the future. HNF1A seems to be rare in our population. Further studies are needed to clarify the genetic etiology of other forms of monogenic diabetes in our population.

**Financial support:** FAPESP grant 2015/05123-9 and CNPq grant 454014/2014-7.

### P198 Moringa oleifera seed preparation decreases inflammation and improves glucose tolerance in obese mice

#### Marília kalinne da Silva Torres^1^, Yasmim Alline de Araújo Castro^2^, Ana Maria Rampeloti Almeida^2^, Amanda Mota Vieira^2^, Luana Cassandra Breitenbach Barroso^1^, Thiago Henrique Napoleão^1^, Bruno de Melo Carvalho^2^, Patrícia Maria Guedes Paiva^1^

##### ^1^Universidade Federal de Pernambuco, Recife, Brazil; ^2^Universidade de Pernambuco, Recife, Brazil

*Diabetology & Metabolic Syndrome* 2019, **11(Suppl 1):**P198

**Introduction:** Obesity is characterized by a state of low-grade chronic inflammation that leads to impaired insulin signaling. Plants are sources of compounds for treatment of obesity and insulin resistance. Seeds of *Moringa oleifera* contain bioactive proteins including the lectin WSMoL that shows immunomodulatory activity.

**Objective:** To evaluate the effect of *M. oleifera* seed preparations on the insulin tolerance, glucose tolerance and levels of inflammatory cytokines in diet-induced obese mice.

**Methods:** *M. oleifera* seed powder was extracted with distilled water and after centrifugation the supernatant corresponded to water extract (WE). The protein fraction (PF) was obtained after precipitation of WE proteins using ammonium sulfate (60% saturation). The lectin WSMoL from PF was isolated by chitin chromatography. Obesity and insulin resistance in Swiss mice (n = 6, per group) were induced with a high fat diet (55% energy from fat) for 8 weeks. Oral gavage treatment with *M. oleifera* preparations (WE at 400 mg/kg, PF at 40 mg/kg and WSMoL at 5 mg/kg) or vehicle was performed for 4 weeks. After the treatment period, the insulin (ITT) and glucose tolerance tests (GTT) were performed. Additionally, inflammatory cytokines were determined using the cytometric bead array system. The study was approved by the Animal Use Ethics Commission of the Federal University of Pernambuco (Process number 0042/2017).

**Results:** ITT was not altered by treatment with *M. oleifera* preparations. The obese mice treated with PF showed an increase in glucose tolerance compared to the untreated obese group. In the glycemic curve, only the group treated with PF showed a significant reduction in glycemic levels at 90 and 120 min. WE, PF and WSMoL-treated obese mice showed a significant decrease on the TNF-α and INF-© circulating levels when compared to the untreated obese group, reaching the levels presented by standard diet fed animals.

**Conclusions:** *Moringa oleifera* protein fraction improved glucose tolerance in obese mice and this effect may be due to reducing of proinflammatory cytokines.

**Financial support:** CAPES, CNPq and FACEPE.

### P199 My diary of treatment: construction and validation of an educational tool of the relationship between glycemic variation and insulin therapy in adults with diabetes mellitus

#### Luiz Henrique Diniz Miranda, Janice Sepúlveda Reis, Helem De Sena Ribeiro, Tatiane Géa Horta Murta, Suelen Rosa De Oliveira

##### Santa Casa Belo Horizonte Ensino e Pesquisa, Belo Horizonte, Brazil

*Diabetology & Metabolic Syndrome* 2019, **11(Suppl 1):**P199

**Introduction:** Treatment of Diabetes Mellitus (DM) needs adherence to specific aspects related to insulin therapy and carbohydrate counting. It is up to the interdisciplinary team to invest in educational strategies to support the self-care and autonomy of people with DM as educational tools to demonstrate the relationship between insulin therapy and glycemic variation.

**Objective:** To elaborate and validate an educational tool to promote the understanding of the relationship between insulin therapy and glycemic variation in adults with type 1 and type 2 diabetes mellitus.

**Methods:** This was a methodological study for the development of educational material according to the following steps: (i) elaboration of the tool; (ii) validation of content and point of view by the committee of judges; iii) pre-tests with the target population. The second step included 10 judges selected according to the inclusion criteria, and the third step included 12 people with heterogeneous characteristics and type 1 or 2 diabetes mellitus who were using insulin.

**Results:** Based on the literature review, discussions with specialists and the researcher´s empirical experience in the area of ​​DM, the first version of the educational tool called “My Diary of Treatment (MDT)” was formulated, containing: (i) virtual manual (booklet with detailed explanation and presentation of the educational tool); (ii) MDT educational tool (printed leaflet with scales and subscales for filling and interpretation) and (iii) self-adhesive labels sheets (to be highlighted and used to complete the scales). Up to the final version of the tool, it was necessary to develop three versions that during the study their items were changed as suggested by the specialists (tool development step), judges (content validation phase and point of view) and target population (pretest step). The Content Validity Index reached was 99.58% by the evaluation of the committee of judges and a high index of understanding of the MDT was reached in the pretests step.

**Conclusions:** The content and point of view of the educational tool called “My Diary of Treatment (MDT)” were validated and culturally appropriate for the adult population with type 1 and type 2 diabetes mellitus.

### P200 Nanoparticle-incorporated chloroquine as a possible anti-inflammatory therapy in type 1 diabetes mellitus

#### Renato Ferreira de Almeida Júnior^1^, OnyAraujo Galdino^1^, Karla Simone Costa de Souza^1^, Ricardo Fernando Arrais^2^, Arnóbio Antonio da Silva Júnior^3^, Paula Renata Lima Machado^1^, Kleber Juvenal Silva Farias^4^, Adriana Augusto de Rezende^1^

##### ^1^Department of Clinical and Toxicological Analyses, Federal University of Rio Grande do Norte, Natal, Brazil; ^2^Department of Pediatrics, Federal University of Rio Grande do Norte, Natal, Brazil; ^3^Department of Pharmacy, Federal University of Rio Grande do Norte, Natal, Brazil; ^4^Federal University of Campina Grande, Campina Grande, Brazil

*Diabetology & Metabolic Syndrome* 2019, **11(Suppl 1):**P200

**Introduction:** The type 1 *Diabetes mellitus* (T1DM) induced hyperglycemia causes an intense formation of advanced glycation end products and reactive oxygen species, triggering intracellular signaling cascades that culminate in the production of cytokines. These cytokines contribute to the development of micro and macrovascular complications. To date, the glycemic control performed by insulin treatment is the main way to prevent T1DM complications, however in some cases it is not effective. In this context, chloroquine (CQ) emerges as an adjunctive treatment to insulin in order to reduce the T1DM-related inflammation. In addition, the use of this drug incorporated in nanoparticles will increase its bioavailability, improving the pharmacological response and reducing the onset of adverse effects.

**Objective:** To evaluate the effect of CQ and nanoparticle-incorporated chloroquine (CQNPs) on *IL1B*, *IFNG* and *IL10* mRNA expressions in peripheral blood mononuclear cells (PBMCs) of patients with T1DM.

**Methods:** Twenty-five patients with T1DM aged 8–16 years, were recruited, from July 2017 to May 2018, in the pediatric endocrinology unit of a University Hospital. PBMCs were isolated from all individuals and then cells were plated. Cytotoxicity test was performed to assess which drug concentration (5, 10, 25, 50, 100 and 200 µM) would be used. After choosing the ideal CQ concentration, the PBMCs were tested for 24 h and 48 h, and submitted to 3 different conditions: without treatment, treatment with CQ, and treatment with CQNPs. In the end of each period, mRNA expressions of *IL1B*, *IFNG* and *IL10* were determined by relative quantification in real time PCR(qRT-PCR).

**Results:** Ten µM concentration was used in the study, once this concentration promoted 100% cell viability. *IL1B* (after treatment with CQ for 24 and 48 h; and treatment with CQNPs for 48 h)*, IFNG* (after treatment with CQ and CQNPs for 24 and 48 h) and *IL10* (after treatment with CQ and CQNPs for 24 h) mRNA expressionswere significantly reduced in the cells treated when compared to the untreated (p < 0.05).

**Conclusions:** This study was the first to demonstrate in vitro the effect of CQ and CQNPs in the reduction of *IL1B, IFNG* and *IL10* mRNA expression, demonstrating that CQ reduces inflammation in T1DM. These results open perspectives of the adjuvant therapeutic effect of CQ in T1DM to prevent the emergence of its chronic complications.

**Financial support:** CNPq.

### P201 Nephrin as an early biomarker of diabetic nephropathy

#### Iago De Souza Gomes^1^, Karla Simone Costa De Souza^1^, Heglayne Pereira Vital Da Silva^1^, Renato Ferreira De Almeida Junior^1^, Ony Araújo Galdino^1^, Bento João Da Graça Azevedo Abreu^2^, Marcela Abbott Galvão Ururahy^1^, Adriana Augusto De Rezende^1^

##### ^1^Department of clinical and toxicological analyses, Federal University of Rio Grande do Norte, Natal, Brazil; ^2^Department of morphology, Federal University of Rio Grande do Norte, Natal, Brazil

*Diabetology & Metabolic Syndrome* 2019, **11(Suppl 1):**P201

**Introduction:** Diabetic nephropathy (DN) is a complication of type 1 *Diabetes mellitus* (T1DM). Currently, the diagnosis of DN is made through the analysis of urinary Albumin/Creatinine Ratio (ACR), however this marker is not a specific finding of renal injury. Thus, it is necessary to identify new sensitive and specific biomarkers that isolated or associated with albuminuria, enable the early diagnosis of DN. Considering that an early and key event in the development of DN is the loss of podocytes, changes in the expression of podocyte-related proteins, such as nephrin, that is involved in the stabilization of the glomerular slit diaphragm, may become potential biomarker of glomerular damage in DN.

**Objectives:** To investigate the potential role of nephrin as an early, sensitive and specific biomarker of glomerular damage and DN in an experimental T1DM model.

**Methods:** Twenty-eight male Wistar rats (Ethics: 026/2014) were divided into 4 groups [control 30 days (CG30), control 60 days (CG60), diabetic 30 days (DG30) and diabetic 60 days (DG60)]. T1DM was induced by streptozotocin. In both study periods, body weight, biochemical parameters (glucose, creatinine, urea and ACR), histomorphometric parameters and nephrin protein expression (renal cortex by Western blot) were evaluated.

**Results:** Regarding the biochemical renal function parameters, reduction in urea concentrations (DG30: p = 0.003, DG60: p = 0.002) and increased ACR (DG30: p = 0.001 and DG60: p = 0.028) were observed in the diabetic groups when compared to controls in both periods. In addition, the animal of the DG60 presented with hyalinosis (p = 0.009) and glomerular fibrosis (p = 0.048) in the histomorphometric analysis. In the evaluation of nephrin, a reduced expression was observed in diabetic groups (DG30: p = 0.010 and DG60: p = 0.042) compared to control groups. In addition, negative correlations in the diabetic groups were found between serum creatinine and nephrin expression (r = -0.899 and p = 0.015), collagen (%) and Bowman space (r = -0.929 and p = 0.003) and blood glucose and nephrin expression (r = -0.893 and p = 0.007).

**Conclusion:** The hyperglycemia from T1DM is leading to hyalinoses and glomerular fibrosis, promoting the loss of nephrin of the glomerular slit diaphragm, which favor DN emergence and installation. Therefore, the results of this study attest the role of this protein as a marker of renal injury in DN.

**Financial support:** CNPQ.

### P202 New-onset diabetes mellitus after kidney transplantation

#### Matheus Pereira Salgado^1^, Patricia Moreira Gomes^1^, Gustavo Leite Jacovelli^2^, Maria Cristina Foss-Freitas^3^, Elen Almeida Romão^4^

##### ^1^Divisão de Endocrinologia e Metabologia - Hospital das Clínicas - Faculdade de Medicina de Ribeirão, Ribeirão Preto, Brazil; ^2^Faculdade de Medicina de Ribeirão Preto - Universidade de São Paulo, Ribeirão Preto, Brazil; ^3^Divisão de Endocrinologia e Metabologia - Departamento de Clínica Médica - Faculdade de Medicina, Ribeirão Preto, Brazil; ^4^Divisão de Nefrologia - Departamento de Clínica Médica - Faculdade de Medicina de Ribeirão Preto, Ribeirão Preto, Brazil

*Diabetology & Metabolic Syndrome* 2019, **11(Suppl 1):**P202

**Introduction:** New-onset diabetes after transplantation (NODAT) is a complication of kidney transplantation associated with increased risk of graft failure, cardiovascular disease, and mortality. Incidences of NODAT vary from 7% to 30% in the first year after transplant (this variation is due to the length of follow-up, immunosuppression regimen and diagnostic criteria). The risk factors include both general diabetes risks and those specific to transplant patients, such as immunosuppression.

**Objective:** To determine the incidence of NODAT and it’s associated factors in kidney transplant patients in a high complexity hospital in the state of São Paulo, Brazil.

**Methods:** This is a single center retrospective study that analyzed, based on medical records, the features of patients that developed NODAT after kidney transplantation performed between 2014 and 2018 at a high complexity hospital. NODAT was defined by the new onset or maintenance of hyperglycemia 30 days after transplantation (assuming that the patient was stable on an immunosuppressive regimen and in the absence of acute infection), according to American Diabetes Association (ADA, 2019). Patients with previous diagnose of Diabetes Mellitus were excluded.

**Results:** From the 682 patients who underwent kidney transplantation during this period, 41 (6%) died, 331 (48.5%) maintained normal glycemia, 140 (20.5%) had Type 2 Diabetes Mellitus, 23 (3.4%) had Type 1 Diabetes Mellitus, 90 (13.2%) had Pre Diabetes and 57 (8.4%) developed NODAT. The mean age of patients with NODAT was 56.6y 68.4% received from a deceased donor and 19.2% from a living donor. The main immunosuppressive agents used in this group were mycophenolate (87.7%), prednisone (84.2%), tacrolimus (63.2%), sirolimus (12.3%), and cyclosporine (1.8%). No patient were in use of azathioprine. In the group that did not develop NODAT, the mean age was 48.6y, 59.7% received from a deceased donor and 25.2% from a living donor. The immunosuppressive agents used in this group were mycophenolate (69.4%), prednisone (83%), tacrolimus (56.4%), sirolimus (15.5%), cyclosporine (7.3%), and azathioprine (11.5%).

**Conclusion:** In concordance with the incidence rate variety reported in other studies, NODAT was present in 8.4% of patients who underwent kidney transplantation. From these patients, 84.2% used prednisone and 63.2% used tacrolimus, which are known to be commonly associated with NODAT.

### P203 Night work is related to higher global and central adiposity in Brazil: National Health Survey, 2013

#### Brena Custodio Rodrigues^1^, Francisco Gustavo Silveira Correia^1^, Marcelo Jose Monteiro Ferreira^1^, Luana Giatti^2^, Lidyane Do Valle Camelo^2^, Larissa Fortunato Araujo^1^

##### ^1^Universidade Federal do Ceará, Fortaleza, Brazil; ^2^Universidade Federal de Minas Gerais, Belo Horizonte, Brazil

*Diabetology & Metabolic Syndrome* 2019, **11(Suppl 1):**P203

**Introduction:** Night work can disturb the “natural” circadian rhythm leading to disruptions in metabolic rates and consequently causing overall gain weight or even more harmful abdominal adiposity.

**Objective:** To investigate the association between night work frequency and markers of overall and central obesity and also verify if the association remains statically significant after removing the effects of potential mediators.

**Methods:** We carried out a cross-sectional analysis with over 35,500 current workers from the Brazilian National Health Survey (NHS). The exposure to current night work was composed of three categories: daytime work (reference category), ≤ 1 night per week, and ≥ 2 nights per week. Body mass index (normal weight, underweight, overweight, and obese) and waist circumference (adequate or elevated) were used as adiposity markers. Logistic and multinomial regression models were used. The NHS was approved by the National Research Ethics Committee (Protocol 328,159, of June 26, 2013). All interviewees were consulted, informed and accepted to participate in the research. As this work used it secondary data, there is no necessity of Ethics approval or informed consent.

**Results:** After removing influences of social demographics characteristics, work conditions, self-related health and also possible mediators as health-related behaviors, and compared to daytime workers, those who worked 2 or more nights a week had higher odds of overweight (odds ratio: 1.20, 95% CI: 1.04; 1.38), obesity (OR: 1.38, 95% CI: 1.17; 1.64) and elevated waist circumference (OR: 1.27, 95% CI: 1.10; 1.46).

**Conclusions:** Our results highlight the importance of actions that take into account employment and working conditions such as controlling the numbers of work nights per week or promoting better workplaces with healthy meals and the opportunity to do physical exercise at work. Funding: Ministry of Health of Brazil.

### P204 Nurse’s experience report on the diabetes educations process in a day-camp with children and adolescents with type 1 diabetes mellitus

#### Elessandra Da Silva Sicsu^1^, Alcemira Oliveira Bandeira^2^, Rebecca Ortiz La Banca^3^

##### ^1^SAMU.MANAUS, Manaus, Brazil; ^2^Uniao Noroeste Brasileira, Manaus, Brazil; ^3^joslin Diabetes Center, Boston, USA

*Diabetology & Metabolic Syndrome* 2019, **11(Suppl 1):**P204

**Introduction and objective:** Type 1 diabetes (T1D) is an autoimmune disease resulting in deficient insulin production, mainly affecting children and adolescents. Strategies such as camps help to improve lives of this population, and nurses play an essential role in this educational process. The objective of this study is to report the educational experience of nurses in a day-camp for children and adolescents with T1D.

**Methods:** Descriptive report of diabetes education strategies in a day-camp for youth with T1D. The camp took place on a Sunday (from 07:00am to 6:00 pm) in a private school in the west of Manaus, Amazonas. Nurses delivered the educational workshops after lunch about glycemic monitoring and insulin therapy including games, toys and puppets.

**Results:** Children and adolescents their guardians participated in the teachable moments proposed by nurses. In the glycemic monitoring workshop, they received information about the fingerstick locations, the importance of wheeling, hand hygiene, care with the glucometer and proper disposal of the waste. At the insulin therapy workshop, they were instructed on care for storage, preparation, hygiene and application of insulin, sites and application rotation, proper waste disposal and attitudes towards hypoglycemia and hyperglycemia. In the educational practice with games, toys and puppets, they were divided by age, and in small groups could understand important concepts about diabetes mellitus explained by nurses.

**Conclusion:** Day-camp has proved to be an important mode of education to promote self-care of children and adolescents with T1D and their guardians. Nurses delivered age appropriate strategies that become a major contributor to the diabetes education process.

### P205 Nutritional status and metabolic profile of children and adolescents with type 1 diabetes mellitus

#### Maria da Conceição Chaves de Lemos^1^, Lílian Caroline de Souza e Silva^1^, Dalila Fernandes Bezerra^1^, Skalyt Lee Barbosa e Silva^2^, Ávilla Monalisa Silva de Oliveira^2^, Lidiana de Souza Holanda^2^

##### ^1^Universidade Federal de Pernambuco, Recife, Brazil; ^2^Hospital das Clínicas - UFPE, Recife, Brazil

*Diabetology & Metabolic Syndrome* 2019, **11(Suppl 1):**P205

**Introduction:** The current nutritional profile characterized by the predominance of overweight and obesity has affected young individuals, including children and adolescents with type 1 Diabetes Mellitus (T1DM), thus contributing to the development of metabolic alterations and increasing the risk of cardiovascular diseases in this public.

**Objective:** To evaluate the nutritional status and metabolic profile of children and adolescents with T1DM.

**Methods:** The research was approved by the ethics committee under number 83335318.2.0000.5208 and the study had a cross-sectional design, whose sample consisted of T1DM patients from five to eighteen years old, of both sexes, followed in a university hospital in the Northeast of Brazil. Weight, height and waist circumference were measured, and the nutritional diagnosis was made according to the body mass index curves recommended by the World Health Organization. Lipid profile and glycated hemoglobin (HbA1c) were also evaluated. Data were computed and analyzed using SPSS version 13.0 and Epi-Info version 3.5.4 and the significance level of the results was maintained at 5%.

**Results:** A total of 102 patients were evaluated, of which 54.6% were female and 66.7% adolescents. 29.4% were overweight and waist circumference was high (above the 90th percentile) in 45.1%. The lipid profile showed hypertriglyceridemia in 38.2%, hypercholesterolemia in 44%, while high LDL, reduced HDL and VLDL in borderline values were evident in 24.5%, 25.5% and 10.8%, respectively. The average HbA1c was 9.4% (± 1.8), being high in 91.9% of the evaluated.

**Conclusions:** There was a significant frequency of overweight and central adiposity accumulation, revealed by the measurement of BMI and waist circumference. Regarding the metabolic profile, it was found the presence of dyslipidemia, whose occurrence may have been favored by the predominance of inadequate glycemic control in the sample. In this sense, since it is a pediatric public, preventive measures are necessary to avoid future cardiometabolic complications.

### P206 Nutritional status of zinc and magnesium in patients with congenital generalized lipodystrophy

#### Fábia Karine de Moura Lopes^1^, Camilla Oliveira Duarte de Araújo^2^, Natasha Vasconcelos Albuquerque^1^, Synara Cavalcante Lopes^1^, Ana Paula Dias Rangel Montenegro^1^, Virgínia Oliveira Fernandes^1^, Carla Soraya Costa Maia^3^, Renan Magalhães Montenegro Júnior^1^

##### ^1^Universidade Federal do Ceará (UFC), Fortaleza, Brazil; ^2^Universidade Federal de São Paulo (Unifesp), São Paulo, Brazil; ^3^Universidade Estadual do Ceará (UECE), Fortaleza, Brazil

*Diabetology & Metabolic Syndrome* 2019, **11(Suppl 1):**P206

**Introduction:** Congenital Generalized Lipodystrophy (CGL) is a rare syndrome characterized by the almost complete absence of adipose tissue, which leads to the development of metabolic complications such as severe insulin resistance (IR) and diabetes, being a human model for the study of IR and its consequences. Zinc plays an important role in the synthesis, storage and release of insulin, and has a beneficial effect on tissue sensitivity to this hormone. Magnesium is also involved in glucose metabolism and insulin homeostasis.

**Objective:** To evaluate the nutritional status of zinc and magnesium in patients with CGL.

**Methods:** This is quantitative descriptive study, approved by the Ethics Committee (CPEA: 2.627.842), performed at a hospital of reference in the care of these individuals in the state of Ceará, conducted from November 2014 to February 2015. Dietary intake of zinc and magnesium was assessed by three 24-h dietary recalls (24-HDR) collected on different days in seven patients with CGL. The results of the 24-HDR analysis were grouped as means and standard deviation and compared with the recommendations proposed by the Dietary Reference Intakes. The prevalence of micronutrient inadequacy was calculated by the Estimated Average Requirement (EAR) method as the cut-off point. Serum zinc and magnesium levels were evaluated. Reference values were 1.6 to 2.4 mg/dL for magnesium and for zinc the reference values were according to age. Blood was collected at night fasting for at least 10 h.

**Results:** Six patients were female, with a median age of 10 years, ranging from 5 to 31 years. The average daily intake of zinc and magnesium was 10.39 ± 3.67 mg and 293.27 ± 70.70 mg, respectively. It was observed that 85.71% and 71.43% of the patients had possibly adequate zinc and magnesium consumption, respectively. However, the male patient had possibly inadequate intake of both. The meanserum zinc level was 81.73 ± 7.48 μg/dL and magnesium was 1.47 ± 0.14 mg/dL. All patients had serum zinc levels within the recommended values, in contrast, 85.71% showed lower serum magnesium levels than recommended.

**Conclusions:** Despite the possibly adequate intake of zinc and magnesium, serum magnesium levels were lower than recommended. Therefore, additional studies evaluating the association of hypomagnesemia with IR worsening and the potential benefit of magnesium supplementation in CGL are important.

### P207 Observational study about the growth and distribution of bariatric surgeries performed in the hospitals of the Unified Health System in Brazil from 2009 to 2019

#### Márcio Ribeiro Melhor^1^, Eric Aguiar Wittlich^1^, Joice dos Santos de Jesus^1^, Adrienne Lilia Brandão de Jesus^2^, Catherine Castelo Branco de Oliveira^1^, Maria Luiza Nogueira de Barreiros Gavazza^1^, Mateus da Silva Santana^1^, Victor Costa Araújo^3^

##### ^1^Universidade Federal da Bahia (UFBA), Salvador, Brazil; ^2^Escola Bahiana de Medicina e Saúde Pública (EBMSP), Salvador, Brazil; ^3^Universidade do Estado da Bahia (UNEB), Salvador, Brazil

*Diabetology & Metabolic Syndrome* 2019, **11(Suppl 1):**P207

**Introduction:** Studies about obesity trends, mainly secondary to modern lifestyle, show that more than half of the country’s population is affected by this pathology. In a society where aesthetics has a place of relevance, bariatric surgery (BS) has become an alternative meansto escape from obesity and to seek better physical and health conditions. However, BS also promotes anatomic and biochemical changes in the gastrointestinal tract and brings risks both at the surgical process and in the post-surgery period, requiring several prerequisites and cautions that must be followed, what may also exclude some of the petitioners.

**Objective:** Toevaluate the numbers of BS performed by the Unified Health System (SUS) in Brazil between 2009 and 2019.

**Methods:** A descriptive observational study about progression of BS performed by the Brazilian SUS, based on database calculations administered by the national Ministry of Health (MS), DATASUS.

**Results:** The period analyzed revealed a 290% increase in thecountry’s hospitalizations, with a total of 75,577 patients admitted for bariatric surgery, mostly in the South region, with 57.8% of total and presenting the shortest average length of stay (3.2 days). Paraná stands out with more than half of theCountry’s total surgeries while theNorth is the region with less than 0.8% of the total procedures and the longest average stay (7.1 days). The highest mortality rate is in the Midwest region (0.80) and the lowest is in the Northeast (0.10) with no deaths recorded between 2009 and 2015.

**Conclusions:** Two main patterns may be identified, one related to the raise in the hospitalization rates due tothe procedure, and the other one linking surgery prevalence and hospitalization periods among country regions. As pointed, this health/aesthetic trend is growing and it’s important to have professionals well prepared to triage and properly support the ones who need the surgery and the ones who don’t. Likewise, this increase of almost three times more procedures in 6 years also puts pressure in the system structurally and economically, what perhaps may be aggravated due the recent changes in public policies, especially for less wealthy regions. The data revealed an inequality among regions, with the South projecting a better tendency when it comes to either volume of surgeries or length of stay when compared with the northern states, which is aligned with the current patterns in Brazil’s wealth and socialdevelopment distribution.

### P208 Offspring of diabetic mother: the neonatal profile

#### Cristina Figueiredo Sampaio Façanha^2^, Priscila Macedo Fernandes^1^, Priscila Alencar Barreira Higino^1^, Ana Luiza Oliveira Bastos^2^, Juliana Leitão Mesquita^2^, Sarah Costa Alencar^2^, Pedro Henrique de Santana Pereira^2^, Dayana de Alencar Mesquita Lima^1^

##### ^1^Centro Integrado de Diabetes e Hipertensão, Fortaleza, Brazil; ^2^Centro Universitário Christus, Fortaleza, Brazil

*Diabetology & Metabolic Syndrome* 2019, **11(Suppl 1):**P208

**Background:** Exposure to intrauterine hyperglycemia increases the risk of adverse fetal outcomes in the neonatal period and also throughout the adulthood. A phenotype of insulin resistance with obesity, dyslipidemia, hypertension, diabetes as well as cognitive and psychiatric disorders are also related to intrauterine hyperglycemia. Careful follow-up of these children is recommended, but the ideal model for that is not well documented. In order to understand the demands of this population, our institution organized a protocol for the follow-up of these children. This study aimed to describe the profile of the offspring of diabetic mother attending ambulatorial follow-up program in our institution during the first year of evaluation.

**Methods:** A cross-sectional descriptive study with data of 106 children born to diabetic mother that answered the invitation to attend an annual visit at our institution during the period of June 2018 to July 2019. Data were collected through an interview and physical examination during the medical visit and review of mother’s medical records. An informed consent was obtained from all families. The statistical analysis was performed using R 3.6 program.

**Results:** On the mother’s profile, 16% had type 1, 26.4% had type 2 and 57.5% gestational diabetes. Insulin treatment was used by 49% and metformin by 36.7%. Cesarean was the delivery mode in 82.5% of them, with a mean gestational age at delivery of 37.7 ± 1.8 weeks and 17% were pre-term and 15.9% macrossomia. In regard to neonatal outcomes, mean birth weight was 3.291 ± 7.6 and fetal length of 49.1 ± 3. Respiratory distress was reported for 19.8% of the group, 11% were sent to Intensive care unit and 2 needed neonatal cardiopulmonary resuscitation. Jaundice was observed in 28% and Hypoglycemia in 10.3%. Malformations were found in 10.3% of the group: 2 (1.8%) Cardiovascular, 2 (1.8%) digestive; 1 (0.9%) Neurologic and 5 (4.7%) on musculoskeletal system.

**Conclusions:** The C-section rate was very high among the group, as it is expected in this high-risk population. Pre term delivery was also frequent, as it was jaundice and neonatal hypoglycemia. There was a considerable increase in frequency of respiratory distress syndrome in the offspring of diabetic mothers compared to general population (19.8% vs 0.5%). We are aware that this sample might have a selection bias, as families of children with a more committed health history tend to be more responsive to this kind of health care action.

### P209 Outcome indicators of assistance from a multidisciplinary diabetes care team

#### Tatiana Rebouças Moreira^1^, Lucilane Maria Sales da Silva^2^, Francisca Diana da Silva Negreiros^1^, Silvana Linhares de Carvalho^1^, Samila Torquato Araújo^1^, Maria de Jesus Nascimento Aquino^1^, Synara Cavalcante Lopes^1^, Renan Magalhães Montenegro Júnior^1^

##### ^1^Hospital Universitário Walter Cantídio/Universidade Federal do Ceará, Fortaleza, Brazil; ^2^Universidade Estadual do Ceará, Fortaleza, Brazil

*Diabetology & Metabolic Syndrome* 2019, **11(Suppl 1):**P209

**Introduction:** Diabetes mellitus (DM) is a public health problem of the 21st century, becoming a disease of high incidence and prevalence globally. Disease control is complex, involving drug therapy and lifestyle’s changes, with a view to preventing and delaying the onset of related complications. Thus, a multidisciplinary approach is required.

**Objectives:** To evaluate outcome indicators of assistance from a multidisciplinary outpatient diabetes care team at a university hospital in Ceará.

**Methods:**This is na evaluative study, according to the health evaluation benchmark proposed by Avedis Donabedian. The study sample was composed of 173 subjects. Data collection took place from August to October, 2018, through documentary analysis of the medical records, considering the records of the years 2017 and 2018. The database was analyzed in the statistical program *Statistical Package for Social Science* version 22.0, using descriptive statistics as well as variable association, with Chi Square and Wilcoxon tests, with p values ≤ 0.05 considered statistically significant. The study complied with the ethical guidelines for research involving human beings.

**Results**: As for the assisted users, there was a predominance of elderly women,106 (61.3%), with an average time of diagnosis of DM of 11.9 years. Hypertension and dyslipidemia were the main associated comorbidities. 171 (98.3%) patients were benefited from multiprofessional care (endocrinologists and specialized team of nurses, nutritionists and physiotherapists). The tracing of complications related to DM occurred in 90.2% of the users, and the prevalence of these was 68.2%. The outcome’s indicators suggested that, although the glycated hemoglobin did not reach the proposed goals, Wilcoxon’s test showed there was a significant reduction of the final parameters (7.9%) when compared to the initial ones (8.9%), as well as an increase in the proportion of users who reached glycemic control goals, 92 (53.2%) at the end of the evaluated period, when compared to the initial ones, 51 (29.5%), with Chi Square test.

**Conclusions**: Attention to DM requires a reflexive attitude about the importance of an effective multidisciplinary care model, seeking effective care strategies.

### P210 Pancreatic autoimmunity and beta cell function assessment: experience from a tertiary diabetes service

#### Rodrigo Do Rego Barros De Lucena Washington, Alessandra Saldanha Matheus Fernandes Da Costa, LucianneRigheti Monteiro Tannus, Marcela Martins Campos, Juliana Brazil Fontes Pascoal, Paula Ferreira Do Rego Barros, Roberta Arnoldi Cobas, Marília Brito Gomes

##### Universidade do Estado do Rio de Janeiro, Rio de Janeiro, Brazil

*Diabetology & Metabolic Syndrome* 2019, **11(Suppl 1):**P210

**Introduction:** Type 1 diabetes is characterized by the presence of immune-mediated destruction of beta cell. Moreover, pancreatic autoimmunity can be detected years before the diabetes (DM) diagnosis and the presence of multiple autoantibodies seems to predict the progression to beta cell loss and overt DM.

**Aims:** To describe the prevalence and clinical characteristics of DM-related autoimmunity and beta cell function.

**Methods:** We retrospectively reviewed patients with DM in whom autoimmunity and C-peptide were evaluated from 2016 to 2019. Clinical disease’s characteristics and laboratory evaluation with serum fasting C-peptide and pancreatic autoantibodies (anti-glutamic acid decarboxylase (GAD), insulin autoantibody, islet antigen, and anti-IA2) were assessed. A fasting C-peptide level ≥ 0.6 ng/mL was used to define “preserved beta cell function”. Results are presented as mean ± SD, median [interquartile range] or n (%). Statistical analysis was performed using SPSS-IBM 22.0 package.

**Results:** We included 127 subjects aged 23.4 ± 15.3 years, with age at DM diagnosis of 13 [10–25] years and DM duration of 37 [12–84] months. Anti-GAD, anti-IA2, anti-insulin and anti-islet were detected in 39%, 32%, 37.5% and 11.2% of the patients, respectively and ZnT8 was positive in 1 patient out of 3. DM duration and age at onset of DM were 26 (8–65) months and 13 (9–19.3) years, and 28 [8–58] monthsand 10 [8–14] years for patients with positive anti-GAD and anti-IA2, respectively. Among 61 patients with positive autoantibodies, the prevalence of 1, 2, 3 and 4 autoantibodies was 49.2%, 34.4%, 14.8% and 1.6%, respectively. Comparing those with (at least one positive autoantibody) and without autoimmunity, DM family history was present in 17.3% × 31.7% (p = 0.1), age at DM diagnosis was 12 (8–17) vs 15 (11–27) years (p = 0.02) and diabetic ketoacidosis at diagnosis was present in 32.8% × 19% (p = 0.07), respectively. C-peptide coupled with autoimmunity were assessed in 47 (37%) patients. Beta cell function was preserved in 66% of the patients and among them, 16.1% showed autoimmunity. In the group with low or absent pancreatic function (34%), autoimmunity was present in 75% of them (p < 0.01)

**Conclusion:** The vast majority presented only 1 positive autoantibody and the most prevalent autoantibody found were anti-GAD, followed by anti-insulin and anti-IA2. Those with positive autoantibodies had lower levels of C-peptide and a clinical onset of DM at earlier ages.

### P211 Patients with type 2 diabetes (T2D) on the maximum dose of insulin degludec/liraglutide (IDEGLIRA) achieve glycemic target: analyses from the dual program

#### Jung Yoon^1^, Luigi Meneghini^2^, Sultan Linjawi^3^, Pierre Serusclat^4^, Tina Vilsbøll^5^, Bue Agner^6^, Trine Hansen^6^, Lawrence Leiter^7^

##### ^1^Novo Nordisk Farmacêutica do Brasil; ^2^University of Texas Southwestern Medical Center, Dallas, USA; ^3^Coffs Diabetes and Endocrine Services, Coffs Harbour, Australia; ^4^Clinique Les Portes du Sud, Vénissieux, France; ^5^Gentofte Hospital, Hellerup, Denmark; ^6^Novo Nordisk A/S, Søborg, Denmark; ^7^University of Toronto, Toronto, Canada

*Diabetology & Metabolic Syndrome* 2019, **11(Suppl 1):**P211

**Introduction:** The efficacy and safety of IDegLira has been established in the DUAL clinical development program.

**Methods:** This post hoc analysis evaluated glycemic control in the subgroup of patients titrated to the maximum approved IDegLira dose of 50 units (U) (50 U insulin degludec + 1.8 mg liraglutide), from trials that evaluated IDegLira versus other comparators (DUAL I–V and VII).

**Results:** In all DUAL trials, baseline A1C was similar between IDegLira and comparator arms. In DUAL I–V, regardless of end-of-trial (EOT) doses (50 or < 50 U), more patients on IDegLira achieved the American Diabetes Association target of A1C < 7% versus monotherapy of basal insulin, glucagon-like peptide-1 receptor agonist or placebo comparators. In DUAL VII, compared with basal–bolus insulin therapy (insulin glargine 100 U/mL + insulin aspart ≤ 4 times daily [mean total daily insulin dose of 84 U at EOT]), the percentage of patients achieving an A1C < 7% was greater for patients on < 50 U of IDegLira and lower for patients at 50 U of IDegLira at EOT. For patients at 50 U and < 50 U of IDegLira, the mean change in A1C at EOT was numerically greater than or similar to comparators for all trials.

**Conclusions:** In conclusion, a high proportion of patients receiving the maximum approved dose of IDegLira are able to achieve good glycemic control.

### P212 Patients with type 2 diabetes treated with ideglira have a greater chance of achieving hba1c targets without hypoglycemia and weight gain than with basal insulin or basal bolus therapy

#### Giulliana Parisi Rodrigues^1^, Leonard E Egede^2^, Alexandra Bargiota^3^, Anthony J Cannon^4^, Lawrence A Leiter^5^, Alisa Schiffman^6^, Mattis F Ranthe^7^, Ankur Doshi^8^

##### ^1^Novo Nordisk Farmaceutica do BrasilLtda, São Paulo, Brazil; ^2^Division of General Internal Medicine, Froedtert & the Medical College of Wisconsin, Milwaukee, USA; ^3^Department of Internal Medicine, University of Thessaly, Volos, Greece; ^4^Endocrinology, Kennedy Health Alliance, Voorhees, USA; ^5^Li Ka Shing Knowledge Institute, St Michael’s Hospital, University of Toronto, Toronto, Canada; ^6^Novo Nordisk Inc., Plainsboro, USA; ^7^Novo Nordisk A/S, Søborg, Denmark; ^8^PrimeCare Medical Group, Houston, USA

*Diabetology & Metabolic Syndrome* 2019, **11(Suppl 1):**P212

**Introduction:** The safety and efficacy of insulin degludec/liraglutide (IDegLira) has been investigated in insulin-treated patients with type 2 diabetes (T2D) in randomized controlled treat-to-target trials, such as DUAL V and DUAL VII. The latest guidelines for management of T2D recommend individualizing HbA1c targets. Therefore, the HbA1c targets used in clinical practice and for quality metrics, such as the Healthcare Effectiveness Data and Information Set (HEDIS), are often higher than those used in clinical trials.

**Objective:** This post hoc analysis explored whether patients receiving IDegLira were more likely to achieve composite endpoints, incorporating alternative, higher HbA1c targets (< 7.5%/< 8.0%/≤ 9.0%), that are often used in clinical practice, compared with up-titrated IGlar U100 or IGlar U100 + IAsp ≤ 4 times daily (BB).

**Methods:** DUAL V and DUAL VII were 26-week, open-label, treat-to-target trials in which patients with T2D uncontrolled on 20–50 units IGlar U100 + metformin were included. Attainment of HbA1c targets without weight gain by week 26 and/or without hypoglycemia in the last 12 weeks were analyzed using a logistic regression model with a logit link. Missing data were imputed using last observation carried forward and using a mixed model for repeated measurements.

**Results:** At baseline, mean HbA1c was 8.4% and 8.2% for the IDegLira arm and up-titrated IGlar U100 arm, respectively, in DUAL V and 8.2% for both treatment arms in DUAL VII. In DUAL V, wherein IDegLira resulted in superior HbA1c reductions, a greater percentage of patients achieved HbA1c < 7.5% and < 8.0% with IDegLira vs IGlar U100. In DUAL VII, wherein non-inferiority was confirmed for HbA1c reductions with IDegLira vs BB, similar percentages of patients achieved all HbA1c targets with IDegLira vs BB. In both DUAL V and VII trials, over 99% of patients in both arms achieved HbA1c ≤ 9.0%. The odds of achieving the double composite endpoints of any HbA1c target without hypoglycemia or weight gain were significantly higher for IDegLira vs comparator in DUAL V and VII. Across all HbA1c targets, the odds of achieving the triple composite endpoint were significantly higher for IDegLira vs comparator.

**Conclusions:** Across a broad range of HbA1c targets, including those relevant to the HEDIS criteria, a greater percentage of patients succeed in achieving the clinically relevant endpoint of HbA1c targets without weight gain and/or hypoglycemia with IDegLira vs up-titrated IGlar U100 and vs BB.

### P213 Perceptions of nursing staff about their role in a diabetes camp

#### Naomi Santos Cerqueira^1^, Maria Gabriela Secco Cavicchioli^2^, Marco Antonio Vívolo^3^, Odete de Oliveira Monteiro^1^, Rebecca Ortiz La Banca^4^

##### ^1^Escola Paulista de Enfermagem, UNIFESP, São Paulo, Brazil; ^2^Okum Consultoria em Saúde, São Paulo, Brazil; ^3^NR Acampamentos, São Paulo, Brazil; ^4^Joslin Diabetes Center, Harvard Medical School, Boston, USA

*Diabetology & Metabolic Syndrome* 2019, **11(Suppl 1):**P213

**Introduction:** The first summer camp for youth with diabetes started in 1925, only 3 years after the discovery of insulin. Diabetes camps offer the opportunity for children and adolescents to meet people at same age, learn about diabetes, feel less loneliness and have fun in a safe environment. Nurses have a prominent role within camps, once these professionals are responsible for developing individual and collective activities in health education. However, the attributions of nurses in diabetes camps remains unclear.

**Objectives:** To explore the perceptions of a nursing staff about their role in a multiprofessional camp for children and adolescents with diabetes.

**Methods:** Since 1980 a Brazilian camp provides an annual summer session for diabetes care and education. We recruited nurses and undergraduate nursing students (N = 85) who participated as nursing staff in this camp from 2011 to 2018. Respondents answered 4 guiding questions about their role in the diabetes camp, during individual in person or videoconference interviews. Two research assistants transcribed the interviews and performed the content thematic analysis.

**Results:** Nursing staff reported their attributions before and during the camp with regard to diabetes care and nursing management. Before the camp, nurses deliver trainings on diabetes for the staff, as well as revise camp routines. During the camp, nurses perform/supervise blood glucose tests and insulin injections, stimulate carbohydrate counting and administer medications. Also, nurses carry a hypoglycemia kit for correction during the camp activities, participate in multidisciplinary meetings, conduct therapeutic play sessions and make the nursing assessment of each child. The nursing management role comprises controlling supplies, dividing the nursing staff according to the proposed activities and ensuring that there is a hypoglycemia kit in all activities.

**Conclusion:** Nursing staff of diabetes camp perceived their role as being responsible for several activities comprising diabetes care and management. These findings may aid diabetes research and nursing trainings aimed at improving the quality of the organization in diabetes camp.

### P214 Performance of continuous glucose monitoring in patients with poor-controlled diabetes mellitus

#### Maria de Lourdes Machado^2^, Bárbara Gabriela Gomes Silva^3^, Carla Raquel Pereira Oliveira^3^, Clara Simony de Souza Santos^3^, Matheus Cisneiros Silva de Oliveira^3^, Mariana Garcez Varela^1^, Naira Horta Melo^1^, Karla Freire Rezende^1^

##### ^1^Universidade Federal de Sergipe e IPESAUDE Diabetes Center, Aracaju, Brazil; ^2^Ipesaude Diabetes Center, Aracaju, Brazil; ^3^Universidade Federal de Sergipe, Aracaju, Brazil

*Diabetology & Metabolic Syndrome* 2019, **11(Suppl 1):**P214

**Introduction and objectives:** Diabetes Mellitus (DM) is a relevant health problem for many countries, in addition, increasingly prevalent, most of patients continue to have inadequate control and at risk of developing chronic complications. In recent years, technologies have been frequently used to assist in this challenge and continuous glucose monitoring (CGM) through the use of sensors such as FreeStyle Libre^®^ has shown a positive impact on reducing HbA1c levels. The objective of this study was to evaluate the performance of FreeStyle Libre^®^ in DM patients assisted at two Diabetes Centers.

**Methods and results:** This is a cohort study. Inclusion criteria were type 1 and type 2 DM volunteers assisted at IPESAUDE Diabetes Center and CEMAR who wished to optimize their glycemic control. Exclusion criteria were: HbA1c levels < 7.5%, glomerular filtration rate < 15 ml per minute, or difficulties in using CAAE technology. The sample was constructed by patients with previous diagnosis of T1DM and T2DM. Patients had access to FreeStyle Libre^®^ sensors and every 15 days returned to the units for sensor replacement and re-evaluation of a multidisciplinary team (endocrinologist, nurse, nutritionist and pharmacist) for a period of 3 months. At the first and last meeting, HbA1c was evaluated by the HPLC method. The study population consisted of 57 volunteers, 35 of whom T1DM and 22 of them, T2DM. Most participants were female (64.9%), with a mean age of 43.32 ± 17.74 years. Regarding the time of diabetes, 66.7% had more than 10 years of diagnosis. The mean HbA1c at baseline was 9.15% ± 1.53. At the end of the study, we achieved an average HbA1c of 7.88% ± 0.76 (p = 0.001) and an average reduction of 1.27%, which represents approximately 35 points in the average blood glucose; the largest reduction in T2DM patients (− 2.15%). The average HbA1c estimated by the sensor was 6.96% ± 0.82. The difference between serum HbA1c and HbA1c estimated by the sensor was 11.67%. There was only 1.03% increase in hypoglycemia, none of these was severe.

**Conclusion:** FreeStyle Libre^®^ can improve HbA1c in both T1DM and T2DM poorly controlled patients without increasing hypoglycemic episodes. The use of this technology should be considered as an alternative in approaching patients with difficulties in achieving adequate glycemic control.

**Financial support:** LIGA PHARMACOLOGY - UFS.

### P215 Peripheral diabetic neuropathy in patients treated at a referral service in the city of Aracaju

#### Lucas Moura Araújo, Matheus Cisneiros Silva de Oliveira, Clara Simony de Sousa Santos, Larissa de Oliveira Conceição, Letícia Luiza Gomes Marques, Elenalda Ferreira Santos, Marcelle Vieira Freire, Carla Raquel Pereira Oliveira

##### Universidade Federal de Sergipe, Aracaju, Brazil

*Diabetology & Metabolic Syndrome* 2019, **11(Suppl 1):**P215

**Introduction:** Peripheral Diabetic Neuropathy (PDN) is the most prevalent complication of Diabetes Mellitus (DM), and is associated with significant morbidity and mortality. It is characterized as a distal symmetric polyneuropathy that, in association with vasculopathies and other risk factors, may progress to Diabetic Foot. Objective: In view of this, the present study aimed to evaluate the risk of developing PDN in diabetic patients.

**Methodology:** For this, data were collected from 192 patients treated at a public reference service in the municipality of Aracaju-SE, through data from the Saving Diabetic Foot System (SiSPED). In this, PDN is classified based on two scores: neuropathic symptoms (ESN) and neuropathic impairment (ECN). Based on them, the probability of patients developing PDN through binominal logistic regression was determined.

**Results:** The prevalence of PDN in the study participants was 67.19%, mostly patients with T2DM. In addition, expressive sensitivity and specificity had been demonstrated, 93.5% and 34.6%, respectively, of the ESN and ECN scores regarding the diagnosis and prediction of PDN. Thus, it is estimated that the one-point increase in ESN and ECN represented a 2.98 and 9.35-fold increased risk for NDP development, respectively.

**Conclusion:** This reinforces the importance of SiSPED as a tool in diabetic patient follow-up, as it is easily applicable and expressively effective in diagnosing a highly prevalent complication associated with DM.

### P216 Peripheral polyneuropathy after bariatric surgery: independent association with higher triglyceride and decrease in high-density lipoprotein (HDl) cholesterol serum levels

#### Otto Henrique Nienov, Fernanda Dapper Machado, Helena Schmid

##### Programa de Pós-Graduação em Ciências da Saúde: Ginecologia e Obstetrícia - UFRGS, Porto Alegre, Brazil

*Diabetology & Metabolic Syndrome* 2019, **11(Suppl 1):**P216

**Introduction:** The most common neurological complication described after bariatric surgery (BS) is peripheral polyneuropathy (PPN). However, there is poor evidence about the impact of BS on the incidence and progression PPN.

**Objective:** To evaluate the incidence and progression of PPN in non-diabetic severe obese subjects after laparoscopic bariatric surgery (BS) and to seek for the presence of risk factors.

**Methods:** In this prospective cohort study, 341 subjects undergoing laparoscopic BS, Roux-en-Y gastric bypass (RYGB) or sleeve gastrectomy (SG), were evaluated for PPN by the Michigan Neuropathy Screening Instrument (MNSI) before and after 6 months of BS and divided according to presence (+) or absence (−) of PPN at baseline. Known causes of PPN, as type 2 diabetes mellitus, decompensated hypothyroidism, vitamin B12 deficiency and alcoholism, were excluded. Anthropometric, blood pressure, clinical, laboratory and physical activity data were collected before and after 6 months of BS.

**Results:** The prevalence of pre-BS PPN was 21.7% (n = 74) and decreased to 8.2% (n = 28) post-BS (p < 0.001). There was no difference between RYGB and SG in PPN prevalence’s before and after 6 months of BS (p = 0.859 and p = 0.714, respectively). When we looked to the two groups, from baseline to 6 months, for PPN (+) group (n = 74) the persistence of post-BS PPN was 18.9% (n = 14) and, for the PPN (−) group (n = 267) the incidence of post-BS PPN was 5.2% (n = 14). In the PPN (+) group, all the models we used in order to determine which factors were independently related to the progression of PPN were not significantly related. In the PPN (−) group, the incidence of post-BS PPN was independently associated with higher serum triglycerides levels (99.0 mg/dL versus 80.0 mg/dL; p ≤ 0.050) and higher decrease in high-density lipoprotein (HDL) cholesterol (HDL-C) (7.0 mg/dL versus 2.0 mg/dL; p ≤ 0.050). There was no difference between post PPN (+) and (−) groups from the baseline PPN (−) group related to preoperative serum triglycerides (p = 0.506) and HDL-C (p = 0.981) levels.

**Conclusion:** The prevalence of PPN decreased after 6 months of BS, but new cases of post-BS PPN appeared and they were independently associated with higher serum triglycerides levels and higher decrease in HDL-C. The PPN risk increased from 1.1 to 1.4% at each 1 mg/dL increase in triglycerides and 4.0% at each change of 1 mg/dL in HDL-C decrease. More studies are needed in order to support our findings.

### P217 Phase angle association, derived from electrical impedance with a1c in type 1 diabetic patients

#### Natália Fenner Pena, Paulo Augusto Miranda, Virgínia Capistrano Fajardo, Marina Manoela Meireles Corrêa, Jussara de Sousa Alonso, Jéssica da Cunha Ambrósio, Marcio Weissheimer Lauria, Henrique Oswaldo Da Gama Torres

##### UFMG - Universidade Federal de Minas Gerais, Belo Horizonte, Brazil

*Diabetology & Metabolic Syndrome* 2019, **11(Suppl 1):**P217

**Introduction:** Phase Angle (PA) has been related to the integrity of cell membranes, as a good prognostic indicator, with the general health and nutritional status of patients. Hyperglycemia has already been shown to be a common outcome seen in patients with type 1 diabetes mellitus (TDM1) and is capable of causing glycotoxicity and cell damage if not regulated; mainly in the long term. A1C is currently considered the gold standard for assessing metabolic control in T1DM patients, as the relationship between their increased levels (> 7%; which means blood glucose of 154 mg/dL or less) has been consistently demonstrated and the risk of chronic complications and adversities, and worsening prognosis in this population.

**Objective:** To evaluate the possible association of PA derived from electrical bioimpedance with A1C in patients with TDM 1.

**Methods:** This was a prospective cross-sectional study conducted in as ambulatory of public; in Belo Horizonte, Minas Gerais. The PA of the patients was calculated by the electrical bioimpedance exam on the day of the medical appointment. A1C was collected from medical records and classified as good control ≤ 7% and poor control > 7%. To compare the means of PA, according to the categorization of A1C; Student’s t-test was used after verifying normality by the Shapiro–Wilk test and homoscedasticity by the Levene test. For all analyzes, a significance level of 5% was adopted.

**Results:** Sixty-two patients were analyzed and 45.2% were male (n = 28) and 54.8% female (n = 34). The average age of the population was: 36 ± 11.3 years. 10.4%; (n = 5) of the individuals presented A1C ≤ 7% and 89.6% (n = 43) A1C > 7%. The average of PA observed among individuals with A1C ≤ 7% was 6.36 ± 1.06 degrees and among individuals with A1C > 7% was 5.93 ± 0.93 degrees; (p = 0.340). The average of standardized PA by gender and age (SPA) among individuals with A1C ≤ 7% was -0.67 ± 1.07 and among individuals with A1C > 7% was -1.66 ± 0.95 (p = 0.035).

**Conclusions:** Patients with TDM1 seem to have the most compromised health status when assessed by PA, since they have this parameter below the general population average. Nevertheless, those with poor control by A1C presented higher nutritional risk by SPA. Educational actions on diabetes and future studies are necessary, because, until now, this was the first study to evaluate this association in this population.

### P218 Platelet-rich plasma in people with diabetic foot: experience report

#### Amelina De Brito Belchior, Ricardo De Oliveira Lima, Nariane Monique Mendes De Lima, Michele Pontes Moreira, Shérida Karanini Paz De Oliveira

##### Universidade Estadual do Ceará, Fortaleza, Brazil

*Diabetology & Metabolic Syndrome* 2019, **11(Suppl 1):**P218

**Introduction:** Foot problems in people with diabetes are common worldwide, with economic, social and emotional repercussions for patients. Foot injuries result from a combination of several factors such as biomechanical stress, decreased skin perfusion, loss of sensation, and external trauma. Approaching and healing the lesion requires proper cleaning and use of appropriate cover. In this sense, there is Platelet Rich Plasma (PRP) as an alternative for wound healing. PRP is a platelet concentration in a small volume of plasma obtained by centrifuging whole blood.

**Objective:** to describe the experience of autologous PRP preparation and use in patients with diabetic foot.

**Methodology:** Experience report on the use of autologous PRP in diabetic foot patients treated at an outpatient clinic of a tertiary hospital from February to April 2018. Plasma was obtained according to the literature and adapted to the hospital laboratory centrifuge.

**Results:** To obtain autologous PRP, on the day of application, 30 ml of blood were collected by venipuncture of the patient himself. As a result, the samples were taken to the laboratory for plasma preparation and centrifugation and then injected directly into the lesions. The application occurred in seven weeks. From the first to the sixth, a care plan was executed, and injectable PRP was applied directly to the full extent of the lesions. In the seventh week, the lesions were reevaluated without the application of PRP. They obeyed the ethical and legal precepts advocated by the National Health Council.

**Conclusion:** The use of injectable autologous PRP in people with diabetic foot has been shown to be a low cost, quick preparation and easy application procedure.

### P219 Potassium behavior in diabetic patients with reduced glomerular filtration rate and initial mild hyperkalemia

#### Marcel Catão Ferreira dos Santos, Ana Karenina Carvalho de Souza, Rebecca Paiva de Araújo Silva, André Miller de Melo Henrique, Alyssa Evelyn Oliveira da Silva, Vinícius Silva Costa, Josivan Gomes de Lima, André Gustavo Pires de Sousa

##### Universidade Federal do Rio Grande do Norte(UFRN)/Hospital Universitário Onofre Lopes(HUOL), Natal, Brazil

*Diabetology & Metabolic Syndrome* 2019, **11(Suppl 1):**P219

**Introduction:** Diabetes Mellitus (DM) is a condition often associated with electrolyte changes. In the long-standing patient with DM, mild hyperkalemia is commonly associated with hyporeninemic hypoaldosteronism and it could lead concerns about the use of angiotensin-converting enzyme inhibitors (ACEIs) and angiotensin receptor blockers (ARBs).

**Objective:** The study aims to evaluate the behavior of potassium levels in patients with long-standing DM and its consequences on the management of these patients, especially regarding the associated antihypertensive treatment.

**Methods:** The longitudinal retrospective study performed analysis of medical records and laboratory data in patients with DM and initial potassium dosage ≥ 5.5 mEq/L, evaluating the behavior of this electrolyte from this initial value. A median follow-up of 14.5 (8–32) months was performed, with a analysis of 100 patients in total, of which 9 were excluded because they were on dialysis therapy, 13 because they were using spironolactone and 20 because they did not continue to follow potassium levels.

**Results:** A total of 58 patients were included, of which 5 were patients with type 1 DM and 53 with type 2 DM. Of these patients, 45 (77.6%) used ACEIs or ARBs. Baseline laboratory data: Potassium: 5.6 (5.5–5.8) mEq/L; Glycated hemoglobin A1c (HbA1c): 8.1 (7.4–9.3) % and eGFR: 42.93 (32.40–54.72) mL/min/1.73 m^2^. Aftermedian of 14.5 months, 42 (77.8%) of the patients were still using ACEIs or ARBs; potassium levels [5.6 (5.5–5.8 vs. 5.25 (4.8–5.5) mEq/L, p = 0.002)] reduced significantly compared to baseline; HbA1c [8.1 (7.4–9.3) % vs. 7.9 (6.9–9.3) %, p = 0.276] and GFR [42.93 (32.40–54.72) vs 42.03 (31.47–57.72)) mL/min/1.73 m^2^, p = 0.412] also reduced but it was not statistically significant. Potassium decreased in people with or without ACEI/ARB and there was no statistical difference between the groups [5.2 (4.8–5.5) vs. 5.4 (5.0–5.6), p = 0.35]; only 1 (2.4%) patient in the group with ACEI/ARB and 4 (8.3%) in the group without ACEI/ARB developed for potassium levels above 6 mEq/L (p = 0.398 between the groups).

**Conclusions:** In the analysis of this study, the monitoring of potassium levels of diabetic patients with reduced GFR and initial mild hyperkalemia showed a tendency to stability, despite the use of ACEI/ARB, favoring the maintenance of arterial hypertension treatment and nephroprotection with these drugs.

### P220 Potency and acuteness of a single small dose of sglt2 inhibitor in normal glucose tolerant individuals

#### Flora Tamires Moura Bandeira^1^, Marcel Catão Ferreira dos Santos^1^, Breno Carvalho Cirne de Simas^1^, Taisa de Abreu Marques Nogueira^2^, Ricardo Henrique Toscano de Menezes Filho^2^, Pedro Henrique Móra^2^, ÀlexEspinaltDaví Lima de Freitas^2^, Josivan Gomes de lima^1^

##### ^1^Universidade Federal do Rio Grande do Norte(UFRN)/Hospital Universitário Onofre Lopes(HUOL), Natal, Brazil; ^2^Universidade Potiguar (UNP), Natal, Brazil

*Diabetology & Metabolic Syndrome* 2019, **11(Suppl 1):**P220

**Introduction**: Sodium Glucose Cotransporter 2 inhibitors (SGLT2i) are associated with improved cardiovascular and renal outcomes in subjects with diabetes. Considering their potential role in patients without diabetes, it would be of particular interest to assess their acute effect on glycemia and glycosuria.

**Objective:** To assess the acute effect of a single dose of the SGLT2i dapagliflozin in healthy young adults.

**Methods:** Ten healthy young adults who volunteered for our study received half a pill of dapagliflozin 10 mg. We assessed urinary glucose before the drug was taken while subjects were fasting, and thereafter at 3.5, 24, 48 and 72 h(h). At 72 h only subjects who had detectable urinary glucose at 48 h were reassessed. If readings indicated glycosuria higher than 1000 mg/dL we registered it as 1000 mg/dL. Capillary Glycemia (CG) was assessed before the drug was taken, and then 3.5 and 24 h afterwards. The effects of dapagliflozin on glycosuria and CG were assessed using student’s T-test or Wilcoxon’s test if they were parametric or non-parametric, respectively. Spearman’s test was used to assess correlations between glycosuria and baseline fasting CG.

**Results:** Half of volunteers were male. Their median age was 20.6 (19–25) years, mean BMI 22.7 ± 3.5 kg/m^2^, and waist-to-hip ratio 0.90 ± 0.07. Baseline glycosuria was zero in all subjects and mean fasting CG was 96.3 ± 8.7 mg/dL. At 3.5 h, 1000 mg/dL glycosuria was detected in all samples; CG decreased to 89.2 ± 9.3 mg/dL (mean difference 7.1, 95%CI 1.4–12.8, p = 0.019). At 24 h, 8 out 10 subjects persisted with glycosuria (median was 1000 mg/dL [0–1000, p = 0.001] as compared with baseline levels). All male subjects persisted with urinary glucose readings at 1000 mg/dL; the mean CG was 94.9 ± 6.5 mg/dL, not significantly lower than at baseline (p = 0.324). At 48 h, 5 subjects (all male) still had glycosuria (median 230 mg/dL, p = 0.038 as compared with baseline). At 72 h glycosuria normalized in all volunteers who had urine samples tested. One subject did not provide urine sample for testing at 72 h. All correlations between baseline fasting CG and glycosuria were nonsignificant.

**Conclusions:** Dapagliflozin exerts acute effects on healthy subjects, lowering capillary glucose and inducing glycosuria. This could indicate a potential role for SGLT2i in the prevention of type 2 diabetes mellitus.

**Financial support:** AstraZeneca provided dapagliflozin samples used in this study.

### P221 Precocious use of continuous subcutaneous insulin infusion in infants and preschool children is related to less short-term glycemic variability and HBA1c on the goals

#### Monica Andrade Lima Gabbay, Maria Gabriela S Cavicchioli, Paula Maria Pascali, Tarcila B Ferraz de Campos, Vanessa Araujo Montanari, Carolina Sallorenzo, Dalva Oliveira, Sergio Atala Dib

##### Universidade Federal de São Paulo, São Paulo, Brazil

*Diabetology & Metabolic Syndrome* 2019, **11(Suppl 1):**P221

**Introduction:** Type 1 Diabetes (T1D) in young children is rare but seems to be growing in the last decade. Recently ISPAD, states that continuous subcutaneous insulin infusion (CSII) is the recommended therapy for children under the age of seven because of the risk of hypoglycemia, low insulin doses requirement and glycemic variability. **Aim**: To retrospectively evaluate CSII parameters of 28 infants and preschoolers divided according to A1c goal for age (< 7.5%).

**Methods:** Carelinkpro (Medtronic) and Accu-chekSmartpix (Roche) data upload. Children with Roche CSII did 6.8 SMBG/day.

**Results:** Group: A1c < 7.5% (7.0 ± 0.5%); 8 children and Group 2: > 7.5% (8.0 ± 0.4%); 20 children. Group 1 got started CSII at a lower average age (1.9 ± 1.2 years vs 3.1 ± 1.1 years; p = 0.02), had less DKA at diagnosis (58.0% × 87.0%; p = 0.00), more symptomatic days before diagnosis (11.8 ± 6.4 vs 5.3 ± 4.8; p = 0.03). In the last visit, group 1 had more time in range (59.8 ± 15.0%; 43.4 ± 9.8% × p = 0.02);less time above 180 mg/dl (34.6 ± 15.0% vs 52.0 ± 11.0% p = 0.01) lower FPG (160.0 ± 25.0 vs 181.0 ± 36.0 mg/dl; p = 0.05); lower mean glucose intra-day (149.0 ± 21.5 vs 172.0 ± 37. mg/dl; p = 0.04); lower total daily insulin dose per kg (0.79 ± 0.1 vs 0.62 ± 0.1 u/kg/dia; p = 0.04). We found no difference between groups in time in hypoglycemia, coeffience of variation, bolus/day, insulin carbo ratio (mean of 23.3 ± 86 g) and correction fator (mean 156 ± 56.0 mg/dl). In Group 1 A1c demonstrated positive correlation with mean glucose, standard desviation, time in hyperglycemia and negative correlaction with time in range and age that got started CSII. Group 2 A1c was correlated only with mean glucose and standard desviation.

**Conclusion:** Precocious use of CSII in Infants and preschool children is related to less short-term glycemic variability and better HbA1c

### P222 Pregnancy planning in women with diabetes: an actual reality?

#### Roberta Yukari Imai^1^, Patrícia Teófilo Monteagudo^2^, Rosiane Mattar^3^, Sérgio Atala Dib^2^, PatriciaMedici Dualib^2^, Bianca de Almeida Pititto^4^

##### ^1^Escola Paulista de Medicina (EPM), Universidade Federal de São Paulo (UNIFESP), São Paulo, Brazil; ^2^Disciplina de Endocrinologia, Escola Paulista de Medicina, Universidade Federal de São Paulo, São Paulo, Brazil; ^3^Disciplina de Obstetrícia, Escola Paulista de Medicina, Universidade Federal de São Paulo, São Paulo, Brazil; ^4^Departamento de Medicina Preventiva, Escola Paulista de Medicina, Universidade Federal de São Paulo, São Paulo, Brazil

*Diabetology & Metabolic Syndrome* 2019, **11(Suppl 1):**P223

**Introduction and objectives:** Pregnancy planning in women with previous diabetes mellitus (DM) is relevant considering risks of maternal and fetal complications. The present study aims to evaluate current pregnancy of women with previous DM regarding to the planning of pregnancy, contraception counselling and glycemic control when they got pregnant.

**Methods:** The study included 39 pregnant women with Type 1 DM (T1DM), Type 2 (T2DM) or other DM (ODM) in an outpatient care of Diabetes and Pregnancy at the *Centro de DM*, from October/2018 to May/2019. A standardized questionnaire was used and data were collected from the medical records. The continuous variables were represented by mean (standard deviation) and the categorical variables, by percentages (n). Comparisons of variables of interest (Student t-test or Chi square test, respectively) were performed between two groups: planned and non-planned pregnancy.

**Results:** 74.4% (29) had T1DM, 23.1% (9) T2DM and 2.6% (1) ODM, with mean(SD) age of 28.5 (6.1) years and body mass index of 26.7 (6.7) kg/m^2^; 64.1% (25) studied ≤ 13 years. Regarding previous follow-up, 56.4% (22) were from DM Center, 25.6% (10) SUS, 7.7% (3) private clinics and 10.3% (4) did not have any. For 53.8% (21) this pregnancy was the first one; 82.1% (32) related that had received contraception counselling before getting pregnant (59.4% (19) gynecologists, 25% (8) endocrinologists and 15.6% (5) other), however, 25.6% (10) reported that had planned the current pregnancy and 41% (16) reported regular contraceptive use before the first pregnancy and 31.6% (6) after the last one. Before the current pregnancy, the HbA1c was 9.1% (2.0). Who planned gestation had a borderline higher education (≥ 14 years, 60% vs. 27%, p = 0.06) and were older (32(5) vs. 27 (6) years, p = 0.019) than those who had not planned it. There was no difference in planning pregnancy related to contraception counselling, site of follow-up, type of DM, father’s schooling or pre-gestational HbA1c.

**Conclusion:** Although many women reported having received contraception counselling, the planning and glycemic control before pregnancy in women with DM did not prove to be a reality in our setting. Nevertheless, higher age and schooling may be contributing factors in planning pregnancy. This scenario shows the importance of an effective contraception counselling and reinforces the relevance of DM management in women in the reproductive period.

**Financial support:** The São Paulo Research Foundation (FAPESP).

### P223 Pregnancy prevention e-book development and validation for diabetic teenagers

#### Márcia Danielle Sodré de Almeida^1^, Aleida Nazareth Soares^1^, Alexandra Dias Moreira^2^, Janice Sepúlveda Reis^1^, Suelen Rosa de Oliveira^1^, Helem de Sena Ribeiro^1^, Suzana Veloso da Rocha Arabi^1^, Tatiane Géa Horta Murta^1^

##### ^1^Ensino e Pesquisa Santa Casa BH, Belo Horizonte, Brazil; ^2^Escola de Enfermagem. UFMG, Belo Horizonte, Brazil

*Diabetology & Metabolic Syndrome* 2019, **11(Suppl 1):**P223

**Introduction:** Diabetes Mellitus (DM) is a worldwide public health problem that affects people of all walks of life. In addition to the main complications of DM, such as macro and microvascular, ocular, renal and neurological lesions, it is important to consider that DM is a recognized reproductive risk factor. Therefore, it is necessary to develop educational strategies aimed at contraception and reproductive planning, especially in adolescence.

**Objective:** to describe the development and validation process of an e-book for contraceptive methods concerning adolescents with diabetes mellitus.

**Method:** qualitative research, developed in seven different steps: bibliographical survey, elaboration of the educational material, calculation of readability indexes, validation by a judge panel, discussion among specialists, validation of the e-book by the target population, and final discussion among specialists. The validation was performed by eleven judges and the face-to-face test was performed by thirteen adolescents. A minimum content validity coefficient of 0.80 was considered.

**Results:** The content validity coefficient of the e-book was 0.95. The suggestions by the judges and adolescents were discussed by the specialists and the required modifications were made.

**Conclusions:** the e-book was elaborated, adapted and validated in relation to content and relevance, being suitable for use in counseling adolescents with diabetes on contraception.

### P224 Prevalence and associations of peripheral polyneuropathy in grade ii and iii obese patients without diabetes before and after 1 year of bariatric surgery

#### Fernanda Dapper Machad, Otto Henrique Nienov, Helena Schmid

##### Programa de Pós-Graduação em Ciências da Saúde: Ginecologia e Obstetrícia - UFRGS, Porto Alegre, Brazil

*Diabetology & Metabolic Syndrome* 2019, **11(Suppl 1):**P224

**Introduction:** Peripheral Polyneuropathy (PPN) is a diabetes complication also described in pre-diabetic patients and in patients with obesity and metabolic syndrome. For obese patients it is not clear which factors are associated with PPN prevalence.

**Objective:** Our aim was to evaluate PPN prevalence in grade II and III obese patients without diabetes and in subjects who had undergone a bariatric surgery (BS) 1 year before.

**Methods:** On a cross-sectional study we evaluated 710 grade II and III obese subjects without diabetes and 525 subjects after BS, Roux en-Y gastric bypass (RYGB) or sleeve gastrectomy (SG) for PPN related to obesity by Michigan Neuropathy Screening Instrument (MNSI). The cut point used for the diagnosis of PPN was ≥ 2.5 signs plus a symptom (79% of specificity and 61% of sensibility). Patients with known causes of PPN, as type 2 diabetes mellitus, decompensated hypothyroidism, vitamin B12 deficiency and alcoholism, were excluded.

**Results:** Between obese participants PPN prevalence was 20.8%, while on post-BS was 10.3% (p < 0.001). On post-BS, there was not significant difference in PPN prevalence between the two kinds of surgery (10.9% for RYGB and 9.7% for SG; p = 0.742). On obese patients, PNP was associated with postmenopausal status (p < 0.001), age (p < 0.001), stature (p = 0.0.14) and waist circumference (p = 0.021). Post-BS, PPN was associated with weight (p = 0.024), stature (p = 0.011), waist circumference (p = 0.042), fasting glucose levels (p = 0.007) and triglycerides levels (p = 0.011). On two Poison regression models, postmenopausal status (p < 0.001, CI9% % 3.253 (1.854–5.709)), age (p < 0.001, CI95% 1.030 (1.014–1.047)) and stature (p = 0.007, CI95% 1.037 (1.010–1.066) were independently associated with PPN on obese. On the other hand, on two post-BS models only fasting glucose levels were independently associated with PPN (p = 0.016, CI95% 1.030 (1.006–1.060).

**Conclusion:** One year after BS PPN prevalence is lower than in severe obese subjects without diabetes. In the obese group, being postmenopausal increased by 3.2 times the chance of PPN, each year of age increased it by 3% and, each cm of a higher stature increased it by 3.7%. On post-BS group, each mg/dL increase on blood glucose levels increased by 3.3% the chance of PPN.

### P225 Prevalence and gravity of diabetic distal polyneuropathy in plantar ulcer appearance

#### Cristiany Azevedo Martins^1^, Camylla Bandeira Miranda^1^, Hortência Diniz Teixeira^1^, Geyse Gomes de Oliveira^1^, Priscila Sampaio Silva^2^, Débora Fidélis de Oliveira^2^, Daniela GardanoBucharles Mont’Alverne^2^, José Carlos Tatmatsu Rocha^2^

##### ^1^Hospital Universitário Walter Cantídeo -UFC, Fortaleza, Brazil; ^2^Universidade Federal do Ceará, Fortaleza, Brazil

*Diabetology & Metabolic Syndrome* 2019, **11(Suppl 1):**P225

**Introduction:** Diabetic neuropathy is the most important factor for the onset of plantar ulcer in individuals with diabetes mellitus (DM), and is present in 50% of patients with T2DM, the most common form being distal symmetric polyneuropathy or peripheral diabetic polyneuropathy (PND).

**Objective:** To verify the prevalence and severity of PND in the onset of plantar ulcers in diabetic patients.

**Methods:** An observational, cross-sectional, comparative and quantitative study carried out at AnastácioMagalhães Health Clinic in Fortaleza, Ceará, Brazil, from June 2018 to May 2019, with diabetic patients with plantar ulcer (G1) and diabetic patients. without plantar ulcer (control group—G2). Consecutive, non-probabilistic, convenience sample, where participants in the control group were matched for age, sex, and years of pathology with G1. Afterwards, the participants underwent the Diabetic Distal Polyneuropathy (PND) Diagnostic Scale, which evaluated the Neuropathic Symptom Score (ESN) and the Neuropathic Impairment Score (NEC). From this result they were classified as painful PND, with risk of ulceration, mild, moderate or severe asymptomatic and neuropathic pain. Results were expressed as mean ± standard deviation and/or percentage. For associations, t test for independent samples and Chi square test were performed, being considered as statistically significant when p less than or equal to 0.05.

**Results:** We evaluated 52 diabetic patients, 26 in G1 and 26 in G2, 30 men (15 in each group). 25 (96.2%) patients in G1 had PND and 18 (69.2%) in G2, with no statistical difference (p = 0.692). In the PND classification, a statistically significant difference was observed between the groups (p = 0.019), and in G1 57.7% (n = 15) presented PND with risk of ulceration and 15.4% (n = 4). with painful PND. In G2, 23.1% (n = 6) presented neuropathic pain PND and 19.2% (n = 5) mild asymptomatic PND. Only 11.5% (n = 3) had PND at risk of G2 ulceration.

**Conclusions:** There was a high prevalence of PND in the population, regardless of the group, but the severity of PND was higher in the plantar ulcer group, and was directly related to its onset.

### P226 Prevalence and risk factors of diabetic foot in a population assisted at the world diabetes day in Presidente Prudente-SP

#### Jaqueline Dias Cardoso, Carolina de Castro Rocha Betônico, Izabela Maria de Almeida Miotti, Ana Caroline Cavallari, Gabriela Cristiane Rodrigues Juki Miotto

##### Faculdades Adamantinenses Integradas, Presidente Prudente, Brazil

*Diabetology & Metabolic Syndrome* 2019, **11(Suppl 1):**P226

**Introduction:** Diabetes mellitus (DM) is a metabolic disease associated with several chronic complications. The diabetic foot is one of the main complications of DM. In Brazil, few population studies evaluate the prevalence of the diabetic foot complications and its associated risk factors.

**Objective:** To identify the prevalence of the diabetic foot, as well as the presence of risk factors for development of diabetic foot ulcers and amputations in the DM population assisted at the World Diabetes Day Campaign (24th of November of 2018) in Presidente Prudente-SP and their epidemiological characteristics.

**Methods:** Descriptive cross-sectional and population study was performed based on the collected data at diabetes campaign. The participants with DM had their feet examined and classified according to the presence of amputation, insensitive foot, both new and old foot ulcers, deformity and mycosis. For vascular examination, tibial and pedal pulses were assessed. The used criteria were the analysis of the loss of the protective sensation (LOPS), peripheral vascular disease (PVD) and both deformity and previous ulcers or amputation. It was assessed from 0 to 3 stages, described as: 0 the absence of LOPS and PVD and 3 previous amputation and ulcers.

**Results:** Among the 943 participants that fulfilled the inclusion criteria,58.4% had DM, 2.6% diagnosed at the campaign, and *foot examination* was *performed* on 46.5% of all the participants. Among them, 67.9% were over 60 years-old, 48.5% with DM diagnosis over 10 years ago and 37.1% on insulin therapy. The prevalence of the diabetic foot was 43%. Foot injuries were found in 23.5% on stage 1, 16.4% on stage 2 and 3.2% on stage 3. Also, there were 28.0% cases of sensitivity alteration to the monofilament test, 28.5% and 19.4% with reduced or absence of tibial and pedal pulse respectively, 21.4% cases of deformity, 6.4% with old ulcerations, 2.3% with new ulcerations and 0.9% with amputations. Almost half of the patients (46.2%) had mycosis on their feet and 43.0% participants used inadequate footwear. Regarding the DM participants, 84.0% reported that never had their feet examined previously, and among them 37.3% were diagnosed with diabetic foot.

**Conclusions:** Them*ajority people with DM at the campaign have never had their feet* examined during a consultation before. Health professionals must be aware about the importance of foot exam and patient education focused on foot care to reduce amputations among patients with DM.

### P227 Prevalence of diabetes in liver transplant patients

#### Claudia Pinheiro Sanches^1^, Helen Carolina Perussolo Alberton^2^, Alcindo Pissaia Junior^2^, Andressa Miguel Leitão^1^

##### ^1^Centro de Diabetes Curitiba - Hospital Nossa Senhora das Graças, Curitiba, Brazil; ^2^Hospital Nossa Senhora das Graças, Curitiba, Brazil

*Diabetology & Metabolic Syndrome* 2019, **11(Suppl 1):**P227

**Introduction:** Diabetes (DM) is present in 15% of cirrhotic patients. The main causes of liver transplantation(TX) are hepatitis C (HCV) and nonalcoholic liver steatosis(NASH), both strongly associated with the development and progression of DM. Cirrhotic patients have insulin resistance due to increased hepatic gluconeogenesis, impaired muscle glucose uptake and hyperinsulinemia. When liver TX is indicated, the prevalence of DM reaches 53%. Non-diabetic patients may present new-onset diabetes after transplantation(NODAT) due to stress hyperglycemia, use of corticosteroids and immunosuppressants. Few studies correlate the prevalence of DM with the determinant etiologies of TX (HCV, hepatocarcinoma, NASH, alcohol), and NASH is the only growing cause today. NODAT is estimated to be more likely in previously diabetic patients, prolonged use of corticosteroids or in HCV transplant recipients. The impact of DM on liver TX outcome is assessed by patient survival rate, with higher morbimortality among diabetic recipients. However, although Brazil has the second position of liver TX in the world, there are no data available to estimate the prevalence of DM or NODAT in our population, and further studies on the subject are needed. 

**Objective:** Evaluation of the DM prevalence or NODAT during 7 years of follow-up at a referral service in Curitiba-PR.

**Methods:** Retrospective cross-sectional study through the analysis of 85 records of patients submitted to liver TX, from 2011 to 2017, after approval by an ethics committee. Analysis of variance of quantitative variables, prevalence by bimodal test and correlation analysis between causes of TX and DM progression by Chi square test.

**Results:** Among the 85 liver transplanted patients, the main etiologies were HCV(32.9%), alcohol(20%) and NASH(17.6%), highlighting that, in 2017, NASH was the main cause of TX, which is a worldwide trend. The prevalence of pre-TX DM was 41.2%(35), increasing to 60%(51) after TX(p < 0.001). Among patients with HCV, 50% (14) were pre-TX diabetics, and NODAT increased to 71.4% (20)(p = 0.031), corroborating data of higher prevalence of DM in this population.

**Conclusions:** Liver TX from NASH has been increasing possibly associated with the obesity pandemic. The increased prevalence of NODAT is closely correlated with morbimortality and organ rejection, as well as predicting higher cardiovascular risk and metabolic syndrome. An integrative approach is necessary to improve survival and quality of life of these patients.

### P228 Prevalence of diabetic cheiroarthropathy and its relation with diabetic peripheral neuropathy among patients of a referral outpatient clinic in the city of Fortaleza - Ceará

#### Geyse Gomes de Oliveira^1^, Francisca Janiele Ribeiro Tavares^2^, Wescley Franco Lima^2^, Emanuel Davi Simões dos Santos^2^, Georgia de Melo Castro Gondim^1^, Taynara Guedes da Silva^1^, José Carlos Tatmatsu Rocha^2^, Daniela GardanoBucharles Mont’Alverne^2^

##### ^1^Hospital Universitário Walter Cantídio| Universidade Federal do Ceará, Fortaleza, Brazil; ^2^Universidade Federal do Ceará, Fortaleza, Brazil

*Diabetology & Metabolic Syndrome* 2019, **11(Suppl 1):**P228

**Introduction:** Musculoskeletal disorders are present in 36–75% of people with diabetes mellitus (DM). Diabetic cheiroarthropathy (DC), also known as limited joint mobility syndrome, presents with flexion deformity of the fingers, thickening of the skin, periarticular connective tissue and palmar fascia, limiting joint extension and may affect other joints in more severe cases.

**Objective:** To verify the prevalence of QC and its relation with diabetic peripheral neuropathy (DPN) in patients of a referral outpatient clinic in the city of Fortaleza, Ceará.

**Methods:** A cross-sectional quantitative approach study was conducted with diabetic patients in follow-up at a *university hospital* in Cearábetween the 2017 and 2018. All participants were evaluated for age, gender and *duration of DM.* DPN was assessed by Neuropathy Symptom Score (NSS), Neuropathy Disability Score (NDS) and classified according to its severity. The presence or absence of DC was obtained by the prayer sign test and the tabletop test. Results were expressed as mean ± standard deviation and/or percentage. For associations, t-test was performed for independent samples and for correlations, Chi square was performed, being considered as statistically significant when p less than or equal to 0.05.

**Results:** We evaluated 458 individuals, most of them female (n = 292, 63.8%), with a mean age of 62.9 ± 11 years old, and mean *disease duration*15.8 ± 9.4 years. 127 patients (27.7%) had no DPN. Of the 331 who had DPN, 170 (51.4%) were classified as neuropathic pain type DPN, while 50 (15.1%) had risk of ulceration type DPN. The prayer sign was present in 256 participants (55.9%), while the tabletop test was positive in 185 individuals (40.4%). There was an association between the prayer sign with the tabletop test (p = 0.00001), prayer sign and DPN (p = 0.004) and the tabletop test with DPN (0.00001). It was also observed positives associations between prayer sign with age (p = 0.017) and with the years of *disease duration*(p = 0.024). The prevalence ratio of those with DC having DPN in participants was 1.33 using the prayer sign.

**Conclusion:** A high prevalence of DC was observed in the participants, which was related to age and time since diagnosis. The presence of DPN is also correlated with DC. This is overlooked in assessments and can cause major functional limitation in patients with DM.

### P229 Prevalence of diabetic foot in a primary care unit in southern São Paulo, Brazil

#### Giovana Alves de Oliveira Souza, Regis Rodrigues Vieira, Daniele Scherbaty, Camila Nascimento Monteiro

##### Hospital Israelita Albert Einstein, São Paulo, Brazil

*Diabetology & Metabolic Syndrome* 2019, **11(Suppl 1):**P229

**Introduction:** Diabetes Mellitus affects about around 12.5 million people in Brazil. The country is the fourth in the world in number of people with this diagnosis. The overall prevalence of diabetic foot is 6.4% on average and amputations are 10 to 20 times more common in the diabetes population than in the general population.

**Objective:** To evaluate the prevalence of diabetic foot in a Primary Health Care Unit in Brazil

**Methodology:** Descriptive cross-sectional study, with a total of 1433 patients, with type 2 diabetes mellitus from dataset of a primary care unit in the south of São Paulo, Brazil. For 95% confidence interval, 5% sample error and 50% heterogenicity, we analyzed 380 altered cases in the last 12 months and were related to the time of diagnosis. The study was approved by the research ethics committee.

**Result:** Of the 380 patients, 29.5% had some degree of neuropathy, of these about 15% had less than 5 years of diagnosis. One third of patients with less than 1 year of diagnosis had some degree of commitment. And over 5 years of diagnosis about 5% of patients with Type 2 Diabetes Mellitus developed at least severe ulcers.

**Conclusion:** About 30% of patients diagnosed with Type 2 Diabetes Mellitus in a Primary Care Unit in southern São Paulo are estimated to have a change in their feet.

### P230 Prevalence of gastroparesis-related symptoms among patients with type 1 and type 2 diabetes

#### Mayara Ponte Madeira^1^, Pedro Igor da Silva Porfirio^2^, Luís Felipe Viana Correia^3^, Letícia de Sousa Guerin^2^, Bruna Sobreira Kubrusly^2^, Renan Galvão Ozório^2^, Angela Delmira Nunes Mendes^1^, Renan Magalhães Montenegro Júnior^1^

##### ^1^Hospital Universitário Walter Cantídio - HUWC/UFC, Fortaleza, Brazil; ^2^Universidade Federal do Ceará, Fortaleza, Brazil; ^3^Liga Acadêmica de Diabetes - Universidade Federal do Ceará, Fortaleza, Brazil

*Diabetology & Metabolic Syndrome* 2019, **11(Suppl 1):**P230

**Introduction:** Diabetic neuropathies are one of the most prevalent chronic complications among subjects with diabetes mellitus. Gastrointestinal neuropathies may involve all extension of the gastrointestinal tract. Gastroparesis is characterized by delayed gastric emptying in the absence of mechanical obstruction of the stomach. The prevalence and incidence of gastroparesis in patients with type 1 diabetes (T1DM) and type 2 diabetes (T2DM) has varied widely between studies.

**Objective:** To determine the prevalence of gastroparesis symptoms in T1DM and T2DM patients.

**Methods:** Cross-sectional study for a period of five weeks in a tertiary referral hospital of Ceará. The Gastroparesis Cardinal Symptom Index (GCSI) questionnaire was applied and laboratory tests and anthropometric data were analyzed. The GCSI score ranges from 0 (none) to 5 (very severe). Patients with gastroparesis symptoms were those with a score greater than or equal to 1.9. It was approved by the Research Ethics Committee.

**Results:** We evaluated 98 patients, but one was excluded because he was using GLP1 analogue. T1DM was observed in 27 (28%) and T2DM in 70 (72%). When analyzing T1DM patients, 5 (18%) had GCSI ≥ 1.9. Median GCSI was 3.0 (2 to 3.2). The median age was 28.7 years (22 to 46 years), 4 (80%) female and 1 (20%) male and 4 (80%) had less than 10 years of diagnosis. The values of glycated hemoglobin (8.5 vs 7.5%) and albumin/creatinine ratio in isolated urine (A/C ratio) (461.3 vs 22 mg/g) were higher in the symptomatic group (p = 0.33 and 0.5). There were no difference in creatinine clearance and waist circumference in both groups (p = 1.0 and 0.67). When we analyzed T2DM subjects, 11 (15.7%) had symptoms. GCSI median 2.67 (2.11 to 4.5). The median age was 57.3 years (47 to 69 years), 8 (73%) female and 3 (27%) male and 8 (72.7%) had more than 10 years of diagnosis. The glycated hemoglobin (9.8 vs 8.8%), creatinine clearance (88.6 vs 82) and waist circumference (105 vs 98.5 cm) were higher in symptomatic group (p = 0.28 and p = 0.12). Creatinine clearance values were higher (88.6 vs 82, p = 0.55). There were no difference in A/C ratio and BMI in both groups (p = 0.82 and 0.23).

**Conclusions:** There was observed a significant frequency of gastroparesis symptoms and it was associated with worse glycemic control. This condition affects the quality of life of diabetic individuals and leads to complications and it is important be aware of these complaints.

### P231 Profile of people undergoing diabetes mellitus-related amputations

#### Francineide Pereira Da Silva Pena^1^, Antônio Odilon do Espírito Santo Teixeira^2^, Odilson Rocha Alves^2^, Samuel dos Santos Miranda^2^, José Luis da Cunha Pena^1^, Érika Tatiane de Almeida Fernandes Rodrigues^1^, Vanessa da Silva Oliveira^1^, Luan de Souza Andrade^3^

##### ^1^Universidade Federal do Amapá, Macapá, Brazil; ^2^Hospital de Emergencia; ^3^Faculdade Fama, Macapá, Brazil.

*Diabetology & Metabolic Syndrome* 2019, **11(Suppl 1):**P231

**Introduction:** Diabetes Mellitus-DM is one of the largest health emergencies of the 21st century. The chance of a person with DM developing foot ulcer reaches 25%. It is believed that every 30 s lower limb amputation occurs in a proportion of 50% to 75%, and this is due to lack of care; neuropathy; peripheral vascular disease. People with DM are 15 times more likely to have lower limb amputation compared to those without the disease.

**Objective:** To describe the sociodemographic and clinical profile of people undergoing DM-related amputation.

**Methods:** Cross-sectional, descriptive and quantitative study, conducted at an Emergency Hospital, approved by the Research Ethics Committee, opinion no: 2.416.792. The population was 330 cases attended from 2011 to 2017, whose sample was 106 cases, but 15 were excluded because they had crying, sadness, and anguish in the interview, as it would bias the results. Of the remaining 91 people 5 refused to participate, 2 the address was not found and 4 has a mental disorder, so 80 cases were studied. For data collection, a questionnaire with sociodemographic and clinical identification was used.

**Results:** Male predominance 72.5%, mean age 58 ± 10.4, married 35%, brown ethnicity 66.3%, retired and working in the formal market 41.3%, respectively. Schooling 12.5% illiterate and 52.5% had an incomplete elementary school. 50.0% have a history of smoking and 63.8% alcoholics. CLINICAL VARIABLES: Hypertension 62.5%, neuropathy 36.3%, retinopathy 35.0%. Time to seek assistance since initial manifestation 31.3% from 1 to 7 days and 21.3% from 8 to 14 days. Has DM2 98.8%, family history 69.6%, diagnosis over 10 years 63.8%, mean blood glucose 228.5 ± 91.2 mg/dl. Treatment: 20% NPH Insulin, 20% Metformin/Glibenclamide, 11.3% Metformin. Amputation Type: lower 72.5%, higher 27.5%. New amputation: did not resample 61.3%, resampled with time ≤ 1 year 31.3%. Meantime: from hospitalization (15k5 ± 7.1) days and last amputation (17.8 ± 16.4) months.

**Conclusion:** It was found that the sex most affected was male, age and length of stay relatively shorter than what is found in the literature. The alcoholism present in most of the participants, associated with the time demanded by the service, may have negatively influenced the lesion complication with amputation outcome. The expressive rates of reamputations ≤ 1 year show that there is no follow-up in the post-amputation period.

### P232 Puppet theater as a pedagogical strategy in the food and nutrition education of children in the early childhood education

#### Hugo dos Santos Silva Junior, Luciana Neri Nobre, Maylza de Fátima do Nascimento, Paola Aparecida Alves Ferreira, Isabela Maria Lemes Machado, Ana Cláudia Chaves, Andréia Soares de Carvalho, Edson da Silva

##### Universidade Federal dos Vales do Jequitinhonha e Mucuri (UFVJM), Diamantina, Brazil

*Diabetology & Metabolic Syndrome* 2019, **11(Suppl 1):**P232

**Introduction:** In recent years the prevalence of obesity and type 2 diabetes (T2DM) among children and adolescents has become a worldwide problem. To combat this problem in the school environment, actions of the ‘Diabetes in the School Project” to promote an program of Food and Nutrition Education. This Project uses diverse methodologies and educational resources, including puppet theater. The theater is able to sensitize students, challenge them to seek new knowledge and life experiences. Regarding the formal contents of the school, theater becomes a resource to arouse curiosity and a taste for research. The theater is capable of exercising attention, patience, solidarity and living with diferences.

**Objective**: To describe the development and utilization of a puppet theater as a pedagogical strategy in Food and Nutrition Education.

**Methods**: This is a descriptive study about the experience of a Diabetes Reference Center at Schools (DRCS) team working at a Municipality of Minas Gerais. The story of the theater was designed by the University’s Department of Nutrition and presented by teachers, professionals and undergraduate students from the interdisciplinary team of the DRCS. The theme addressed was healthy eating focusing on ultra-processed foods and organic foods. The actions were developed two stages: the first took place at the university and involved the creation of a children’s tale about the importance of healthy food and adaptation as a theater script. Subsequently, the play took place in the form of puppet theater. The second stage was with the presence of interdisciplinar team in the school, for the presentation of the theater and clarification of doubts with the children. The children were first grade students at a municipal public school, about 5 years old.

**Results**: The theater benefited about 150 students who were motivated and interactive during the activity. We found that healthy eating was reinforced and demystified with the puppet theater. After the theater a fruit salad was offered for positive reinforcement of the ideas presented at the theater. From this experience the DRCS adopted the puppet theater for use in public and private schools of the municipality.

**Conclusions**: With puppet theater we can show that health education is essential for the development and quality of life of children. In addition, approaching healthy eating with theater may be a different way of expressing the importance of addressing obesity and T2DM from Childhood.

### P233 Rapid response of 1,25(OH)_2_-vitamin D_3_ on calcium influx as a trigger for insulin secretion from rat pancreatic islets

#### Ana Karla Bittencourt Mendes, Paola Miranda Sulis, Fátima Regina Mena Barreto Silva

##### Universidade Federal de Santa Catarina, Florianópolis, Brazil

*Diabetology & Metabolic Syndrome* 2019, **11(Suppl 1):**P233

**Introduction:** Diabetes is a chronic disease which results in failure on insulin action or secretion. Previous studies have shown that 1,25-(OH)_2_ vitamin D_3_ (1,25-D_3_) plays a role in modulating and enhance glucose-stimulated insulin secretion of rat pancreatic islets. However, the mechanisms linking 1,25-D_3_ and insulin secretion are still not well understood.

**Objective:** To study the rapid response of 1,25-D_3_on ^45^Ca^2+^ influx and its mechanism of action for insulin secretion in rat pancreatic islets.

**Methodology:** Adult male Wistar rats were used (CEUA/UFSC/2119280317). Pancreatic islets were isolated by pancreas dissection with KRb‐HEPES buffer and collagenase. Static insulin was dosed by ELISA. For calcium influx, isolated pancreatic islets were incubated in KRb‐HEPES buffer, 5 mM glucose, 0.1 μCi/mL ^45^Ca^2+^ for 60 min. After that, islets were incubated with/without 1,25-D_3_ (1 ηM – 60 s). The channel activator/blocker were added at 45 min of incubation. Aliquots were taken for radioactivity measurement. Proteins were quantified by the Lowry method.

**Results:** 1,25-D_3_ stimulated calcium influx and increased insulin secretion in pancreatic islets. The stimulatory effect of 1,25-D_3_ was blocked by glibenclamide and diazoxide, a K^+^-ATP channel inhibitor and activator, respectively. When vesicular transport was blocked by N-ethylmaleimide, the stimulatory effect of 1,25-D_3_ on calcium influx increased, appearing as the 1,25-D_3_ mechanism does not depends on this pathway. The stimulatory effect of 1,25-D_3_ was decreased by apamine, thapsigargin, dantrolene, 2-APB and was totally nullified by nifedipine. When PKA and PKC inhibitor were used, the effect of compound was abolished, as well as TEA, a voltage-dependent K^+^ channel inhibitor.

**Conclusion:** The stimulatory effect of 1,25-D_3_ for insulin secretion involves the activation of K^+^-ATP, Ca^2+^‐ATP channels that contribute to depolarization. These ionic changes also lead to the downstream participants activation. After intracellular abrupt calcium augment and insulin secretion, the activation of voltage-dependent K^+^ channels may be crucial for cell repolarization. Therefore, these channels can also be a therapeutic target to stimulate insulin secretion, together with the role of 1,25-D_3_ as an oral hypoglycemic agent. The development of new drugs capable of acting in these channels would contribute to the use of 1,25-D_3_ on insulin secretion.

**Financial support:** CNPq; CAPES; PPG-Farmácia; LAMEB/CCB; PROAP/UFSC.

### P234 Rates of major adverse cardiovascular (cv) events (mace) and mortality with basal insulin by liraglutide use: a devote subanalysis

#### Paulo Roberto Rizzo Genestreti^1^, Scott S. Emerson^2^, Steven P. Marso^3^, Darren K. McGuire^4^, Thomas R. Pieber^5^, Neil R. Poulter^6^, Bernard Zinman^7^, Richard E. Pratley^8^

##### ^1^Instituto do Coração-Hospital das Clínicas -Universidade de São Paulo, São Paulo, Brazil; ^2^University of Washington-USA; ^3^HCA Midwest, Kansas City-USA; ^4^University of Texas Southwestern Medical Center, Houston, USA; ^5^Medical University of Graz-Austria; ^6^Imperial College, London, UK; ^7^Mount Sinai Hospital, University of Toronto, Toronto, Canada; ^8^Florida Hospital, Orlando, USA

*Diabetology & Metabolic Syndrome* 2019, **11(Suppl 1):**P234

**Introduction:** CV safety profiles for insulin degludec (degludec) and insulin glargine 100 units/mL (glargine U100) were established by the DEVOTE and ORIGIN trials. In the LEADER trial, the GLP-1 analog liraglutide significantly reduced risks of MACE and mortality vs placebo in patients with type 2 diabetes (T2D) and high CV risk.

**Methods:** This*post hoc* analysis compared effects of concomitant liraglutide vs no liraglutide use on MACE and mortality in 7637 patients with T2D and high CV risk randomized 1:1 to degludec/glargine U100 in DEVOTE (NCT01959529). Hazard ratios (HRs) for MACE/mortality were calculated using a Cox regression model adjusted for treatment and time-varying liraglutide use at any time in the trial, without interaction. Sensitivity analyses adjusted for baseline covariates including age, sex, smoking, T2D duration, CV risk, insulin therapy, A1C, LDL, HDL and liver/kidney function. At baseline, 436 (5.7%) patients were on liraglutide: 187 (2.4%) started and 210 (2.7%) stopped liraglutide thereafter. Mean liraglutide exposure from randomization was 731 days.

**Results:** Liraglutide use was associated with significantly lower HRs for MACE and mortality vs no liraglutide use. HRs from sensitivity analyses were consistent with these results.

**Conclusion:** Liraglutide was associated with significantly lower MACE and mortality rates in basal insulin users.

### P235 Reasons for food choices among adults and older adults in urban areas, with and without diabetes

#### Sara Franco Diniz Heitor, Julia Elba de Souza Ferreira, Sybelle de Souza Castro

##### Universidade Federal do Triângulo Mineiro - UFTM, Uberaba, Brazil

*Diabetology & Metabolic Syndrome* 2019, **11(Suppl 1):**P235

**Introduction:** Food choices are motivated by different factors such as economic, sociocultural, emotional, and biological, among others factors. Identifying the reasons for food choices is relevant to guide the actions of food and nutrition education.

**Objective:** To indentify the reasons for food choices among patients with and without diabetes.

**Methods:** Study conducted with 652 adults/elderly residents in the urban area of Frutal (MG), between January and July/2016. Of these, 55 (8.4%) self-reported diabetes. The instrument for general identification and the brazilian version of the Food Choice Questionnaire (FCQ) were used. The FCQ consists of 36 items distributed in nine factors: health, mood, convenience, sensory appeal, natural content, price, weight control, familiarity and ethical concern. Answers are presented on a likert scale ranging from 1 to 4 points: 1 (not important); 2 (a little important); 3 (moderately important) and 4 (very important). Descriptive statistical analysis and Student’s t-test for independent samples were performed. Project approved by the Research Ethics Committee No. 972.883/2015.

**Results:** Most diabetes patients were women (67.3%), married/with partners (69.1%), aged between 18 to 96 years old (average 46.4; DP 19.3), 1 to 5 years of schooling (32.7%), with per capita family income up to two minimum wages (49.1%) and with overweight (72.7%). *Sensory Appeal* was the most important determinant of food choices, with the highest average score, both among patients with diabetes (x̅ = 3.76, DP 0.50) and among other respondents (x̅ = 3.65, DP 0.60). In the extreme, the lowest average scores (x̅ = 1.79, DP 1.02) occurred in the factor *Ethical Concern* (p < 0.05) among those without diabetes when compared to those with diabetes. The *Health* factor, ranked fifth in food choices, had the highest score among respondents without diabetes (p < 0.05).

**Conclusion:** The food choices of patients with diabetes were not guided by the aspects that make up the *Health* factor, but by the fact that it is tasty and good looking, items present in the *Sensory Appeal* factor. This result indicates that dietary prescriptions, widely used in glycemic control of individuals with diabetes, should not be based solely on rational criteria of scientific knowledge, but also on elements of personal and social culture experience of eating.

### P236 Reduction in hba1c using flash glucose monitoring in insulin-treated type 2 diabetes patients in a secondary care setting

#### Maria de Lourdes Machado^1^, Naira Horta Melo^1^, Mariana Garcez Varela^1^, Kamila Silva Oliveira^2^, Bárbara Gabriela Gomes Silva^1^, Lysandro Borges Pinto^3^, Carla Raquel Pereira Oliveira^3^, Karla Freire Rezende^1^

##### ^1^Universidade Federal de Sergipe e IPESAUDE, Aracaju, Brazil; ^2^IPESAUDE; ^3^Universidade Federal de Sergipe, Aracaju, Brazil

*Diabetology & Metabolic Syndrome* 2019, **11(Suppl 1):**P236

**Introduction and objectives:** There are almost 425 million adults with diabetes worldwide. Despite the increasing technological evolution in treatment, most patients remain with inadequate glycemic control. Continuous glucose monitoring by an interstitial sensor, FreeStyle Libre^®^ was available in recent years, which allows patients to obtain the instantaneous glucose concentration value, the retrospective kinetic data, as well as a prospective trend of its glycemic kinetics. The aim of this study was to evaluate the impact of using this system in a group of bad controlled type 2 DM (T2DM) patients.

**Methods and results:** This is a prospective cohort study. Inclusion criteria were IPESAUDE-assisted T2DM volunteers with HbA1c greater than 8.0% and using at least basal insulin and some oral drug. Exclusion criteria were glomerular filtration rate < 15 ml per minute or difficulties in using the technology. Each participant received a continuous monitoring sensor and went through a multidisciplinary team (endocrinologist, nurse, nutritionist) where they obtained clinical and educational conducts based on the data obtained by the glucose sensor. Patients were reevaluated every 2 weeks and followed for 12 weeks. At the first and the last meeting, biochemical tests and anthropometric measurements were performed. The study population consisted of 22 volunteers and 18 completed the program. Most were female (68.2%), with a mean age of 61.77 ± 9.21 years. All patients used basal insulin, and 82.8% used prandial insulin bolus. Regarding the time of illness, 72.7% had more than 10 years of diagnosis. The mean HbA1c at baseline was 9.6% ± 1.35 and at the end of the study, we achieved an average HbA1c of 8.1% ± 0.7, an average reduction of 1.5% (p < 0.001). At this time, 76.5% of participants had adequate glycemic control for their chronological age. Despite intensification of treatment, there was a slight reduction in BMI (0.27 kg/m^2^ - p = 0.62) and a reduction in total insulin dose (reduction of 11.5 units—p < 0.001), with 9.9% patients left insulin therapy. There were no episodes of severe hypoglycemia.

**Conclusion:** This protocol using FreeStyle Libre^®^ proved to be effective in improvement of HbA1c of bad controlled T2DM patients, without weight gain, with reduction of basal insulin dose and without occurrence of severe hypoglycemia. However, additional studies should be performed to analyze the cost effectiveness of introducing this device into the clinical practice.

### P237 Relationship between diabetic retinopathy and cardiovascular disease in patients with type 1 diabetes

#### Laura Gomes Nunes De Melo^1^, Bianca Senger Vasconcelos Barros^1^, Deborah Conte Santos^1^, Marcela Haas Pizarro^1^, Luiza Harcar Muniz^1^, Dayse Aparecida Silva^2^, LuisCristovao Porto^3^, Marilia Brito Gomes^1^

##### ^1^Universidade do Estado do Rio de Janeiro, Departamento de Medicina Interna, Diabetes, Rio de Janeiro, Brazil; ^2^Universidade do Estado do Rio de Janeiro, Laboratorio de Diagnostico DNA, Rio de Janeiro, Brazil; ^3^Universidade do Estado do Rio de Janeiro, Laboratorio de Histocompatibilidade e Criopreservação, Rio de Janeiro, Brazil

*Diabetology & Metabolic Syndrome* 2019, **11(Suppl 1):**P237

**Introduction:** Cardiovascular disease, the leading cause of death worldwide, and diabetic retinopathy, the main cause of blindness in economically active populations, share clinical risk factors and pathophysiological features.

**Objective:** The aim of this study is to examine the association between diabetic retinopathy, cardiovascular disease, and common risk factors in patients with type 1 diabetes.

**Methods:** This nested case–control study was performed in patients from the Brazilian Type 1 Diabetes Study Group, a nationwide survey that was conducted in Brazil that enrolled 1760 patients with type 1 diabetes. A total of 342 patients were selected (57 cases with macrovascular disease and 285 controls who were matched for duration of diabetes and gender). The study protocol was approved by the coordinating center and the ethics committee of each participating center.

**Results:** In theexploratory analysis, stratified by cardiovascular disease, the following variables were statistically significant: age (p = 0.037), hypertension (p = 0.035), high BMI (p = 0.046), diabetic retinopathy (p = 0.003), and chronic kidney disease (p = 0.026). In multivariate logistic regression, patients with diabetic retinopathy were more likely to develop cardiovascular disease (OR 2.16, 95% CI 1.16–4.02, p = 0.015). Although to a lesser extent than diabetic retinopathy, higher BMI levels were also related to an increase in the risk of cardiovascular disease of 1.08 (95% CI 1.01–1.15, p = 0.024).

**Conclusion:** The presence of diabetic retinopathy indicates a greater risk for cardiovascular disease in Brazilian patients with type 1 diabetes. Further studies are warranted to determine whether a noninvasive exam, such as fundoscopy, could help identify patients who show an increased risk for cardiovascular disease.

**Financial support:** This work was supported by the FAPERJ (grant number 1989.0246.5) and CNPq (grant number 563753/2010-2).

### P238 Relationship between knowledge and the risk degree of developing diabetic foot

#### Vicente Rubens Reges Brito, Valdenia Maria de Sousa, Francisco João de Carvalho Neto, Lucas Sallatiel Alencar Lacerda, Antônia Fabiana Rodrigues da Silva, Samila Lacerda Pires, Kaline Elisa dos Santos, Ana Roberta Vilarouca da Silva

##### Universidade Federal do Piauí, Terezina, Brazil

*Diabetology & Metabolic Syndrome* 2019, **11(Suppl 1):**P238

Introduction and Objectives: Knowledge is essential for individuals to have control over their health conditions and risk control. Thus, this study aimed to analyze the knowledge about the preventive measures for the development of the diabetic foot. Methods and results: Analytical study and cross-sectional with qualitative approach, performed with 171 diabetic patients accompanied by the Family Health Strategy of the city of Picos-Piauí. Data collection was performed at the Basic Health Units and at home, from September to November 2018, through 03 forms, already prepared and available for use, which comprise: demographic data, social and epidemiological diagnosis, clinical examination of the feet and knowledge of essential feet care. Data were collected through interview where the forms were applied, after physical examination of the feet. The study was approved by the Research Ethics Committee of the Federal University of Piauí, according opinion nº 2.389.111. The results indicated the predominance of female (62.6%), had between 60 and 69 years of age (29.8%), with 1 to 5 years of study (51.4%), monthly income between 1 and 2 minimum wages (70.2%), economy class D-E (61.3%), married (55.5%), retired people/pensioner (59.6%), brown (48%). In the sample selection approximately 25.73% present risk degree 0, 49.71% presents risk degree 1 and 24.56% risk level 2, according to Diabetic Foot Manual (2016), in relation to degree knowledge to notice that 35 (20.5%) of the interviewed patients had no or little knowledge, 143 (78.4%) have good knowledge and 2 (1.2%) very good knowledge about essential feet care. About knowledge, predominated none or very little knowledge of people at risk 2 for diabetic foot development 17 (40.5%). Among the good knowledge, there was a higher number among Risk 1, 76 (89.4%). And very good knowledge prevailed risk 0 and 2, degree 0 (2.3%) and degree 2 (2.4%), respectively. Conclusion: Thus, conclude that most participants have a good level of knowledge and low risk of developing diabetic foot, this way, the study is of fundamental importance, positively contributing to the production of knowledge that can be used in the healthcare practice of health professionals.

**Keywords:** Diabetes Mellitus; Diabetic foot; Complications of diabetes; Risk factors.

### P239 Relationship between quality of life and referred pain to diabetic polineuropathy and stiff hand syndrome in diabetic patients

#### Taynara Guedes da Silva^1^, Débora Fidélis de Oliveira^2^, Francisca Janiele Ribeiro Tavares^2^, Pedro Miguel Afonso de Almeida e Silva^2^, Wescley Franco Lima^2^, Natália Virgínia da Silva Castro^2^, José Carlos Tatmatsu Rocha^2^, Daniela GardanoBucharles Mont’Alverne^2^

##### ^1^Hospital Universitário Walter Cantídio - Universidade Federal do Ceará, Fortaleza, Brazil; ^2^Universidade Federal do Ceará, Fortaleza, Brazil

*Diabetology & Metabolic Syndrome* 2019, **11(Suppl 1):**P239

**Introduction:** Diabetes Mellitus (DM) can lead to musculoskeletal complications and pain that can interfere with the functionality and quality of life of individuals.

**Objective:** To investigate the relationship between quality of life and referred pain with diabetic distal polyneuropathy and stiff hand syndrome in diabetic patients.

**Methods:** A cross-sectional study and quantitative approach was performed in DM-independent patients over 18 years of age. All participants were assessed for demographic (age, gender), anthropometric, clinical (time since diagnosis, last fasting blood glucose, last HbA1c values), quality of life (Diabetes Quality Measurement Questionnaire, DQOL—Brazil), referred lower limb pain (analogue pain scale) and Diabetic distal polyneuropathy diagnosis scale where the neuropathic symptoms score (NSS) and the neuropathic impairment score (NIS) were evaluated. The presence of the stiff hand syndrome was obtained by the Prayer Sign test. Results were expressed as mean ± standard deviation and/or percentage. For associations, t-test was performed for independent samples, and, for correlations, Pearson’s test was performed, considering statistically significant p ≤ 0.05.

**Results:** 35 patients participated in the study, 91.4% (n = 32) with type 2 DM, with a mean age of 61 ± 12 years and predominance for males (n = 19; 54.3%). They had a mean years of diagnosis of 15.7 ± 8.7 years, with a mean last glycemia of 186.4 ± 85 mg/dL and a mean glycated hemoglobin (HbA1c) of 8.2 ± 1.7%. A median quality of life was observed (mean total score: 105.85 ± 26.89), with the level of overall satisfaction being the worst assessed domain (40.2 ± 10.5). 65.7% (n = 23) presented positive Prayer sign. The mean pain reported was 5.6 ± 3.3. When correlating the Prayer Sign with the other variables, no association was observed. There was a positive correlation between NSS with reported pain (r = . 736 p = 0.0001), years of illness with quality of life (r = . 492 p = 0.003) and years of illness with HbA1c (r = . 600 p = 0.039).

**Conclusions:** There was no relationship between stiff hand syndrome and quality of life, however, it was observed that the higher the pain, the higher the scores on the NSS, as well as the more years with the disease, the worse the quality of life of diabetic patients.

### P240 Relationship between sleep disturbances and metabolic profile in type 1 diabetic patients

#### Giovanna Aparecida Balarini Lima, Rubens Antunes da Cruz Filho, Ana Carolina Musser Tavares de Mattos, Bruna Landeiro Tavares, Yuri Sofiati Campos, Yasmin Sab, Vitória Oliveira Fiorini

##### Universidade Federal Fluminense, Rio de Janeiro, Brazil

*Diabetology & Metabolic Syndrome* 2019, **11(Suppl 1):**P240

**Introduction:** Sleep disturbances are poorly evaluated in type 1 diabetic patients (T1D).

**Objectives:** To analyse if sleep deprivation is associated with glycemic control and its relation with the diagnose of metabolic syndrome (MS) in this population.

**Methods:** Cross-sectional study with patients from the Endocrinology Outpatient Clinic of a Public University Hospital. Inclusion criteria were age ≥ 18 years and body mass index (BMI) 20 to 25 kg/m^2^. We evaluated the presence of MS (by the International diabetes federation), glycated hemoglobin (HbA1c,  %) and lipids (mg/dl) levels. The patients answered the Pittsburgh Sleep Quality Index (PSQI) and wore a wrist actimetry for 7 days, which informed the total sleep time (TST) during a 7-day period. We considered sleep deprivation a mean TST ≤ 390 min/day. The study was approved by the Ethics Comitee (n^o^ 1.623.289).

**Results:** 33 patients with mean age 30.1 ± 10.2y-o, BMI 23.6 ± 2.2 kg/m^2^ and HbA1c 8.3 ± 1.6%. Two (6%) patients had MS, both women. Eighteen (54%) patients had a positive PSQI, demonstrating bad sleep quality. Sleep deprived patients had a bad sleep quality in PSQI during the weekdays comparing with those with a normal PSQI (70% vs. 30% respectively, p = 0.04) and a higher chance of MS diagnosis (p = 0.03). In the sleep deprived group, patients with HbA1c ≥ 9% slept less minutes than those with HbA1c between 7 and 9% (301.7 ± 28.1 v. 346.0 ± 35.1 min, respectively, p = 0.03).

**Conclusion:** T1D adult patients sleep the same amount of minutes/day in the 5 weekdays and 7-day period. In contrast to the literature, we found higher frequency of patients with bad quality of sleep by PSQI and a high prevalence of sleep deprivation. Two sleep deprived patients had MS, suggesting a relationship between sleep deprivation and worse metabolic profile. Sleep deprived T1D individuals during the weekdays with HbA1c > 9% slept less than those with a HbA1c between 7 and 9%, suggesting that lower TST is associated with worse glycemic control and a higher frequency of MS.

**Financial support:** CAPES.

### P241 Relationship between the percentage reduction of lower limb skeletal muscle mass by bioimpedance and the degree of glucose intolerance

#### Severino de Almeida Farias^1^, Ana Carolina Turrioni Azevedo Pacheco^2^, Diana Viegas Martins^1^, Larissa Antunes Magina^3^, Lorena Rejane Maia de Jesus^4^, Raquel Viegas Martins Farias^2^

##### ^1^Cliendi Prev Clinica de Endocrinologia e Prevençao de Doenças LTDA, Salvador, Brazil; ^2^Faculdade de Tecnologia e Ciencias, Salvador, Brazil; ^3^Centro Universitario Serra dos Orgãos, Terezópolis, Brazil; ^4^Universidade Federal da Bahia, Salvador, Brazil

*Diabetology & Metabolic Syndrome* 2019, **11(Suppl 1):**P241

**Introduction:** In Type 2 Diabetes Mellitus (DM), the glucose-insulin interaction becomes compromised when this hormone does not promote muscle glucose uptake or suppresses its production by the liver. In order to understand the role that muscle glycogen synthesis plays, we present an observational clinical study to evaluate the correlation between the percentage reduction of lower limb skeletal muscle mass (MME) through the bioimpedance test and the presence of DM2 and Pre -Diabetes (Pre-DM).

**Methods:** Bioimpedances were performed in 210 patients followed in a private clinic, using the InBody120^®^ equipment, from July 13 to November 13, 2018. The records were retrospectively analyzed. Patients with DM1, under 20 years old and with deformities in lower limbs were excluded. The results from the lower limb (< 90%), normal (90 to 110%) and above (> 110%) MME bioimpedance results according to the equipment specifications. Microsoft Excel 2016^®^ software was used for data analysis and calculation of the MME arithmetic mean of each group.

**Results:** The mean of lower limb MME obtained in patients with T2DM was 88.97 ± 6.57%, in Pre-DM 88.98 ± 8.76% and in patients with normal glucose tolerance 89.61 ± 7.92. %. Among patients aged 20 to 30 years, 12.5% were pre-DM and 87.5% had normal glucose tolerance values. Among pre-DM, 100% had lower limb MME within normal range, while in those with normal glucose tolerance, 35.71% had low MME and 64.29% normal. Among those between 30 and 60 years old, 14.29% were diabetic, 16.96% pre-DM and 68.75% had normal glucose tolerance. Among diabetics, 37.5% had lower than average and lower limb MME, and 62.5% were normal, while among pre-DM, 42.11% were below, 52.63% normal and 5.26% above. In those with normal glucose tolerance, 42.86% had low MME, 55.84% normal and 1.30% above. Among patients over 60 years old, 40.24% were diabetic, 31.71% pre-DM and 28.05% had normal glucose tolerance. Among diabetics, 60.61% had low MME and 39.39% normal. Among pre-DM, 76.92% had low MME and 23.08% normal, while among those with normal glucose tolerance, 73.91% had low MME and 26.09% normal.

**Conclusion:** The percentage reduction in the lower limb MME was directly related to the degree of glucose intolerance. In turn, this relationship was more evident in patients older than 60 years.

### P242 Relative muscle strength suppression in diabetic patients with cardiovascular disease

#### Luciana de Brito Gonçalves Nascimento^1^, Viviane Sahade^2^, Bernadete Weber^3^, Carla Hilário da Cunha Daltro^1^

##### ^1^Programa de Pós Graduação em Medicina e Saúde (PPgMS)/Universidade Federal da Bahia (UFBA), Salvador, Brazil; ^2^Escola de Nutrição/UFBA, Salvador, Brazil; ^3^Hospital do Coração (HCOR), Salvador, Brazil

*Diabetology & Metabolic Syndrome* 2019, **11(Suppl 1):**P242

**Introduction:** Diabetes Mellitus (DM) is associated to muscle tone strength. Relative muscle strength adjusted by body mass index (RMS/BMI) is a low-cost method of easy applicability that has been used in the following up of muscle strength.

**Objective:** Evaluate the factors that are associated to the reduction of RMS/BMI in patients with DM2.

**Methods:** cross-sectionalstudyin individuals with cardiovascular disease (CVD), aged **> **45 years old, attended between 2013 and 2015 in a Nutrition ambulatory. There were measuring of hand grip strength tests (HGS), with Jamar^®^ dynamometer (dominant hand) and the relation RMS/BMI, according to the Foundation *for the National Institutes of Health Sarcopenia Project*. The risk of sarcopenia was defined by criteria from the *European Working Group on Sarcopenia in Older People*. There were evaluated sociodemographic data that refer to lifestyle, body composition and food intake, from a 24-h food recall by Nutriquanti^®^. Data were shown through simple and relative attendance, average and standard deviation or median (Md) and interquartile range (IQR). The groups were compared by using the following tests: t Student, Mann–Whitney and Chi square—Pearson.

**Results:** We studied 97 individuals with average (SD) age of 61.4 (9.0) years old, mostly elderlies (60.8%), women (54.6%), sedentary (61.9%), overweight or obese (52.6%). Sampling was stratified according to the presence (47.4%) or absence (52.6%) of DM. There were no differences among the groups regarding age [63.1 (8.3) × 59.8 (9.4); p = 0.75), physical activity (39.1% × 37.3%; p = 0.849), muscle mass (45.7% × 54.3%, p = 0.695), excess of adipose body tissue (22.5% × 22.2%; p = 0.976), IMC [29.7 (5.4) × 27.9 (4.7); p = 0.86] and risk of sarcopenia (21.7% vs 9.8%; p = 0.105). Diabetic patients showed a higher depletion of HGS/BMI [Md: 0.8 (IIQ: 0.6–1.2) vs Md: 1.1 (IIQ: 0.7–1.4); p = 0.034], lower relation non-protein calorie/g nitrogen [Md:104.1 (IIQ: 79.7–138.2) vs Md:123.0 (IIQ: 97.2–147.5); p = 0.039], when compared to non-diabetic patients.

**Conclusion:** Diabetic patients with VCD showed higher decline of relative muscle strength, lower hand grip strength, lower relation non-protein calorie/g nitrogen and higher risk of sarcopenia, although this last difference hasn’t reached significant statistics (Study supported by PROADI-MS).

### P243 Repercussion of emotional aspects in self-care practices in diabetes mellitus type 2

#### Laura Barbosa Nunes, Jéssica Caroline dos Santos, Heloísa de Carvalho Torres

##### Universidade Federal de Minas Gerais, Belo Horizonte, Brazil

*Diabetology & Metabolic Syndrome* 2019, **11(Suppl 1):**P243

**Introduction**: Approximately 425 million people worldwide have diabetes mellitus (DM), however, about half of them disregard the diagnosis. Interventions to promote self-care practices of people with chronic conditions should understand emotional aspects through the analysis of feelings.

**Objective**: To identify the repercussion of emotions of people with type 2 diabetes mellitus in self-care practices.

**Methods**: This is a qualitative and descriptive exploratory study conducted with 10 users participating in the behavioral program and linked to 2 basic health units (BHU) in Belo Horizonte, Minas Gerais, 2019. Data collection was performed using the group technique focus and sociodemographic characteristics through face-to-face interviews. The focus group technique was guided by a script that addresses the question “what did you feel when you found out you had diabetes? Did you manage to associate it with any feelings?”, This script allowed users to be integrated, besides generating the creation of a bond through the recognition of emotions. The meetings were recorded, recorded, transcribed, systematized and categorized to compose a database. Data were interpreted based on the thematic analysis adapted by Bardin, in three stages: pre-analysis, pre-analytical categories and the treatment of information with inferences and interpretation. All research ethics standards were met in accordance with National Health Council Resolution 466/12.

**Results**: Participants were female, aged 45 to 62 years, average diabetes diagnosis time around 10 years, and the level of education was up to literacy class. The categories identified through content analysis showed the repercussion of emotions on self-care practices such as: Sadness (60%), joy (20%) and fear (20%). Negative emotions such as may be associated with depression and anxiety. It is understood that the emotions stimulated by the chronic condition are related to the users’ behaviors, which can interfere with the acceptance of the diagnosis, hindering self-care practices.

**Conclusions:** Reduce the emotional impact on coping with behavioral changes for diabetes self-care practices. Financial Support: CNPq 432824/2016; FAPEMIG APQ-03865-16.

### P244 Risk foods and protection for dislipemide and related factors in adolescents from public schools in Recife-PE

#### Pedrita Mirella Albuquerque Queiroz^1^, Ilma Kruze Grande De Arruda^2^, Iii.Alcides Da Silva Diniz^2^, Ísis Lucília Santos Borges De Araújo^3^

##### ^1^Faculdade de Comunicação, Tecnologia e Turismo de Olinda-PE, Olinda, Brazil; ^2^Universidade Federal de Pernambuco, Recife, Brazil; ^3^Plataforma Enfrente, Recife, Brazil

*Diabetology & Metabolic Syndrome* 2019, **11(Suppl 1):**P244

**Introduction:** The prevalence of dyslipidemia in adolescents has been occurring concomitantly with the advent of obesity. Studies have been conducted to evaluate the factors associated with dyslipidemia.

**Objective:** To evaluate the consumption of risk foods and the protectors for the development of dyslipidemia and its association with some factors.

**Methods:** Cross-sectional study with 411 adolescents recruited from public schools in Recife, Brazil, between March/April 2013. Sociodemographic, anthropometric, behavioral and biochemical variables were evaluated. The data were described as median and their respective interquartile ranges. Man–Whitney and Kruskal-Wallis tests were used to buy groups. Statistical analysis was performed by Epi-info 6.04 and SPSS 13.0.

**Results:** The median monthly risk food consumption score (Group I) was higher (0.13; IQ = 0.08–0.27) than the protectors (Group II) (0.00; IQ = 0 0.00 to 0.08) (p = 0.001). The Group I score median tended to be higher for normal WC compared to increased WC (p = 0.055). There was a higher trend in the Group II median for assets than under-active ones (p = 0.056). The medians of Group II were higher for those who exercised in the last year than those who did not (p = 0.019). The dyslipidemics had group I score medians higher than those without (p = 0.005). There was a positive correlation between the hours of sedentary activities and the group I median (p = 0.013), and the weekly physical activity time with the group II median (p = 0.047).

**Conclusion:** The results showed a high frequency of risky food consumption, associated with maternal education, dyslipidemia and behavioral variables, which point to the need to adopt measures to control the excessive consumption of this group of foods.

**Keywords:** Food consumption; Adolescent; Dyslipidemia; Anthropometry; Lifestyle

### P245 Risk profile for high glycemia in Brazil: results from blue diabetes November campaign 2018

#### Wendel Coura Vital^1^, Eliete Bachrany Pinheiro^2^, Mônica Soares Amaral Lenzi^2^, Wesley Magno Ferreira^2^, Janice Sepulveda Reis^3^, Karla Melo^3^, José Vanilton de Almeida^2^, Hermelinda Cordeiro Pedrosa^3^

##### ^1^Universidade Federal de Ouro Preto, Ouro Preto, Brazil; ^2^Conselho Federal de Farmácia, Brasília, Brazil; ^3^Sociedade Brasileira de Diabetes, São Paulo, Brazil

*Diabetology & Metabolic Syndrome* 2019, **11(Suppl 1):**P245

**Introduction**: It is estimated that more than 41 million adults, over 20 years of age, have diabetes mellitus (DM) in Latin America and the Caribbean. DM represents a high economic impact, with a total cost of US$102 to 123 billion, including direct costs with treatment and complications, and indirect costs, premature mortality and temporary or permanent disability. In this context, it is important to investigate the factors associated with high glycemic rates in individuals without a previous diagnosis of DM.

**Objective**: To carry out the screening of participants without previous diagnosis of DM who present high glycemia in Brazil and to investigate the risk factors associated with this condition.

**Methods:** A national cross-sectional study was conducted in November 2018, involving pharmacies from all over the country. People without previous diagnosis of diabetes, aged 20 to 79 years, were invited to participate in the study. Capillary blood glucose was assessed and a validated questionnaire (Findrisc) was applied to the participants of the study. Glycemia was considered high when fasting glycemia ≥ 100 mg/dL or when casual glycemia ≥ 140 mg/dL. Risk factors associated with high glycemia were investigated using the Poisson model with robust variance.

**Results:** A total of 977 community pharmacists from 345 cities participated in the study and 17,580 people were evaluated. The majority of encounters (87.8%) occurred in consulting rooms within retail pharmacies. The population consisted mainly of women (59.5%) and people aged < 45 years (47.9%). The Brazilian prevalence of patients with high glycemia was 18.4% (95% CI: 17.9–19.0). The risk factors associated with high glycemia were: increased body mass index, increased abdominal circumference, lower academic level, lack of fruits or vegetables on daily diet, hypertension, history of high glycemia and having parents, siblings or children with type 1 or type 2 diabetes. The most frequent risk factors were: increased abdominal circumference (present in 78.8% of women and 59% of men), lack of vegetables and/or fruits on daily diet (43%) and history of the disease in parents, siblings or children (37%).

**Conclusions:** This study may contribute to a better understanding of factors associated with elevated glycemic levels in Brazil. Aware of these factors, health professionals can give greater attention to certain patient profiles, contributing to an early diagnosis of DM.

### P246 Roles of aging and menopause-induced lipid subfraction changes for cardiovascular risk in women

#### Marilia Fonseca^1^, Bianca De Almeida-Pititto^2^, Isabela M Bensenor^3^, Peter P Toth^4^, Steven R Jones^4^, Michael J Blaha^4^, Paulo A Lotufo^3^, Sandra R G Ferreira^1^

##### ^1^Department of Epidemiology, School of Public Health, University of São Paulo, São Paulo, Brazil; ^2^Department of Preventive Medicine, Federal University of São Paulo, São Paulo, Brazil; ^3^Internal Medicine Department, University of São Paulo, São Paulo, Brazil; ^4^Johns Hopkins Ciccarone Center for the Prevention of Heart Disease, Baltimore, USA

*Diabetology & Metabolic Syndrome* 2019, **11(Suppl 1):**P246

Roles of aging and menopause-induced lipid subfraction changes for cardiovascular risk in women from the ELSA-Brasil.

**Introduction:** It is unclear how aging and menopause-induced lipid changes contribute to the elevated cardiovascular risk in menopausal women. We examined the association between status and duration of menopause with lipid profile in the Longitudinal Study of Adult Health (ELSA-Brasil).

**Methods:** This is a cross-sectional analysis of baseline data of women from the Sao Paulo centre of ELSA-Brasil, stratified by status and duration of menopause into 5 groups: pre-menopause, < 2 years, 2–5.9 years, 6–9.9 years and ≥ 10 years of menopause, excluding menopause < 40 years or non-natural cause, use of lipid-lowering drugs or hormone replacement therapy. Comparisons were performed using ANOVA with Bonferroni correction. Associations of menopause categories and time since menopause with cardiometabolic and vertical auto-profile-determined lipid variables were tested using multiple linear regression, considering premenopausal women as reference. Adjustments for age, ethnicity, education level, central obesity, diabetes, hypertension, smoking, alcohol intake and physical activity were performed.

**Results:** From 1,916 women (49.6 years ± 8.5 years), postmenopausal groups were older and had a worse cardiometabolic risk profile than the premenopausal one. Unadjusted higher total cholesterol, LDL-c, real LDL-c, IDL-c, VLDL-c, triglycerides, non-HDL-c, VLDL_3_-c, triglyceride-rich lipoprotein remnants (TRL-c) and buoyant LDL-c concentrations differed from pre-menopausal women, but no difference among menopausal groups was observed. In multiple linear regression, duration of menopause < 2 years was significantly associated with TRL-c [7.21 mg/dL (95% CI 3.59–10.84)] and VLDL_3_-c [2.43 mg/dL (95%CI 1.02–3.83)]. No association of menopausal categories with HDL-c or LDL-c subfractions were found, nor associations of time since menopause with lipid subfractions, either.

**Conclusions:** A strong effect of menopause on triglyceride-rich lipoprotein remnants seems to occur right after its onset, which tends to plateau after 2 years. More studies are needed to evaluate the role of TRL-c on cardiovascular risk in postmenopausal women.

**Keywords:** Menopause; Cardiovascular risk; Lipoprotein subfractions; Triglyceride-rich lipoprotein remnants.

### P247 Screening of gck-mody in diabetes diagnosed during the pregnancy

#### Juliana Cristina Cardoso Ferreira, PatriciaMedici Dualib, Giovanna Campos Paranhos Abrahao, Felipe Crispim, Andre Fernandes Reis, Regina Celia Mello Santiago Moises

##### Universidade Federal de São Paulo, São Paulo, Brazil

*Diabetology & Metabolic Syndrome* 2019, **11(Suppl 1):**P247

**Introduction:** Mutations in the gene encoding the enzyme glucokinase are frequent causes of MODY (MODY-GCK). Affected individuals have a nonprogressive mild fasting hyperglycemia (100–150 mg/dL) phenotype and are often asymptomatic. As all pregnant women are routinely evaluated for hyperglycemia, prenatal care may be an opportunity to identify MODY-GCK.

**Objective:** To determine the frequency of MODY-GCK among women with diabetes mellitus diagnosed during pregnancy. Identification of clinical criteria indicative of this form of DM in a population with a high degree of miscegenation, such as the Brazilian population.

**Methods:** Patients with the following inclusion criteria were selected: pregnant women with fasting glucose ≥ 92 mg/dl and BMI < 30 kg/m^2^ before pregnancy; or, non-obese patients diagnosed with DM during pregnancy and persistent hyperglycemia after pregnancy (fasting glucose ≥ 100 mg/dl). Direct sequencing of the entire codon region and exon–intron of the GCK gene was performed.

**Results:** Fifty patients were evaluated, with a mean age of 33.16 ± 5.27 years and pre-pregnancy BMI of 24.86 ± 2.57 kg/m^2^. Four heterozygous mutations in the GCK gene were identified in 4 patients (T255S, P59S, G162S, D124 N). Diabetes was diagnosed in the first trimester of pregnancy in the 4 MODY-GCK patients; and 56% (26/46) of the patients in whom the mutation was not identified. Family history for diabetes in first-degree relatives was identified in all MODY-GCK patients (in three MODY-GCK patients and unknown in one of these) and in 47% (22/46) of patients without mutation.

**Conclusion:** Our findings indicate a significant proportion of MODY-GCK among DM patients diagnosed during pregnancy. Clinical screening criteria for GCK variant may be considered.

### P248 Self-care among individuals with type 1 diabetes using continuous insulin infusion system

#### Eliziane Brandão Leite^2^, Luciana Lopes Ribeiro^1^, DanyelleLorrane Carneiro Veloso^3^, LuisianeDe Ávila Santana^3^, Angélica Amorim Amato^3^, Virginia Carvalho Martins Brandão Leite^4^

##### ^1^Escola Superior de Ciências da Saúde, Brasília, Brazil; ^2^Centro Especializado em Diabetes, Obesidade e Hipertensão da Secretaria de Estado de Saúde do DFi, Brasília, Brazil; ^3^Universidade de Brasília, Brasília, Brazil; ^4^Universidade de Ciências do Planalto Central, Brasília, Brazil

*Diabetology & Metabolic Syndrome* 2019, **11(Suppl 1):**P248

**Introduction:** The therapeutic success for a type 1 diabetes mellitus (DM1) adult in use of the Continuous Insulin Infusion System (SIC) depends heavily on their lifestyle and willingness to self-care.

**Objective:** To evaluate self-care practices of patients with DM1 under treatment with the Continuous Insulin Infusion System (SIC) at the Federal District Health Department (SES/DF).

**Methods:** This was a descriptive cross-sectional study in which individuals with DM1 under SIC treatment from the SES/DF Insulin Therapy Program were evaluated from September 2017 to February 2018. Data collection was performed using the “Questionnaire of Diabetes Self-Care Activities”, adapted to the Portuguese language. This study was approved by the Research Ethics Committee of the Teaching and Research Foundation of the Federal District through Opinion No. 2,231,419.

**Results:** The sample consisted of 21 eutrophic individuals with ages ranging from 18 to 79 years, with female predominance. Just over half of the people surveyed have HbA1c rates within the target. Diet related self-care, glycemic self-monitoring, and medication use were close to desirable for all items on average days. For the items related to physical activity or exercise, the averages were 3.3 and 2.7 respectively. Regarding foot care practices, the average days of the week ranged from 3.9 to 5.4 among hygiene, self-examination and shoe care activities. Only 1 individual, 5% of the sample reported being a smoker.

**Conclusions:** The majority of the sample reported playing their role in self-care to maintain disease control. Most individuals report adherence to treatment, maintaining a consistent exercise routine, healthy diet, adequate insulin therapy, glycemic self-monitoring and glycated hemoglobin within the target. This study received financial support from the Health Sciences Teaching and Research Foundation (FEPECS).

### P249 self-care assessment of patients with type 1 diabetes mellitus in a university hospital

#### Teodoro Dias De Oliveira Ferreira, Dandara Sampaio Leão De Carvalho, Isabella Carvalho Oliveira, Gabriela Ferreira Fernandes Ribeiro, Lucas Wilson Matos Gomes, Larissa Dias Rodrigues, DálletyZany Da Silva Gomes, Daniela Espindola Anteus

##### Universidade Federal de Goiás, Goiânia, Brazil

*Diabetology & Metabolic Syndrome* 2019, **11(Suppl 1):**P249

**Introduction:** The treatment for Type 1 Diabetes Mellitus (T1DM) is understood as a pharmacological and non-pharmacological therapy, with the necessity of an active process of self-care that comes from the patient. The adherence to medication, a healthy and individualized diet, regular routine of exercises, daily glycemic monitoring and foot-care are the pillars for a therapeutic success, being indispensable for the patients’ survival and also for a reduction in the complications.

**Objective:** To describe the access to recommendations of self-care in T1DM individuals being treated in an university hospital.

**Methods:** A cross-sectional observational study that used a non-probalistic population sample constituted by 52 individuals that have T1DM accompanied by The Endocrinology department of an university hospital. The data collected in the first semester of 2019, with the application of the instrument and validated to the Brazilian reality, *Summary of Diabetes Self*-*Care Activities Questionnaire* (SDSCA), instrument of the World Health Organization for the evaluation of self-care. The variables used were gender, age, adhesion to a nutritional recommendation and physical activity, glycemic monitoring medication and foot-care.

**Results:** From the 52 patients studied, 67.3% were women. The average age was 22.3 years old (DP ± 10.8). 32,6% referred to follow diet guidelines for more than 5 days in the week and 40.3% referred to practice exercises for at least 150 min per week. Additionally, 90.5% of the patients referred adhesion to the pharmacological therapy according to the prescription for more than 5 days specifically the week before and 55.7% made the fingerstick glucoses with the oriented frequency. Related to foot-care, only 30.7% checked their feet more than 5 times a week; 38.4% checked inside the shoes before wearing them and 55.7% dried the spaces between the toes after washing their feet.

**Conclusions:** In this recent study it was verified a good adhesion to pharmacological treatment in contrast with a low adhesion to a non-pharmacological treatment, mainly when it refers to diet recommendations and foot-care. The results show the necessity of a focus in self-care for better therapy optimization.

### P250 Self-perception of sodium consumption and food processed in employees of a higher education institution

#### Pedrita Mirella Albuquerque Queiroz^1^, Laura Nayara de Medeiros^2^, SheromReevy dos Santos Lima^2^, Maricelly Priscilla de Andrade Nascimento^2^, Ísis Lucília Santos Borges de Araújo^3^

##### ^1^Faculdade de Comunicação, Tecnologia e Turismo de Olinda-PE, Olinda, Brazil; ^2^Centro Universitário Estácio do Recife; ^3^Plataforma Enfrente, Recife, Brazil

*Diabetology & Metabolic Syndrome* 2019, **11(Suppl 1):**P250

**Introduction:** High sodium consumption is linked to the increasing number of chronic diseases such as cardiovascular diseases, which are the leading causes of death in Brazil, among others. The maximum sodium intake level is 2,000 mg/day for adults, the average sodium intake in the Brazilian population is 3,190 mg/day exceeding the allowable limit. High sodium intake by the population has been associated with high food intake industrialized, related to routine changes, life, practicality and ease of consumption.

**Objective:** To evaluate the sodium intake and self-reported intake of processed foods by employees of a Higher Ensono Institution in Recife-PE.

**Methods:** The study consisted of 137 employees of a Recife educational institution. The evaluation with the employees of the educational institution was carried out through a survey that was subdivided into socioeconomic data and a food questionnaire that included questions related to eating habits that included them; how would you rate salt consumption; preference for industrialized products with lower salt content, from Vigitel (2016), specific for chronic diseases.

**Results:** In the evaluation of eating habits, it was observed that within the group most classified their salt intake as adequate (54.88%), high (17.51%), very high (7.29%), low (17.51%), very low (2.18%), do not know (1.45%) of which 71.53% had no preference for foods with lower sodium content and 28.47% at the time of purchase or food consumption preferred those with the lowest sodium content. It should be noted that even individuals who rated their sodium intake as adequate do not take into account its content at the time of food consumption.

**Conclusion:** It is concluded, therefore, that of the evaluated employees the most part classifies their adequate sodium consumption, however, the great majority do not consider having sodium as relevant at the time of purchase and/or consumption, leading to the hypothesis that Individuals who rate their adequate sodium intake do not consume it adequately, leading to a risk alert for the study population because high sodium intake can be harmful to the body and can cause chronic noncommunicable diseases or other disorders. In addition, foods rich in sodium are also mostly rich in other chemical additives. The Ministry of Health has coordinated national strategies aimed at reducing consumption of sodium and processed and ultra-processed foods.

**Keywords:** Sodium; Population; Food consumption; Cheers.

### P251 Serious game for children with t1d: an educational strategy for learning about disease and self-care

#### Valéria de Cássia Sparapani^1^, Lucila Castanheira Nascimento^2^

##### ^1^Universidade Federal de Santa Catarina, Departamento de Enfermagem, Florianópolis, Brazil; ^2^Escola de Enfermagem de Ribeirão Preto da Universidade de São Paulo, Ribeirão Preto, Brazil

*Diabetology & Metabolic Syndrome* 2019, **11(Suppl 1):**P251

**Introduction:** Diabetes education interventions might impact positively the self-management of youth with Type 1 Diabetes (T1D). The knowledge about the disease and self-care may help children to better understand their condition and motivate them to behavior change and to chase optimal glycemic control. Serious games (SG), videogames developed to educate beyond entertain, have been a promising educational strategy in the health field when designed based on behavioral theories.

**Objectives:** To present a web-based SG designed for Brazilian children about T1D knowledge and self-care.

**Methods:** A theoretical framework guided the SG development, in which the user-centered design approach and health behavior theories were applied. Children (n = 21) aged 7 to 12 years from a public hospital of the State of São Paulo, Brazil participated in focus groups sessions to report their preferences, learning needs and game ideas related to T1D knowledge and self-care. The quali-quantitative usability testingwere conducted with diabetes educators, technology experts (n = 12) and children with T1D (n = 5) along the SG development. The evaluation had high validity in content validation with respect to content, presentation and educational aspects. The research team is improving identified failures of its design. The study was approved by the Institutional Review Board.

**Results:** The SG presents an immersive narrative where health professionals and a mascot guide and support the player at a multidisciplinary center. To achieve the knowledge about disease and self-care the player needs to accomplish 3 entertaining tasks (short-term goals): to make choices during a simulation day that will immediately reflect on the child’s health condition, to order foods into 4 groups, and to travel inside the body breaking foods into small pieces and take its energy to the cells using the insulin. The tasks aim to promote gradual learning and children sense of competence and autonomy. Rewards and positive feedback are provided throughout the game to motive the player. In this way, health determinants as goal setting, extrinsic e intrinsic motivation and social support assist the knowledge through meaningful learning experiences.

**Conclusions:** The SG developed presents game’s strategies based on children’s contributions and theoretical frameworks which contribute to discussion about the importance of the interactive technologies to diabetes education.

**Financial support:** FAPESP and CAPES.

### P252 Short dietary intervention with olive oil reduces fat liver content in lean, but not overweight/obese subjects

#### Belisa Brunow Ventura Biavatti, Licio Augusto Velloso, Milena Monfort-Pires

##### Universidade Estadual de Campinas, Campinas, Brazil

*Diabetology & Metabolic Syndrome* 2019, **11(Suppl 1):**P252

**Introduction:** The prevalence of obesity is growing alarmingly worldwide. This is accompanied by an increase in the prevalence of nonalcoholic fatty liver disease (NAFLD). Studies show that dietary factors are amongst the most important modifiable risk factors that determine the progression of NAFLD. However, it is not clear how changes in the pattern of fatty acids intake could affect the liver fat content.

**Objective:** To investigate the effects of the consumption of olive oil (OO, rich in monounsaturated fatty acids) for 4 weeks on liver fat content and metabolic parameters in human adults.

**Methods:** The current study is a four-week, open clinical trial. Overweight/obese and lean voluntaries were submitted to a 4-week dietary intervention with extra virgin olive oil. The percentage of fat was calculated using Fat fraction MRi images before and after the intervention period. Metabolic variables (lipid profile, fasting glucose and insulin) were assessed by standard procedures.

**Results:** The 44 voluntaries (26 women; 15 overweight/obese; 32.5 ± 4.9 years) had a mean BMI of 24.5 ± 4.9 kg/m^2^. After 4 weeks of OO intake (mean intake 650 mL), no differences in body weight or fasting glucose metabolism were observed, but HOMA-IR values were unexpectedly increased in the lean group (0.76 ± 0.26 to 1.20 ± 0.43; p = 0.043). Interestingly, reductions in liver fat (3.85 ± 3.14 to 2.57 ± 1.81%; p < 0.01), but not visceral (77.30 ± 12.80 to 78.03 ± 7.72%) or perirenal fat content (73.68 ± 6.80 to 76.28 ± 6.84%) were detected in lean, but not in overweight/obese participants (7.82 ± 4.78 to 6.68 ± 3.53%; p > 0.05). Also, metabolic benefits, such as reduction in circulating LDL cholesterol (104.96 ± 20.44 to 99.00 ± 24.28 mg/dL; p < 0.05) were detected only in the lean group.

**Discussion/conclusion:** A short dietary intervention with olive oil can reduce fat liver content in lean, but not in obese subjects. Similarly, the metabolic benefits of this oil, rich in monounsaturated fatty acids, might be greater in the first group, in which reductions in cholesterol were detected. However, it is unclear whether the increase fasting insulin and HOMA-IR in this group could act favoring metabolism.

**Financial support:** Fapesp.

### P253 Similarities and differences between adolescents and adults with type 1 diabetes on insulin pump therapy: experience from a Brazilian diabetes center of Unified Health System (SUS)

#### Monica Andrade Lima Gabbay, Vanessa Araujo Montanari, Vanessa Fujimori Galves, Priscila Fernandes Gonçalves Pecoli, William Ricardo Komatsu, Carolina Sallorenzo, Dalva Oliveira, Sergio Atala Dib

##### Universidade Federal de São Paulo, São Paulo, Brazil

*Diabetology & Metabolic Syndrome* 2019, **11(Suppl 1):**P253

**Introduction:** A unique hormonal and emotional milieu in the teenage years result in adolescents Type 1 diabetes (T1D) glycemic control a special challenge. As weight, gain and hypoglycemia are well-known potential complications of intensive insulin therapy by continuous subcutaneous insulin infusion (CSII). **Aim**: This study analyzed the differences through the CSII treatment in adolescents and adults with T1D.

**Methods:** Forty adolescents (15.6 ± 3.3 y.o, mean + sd) and 64 adults (28.6 ± 6.0 y.o.) with T1D. Hypoglycemia was defined as periods of blood glucose (BG) < 70 mg/dl. Insulin pump parameters were obtained from CareLink-Pro^®^ and Smartpix Roche^®^ reports.

**Results:** Adolescents had shorter time of disease (9.7 ± 3.6 vs 17.7 ± 7.4 yrs.; p = 0.000) and they started CSII earlier (10.9 ± 4.9 vs 20.1 ± 9.7 yrs; p = 0.000). However, they have been with higher BG levels (overall BG 201.9 ± 57.0 vs 189.0 ± 39.6 mg/dl; p = 0.039 and A1c: 9.1 ± 1.9% vs 8.3 ± 1.1%; p = 0.019). Besides, they present higher total daily insulin (0.93 ± 0.2 vs 0.73 ± 0.2 U/kg/day; p = 0.002) and a tendency of a higher insulin for BG correction (CF):49.9 ± 11.8 vs 55.8 ± 20; p = 0.07) than adult patients. Nevertheless % basal/bolus, glycemic time in range (TIR), time in hypoglycemia, glycemic coefficient of variation and insulin carbo ratio (ICR 8.2 ± 2.3 vs 9.7 ± 5.9; p = 0.2) were similar between these two groups. A1c had a negative correlation with % bolus/day, TIR and Time in hypo in both age range. Thirty and six per cent of adolescents and 55% of adults present overweight. When we compared normal-weight vs overweight patients there were difference in ICR (7.4 ± 2.2 vs 10.6 ± 5.9 g; p = 0.001) and CF (40.1 ± 12 vs 52.5 ± 14.8 mg/dl; p = 0.000).

**Conclusion:** The insulin resistance on adolescents and overweight adults with T1D potentially are related with similarities and differences between adolescents and adults with T1D on insulin pump therapy. These factors should be considered to improve treatment in these two groups of T1D, out of glycemic goals.

### P254 Sleep in pregnant women with type 1 diabetes

#### Victoria Sudario Alencar^1^, Mariana Salles Ballalai^2^, Adriana Costa Forti^3,4^, Rejane Belchior Lima Macedo^3^, Arthur Sampaio Façanha^1^, Cristina Figueiredo Sampaio^1,3,4^, Veralice Meireles Sales De Bruin^4^

##### ^1^Centro Universitário Christus, Faculdade de Medicina, Fortaleza, Brazil; ^2^Universidade Federal do Ceará, UFC, Faculdade de Medicina, Bolsista do CNPQ, Fortaleza, Brazil; ^3^Centro Integrado de Diabetes e Hipertensão (CIDH) do Ceará, Fortaleza, Brazil; ^4^Universidade Federal do Ceará, UFC, Faculdade de Medicina; PPGMC, Fortaleza, Brazil

*Diabetology & Metabolic Syndrome* 2019, **11(Suppl 1):**P254

**Background:** Pregnancy is a difficult challenge for women with type 1 Diabetes (T1DM), as it is known to worsen metabolic instability. Overall, sleep disorders are frequent during pregnancy. Poor sleep quality and short duration of sleep are associated with insulin resistance, impairment of glucose metabolism and could be an additional influence on pregnancy outcomes. In spite of these important physiologic relationship, the quality of sleep during pregnancy complicated by type 1 Diabetes has not been well documented.

**Objective:** To assess sleep difficulties, the quality of sleep and daytime sleepiness in a group of patients with T1DM during pregnancy attending a referral service in northwest of Brazil.

**Methods:** This is a cross sectional study, with convenience sample. Data were collected through an interview. Sleep quality was assessed using the Pittsburgh Sleep Quality Index (PSQI). Poor sleep was defined if PSQI score > 5. Daytime sleepiness was assessed using the Epworth Sleepiness Scale (ESS): an ESS score ≥ 10 defining excessive daytime sleepiness. The study was approved by Instituto para o Desenvolvimento da EducaçãoLtda-IPADE ethic board, approval number 1.801.860. The statistical analysis was performed by software IBM SPSS Statistics.

**Results:** Twenty pregnant women, mean age 25.5 y (range 16 to 33 years), with an average disease time of 10 y (range 5 to 20 years), high school level of education (60%) and employed (60%) were evaluated. All patients were treated with intensive basal bolus insulin therapy. It was observed that 60% of the patients had proper weight according to gestational age. Sleep quality assessed by PSQI had an average score of 6.14 (SD: 2.34). Poor quality of sleep (PSQI ≥ 5) was observed in 64.3% of the patients, and 75% referred sleep fragmentation due to night time urination. Short sleep (less than 6 h) was observed in 40% of them. Excessive daytime sleepiness, assessed by ESS score was found in 30% of the patients.

**Conclusions:** This study shows that sleep problems were common in pregnant women with T1DM: poor sleep quality was present in the majority and excessive daytime sleepiness affected one third. Fragmented sleep, partially associated with frequent urination, was frequent, and short sleep duration was found in a lesser extent, It is suggested that the sleep profile in type 1 diabetic pregnant women is important to be evaluated as it could be a modifiable risk factor for adverse pregnant outcomes.

### P255 Stratification type 1 diabetes mellitus with autonomic cardiovascular neuropathology based on heart rate variability during rest by k-means clustering

#### Pena FPS, Materko W, Azevedo JAS, Pena JLC, Barbosa LLS, Ferreira DQ, Silva EM, Santos MSL

##### Universidade Federal do Amapá, Macapá, Brazil

*Diabetology & Metabolic Syndrome* 2019, **11(Suppl 1):**P255

**Introduction:** Patients with type 1 diabetes mellitus (T1DM) exhibit impairments in autonomic and cardiovascular control, including reduced heart rate variability (HRV) is associated with sudden arrhythmic death and increased risk of mortality.

**Objective:** The purpose of this study was to stratify the degree of T1DM with autonomic cardiovascular neuropathology from the heart rate variability by Kmeans clustering.

**Methods:** Forty adults both genders, 20–25 years old, served as subjectsfollowing two conditions: T1DM with neuropathology group (Group 1, n = 20) and control group (Group 2, n = 20). The study protocol was approved by a local Ethical Human Research Committee of Sate University of Rio de Janeiro (CAAE 41891315.3.0000.5259), and an informed written consent was obtained from all participants. All subjects were instructed to lay supine position for 5 min at rest while breathing normally for the acquisition of the cardiac signal by means of a conventional 12-lead ECG (Cardiofax 8110, Nikon Konden) and HRV analysis in the time domain parameters (MeanRR, SDNN, RMSSD and pNN50) was performed by the SinusCorMatlab package software. Descriptive statistical analyses of the data were expressed as mean ± standard deviation. The Kolmogorov–Smirnov test confirmed the normality of distribution. The difference HRV parameters were compared between groups by Student *t* test. All procedures assumed p ≤ 0.05 and were processed in theSPSS 22 software.

**Results:** The K-means clustering method was applied to the HRV analysis to obtain two groups with 100% sensitivity, 75% specificity and 87.5% global accuracy and according to Student *t* test, the cluster of Group 1 showed MeanRR (782.0 ± 84.0 vs. 1048.6 ± 67.4 ms, p < 0.01), SDNN (87.5 ± 42.0 vs. 87.5 ± 42.0 ms, p < 0.01), RMSSD (37.5 ± 22.2 vs. 87.5 ± 47.5 ms, p < 0.01) and pNN50 (16.7 ± 19.0 vs. 46.3 ± 26.9%, p < 0.01) values significantly lower than Group 2.

**Conclusions:** The findings indicate that the proposed method appears to be capable of stratifying the degree of T1DM with autonomic cardiovascular neuropathology.

Palavras Chaves: Type 1 diabetes mellitus, diabeticautonomicneuropathy, cardiovascular disease

### P256 Superior efficacy of insulin degludec/liraglutide (IDEGLIRA) vs insulin glargine as add-on to SGLT2I ± oral antidiabetic drug therapy in patients with type 2 diabetes: duaL IX trial (NCT02773368)

#### Emanuela Mello Ribeiro Cavalari^1^, Athena Philis-Tsimikas^2^, Liana K. Billings^3^, Robert Busch^4^, Cristobal Morales Portillo^5^, Rakesh Sahay^6^, RutaGronskyte Juhl^7^, Stewart Harris^8^

##### ^1^Novo Nordisk, Brazil; ^2^ScrippsWhittierDiabetes Institute, San Diego, USA, ^3^NorthShore University HealthSystem, Evanston, USA; ^4^Albany Medical Centre, New York, USA; ^5^Hospital Virgen Macarena, Seville, Spain; ^6^Osmania Medical College, Hyderabad, India; ^7^Novo Nordisk A/S, Søborg, Denmark; ^8^Western University, London, ON, Canada

*Diabetology & Metabolic Syndrome* 2019, **11(Suppl 1):**P256

**Introduction:** At the start of DUAL IX study, there were no trials published with a GLP-1RA in combination with a Sodium-Glucose Co-Transporter-2 Inhibitor (SGLT-2i).

**Objective:** The aim of DUAL IX is to examine the efficacy and safety of insulin Degludec/Liraglutide (IDegLira) as an add-on to SGLT-2i in patients with type 2 diabetes (T2D) failing to achieve glycemic control on SGLT-2i.

**Methods:** In this 26-week, phase 3b, open-label trial, 420 patients with T2D uncontrolled on SGLT2i ± oral antidiabetic drug (OAD) were randomized 1:1 to receive add-on therapy of IDegLira or IGlar U100 (100 units [U]/mL). Starting doses were 10 U in both treatment arms. Doses were titrated twice-weekly to a fasting glucose target of 72 to 90 mg/dL; only IDegLira had a maximum dose (50 U). Inclusion criteria: Age ≥ 18 years, Insulin-naїve, HbA1c 7.0–11.0%, SGLT-2i ± other OADs, BMI ≥ 20 and < 40 kg/m^2^. The primary endpoint is change from baseline in HbA1c after 26 weeks and confirmatory secondary endpoints are: change from baseline after 26 weeks in body weight, number of treatment-emergent severe or blood glucose-confirmed symptomatic hypoglycemic episodes and total daily insulin dose.

**Results:** Mean A1C decreased from 8.2% at baseline to 6.3% at week 26 for IDegLira and from 8.4 to 6.7% for IGlar U100; IDegLira superiority confirmed (*p *< 0.0001). IDegLira treatment resulted in unchanged mean body weight vs 2.0 kg weight gain with IGlar U100 (*p *< 0.0001). The rate oftreatment emergent severe or blood glucose confirmed symptomatic hypoglycemic episodes was 58% lower (*p *= 0.0035) with IDegLira (0.37 events/patient-year of exposure [PYE]) vs IGlar U100 (0.90 events/PYE). Total daily insulin dose after 26 weeks was 36 U for IDegLira vs 54 U for IGlar U100 (*p *< 0.0001). Adverse event rates were low in both treatment arms with no unexpected safety issues.

**Conclusions:** The DUAL IX study demonstrates the efficacy and safety of IDegLira treatment as an add-on to SGLT-2i in patients with T2D uncontrolled on SGLT-2i ± OADs. Superiority of IDegLira vs IGlar U100 as add-on to SGLT2i was confirmed for glycemic control, body weight, hypoglycemia rate and total daily insulin dose. The results of DUAL IX indicate that IDegLira may be a better treatment option than IGlarU100 in patients on SGLT-2i in need of intensification.

### P257 Survival assessment in patients with prostate cancer and diabetes mellitus

#### Carla Simone Moreira de Freitas^1^, Aleida Nazareth Soares^2^

##### ^1^Fundação Cristiano Varella - Hospital do Câncer de Muriaé, Muriaé, Brazil; ^2^Instituto de Ensino e pesquisa da Santa Casa de BH, Belo Horizonte, Brazil

*Diabetology & Metabolic Syndrome* 2019, **11(Suppl 1):**P257

**Introduction:** Prostate cancer is considered the cancer that most affects men in Brazil. Diabetes Mellitus (DM) has been associated with a lower risk of prostate cancer. However, studies do not yet prove whether DM influences the survival of prostate cancer patients after diagnosis.

**Methods:** A retrospective study of 3099 patients enrolled from 2007 to 2018 with prostate cancer, 454 (14.6%) with DM and 2645 (85.4%) without DM on hormone therapy for prostate cancer treatment. Survival curves were calculated by the Kaplan–Meier method and compared by the Log-Hank test. Software used SPSS 22.0.

**Methods:** A retrospective study of 3099 patients enrolled from 2007 to 2018 with prostate cancer, 454 (14.6%) with DM and 2645 (85.4%) without DM on hormone therapy for prostate cancer treatment. Survival curves were calculated by the Kaplan–Meier method and compared by the Log-Hank test. Software used SPSS 22.0.

**Objective:** To compare the survival of prostate cancer patients on hormone therapy with and without diabetes mellitus

**Results:** Mean age group DM: 68.8 years (± 8.7) and 68.2 years (± 8.9) for non-DM group. Gleason classification for DM group: 227 (50%) Gleason 6 (low risk), 139 (30.8%) patients Gleason 7 (intermediate risk), 88 (19.2%) Gleason 8.9 and 10 (high risk). In the group without DM: 1,491 (56.4%) Gleason 6, 749 (28.3%), Gleason 7, 404 (19.2%) Gleason 8,9 and 10, The overall 5-year survival of the DM group was 89.5% (95% CI: 85.5; 94.5%) and the 10-year SG was 73.8% (95% CI: 64.8%; 82.8%). In the group without DM, the 5-year SG was 91.3% (95% CI: 89.8%; 92.8%) and the 10-year SG was 84.4% (95% CI: 81.8%); 87.0%), p = 0.053.

**Conclusion:** This study suggests that patients with prostate cancer and DM have slightly lower SG than the group without DM.

### P258 switching to iglarlixi vs. continued treatment oF GLP-1RA: comparative analysis by daily or weekly GLP-1RAS in the LIXILAN-G trial

#### Julio Rosenstock^1^, Lawrence Blonde^2^, Stefano Del Prato^3^, Robert R. Henry^4^, Naim Shehadeh^5^, Elisabeth Niemoeller^6^, Elisabeth Souhami^7^, Vanita R. Aroda^8^

##### ^1^Dallas Diabetes Research Center at Medical City, Dallas, USA; ^2^Department of Endocrinology, Ochsner Medical Center, New Orleans, USA; ^3^University of Pisa School of Medicine, Pisa, Italy; ^4^UC San Diego and VA San Diego Healthcare System, Center for Metabolic Research, San Diego, USA; ^5^Rambam Health Care Campus, Haifa, Israel; ^6^Sanofi, Frankfurt, Germany; ^7^Sanofi, Paris, France; ^8^MedStar Health Research Institute, Hyattsville, MD, and Brigham and Women’s Hospital, Boston, USA

*Diabetology & Metabolic Syndrome* 2019, **11(Suppl 1):**P258

**Introduction and objectives:** LixiLan-G (NCT02787551) was a randomized, open-label, 26-week trial in T2D participants (pts) with HbA1c 7‒9%, receiving maximum tolerated doses of a once- or twice-daily (QD/BID) GLP-1 RA or a once-weekly (QW) GLP-1 RA with metformin ± pioglitazone ± sodium-glucose cotransporter 2 inhibitor (SGLT2i). Pts were randomized to continue their GLP-1 RA regimen with supported adherence or switch to a fixed-ratio combination of insulin glargine and lixisenatide (iGlarLixi). This exploratory analysis assessed efficacy and safety by daily or weekly GLP-1 RA use at the screening.

**Methods and results:** Among 505 of 514 randomized pts (n = 252 of 257 randomized to iGlarLixi and n = 253 of 257 randomized to GLP-1 RA), 60% and 40% were on daily and weekly GLP-1 RA at screening, respectively (liraglutide QD 54%, dulaglutide QW 20%, exenatide extended-release QW 18%, exenatide BID 5%, albiglutide QW 2%). Mean age was similar across subgroups, but mean T2D duration and GLP-1 RA treatment duration were slightly longer in the daily than in the weekly subgroup. Change in HbA1c was larger with iGlarLixi vs. GLP-1 RA regardless of GLP-1 RA subtype (least-squares [LS] mean difference: − 0.7 [95% confidence interval (CI): − 0.8, −0.5] with daily formulations, and − 0.6 [95% CI: − 0.8, − 0.4] with weekly formulations). Similar results were observed for LS mean differences in fasting plasma glucose (− 28.4 [95% CI: − 36.0, − 20.8] with daily formulations, and − 33.2 [95% CI: − 42.8, − 23.5] with weekly formulations) and 2-h postprandial plasma glucose after a standardized breakfast meal (− 47.5 [95% CI: − 61.3, − 33.6] with daily formulations, and − 57.2 [95% CI: − 72.6, − 41.7] with weekly formulations). Safety profiles of iGlarLixi and GLP-1 RA were consistent with previous publications, as adverse events were nominally more common with iGlarLixi vs. GLP-1 RA (documented symptomatic hypoglycemia: 0.27 vs 0.01 events/pt-year for QD/BID, and 0.21 vs 0 events/pt-year for QW; nausea: 10.5% vs 2.6% for QD/BID, and 5.8% vs 1.9% for QW; vomiting: 4.6% vs 0.7% for QD/BID, and 1.0% vs 1.0% for QW).

**Conclusions:** The benefits of switching to iGlarLixi vs. continuing GLP-1 RA in inadequately controlled T2D are observed irrespective of daily or weekly GLP-1 RA use. The trial was sponsored by Sanofi.

### P259 Taste of the earth: full use of regional foods

#### Ana Paula Tavares De Oliveira^1^, Belmira Silva Faria E Souza^2^, Maria Do Socorro Da Silva Martins^2^, Alessandra Silva Monteiro^2^, Diego Quaresma Ferreira^2^, Eloisa Melo Da Silva^2^, Adriane Stefanny Rocha Ribeiro^2^, Marluci De Souza Lédo Santos^2^

##### ^1^Faculdade Estácio de Macapá, Macapá, Brazil; ^2^Universidade Federal do Amapá-Unifap, Macapá, Brazil; ^3^Faculdade de Macapá-Fama, Macapá, Brazil

*Diabetology & Metabolic Syndrome* 2019, **11(Suppl 1):**P259

**Introduction:** Healthy eating is provided for in national policies and programs, as it promotes people’s quality of life. The eating practices of the Brazilian population are inadequate at all stages of life, due to lack of access and lack of knowledge about the integral use of food. The Getúlio Vargas Foundation reveals that 41.6 lb of food per person is wasted each year. In parallel, there is an increase in obesity and non-communicable chronic diseases such as diabetes mellitus-DM. In this context, the work of nutritional education in the Health Promotion Program for people with DM, was built the Primer “Taste of the Earth: Full utilization of regional foods. Objective: To build educative-care technology (booklet) to aid in the food plan full utilization of food, adding nutritional value and economy to preparations.

**Method:** Methodological study of qualitative approach, for the construction of the booklet to assist people with DM in the eating plan. Conducted interviews with program participants about food waste. From the data analysis, an integrative review was performed, identifying and analyzing the existing categories of food waste in the literature. The interview data and the review categories constituted the basis for the construction of the technology. Approved in zip code no. 2,430,811, CAAE: 80829617.8.0000.0003.

**Results:** The study was presented and explained the technology to the participants, and educational work was done in two meetings. Awareness Raising for the Full Use of Food—“Do not throw anything away, and use creativity”. The nutritional information of each part of the food, as it could be used, including the reuse of daily leftovers, demonstrating economic aspects. The explanation was aided by data show, video and table with fresh food exposition. 2° setting up a table with the preparations with the full utilization of food using recipes from the booklet. Then the tasting of the preparations was made and at the end a conversation wheel for questions and suggestions.

**Conclusion:** The guidelines of the booklet help in the full utilization of food and in the construction of food plans favoring economic aspects and ingestion of greater nutrient input including constituent fibers that are essential to the health of people with DM.

### P260 Techniques for theoretical improvement and practical simulation with insulin analogs: strategies for knowledge acquisition

#### Gemiliana Sombra de Oliveira Carvalho, Samila Torquato Araújo, Marília Araripe Ferreira, Tatiana Rebouças Moreira, Francisca Diana da Silva Negreiros, Synara Cavalcante Lopes, Virginia Oliveira Fernandes, Renan Magalhães Montenegro Júnior

##### Universidade Federal do Ceará, Fortaleza, Brazil

*Diabetology & Metabolic Syndrome* 2019, **11(Suppl 1):**P260

**Introduction:** The occurrence of gaps in professional formation and diabetes training has negatively impacted the quality of care assistance and the population’s physical and mental health. Simulation-based pedagogical approaching may be an alternative strategy to overcome this issue through technical practice and problem-solving tasks, thereby favoring the development of specific skills.

**Objective:** To evaluate the effectiveness of techniques for theoretical improvement and practical simulation with insulin analogues for continuing education of professional residents.

**Methods:** This was a cross-sectional study carried out between February and June 2018 with thirty-seven residents of a university hospital in the state of Ceará, Brazil. Two evidence-based questionnaires developed for the purpose of this study were used: the first questionnaire included sociodemographic data and the second consisted of pretest (P1) and post-test (P2) items to assess the residents’ knowledge before and after intervention, with five multiple choice questions about insulin analogues (storage, validity after seal opening, combinations between insulins, injection sites, rotation of injection sites, injection technique and disposal of sharp material). The simulation was carried out using insulin analogues for the practice of insulin therapy. Seven meetings were undertaken, each containing approximately 5 to 6 participants, with an average length of 2 h. The Wilcoxon test was used for pairwise comparison between P1 and P2. This study followed all ethical principles involving research with human beings.

**Results:** Of the thirty-seven participants, 31 (83.8%) were women, with a mean age of 26.46 years (± 5.6), and 16 (43.2%) had an income higher than five minimum wages. Three (8.1%) participants were residents in Internal Medicine, 4 (10.8%) in Pediatric Endocrinology, 11 (29.7%) in Diabetes, 4 (1.8%) in Oncohematology, 3 (8.1%) in Mental Health, and 5 (13.5%) in Intensive Care. The outcomes indicated a greater correct response rate after intervention, with a significant difference between P1 and P2 (*P *< 0.001). The median of correct responses was 3 at P1 and 5 at P2. The 25th and 75th percentiles were up to 2 and 4 questions at baseline and 4 and 5 questions after the meeting, respectively.

**Conclusion:** Theoretical training combined with simulation of skills for insulin therapy was an effective continuing education strategy for professional residents.

### P261 The effect of exercise in body mass index and glycated hemoglobin levels in type 1 diabetes patients

#### Luiza Costa Monteiro Hadler^1^, Raquel Oliveira Gomes^1^, André Neves Mascarenhas^2^, Iago Barbosa Pinto Rodrigues^2^, Isabela Fernandes Araújo^2^, Amanda Cristine Amador de Moura^2^, Maria Claret Costa Monteiro Hadler^3^, Hermelinda Cordeiro Pedrosa^1^

##### ^1^Unidade de Endocrinologia e Diabetes do Hospital Regional de Taguatinga (HRT), SES/DF, Taguatinga, Brazil; ^2^Escola Superior de Ciências da Saúde (ESCS), SES/DF, Brasília, Brazilia; ^3^Universidade Federal de Goiás (UFG), Goiânia, Brazil

*Diabetology & Metabolic Syndrome* 2019, **11(Suppl 1):**P261

**Introduction:** Exercise is important for the health and well being of people with type 1 diabetes (T1DM). The aerobic exercise tends to lower blood glucose and anaerobic exercise likely to increase glucose, making glycaemic control challenging. However, the hypothesis that combined information from the practice of exercise associated with reduction of glycated hemoglobin (HbA1C) in T1DM individuals has been little tested.

**Objective**: To evaluate the association of exercise practice and reduction of body mass index (BMI) and/or glycated hemoglobin (A1c) in T1DM adult outpatients follow-up.

**Methods:** A cross-sectional, observational and analytical study was carried out with 109 individuals diagnosed with T1DM, who attended an Endocrinology and Diabetes United (UENDO) at a secondary hospital, during two years. HbA1c levels were measured using a high-performance liquid chromatography. Exercise practice was performed with a minimum frequency of twice a week of exercises, at least 30 min, of low to moderate intensity. Normally distributed variables were displayed as means ± standard deviations and the median (interquartile range) were displayed with a non-normal distribution. The Mann–Whitney test was applied to compare medians among the participants and non-participants of exercise group. Chi square test and Student’s t-test were also used. Data were analyzed with the SPSS—Statistical Package for Social Science version 18.0 and the protocol was approved by the regional Ethics Committee.

**Results:** Of the 138 eligible individuals, twenty-six refused to participate or were not contacted and 3 had incomplete data for exercise resulting in 109 participants (aged 16–54 years, 51 males (46.8%)). The age of practitioners (30.21 ± 9.19 years) did not differ from non-practitioners (28.21 ± 9.21) (p = 0.317). About 78.43% (n = 40) of men and 69.0% of women (n = 40) practiced exercise, not differing between gender (p = 0.264). The mean of BMI did not differ between practitioners (24.22 ± 3.47) and non-practitioners of exercise (25.19 ± 5.96). The medians and interquartile ranges (7.7% P25°: 6.90%–P75°: 8.65) of the A1c of the practitioners were significantly lower than the median and interquartile ranges (9.1% P25°: 7.10%–P75°: 10.30%) of the A1c of the non-practitioners of exercise (p = 0.013).

**Conclusion:** The study demonstrated a decrease in A1c levels among exercise practitioners, but did not change BMI compared to non-exercise practitioners.

### P262 The effect of fast-acting insulin aspart versus insulin aspart on glycaemic control according to age at baseline in children and adolescents with type 1 diabetes

#### Victor França De Almeida^1^, Thomas Danne^2^, Bruce Bode^3^, Srikanth Deenadayalan^4^, Johan Ejstrud^5^, Lori Laffel^6^

##### ^1^Novo Nordisk Ltda, São Paulo, Brasil; ^2^Children’s Hospital Auf der Bult, Hannover, Germany; ^3^Atlanta Diabetes Associates, Atlanta, GA, USA; ^4^Novo Nordisk A/S, Søborg, Denmark; ^5^Novo Nordisk A/S, Aalborg, Denmark; ^6^Joslin Diabetes Center, Harvard Medical School, Boston, MA, USA

*Diabetology & Metabolic Syndrome* 2019, **11(Suppl 1):**P262

**Introduction:** The aim of this post hoc analysis was to explore the effect of fast-acting insulin aspart (faster aspart) versus insulin aspart (IAsp) on glycaemic control according to age at baseline in children and adolescents with type 1 diabetes (T1D) (the onset 7 study population).

**Objective:** To explore the effect of fast-acting insulin aspart (faster aspart) vs insulin aspart (IAsp) on glycaemic control according to age at baseline in a paediatric sample with type 1 diabetes (T1D).

**Methods:** A post hoc analysis of a 26-week, treat-to-target, multicentre trial that randomised participants to mealtime faster aspart (n = 260), post-meal faster aspart (n = 259), and mealtime IAsp (n = 258), all with daily insulin degludec. The analysis evaluated the 26-week treatment effect of faster aspart vs IAsp on change in HbA1c from baseline, according to age at baseline as a continuous variable.

**Results:** At week 26, the primary analysis showed that mealtime and post-meal faster aspart were non-inferior (0.4% margin) to IAsp for the change in HbA1c from baseline, with a statistically significant difference in favour of mealtime faster aspart (estimated treatment difference [ETD 95%CI]: − 0.17% [− 0.30; − 0.03]; − 1.82 mmol/mol [− 3.28; − 0.36], p = 0.014). Results of the post hoc analysis showed there were no statistically significant differences between the regression coefficient of faster aspart and IAsp in the change in HbA1c from baseline at week 26 vs age at baseline (ETD [95%CI] mealtime faster aspart—IAsp: − 0.02 [− 0.05;0.02], p = 0.38; post-meal faster aspart—IAsp: 0.00 [− 0.04;0.04], p = 0.99).

**Conclusion:** The treatment difference between faster aspart and IAsp in change in HbA1c from baseline was independent of age in children and adolescents with T1D.

### P263 The knowledge of transplanted healthcare users about diabetes mellitus in a University Hospital

#### Tatiana Rebouças Moreira^1^, Yasmim Néri Pinheiro^1^, Francisca Diana da Silva Negreiros^1^, Rafaella Roque Chagas^1^, Gemiliana Sombra de Oliveira Carvalho^1^, Silvana Linhares de Carvalho^1^, Claudia Maria Marinho de Almeida Franco^2^, Magalhães Montenegro Júnior^1^

##### ^1^Hospital Universitário Walter Cantídio/Universidade Federal do Ceará, Fortaleza, Brazil; ^2^Centro Universitário Christus, Fortaleza, Brazil

*Diabetology & Metabolic Syndrome* 2019, **11(Suppl 1):**P263

**Introduction:** Diabetes mellitus (DM) is a serious public health issue with high treatment and hospitalization costs due to associated complications. Poor diabetes management can lead to severe health impairment, such as kidney disease, and pose a need for organ transplantation. In addition, transplanted patients may develop post-transplant DM as a result of the use of immunosuppressants. Hence, the patient’s knowledge on DM is critical for successful self-management of the condition.

**Objective:** To evaluate the knowledge on DM of kidney-transplanted healthcare users.

**Methods:** This was a descriptive cross-sectional study with a quantitative approach, including 51 kidney-transplanted individuals with DM. The study was carried out between May and November 2018 in a university hospital in the state of Ceará, Brazil. The data were collected using two questionnaires, one to obtain information on clinical variables and the Diabetes Knowledge Scale, which is validated for Brazil and determines the patient’s knowledge about the disease. This study followed all ethical principles of research involving human subjects.

**Results:** The most frequent types of diabetes were post-transplant DM (*n* = 28, 54.9%) and type 2 DM (*n* = 20, 39.2%). The length of time since diagnosis ranged from 1 year to 39 years, with an average of 14.1 years. The length of time since transplantation ranged from 1 year to 30 years, with an average of 8.5 years, with 35 (68.6%) patients transplanted over the last 10 years. Among the drugs used for diabetes control, there was a predominance of oral antidiabetic drugs (*n* = 38, 74.5%), followed by injectables (*n* = 30, 58.8%) (*e.g*., human insulin and insulin analogues). Most surveyed healthcare users were knowledgeable about DM (*n* = 30, 58.8%). The highest correct response rates were observed in questions about diabetes control, food groups, microvascular complications, and management of hypoglycemia. The highest rates of incorrect responses were observed in questions about normoglycemic values, presence of ketonuria as a sign of poor diabetes control, and food substitution.

**Conclusion:** Although the surveyed transplanted patients demonstrated knowledge on DM, there was lack of knowledge about essential aspects related to disease management and self-care. These points need to be better discussed with the transplanted patient by the multiprofessional team.

### P264 The largest difference between lower limb fat and trunk fat is related to the less risk for add cardiovascular diseases: longitudinal adult health study results (ELSA-Brasil)

#### Bárbara Bruna Rodrigues De Oliveira^1^, Carolina Gomes Coelho^2^, Sandhi Maria Barreto^2^, Luana Giatti^2^, Larissa Fortunato Araujo^1^

##### ^1^Universidade Federal do Ceará, Fortaleza, Brazil; ^2^Universidade Federal de Minas Gerais, Belo Horizonte, Brazil

*Diabetology & Metabolic Syndrome* 2019, **11(Suppl 1):**P264

**Introduction:** Previous evidence suggests that fat accumulation in the trunk is more detrimental and lower limb fat exerts a protective effect against metabolic disorders and cardiovascular diseases.

**Objective:** To investigate the association of the ratio between lower limb and trunk fat with the risk for cardiovascular disease in men and women participating in the Brazilian longitudinal study of adult health (ELSA-Brasil).

**Methods and results:** This is a cross-sectional study with 10,917 participants from the second wave (2012–2014) of ELSA-Brasil. The outcome variable was the 10-year risk for cardiovascular disease estimated by the Framingham risk score. The explanatory variable was the ratio between the amount of fat in the lower limb and trunk, in kilograms, determined by an electrical bioimpedance device. The arithmetic means ratio and their confidence interval of 95% were estimated by generalized linear models using sex as the stratification variable. Sequential adjustments were made for age, self-reported race/skin-color, educational attainment, physical activity, alcohol consumption, hypolipidemic drug use and menopausal status (for women). Analyzes were performed using Stata 13.0. The research was approved by the National Commission for Research Ethics (CONEP) through the approval letter of No. 976/2006, by the Research Ethics Committee of the Federal University of Minas Gerais (COEP/UFMG) and by the ethics committees of the other institutions involved in the study. Informed consent was obtained from all individual participants included in the study.

**Results:** Most of the study population were women (55.52%) with 50 and 54 years old (men 25.13%; women 25.68%) and the median percentage risk for cardiovascular disease was 10.86% (6.83–17.66) in men and 4.31% (2.65–7.19) in women. After complete adjustment, we observed that the one-unit increase in the difference between lower limb and trunk fat decreases by 31% (95% CI 0.64–0.74) in men and 77% (95% CI 0.18–0.29) in women the mean risk for cardiovascular disease.

**Conclusion:** Individuals with a higher amount of lower limb fat compared to trunkfat had lower risk for cardiovascular disease and this effect was stronger in women than in men. Technical and financial support provided by Ministry of Health, Department of Science, Technology and Strategic Inputs, Department of Science and Technology, Ministry of Science and Technology, National Council for the Development of Science and Technology.

### P265 type 1 diabetes onset after antiretroviral treatment for HIV infection

#### Elias Neif Srur Filho, Esther Cytrynbaum Young, Mariana de Almeida Pinto Borges, Paula Fernanda Rodrigues de Mattos Cardoso, Márcia Helena Soares Costa, Flavia Regina Barbosa, Clety Larisa Angulo Lierena

##### Universidade Federal do Estado do Rio de Janeiro UNIRIO, Rio de Janeiro, Brazil

*Diabetology & Metabolic Syndrome* 2019, **11(Suppl 1):**P265

**Introduction:** Contemporary antiretroviral treatment (ART) has been shown to be effective in reducing morbidity and mortality associated with human immunodeficiency virus (HIV) infection. However, it changed its clinical course, increasing life expectancy, inducing several metabolic complications such as insulin resistance (IR), diabetes, metabolic syndrome (MS) and turning it into a chronic disease. Both HIV infection and ART use are associated with increased incidence of type 2 diabetes due to increased (RI) and glucagon. HIV infection associated with type 1 diabetes (T1DM) is rarely described.

**Objective:** To present a case of DM1 after ART use.

**Methodology:** Case report of a patient who developed (T1DM) 3 years after ART with protease inhibitors (PI). The project was approved by the local Ethics Committee.

**Case report:** A 23-year-old female patient from Rio de Janeiro, who has the HIV virus by vertical transmission. She started ART at 7 years of age, with subsequent scheme changes due to adverse reactions and resistance to the HIV virus. She developed DM1 at 12 years of age with major symptoms of diabetes and fasting glucose 400 mg/dL. At that time she was using Lopinavir-Ritonavir (LPV-RPV), Didanosine (DDI), and Stavudine (D4T) for 3 years. She was admitted to the endocrinology service using human insulin therapy. During this period the medication was changed to Tenofovir (TDF), Lamivudine (3TC) (both nucleoside reverse transcriptase inhibitors), Atazanavir (ATV) and Ritonavir (RTV) (both PI). Viral load was undetectable. She developed lipodystrophy, severe hypertriglyceridemia (Tg: 1746 mg/dL), being prescribed Rosuvastatin 20 mg, Ciprofibrate 100 mg and started insulin analogues: Levemir and Novorápid. At 21 years of age, she was diagnosed with lumbar spine osteoporosis (bone densitometry: L4–L5: − 2.5), when the ARV scheme was switched to Abacavir (ABC) + Lamivudine (3TC) + Atazanavir (ATV) + Ritonavir (RTV), which remains in use until the present moment.

**Conclusions:** Although most patients with HIV-associated diabetes are classified as type 2 diabetes, some patients may develop DM1 after ART. The appearance of DM1 in these patients has been associated with the improvement of CD4 levels, viral load and genetic predisposition. In the face of the HIV-Diabetes epidemic, knowledge of possible metabolic interactions of these drugs will provide a better approach and preventive measures.

Informed consent to publish had been obtained from the patient.

### P266 Type 2 diabetes mellitus: comorbidities, clinical complications and behavioral variables in an amazonas sample

#### Lucivana Prata de Souza Mourão^1^, Marjory Ximenes Rabelo^1^, Camila Soares Santos^1^, Ricardo Cordeiro Lyra Júnior^1^, Kettyuscia Coelho e Oliveira^1^, Larissa Pereira Gomes Figueiredo^1^, Adolfo José da Mota^2^

##### ^1^Universidade do Estado do Amazonas, Manaus, Brazil; ^2^Universidade Federal do Amazonas, Manaus, Brazil

*Diabetology & Metabolic Syndrome* 2019, **11(Suppl 1):**P266

**Introduction:** Type 2 Diabetes (T2D) is a serious public health problem, its severity is due to the comorbidities while the high death rates are consequence of macro and microvascular complications. Unhealthy habits and genetic factors are pointed as the main risk factors that explain the high rates of the disease and can double within 28 years. Public health policies should ensure a constant surveillance system, efficient in monitoring its progress and whose data could feed effective preventive programs. However, for some regions of the Brazil, there is a complete absence of data. Thus, the main goal of this research was to evaluate the prevalence of comorbidities, clinical complications and behavioral variables associated to the T2D in people attending by the public health system of Manaus-AM, Brazil.

**Methods:** This is a descriptive and analytical research (Ethics Committee approval: CAAE 60172416.8.0000.5020) with people diagnosed with T2D in regular clinical follow-up by the public health system. Participants answered a questionnaire adapted from the FINDRISK. The data were analyzed by descriptive statistics and the variables presented by frequency. Pearson’s Chi square test was used for evaluate the associations between variables.

**Results:** Fifty-three people participated in this survey, being the highest prevalence of females (68.5%), with average age of 61.1 + 14.6 and time for the diagnosis of 9.1 + 9.3 years. For comorbidities, there was a high frequency of hypertension (62.3%), overweight (35.8%) and obesity (43.4%). The ophthalmic complication was the most frequent (73.6%). There was a significant association between (1) time since diagnosis and clinical complications; (2) dyslipidemia, or BMI, and more than one clinical complication; (3) BMI and ophthalmic complications. The participants reported use of tobacco (35.8%), alcohol (54.7%) and healthy eating (67.9%). The frequency of regular physical activity is low (32.1%), while the familial aggregation is highly frequent (69.8%) in this population. Although 67.9% of participants reported healthy diet, the rate of overweight and obese is very high, almost 80%.

**Conclusions:** This study contributed with information about the profile of individuals with T2D in Manaus. Despite the notorious importance of this disease, there is no data in the scientific medical literature for the population of Amazonas that allow the adoption of effective public policies to control of the disease in the state.

### P267 UKPDS cardiovascular risk profile in people with type 2 diabetes mellitus

#### Rosilei Teresinha Weiss Baade^1^, Susamar Ferreira da Silva^1^, Salete Regina Daronco Benetti^2^

##### ^1^Secretaria Municipal de Saúde de São Bento do Sul - Centro de Atendimento ao Diabético - CADIA, São Bento do Sul, Brazil; ^2^UNC - Universidade do Contestado, Mafra, Brazil

*Diabetology & Metabolic Syndrome* 2019, **11(Suppl 1):**P267

**Introduction:** Diabetes Mellitus (DM) is considered a heterogeneous group of diseases, which is between the main causes of morbidity and mortality. Constitute one of the main risk factors for cardiovascular events, because the hyperglycemia oxidize the blood vessels, elevating the occurrence of the angina, heath attack and stroke. The research looked for a form to trace these individuals with unfavorable prognosis, identify diabetic patients type II that are asymptomatic and will be benefited by the tests and diagnosis to early detect the CvD, looking for preventive and therapeutic ways.

**Objective:** Investigate cardiovascular risk in medical records of people with type II diabetes who seek care in the diabetes care center.

**Methods:** Study of quantitative approach, exploratory, documental and bibliographic. 171 of the diabetes care center medical records were searched and 109 eligible were analyzed. The foundation of this research are collect data the software UKPDS Risk Engine was used, a risk calculator for cardiovascular diseases specific for diabetes type II, made by the Oxford University—United Kingdom.

**Results:** It was found that the highest number of people with DM II insulinized were elders between 60 and 64 and 70–74 years of age;19.26% each. From the total of 109 users, 59.6% are women and 40.4% are men. Referring the score of risk to CHD: 37.6% user with results between 4% and 9%—low risk; 35.8% users with results between 11% and 19%—medium risk, 26,6% users with results between 21% and 68%—high risk. Referring the score to fatal risk of CHD: 51.4% users showed results between 2% and 9%,—low risk, 31.2% users with results between 10% and 19%—medium risk, 17.4% users with results between 21% and 60%—high risk. Regarding the possibility of Stroke (AVC): 59.6% with results between 2% and 9%,—low risk, 22% with results between 10% and 19%,—medium risk, 18.3% with results between 21% and 88%,—high risk. Regarding the score for fatal AVC the research shows: 98.2% users showed results between 0.2% and 9%—low risk: 1.8% users with results between 10% and 18%—medium risk, with average of 14%. No users were found in this research with help of the UKPDS calculator that have high risk of having a fatal AVC.

**Conclusions:** The data showed the risk profile of the service users, enabling the implementation of specific preventive and therapeutic measures related to each case.

### P268 Ulceration risk of type 2 diabetes patients: an observational cross-sectional study

#### Rebeca Barbosa da Rocha, Ivoneide Maria Rodrigues de Araújo, Raniel da Silva Machado, Jadiel Marinho Cardoso, Érika Gracy Diniz Sousa, Marília Rufino Mariano, Cristiano Sales Silva, Vinícius Saura Cardoso

##### Universidade Federal do Piauí, Terezinha, Brazil

*Diabetology & Metabolic Syndrome* 2019, **11(Suppl 1):**P268

**Introduction:** The worldwide prevalence of foot wounds in diabetics is about 6%. This is an onerous condition for public coffers and it reduces the quality of life of individuals. The city of Parnaíba-PI does not present information regarding DM and the risk of injury of the individuals, the acquisition of this information is the first step for the prevention of wounds.

**Objective:** To analyze the risk of wounds in type 2 diabetics of Parnaíba-PI.

**Methods:** This cross-sectional, observational study was performed with 129 patients with Type 2 DM in the city of Parnaíba-PI. The patients answered the sociodemographic questionnaire in an interview format and underwent clinical foot evaluation. The evaluation consisted of inspection, palpation of the pulses (posterior tibial and pedal) and verification of the peripheral sensitivity by means of 10 g monofilament and the tuning fork of 128 Hz. The classification of the risk of wounds: 0—neuropathy absent; 1—presence of neuropathy; 2—Neuropathy with peripheral arterial disease and/or deformities; 3—neuropathy with history of ulcer and/or amputation. The research is approved by the ethics committee of the Federal University of Piauí (Number 2,689,629).

**Results:** The sample is composed mostly of female volunteers (74.4%). The mean age of participants is 61.31 SD 13.3, with mean DM duration in years of 8.1 SD 7.5. Regarding the risk of wounds, 68% of the participants had predisposing factors to ulceration. Of these, 25.5% with risk 1, 20.1% risk 2 and 22.4% for risk 3. The other volunteers (32%) received a classification of 0.

**Conclusion:** It is concluded that the population studied in the majority presents a risk for the development of wounds. The data suggest the need to implement self-care measures, a strategy pointed out in the literature as a determinant for the reduction of complications related to DM.

### P269 Ultrasound effects on mineral bone density and mechanical properties after fracture on diabetic rats

#### Ingrid Marianne De Freitas Santos^1^, Cybelle Da Silva Nery^2^, Diana De Andrade Silva^1^, Misael Carvalho Dos Santos^1^, Beatriz Maia Rangel Moreira^1^, Romero Andion De Medeiros Sobrinho^1^, Victor Franklyn De Oliveira^1^, Silvia Regina Arruda De Moraes^3^

##### ^1^Departamento de Fisioterapia, Universidade Federal de Pernambuco - UFPE, Recife, Brazil; ^2^Programa de Pós-graduação em Biotecnologia/RENORBIO, Universidade Federal de Pernambuco - UFPE, Recife, Brazil; ^3^Departamento de Anatomia, Laboratório de Plasticidade Neuromuscular - UFPE, Recife, Brazil

*Diabetology & Metabolic Syndrome* 2019, **11(Suppl 1):**P269

**Introduction and objectives:** The diabetes mellitus affects the bone tissue, promoting alterations on osteogenesis processes, causing osteopenia and increasing fracture fragility. The ultrasound therapy, by favoring the increase of bone formation, arises as an alternative to help the healing process. The aim was to assess the effects of low intensity ultrasound of 100mW/cm^2^ on the recovery process of femur fractures of diabetic rats through mineral bone density (MBD) and biomechanical analysis.

**Methods:** 51 *Wistar* rats were distributed in four groups: GCP (n = 16), GCU (n = 14), GDP (n = 9) e GDU (n = 12). At 60 days of age, the animals were induced to experimental diabetes (injection of Estreptozotocin solution, STZ- Shimadzu^®^; intraperitoneal; 60 mg/kg) and at 74 days of life (2 weeks after induction), an open fracture of the right femur was performed. From the first until the 24th post-cirurgical day, the treatment with the SONOPULSE ultrasound was initiated, 7 times per week, 20 min per day, pulsed modality, 1 MHz frequency, 100 Hz pulse repetition frequency, 2 ms pulse duration—1:5 ratio, 20% duty cycle and 100mW/cm^2^ intensity. After the treatment conclusion, the animals were euthanized and the femurs were collected for MBD analysis and biomechanical three point bending test assessment through maximum force, maximum tension, maximum deformation, elastic modulus, cross sectional area and femur length.

**Results:** the data was expressed in mean and standard ± deviation (median). The level of significance was 5%. It was not observed significant changes between groups on MBD, however, regarding the biomechanical parameters, only maximum tension showed higher values on GDU [1.22 ± 0.63 (1.13) Mpa] when compared to GDP [0.75 ± 0.61 (0.46) Mpa] (p = 0.032).

**Conclusion:** the ultrasound treatment protocol with 100mW/cm^2^ intensity promoted an increase of maximum tension of femurs of diabetic animals after fracture, highlighting a higher capacity of resisting the required load.

### P270 Unrecognized type 2 diabetes in pregnancy: are we late?

#### Maria Amélia Alves de Campos^1^, Alice Angela Slomp^1^, Cristina Schnor Haygert^1^, Vania Naomi Hirakata^2^, Maria Lucia Rocha Oppermann^2^, Angela de Azevedo Jacob Reichelt^2^

##### ^1^Hospital Nossa Senhora da Conceição, Porto Alegre, Brazil; ^2^Hospital de Clínicas de Porto Alegre, Porto Alegre, Brazil

**Introduction:** Type 2 diabetes prevalence is growing in childbearing age women and its first detection is becoming common in pregnancy.

**Objective:** We aimed to describe the frequency of unrecognized type 2 diabetes whose diagnosis was made during pregnancy and to compare the profile of these women to those with diagnosis antedating pregnancy.

**Methods:** Retrospective cohort of Type 2 pregnant women (n = 349) evaluated in two Brazilian hospitals of the public health system, from May 2005 to December 2016. Data were collected from medical records. We compared clinical characteristics of women with diagnosis antedating pregnancy to those whose diagnosis was made during pregnancy, using student’s t test, Chi square and Mann–Whitney tests as appropriate.

**Results:** Diabetes duration was available for 348 women (99.7%, 95% confidence interval (CI) 0.98–1.00). Mean age was 33 ± 6.0 years; time since diagnosis in those with pregestational diabetes was 3 years (interquartile range 1–7 years); pregestational BMI was 34.0 ± 7.5 kg/m^2^ (n = 339); and 300 women (88.5%, 95% CI: 85–92%) had excessive weight (BMI ≥ 25 kg/m^2^) before pregnancy. Type 2 was first diagnosed in pregnancy in 109 women (31.3%, 95% CI: 26–36%). They fairly began specialized follow-up in the first trimester (10.7% compared to 29.7% in those with known previous diabetes, p < 0.001); most arrived in the third (53.4% compared to 28.8% of those with pregestational diabetes, p < 0.001). The comparison of main characteristics between the two groups showed that women with type 2 diabetes diagnosed in pregnancy had previous macrosomia more frequently (31.4% vs 19.1%, p = 0.019), began treatment later (25 weeks (interquartile range (IQR) 18–32) vs 18 weeks (IQR 12–26), p < 0.001) and had gained more weight at booking (5.7 ± 9.0 kg vs 3.5 ± 5.5 kg, p = 0.022), whereas chronic hypertension was more common in those with known pregestational diabetes (22.8% vs 12.7%, p = 0.025). Main maternal and neonatal outcomes were similar between the groups.

**Conclusions:** First diagnosis of diabetes during pregnancy was frequent in women with type 2 diabetes. Pregnancy outcomes were similar to those of women with known pregestational diabetes, even considering that they began specialized prenatal care later. Women with risk factors usually associated to type 2 diabetes deserve special attention to allow detection of hyperglycemia before or at least earlier in pregnancy.

### P271 Validation of treml4 mRNA and polymorphisms in blood leukocytes of type 2 diabetes mellitus patients with subclinical atherosclerosis

#### Victor Hugo Rezende Duarte^1^, Karla Simone Costa de Souza^1^, Adriana Bertolami Manfredi^2^, Mário Hiroyuki Hirata^3^, Rosário Dominguez Crespo Hirata^3^, André Ducati Luchessi^1^, Vivian Nogueira Silbiger^1^

##### ^1^Federal University of Rio Grande do Norte, Natal, Brazil; ^2^Dante Pazzanese Institute of Cardiology, São Paulo, Brazil; ^3^University of São Paulo, São Paulo, Brazil

*Diabetology & Metabolic Syndrome* 2019, **11(Suppl 1):**P271

**Introduction:** Type 2 Diabetes mellitus (T2DM) is risk factor for atherosclerosis (AT). The onset of AT may remain asymptomatic throughout life until an acute cardiovascular event occurs. Thus, detection of AT in its subclinical phase is a priority for primary prevention of coronary artery disease (CAD). In previous studies, our demonstrated that Trem-like transcript 4 (TREML4) mRNA expression was increased in blood leukocytes of patients with CAD, being a potential early biomarker of AT. Nevertheless, the hypothesis that T2DM is associated with increased TREML4 mRNA expression for the development of CAD still has not been tested.

**Objective:** We aim validate the polymorphisms and mRNA expression of TREML4 in subjects with T2DM and subclinical atherosclerosis (SA).

**Methods:** Two hundred and ninety-three individuals with AS, aged 29 to 75 years old, Diabetic (T2DM group) and non-diabetic (control group) were included (Ethics number: 3852). TREML4 polymorphisms [rs2803495 (− 101A > G) and rs2803496 (− 10C > T)] and TREML4 mRNA expression were analyzed by RT-PCR.

**Results:** Patients with SA carrying rs2803495 G allele (AG + GG) are more likely to express TREML4 than AA genotype carriers (OR = 13.31, 95%CI = 5.79–30.61, p < 0.001). However, rs2803495 variant was not associated with the degree of TREML4 (p > 0.05). Analysis of the rs2803496 variant showed that carriers of C allele (CT + CC) are more likely to express TREML4 than subjects carrying TT genotype (OR = 18.99, 95%CI = 7.60–47.44, p < 0.001). Moreover, C allele was associated with high mRNA expression levels (OR = 3.98, 95%CI = 1.67–9.48, p = 0.002). The rs2803495 (AG + GG) and rs2803496 (CT + CC) variants are not related to the development of T2DM (p > 0.05). The risk factor T2DMis not associated with the possibility of the subjects express TREML4 (p > 0.05). Despite this, patients who express TREML4 and are T2DM have higher expression levels of TREML4 when compared to the control group (OR = 2.6, 95%CI = 1.19–5.72, p < 0.001). T2DM patients with rs2803495 (AG + GG) genotypes are more likely to express TREML4 (OR = 20.77, 95% CI = 2.29–188.41, p = 0.007). Already, T2DM carriers of the rs2803496 variant (CT + CC) present similar risk to the control group to express TREML4 (p > 0.05).

**Conclusions:** The degree and expression of TREML4 was influenced by the presence of TREML4 polymorphisms. Moreover, the relation T2DM and polymorphisms contribute to the increase of TREML4 expression and consequently the cardiovascular risk.

**Financial support:** CNPQ and FAPESP.

### P272 Validity evidences of insulin injection checklists for health literacy assessment of children with type 1 diabetes (T1D)

#### Rebecca Ortiz La Banca^1^, Flávio Rebustini^3^, Willyane de Andrade Alvarenga^4^, Emilia Campos de Carvalho^2^, Mayara Lopes^5^, Lucila Castanheira Nascimento^2^

##### ^1^Joslin Diabetes Center - Harvard Medical School, Boston, USA; ^2^Escola de Enfermagem de Ribeirão Preto - USP, Ribeirão Preto, Brasil; ^3^Faculdade de Artes, Ciência e Humanidades - USP, SP, São Paulo, Brasil; ^4^Université du Québec enOutaouais, Quebec, Canada; ^5^Escola Paulista de Enfermagem - UNIFESP, São Paulo, Brasil

*Diabetology & Metabolic Syndrome* 2019, **11(Suppl 1):**P272

**Introduction:** The multiple insulin injections regimen is challenging for children with type 1 diabetes (T1D). The health care team must provide young people with T1D with abilities to safe self-injection. A child’s operational ability to perform an invasive procedure such as the subcutaneous injection is part of the expression of his/her health literacy (HL). However, despite the widespread discussion on the importance of developing LS in childhood, no instruments are currently available for assessing LS operational skills in children with T1D for self-injection.

**Objectives:** To search for validity evidences in two checklists on the syringe and pen injection technique to assess the HL operational skills of children with T1D.

**Methods:** We developed the syringe and the pen injection technique checklists. Both checklists comprised items from a systematic review. The response scale was built based on the polytomicss model, with the yes, no, and not applicable options. The panel of professional experts included those who oversee children with T1D on self-injections; HL specialists; and psychometrics specialists, experienced in developing measuring instruments. The panel evaluated both checklists regarding their clarity, objectivity and relevance. The content validity was assessed using the Content Validity Ratio (CVR).

**Results:** Eleven health care providers (72% nurses or physicians, professional experience 19.4 ± 10.1 years, endocrinology specialists—45%—or pediatrics—18%) completed the content assessment. The syringe checklist scored CVR = .90 and the pen checklist .86. Items containing the word homogeneity were considered inappropriate by the experts, as it is a word difficult to understand. Items regarding the needle insertion angle and the skin fold also did not reach the critical value for content validity. The final version of the checklist for syringe injection comprised 22 items with CVR = .91. The final version of the checklist for pen injection comprised 18 items and obtained CVR = .87.

**Conclusion:** The checklists presented clear, objective, and relevant content by the multiprofessional expert panel. Further research might investigate the checklists’ validity evidences when providers directly assess the skills of children with T1D for insulin self-injection.

### P273 Vascular age as a cardiovascular risk marker in asymptomatic patients with type 2 diabetes

#### Marilia De Brito Gomes, Catia Cristina Silva Sousa Vergara Palma, Pablo Moura Lopes, Eliete Leao Silva Clemente, Maria De Fatima Bevilacqua Da Matta, Caio Lima Correa, Camila Bahia Soares

##### Universidade do Estado do Rio de Janeiro, Rio de Janeiro, Brazil

*Diabetology & Metabolic Syndrome* 2019, **11(Suppl 1):**P273

**Introduction:** There is a wide variety of cardiovascular (CV) outcomes in patients with type 2 diabetes (T2DM), even in asymptomatic individuals. Since risk is multifactorial, the search for non-classical risk factors, such as thyroid function, may contribute to the refinement of these event predictions. The carotid intima-media thickness (CIMT) can be considered as a surrogate marker of atherosclerosis and is widely used to improve accuracy in the stratification of cardiovascular risk.

**Objective:** To evaluate the relationship between Vascular Age (VA) determined from CIMT, Framingham Risk Score (FRS), and thyroid function in asymptomatic patients with type 2 diabetes (T2DM).

**Methods:** Clinical, laboratory, and CIMT parameters were measured in 152 asymptomatic patients with T2DM. FRS was performed with chronological age(CA) and then with VA determined from CIMT. A multinomial logistic regression model was used to analyze variables related to CVR reclassification. The study protocol was approved by the Institutional Research Ethics Committee.

**Results:** The use of CIMT for the determination of VA led to the reclassification of 54 (35.52%) out of 152 asymptomatic T2DM patients, being 20 (37.03%) to a lower risk category and 34 (62.96%) to a higher risk category according to FRS. The variables that were associated to reclassification to a higher risk category were: family history (FH) of premature coronary artery disease (p = 0.046), FH of thyroid disease (p = 0.010), use of statins (p = 0.027), and free T4 (p = 0.009). None of the variables associated with glycemic control influenced the reclassification of cardiovascular risk.

**Conclusions:** In our study, VA determined from CIMT allowed the reclassification of the CVR in asymptomatic T2DM patients demonstrating an improvement of risk stratification and indicating possibly a more precise treatment in routine clinical practice. However, further prospective studies must be performed to evaluate thyroid function as a CV risk marker and establish the predictive values of CIMT on cardiovascular outcomes in asymptomatic patients with T2DM.

### P274 Vascular ultrasound evaluation of lower limbs in individuals with type 2 diabetes mellitus

#### Hélida Cristina Cirilo da Silva^1^, Maria Carolina Simões Vieira de Melo^1^, Alice Sá Carneiro Ribeiro^2^, Douglas Sousa Matos^1^, Diana de Andrade Silva^1^, Danilla Maria do Nascimento^1^, Izabelly Karina Santos Rodrigues^2^, Silvia Regina Arruda de Moraes^1^

##### ^1^Universidade Federal de Pernambuco, Recife, Brazil; ^2^Centro Universitário Estácio do Recife, Recife, Brazil

*Diabetology & Metabolic Syndrome* 2019, **11(Suppl 1):**P274

**Introduction and objectives:** In the pathogenesis of diabetic vascular disease, vascular dysfunction makes an important role, being generally a systemic disorder in which the peripheral arteries become referential for the evaluation of the functional capacity of the whole circulatory system, mainly due to the easy access (Int Angiol., 36, 354–361, 2017). However, to perform a better evaluation of the vessels there is a need for complementary tests, which are able to identify which systems or anatomical levels are involved in the pathological condition. The present study, still in progress, aims to evaluate arterial vascularization of the lower limbs in individuals with type 2 diabetes mellitus (DM2), determining and understanding the behavior of blood flow in the posterior tibial and dorsal artery of the foot.

**Methods:** A cross-sectional study with a sample of 13 individuals of both sexes, aged 45 years or over, diagnosed with DM2 for at least 3 years. Study approved by the Ethics and Research Committee, under CAAE: 84511518.8.0000.5208. The analysis of the blood flow was made with the Ultrasonic Vascular Doppler (BV-620VP, Guangdong, China/Mainland) with sinusoidal waveform and high frequency transducer (8 MHz ± 10%) for superficial vessels (posterior tibial and dorsal artery of the foot bilaterally). Three measurements were performed with a 30 s interval, considering the final result as the average of the three collections.

**Results:** In the results, we obtained for the dorsal artery of the foot an average of 45.69 cm/s (± 17.87) and 46.45 cm/s (± 15.58) for the arteries of the left and right lower limbs, respectively. Regarding the posterior tibial artery, 42.58 cm/s (± 8.69) and 46.47 cm/s (± 15.21) were obtained for the left and right lower limb arteries, respectively.

**Conclusion**: The results obtained until now, when compared to the values of non-diabetic individuals described in the literature, indicate a decrease in blood flow velocity, especially in the posterior tibial arteries, indicative of endothelial dysfunctions that leads to a reduction in the diameter of the vessel, suggesting problems with obstruction or lumen narrowing. However, it is necessary to obtain a total sample of this study so that there is a more consistent analysis of the results found.

### P275 Vitamin D use and DPP-4 inhibitor in preclinical phase of DM1: case report

#### Ana Cecília De Oliveira Resende, Camila Lôbo Andrade De Moraes, Mayara Rios Leite Macedo, JanniniValli Moreira, LayanaTyara Sandes Fraga, Gabriel Barreto Bastos, Loraine Albiero Pellucci, Michelle Patrocíneo Rocha

##### Hospital Santa Marcelina, São Paulo, Brazil

*Diabetology & Metabolic Syndrome* 2019, **11(Suppl 1):**P275

**Introduction:** Preservation of beta-cell reserve in newly onset DM1 by immunomodulation, in addition to the effect of incretins, appears to be possible with an association of DPP-4 inhibitors and vitamin D3.

**Case report:** DMO, female, 18 years old, white, with a history of hypothyroidism of Hashimoto and vitiligo. Patient was referred in 2016 to the endocrinology outpatient clinic with a history of gestational diabetes. She was in late puerperium (7 months) and denied polyuria, polydipsia or hospitalization for diabetic ketoacidosis. Had BMI: 21.7 kg/m^2^. 11/11/16: GJ 117 mg/dL, HbA1C 6.7% Insulin 8.9 µUi/ml and Vitamin D 29.6 ng/ml. 12/12/16: PPC 2.0 ng/mL (VR: 1.1 to 4.4) and Anti-GAD 35.5 IU/mL (VR < 10). On 10/05/17 it had: GJ 120 mg/dL, HbA1C 6.6% and PPC 1.28 ng/mL. Vitamin D was then started at a dose of 2,000 IU/day and then increased to 50,000 IU/week in combination with sitagliptin 100 mg/day. On January 29, 1919 patient returned with new test results: GJ 125 mg/dL, HbA1C 6.4%, Anti-GAD < 5 IU/mL, PPC 2.60 ng/mL and Vitamin D 78.2 ng/mL.

**Discussion:** DPP-4 inhibitors are capable of inhibiting lymphocytes with CD26, a protein with DDP-4 activity that regulates T cell development, migration and production of cytokines. In addition, it increases the incretinic effect on the beta cell through increase in GLP-1 half-life and consequent increase in insulin. Vitamin D regulatory activates T cells, dendritic cells inhibitors and B lymphocyte differentiation into plasma cells. These combined medications may improve the immune response in patients with recent onset DM1 and probably promote sustained clinical remission. Prolonged remission is possible in patients with recent onset DM1. Inhibition of CD26/DPP-4 and the incretin effect caused by sitagliptin associated with the immunomodulatory effect caused by vitamin may explain the beneficial effects observed in this case. The negativity of antibody levels in the case reported here, as well as in others reported in the literature, reinforces this hypothesis. Nevertheless, further studies of DPP-4 and vitamin D3 inhibitors in patients with T1DM should be conducted to confirm their safety and efficacy in these patients.

**Conclusions:** Vitamin D replacement and use of DPP-4 inhibitor may result in long-term preservation of β cells without decompensation in diabetic ketoacidosis or early decompensation, and there is no need for insulin in DM1 at this time.

Informed consent to publish had been obtained from the patient.

### P276 Waist-to-height ratio and cardiovascular risk in post-transplant diabetes patients

#### Maria Yasmin Paz Teixeira, Nathália Bernardo Marinho Leite, Mariana Pimentel Gomes Souza, Lorena Taúsz Tavares Ramos, Fábia Karine de Moura Lopes, Francisca Diana da Silva Negreiros, Synara Cavalcante Lopes, Renan Magalhães Montenegro Junior

##### Universidade Federal do Ceará, Fortaleza, Brazil

*Diabetology & Metabolic Syndrome* 2019, **11(Suppl 1):**P276

**Introduction:** Brazil is the second country in absolute number of kidney and liver transplants. Post-transplant diabetes mellitus is diagnosed in 10 to 40% of recipients. This public have an extremely high rate of cardiovascular complications, particularly those resulting from coronary artery disease.

**Objective:** To evaluate waist-to-height ratio and cardiovascular risk in patients with post-transplant diabetes.

**Methodology:** Cross-sectional and observational study conducted among 33 patients diagnosed with post-transplant diabetes, male and female, aged between 20 and 80 years, treated at the Nutrition service of a specialized outpatient clinic. Weight (kg) and height (m) were measured to obtain Body Mass Index (BMI-kg/m^2^) and waist circumference (WC) (cm) was also measured. Waist-to-height ratio (WHR) was applied. It is based on the assumption that the WC measurement is proportional to the individual’s body height measurement, with the presence of a universal cutoff of 0.5 to identify cardiovascular risk in both gender and in any age group. To assess the normality of quantitative variables, the Shapiro–Wilk test was used. Student’s t-test and Fisher’s exact test were applied for comparison and association, respectively. The level of significance was 5%. The research met international and national ethical standards involving human beings.

**Results:** Among the 33 evaluated, 23 underwent renal transplantation and 10 underwent liver transplantation. The mean age was 51 (± 12.5) years, with male predominance (63.64%). The average BMI was 26.8 (± 5.45) kg/m^2^. When evaluated by transplantation diagnosis, BMI was higher among kidney transplant recipients (p = 0.03). WHR was greater than 0.5 in 78.79% of the sample, indicating higher risks for obesity-related atherosclerotic cardiovascular diseases. This inadequacy was more prevalent among kidney transplant recipients (82.6%) (p > 0.05).

**Conclusion:** Anthropometric indicators, such as WHR, are considered useful to provide a good correlation with coronary events. WHR is presented as a strategy for cardiovascular risk assessment in post-transplant diabetes patients, presenting low cost and easy daily clinical application.

### P277 Which component of health-related fitness has the greatest correlation with glycemic control in physically active type 2 diabetic women?

#### Ricardo Azevedo Cavalcanti Aragão, Maria Elizabeth Queiroz Holanda Do Nascimento, Nathália De Souza Melo Abath Barbosa, Theo Victor Alves Soares Do Rêgo, Anthony Rodrigues De Vasconcelos, Jonathan Nícolas Dos Santos Ribeiro, Denise Maria Martins Vancea, Cláudio Barnabé Dos Santos Cavalcanti

##### Universidade de Pernambuco, Recife, Brazil

*Diabetology & Metabolic Syndrome* 2019, **11(Suppl 1):**P277

**Introduction:** There is evidence that high levels of physical fitness provide additional health benefits and promote the prevention, maintenance and improvement of functional capacity. These benefits can also be found in the diabetic population.

**Objective:** To verify, through correlation, which component of physical fitness has the greatest influence on glycemic control in physically active type 2 diabetic women.

**Methods:** Correlation research, pre-experimental (Ethics Commitment: 0008.0.097.000-09). Eight women with type 2 diabetes, with a mean age of 62.8 ± 10.2, members of the Supervised Physical Exercise Program for Diabetics of a Northeastern Public University, participated in the study. The training program was divided into aerobic exercises (dance), flexibility and strength exercises (alternate exercises by segment, using 2 sets between 10 and 20 repetitions with 1′ rest) lasting 4 months. Physical fitness components (maximal oxygen uptake, strength, body composition and flexibility) were performed before and after the intervention. Blood glucose monitoring was performed before and after each training session. Statistical analysis was performed using the Spearman correlation test.

**Results:** Data analysis showed a positive correlation only between flexibility and capillary blood glucose (Pre Intervention Flexibility = 97.3° ± 10.5° vs Pre Blood Glucose = 157.2 mg/dL ± 29.2 mg/dL; Post Intervention Flexibility = 100.8° ± 11.1° vs Post Blood Glucose = 117.1 mg/dL ± 19.5 mg/dL;—r = − 0.90) of type 2 diabetic women in this sample, i.e., the greater the flexibility the lower the capillary glycemia.

**Conclusion:** According to the results, the physical fitness component that showed a correlation with the decrease in glycemia was flexibility, which indicates that it is a primordial factor for glycemic control and, therefore, it is important to emphasize that the programs of exercise for diabetics prioritize flexibility training.

### P278 Which patients are most likely to achieve diabetes remission after bariatric surgery?

#### Roberto Hangley Mendes Dantas Júnior, Letícia Morais de Andrade, Marcel Catão Ferreira dos Santos, Mônica Raquel de Souza Aquino, Victória Teixeira Leite, Vinicius Silva Costa, Daniel Coelho

##### Hospital Universitário Onofre Lopes, Universidade Federal do Rio Grande do Norte, Natal, Brazil

*Diabetology & Metabolic Syndrome* 2019, **11(Suppl 1):**P278

**Introduction:** Bariatric surgery has been recognized as an important option in the management of type 2 diabetes mellitus, presenting better levels of glycemic control than those achieved with clinical treatment. Additionally, diabetes remission is feasible in patients undergoing this intervention.

**Objective:** To study outcomes related to glycemic control in patients undergoing bariatric surgery, as well as factors capable of predicting a greater chance of achieving total or partial remission of diabetes mellitus.

**Methods:** This is a retrospective longitudinal study based on analysis of medical records of a tertiary hospital. We included patients diagnosed with type 2 diabetes mellitus who underwent bariatric surgery and collected pre- and postoperative clinical, anthropometric and laboratorial data. The primary outcome analyzed was the occurrence of complete or partial remission of diabetes.

**Results:** A total of 97 patients were included in the study, 49 of whom underwent gastric bypass and 48 underwent vertical sleeve gastrectomy. Initially, the patients had a BMI of 42.45 (38.46–46.75) kg/m^2^, A1C of 6.4 (5.6–7.5) % and 89.7% were women. In addition, 18 (18.7%) of the patients used insulin. One year after the intervention, there was a significant weight loss [28.30 (21.95–35.60) kg] with a consequent decrease in BMI [31.18 (27.67–34.07) kg/m^2^, p < 0.001]. Regarding glycemic control, there was a decrease in A1C levels [5.4 (5.0–5.7) %, p < 0.001)], 82 (95.3%) patients reduced medication doses and 17 (94.4%) no longer had to use insulin. 91.8% of the evaluated patients achieved total (76.3%) or partial (15.5%) remission of diabetes mellitus. Patients with A1C levels below 6.8% before surgery had higher remission rates (96.7% vs. 83.3%, p = 0.049, OR: 5.900), as well as those who did not use insulin (97.5% vs. 66.7%, p < 0.001, OR: 19.250) and who lost more than 25 kg after 12 months (96.8% vs. 82.4%, p = 0.021, OR: 6.536).

**Conclusions:** Diabetes remission is a common outcome during the follow-up of patients undergoing bariatric surgery, occurring more frequently in patients with better previous glycemic control, no previous insulin therapy and greater weight loss.

### P279 Why diabetes is much more frequent among women in Brazilian Indians? The bororo and xavante experience with the emergence of diabetes

#### Laercio Joel Franco^1^, Amaury Lelis Dal Fabbro^1^, Nayara RagiBaldoni Couto^1^, Luciana Ferreira Franco^2^, Regina Santiago Moisés^2^, João Paulo Botelho Vieira Filho^2^

##### ^1^Faculdade de Medicina de Ribeirão Preto - Universidade de São Paulo, Ribeirão Preto, Brazil; ^2^Escola Paulista de Medicina - Universidade Federal de São Paulo, São Paulo, Brazil

*Diabetology & Metabolic Syndrome* 2019, **11(Suppl 1):**P279

**Background:** Reports about elevated frequency of diabetes in American Indians focused mainly the North and Central America. Information about diabetes among Brazilian Indians are scarce and the existing ones report a low prevalence. The Bororo population belong to the Macro Jê linguistic group and are in permanent contact with the Brazilian society since the beginning of the XXth Century, and live in the scrubland of Central Brazil. The Xavante Indians live in the same neighborhood, also from the Macro Jê linguistic group, but started to be in permanent contact with the Brazilian society in a process that begun in 1957. Previously, diabetes was an unknown disease among them and the first described case was in 1997 in a Xavante women.

**Aim:** To analyze sex differences in the prevalence of diabetes in the adult Bororo and Xavante population.

**Methods:** Blood pressure, weight, height, waist circumference, 75 g oral glucose tolerance test, HbA1c and lipid levels were measured in 152 (70 women) adult Bororo (84% of the target population) and in 948 Xavante (485 women) (75% of the target population).

**Results:** The overall age-adjusted prevalence of diabetes (WHO criteria), with 95% confidence interval for the Bororo was 9.1% (7.4–12.1), being absent among men and 20.0% (15.2–24.8) among women; for the Xavante was 28.2% (25.3–31.1), being 18.4% (14.9–22.2) for men and 40.6% (36.2–45.1) for women. The prevalence of obesity (BMI ≥ 30 kg/m^2^) among Bororo was 20.1% in men and 40.0% in women; in the Xavante was 52.6% for men and 48.7% for women. Among women, the prevalence of diabetes increases with increased BMI only in the Bororo group.

**Conclusion:** Brazilian Indian women are at higher risk to develop diabetes during the process of integration into the Brazilian society. Reasons for this gender differences are still unknown.

**Financial Support:** FAPESP (Proc. 17/20500-9).

### P280 Wilms tumor protein 1 in urinary extracellular vesicles: a new early biomarker of kidney injury in new-onset diabetes after kidney transplantation?

#### Karla Simone Costa de Souza^1^, César Endrigo Silva de Andrade^2^, Renato Ferreira de Almeida Junior^1^, Ony Araújo Galdino^1^, Maurício Galvão Pereira^2^, José Bruno de Almeida^2^, Marcela Abbott Galvão Ururahy^1^, Adriana Augusto de Rezende^1^

##### ^1^Department of Clinical and Toxicological Analyses, Federal University of Rio Grande do Norte, Natal, Brazil; ^2^Department of Integrated Medicine, Federal University of Rio Grande do Norte, Natal, Brazil

*Diabetology & Metabolic Syndrome* 2019, **11(Suppl 1):**P280

**Introduction:** New-onset diabetes after kidney transplantation (NODAT) is a serious metabolic complication leading to graft loss. Currently, the noninvasive marker used in the evaluation of kidney injury (KI) is the urinary albumin/creatinine ratio (ACR), however, this marker is nonspecific and only evaluates KI belatedly. Thus, it is necessary to search for early biomarkers, more specific for diagnosis. Recently, urinary extracellular vesicles (uEVs) have been studied as a rich source of biomarkers that are released from all nephron segment, including podocytes, one of the primary targets of KI in NODAT. Among the early biomarkers of KI in NODAT found in the uEVs is Wilms’ Tumor protein 1 (WT1), a transcription factor involved in nephrogenesis and podocyte differentiation, which is negatively regulated in case of podocyte damage.

**Objective:** To evaluate the potential role of WT1, isolated from uEVs, as a specific noninvasive marker of KI in the NODAT.

**Methods:** From Aug. 2015 to Nov. 2018, 39 kidney transplant (KTx) patients, who were in 6-months post-KTx, aged above 18 years were evaluated (Ethics Number: 1.144.405). Patients with previous history of *Diabetes mellitus* or segmental and focal glomerulosclerosis were excluded. Patients were divided into 2 groups according to the diagnosis of NODAT (non-NODAT and NODAT groups). Clinical data, fasting blood glucose, glycated hemoglobin, serum ureia, serum creatinine and ACR were determined. Estimated GFR was evaluated by the Chronic Kidney Disease Epidemiology Collaboration equation. uEVS were isolated by ultracentrifugation and WT1, by Western Blot. WT1 bands density were normalized by urinary creatinine.

**Results:** Increased WT1/creatinine ratio was found in the NODAT group when compared to the non-NODAT (p < 0.001). Regarding ACR values, no statistically significant differences were observed. Patients of the NODAT group, who released WT1 in uEVs presented with a reduced eGFR (< 42 mL/min/1.73 m^2^). In the ROC analysis, high AUROC (area under the ROC) values for prediction of proteinuria were observed for WT1/creatinine ratio (AUROC = 0.946; p < 0.001).

**Conclusions:** This study is the first to show increased expression of WT1 released in uEVs in patients with NODAT, while ACR was not increased yet. These results associated with the ones obtained in the ROC analysis and for the eGFR suggest that WT1 obtained from uEVs, can become an important diagnostic tool for the early detection of KI in the NODAT.

**Financial support:** CNPq.

